# Organophosphides: A New Class of Luminophore Ligands for Copper(I) Carbene Based TADF Emitters and Photocatalysts

**DOI:** 10.1002/anie.202518530

**Published:** 2026-02-25

**Authors:** Paul C. Ruer, Julian J. Holstein, Andreas Steffen

**Affiliations:** ^1^ Department of Chemistry and Chemical Biology TU Dortmund University Dortmund Germany

**Keywords:** Circularly polarized luminescence, Copper, Phosphide, TADF, Visible light Photocatalysis

## Abstract

Luminescent carbene copper(I) charge transfer complexes are promising candidates as molecular materials for photonic applications. Apart from steric and electronic modification of the acceptor carbene, most of the research has been dedicated to amide donor ligands to control the luminescence properties, while the remaining pnictogen group as anionic electron donating ligands is photophysically underrepresented. Herein, we demonstrate that dimesityl phosphide (Mes_2_P–) as a heavier homologue in [Cu(cAAC)(PMes_2_)] (cAAC = cyclic amino(alkyl) carbene) gives rise to orange emission with quantum yields of up to *ϕ*_max_  = 0.52 in the solid state that is bathochromically shifted by ∼3000 cm^−1^ in comparison to related amide complexes due to the lower electronegativity of phosphorus. Time‐resolved variable temperature studies reveals that the µs‐lifetimes and radiative rate constants of ca. *k*_r_ *=  *5 ⋅ 10^4^ s^−1^ are due to thermally activated delayed fluorescence (TADF) as the dominating emission mechanism at room temperature. In polystyrene matrices, the complexes exhibit environment dependent chiroptical properties (up to *g*_lum_  =  10^−2^, *B*_CPL_  =  1.31 M^−1^ cm^−1^, *k*_CPL_  =  41.5 s^−1^) and are efficient blue light photocatalysts for hydrophosphination of alkynes in solution, highlighting the potential of heavier pnictogen ligands for photonic materials.

## Graphical abstract

Dimesitylphosphide as a heavier congener of established amide ligands for charge‐transfer copper(I) complexes leads to bathochromic shift of the emission by up to 3645 cm^−1^ into the orange‐red (solid, polymer films) and even NIR (solution). The thermally activated delayed fluorescence exhibits high dissymmetry factors of g_lum_ = 10^−2^ in polystyrene. An inner‐sphere visible‐light photocatalytic mechanism could be exploited for hydrophosphination.

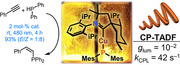

## 1 Introduction

Current research on luminescent transition metal complexes (TMC) focuses on the use of highly abundant 3d transition metals as potential substitutes for photoactive compounds based on precious 4d and 5d elements [1, 2, 3, 4, 5, 6, 7, 8]. The latter can mediate beneficial spin‐orbit coupling (SOC) [9, 10, 11] to populate triplet excited states for photocatalysis, singlet oxygen sensitization and photodynamic and photoactivated therapy (PDT/PAT) [12, 13, 14], but also for formal spin‐forbidden phosphorescence T_1_→S_0_. One of the challenges is that 3d compounds are often prone to potential premature deactivation via the metal‐centered (MC) dd* transitions, which activate the M–L bond vibrational modes, or they exhibit only insufficient SOC, leading to relatively slow and inefficient spin‐flip processes. However, great efforts of creative ligand design have allowed to access emissive complexes of V, [15, 16] Cr, [17, 18, 19, 20, 21] Mn, [21, 22, 23, 24, 25] Fe, [26, 27, 28, 29, 30] Co, [31] Ni, [32, 33, 34, 35] Zn [36, 37, 38, 39, 40, 41, 42], and Cu [43, 44, 45, 46, 47, 48, 49, 50, 51, 52, 53, 54]. The latter is the most widely studied first row transition metal for photonic applications due to its d^10^ electron configuration and resulting absence of MC states. In addition, the convenient oxidation potential of the Cu^I/II^ pair leads to the formation of low energy MLCT states, increasing the density of states and providing SOC so that formally spin‐forbidden ISC processes S_n_→T_m_ become fast up to a few ps.

In the last decade, linearly heteroleptically coordinated Cu^I^ carbene complexes with a donor‐acceptor (D–Cu–A) structure have been extensively studied [52, 53, 54, 55, 56]. This class of emitters provides access to low energy ligand‐to‐ligand charge transfer states (LLCT) in the visible region of the electromagnetic spectrum (EMS), which often results in thermally activated delayed fluorescence (TADF) as the dominating emissive path. The LLCT nature of the excited states lower the singlet‐triplet energy gap Δ*E*_ST_ by increasing the charge separation so that thermal population of higher vibronic states of the T_1_ leads to reverse (r)ISC into the S_1_ state and formation of an excited state equilibrium at ambient temperature [57]. The S_1_ excitons can efficiently decay by spin‐allowed fluorescence circumventing the slow, spin‐forbidden phosphorescence. Sophisticated ligand design allows for dedicated fine‐tuning of the TADF‐relevant parameters [45, 52, 54, 56, 57, 58, 59, 60, 61] and highly efficient emitters with emission kinetics and efficiency competitive to commercially employed Ir^III^ compounds have been obtained [44].

In such D–Cu–A compounds, the carbene ligands typically serve as acceptor moieties, and modification of their electrophilic character could be exploited to achieve deep red to NIR emission of interest for future IT applications or NIR OLEDs [45, 62, 63, 64]. Such low energy emission can also be realized by modification of the π‐basicity of the donor ligand, for which aromatic amides have been used as the most common motif because of their delocalization of the anionic charge enhancing the CT nature of the excited states, which is beneficial for the TADF process. Further increasing the π‐basicity of the amide moiety by employing phenazine derivatives indeed leads to a significant decrease of the emission energy. Interestingly, and to the best of our knowledge, heavier congeners of the pnictogens have not been considered yet for deep red luminescent coinage metal complexes although one would expect them to be more potent donor ligands due to their higher polarizability.

Photophysical investigation of phosphide complexes of coinage metals, especially of copper, was impeded by their tendency to form a wide variety of oligomers or clusters depending on the steric and electronic influences both of the phosphide as well as of the neutral ligands. For example, *Tuck* reported that the electrolysis of an acetonitrile solution containing both diphenylphosphine and bis(diphenylphosphino)methane (dppm) as a neutral tertiary phosphine ligand yields crystalline tetranuclear [Cu_4_(dppm)_4_(μ‐PPh_2_)_4_] [65, 66]. The presence of non‐chelating phosphines in the reaction of CuCl with Me_3_SiPPh_2_ can lead to clusters of the general formula [Cu_m_(PR_3_)_n_(μ‐PPh_2_)_q_] with great structural diversity as demonstrated by *Fenske* et al. [67] *Waterman* and coworkers investigated Cu‐catalyzed hydrophosphination reactions and in the course of their work they isolated [Cu_4_(P(*t*‐Bu)_3_)_2_(μ‐PPh_2_)_4_] as efficient pre‐catalyst [68]. Other neutral chelating ligands such as phenanthroline (phen) were used to stabilize defined complexes, such as [Cu_3_(phen)_3_(μ‐PPh_2_)_3_], which showed to be synthetically useful in the preparation of highly functionalized alkyl phosphines and arsines [69] The bridging properties of the phosphido ligand could be attenuated by classical protection in the form of a borane adduct yielding the tetrahedral monocopper(I) complex [Cu(phen)(PHPh_2_)(PPh_2 _⋅ BH_3_)] [70]. The first synthesis of linear phosphide Cu^I^ complexes that are structurally related to the above mentioned TADF‐active systems was reported by *Whittlesey*, *Nolan* and *Cui* with classical imidazole‐based *N*‐heterocyclic carbene (NHC) ligands *via* internal base method, *σ*‐bond metathesis and one pot reaction [71, 72, 73, 74]. However, it is important to note that the coordination geometries are dependent on the steric demand of the carbene and can lead to monomeric, dimeric or trimeric structures [73, 74]. Interestingly, linear [Cu(IDipp)(PPh_2_)] (IDipp = Bis(2,6‐diisopropyl)imidazolylidene) has been shown to photocatalyse the hydrophosphination reaction of styrene derivatives with HPPh_2_ when irradiated with 360 nm UV light [75]. Linear, monomeric [Au(cAAC)(PPh_2_)] was reported to be a nucleophilic superbase undergoing selective complexation of various metal fragments to form compounds of the type [(cAAC)Au(μ‐PPh_2_)Rh(acac)(CO)] [76]. Based on the demonstrated accessibility of defined linear complexes and bearing in mind the higher polarizability (and thus higher π‐basicity) of the heavier pnictogens in comparison to commonly used nitrogen‐based donors, we were interested in the influence of phosphide donors on the CT excited states and the resulting photophysical properties.

## 2 Results and Discussion

### 2.1 Synthesis and Characterization

**SCHEME 1:**
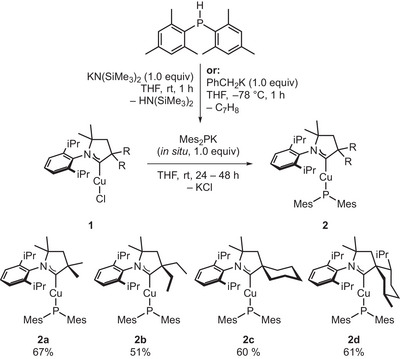
General synthesis of the complexes of type [Cu(^R^CAAC)(PMes_2_)] (**2a‐d**) by salt metathesis using [Cu(cAAC)Cl] (**1a‐d**) as precursor. Mes = 2,4,6‐Me_3_C_6_H_3_.

The linearly coordinated copper(I) phosphido complexes [Cu(^R^cAAC)(PMes_2_)] were prepared by salt metathesis starting from the respective chlorido complexes and in situ formed potassium dimesitylphosphide (Scheme 1). Single Crystals of **2a–2c** were obtained by slow vapor diffusion of *n*‐pentane into a solution of the complexes in diethyl ether/THF (3:1) at –35°C, while **2d** was crystallized by cooling a saturated solution in *n*‐pentane to –35°C. The chiral complex **2d** was obtained enantiopure from chiral and enantiopure **1d**. All complexes were fully characterized by multinuclear NMR spectroscopy, single crystal X‐ray diffraction (SCXRD) studies, high resolution mass spectroscopy (ESI‐HRMS), elemental analysis, and mid‐infrared (MIR) absorption spectroscopy (see Supporting Information). Noteworthy, the high steric demand of the mesityl substituents is crucial for the stability of the desired compounds as analogous attempts to synthesize linear [Cu(cAAC)(PPh_2_)] complexes led to decomposition in solution after several days and during crystallization experiments. However, the isolated PMes_2_ complexes **2a‐d** are highly reactive towards oxygen (see below), water, acetonitrile, and dichloromethane.

**FIGURE 1:**
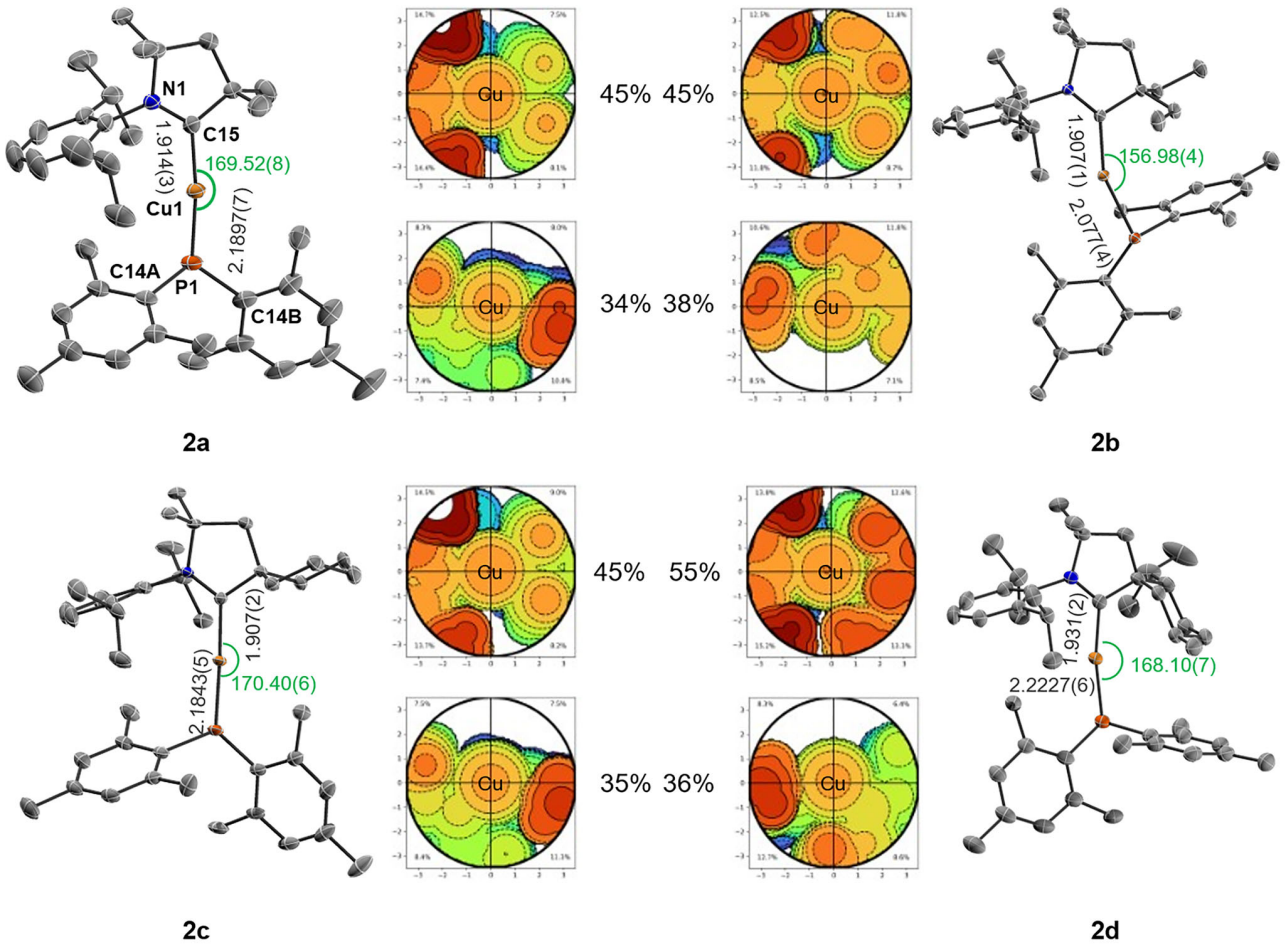
Molecular structure of **2a‐d** in the solid state determined by SCXRD (ellipsoids given for 50% probability; hydrogen atoms omitted for clarity) and steric maps showing the buried volume *V*_bur_ in % for the carbene (top) and phosphide (bottom) ligands, respectively. The numbering scheme applies to all structures [79].

The successful coordination of the PMes_2_ is confirmed by a ^31^P NMR resonance between –75.5 ppm and –76.1 ppm with a large halfwidth of 7.0 Hz due to coupling with the quadrupole moment of the ^63^Cu nucleus. The ^13^C resonance of the carbene atom is slightly shifted towards lower field in **2a‐d** compared to the chlorido complex **1a** (e.g. for **2a**, 252.1 vs. 248 ppm) [77], and appears as a doublet (^2^ *J*_P,C_  =  34.9 – 38.3) due to coupling to the ^31^P nucleus, similar to related NHC complexes [74]. We note that the coordination of PMes_2_ does not lead to an increase of π‐back‐bonding Cu^I^ → cAAC in the ground state as the ^15^N NMR data are very similar to those reported for [cAAC⋅LiOTf] (–159.9 ppm) or protonated cAAC⋅H^+^ (–148.1 ppm) [78]. Complexes **2a‐c** crystallize in a monoclinic lattice with centrosymmetric space group *P*2_1_/*n* with 4 molecules per unit, while **2d** crystallizes in the monoclinic space group *P*2_1_ with 6 molecules per unit and equimolar amounts of *n*‐pentane [79]. The presence of *n*‐pentane was also confirmed by ^1^H and ^13^C{^1^H} NMR spectroscopy. In all complexes, the bond length between the carbene carbon atom and the copper atom is slightly elongated by ca. 0.03–0.05 Å in comparison to the respective chloride complexes **1** (e. g., 1.9136(25) Å for **2a** vs. 1.878(2) Å for **1a**, see Figure 1) due to the electron donating effects of the PMes_2_ ligand. The Cu–P bond distances in **2a** and **2c** of 2.1897(7) and 2.1843(5) Å, respectively are shorter than found in structurally related [Cu(IDipp)(PPh_2_)] (2.2076(6) Å) [74], whereas it is comparable in **2b** (2.2077(4) Å) and elongated in **2d** (2.2222(5) Å), which can be explained by the varying repulsion of the ligands. Depending on the steric influence and flexibility of the substituents at the quaternary carbon of the cAAC ligand, the coordination geometry of the Cu^I^ center as well as the conformation of the phosphide change dramatically. While for **2a**, **2c,** and **2d**, a nearly linear ligand arrangement is observed in the solid state (C–Cu–P angle 168–170°), compound **2b** shows pronounced bending (156.98(4)°) presumably due to packing effects as the buried volume of the carbene ligand in **2b** of 45% is identical to **2a/c**, while the menthyl substituent in **2d** provides larger coverage of the copper center with 55% (Figure 1). The bending of the PMes_2_, indicated by the angle between the C14–P–C14 and the N–C15–Cu planes, strongly depends on the steric influence of the cAAC ligand, leading to only 38° for **2a** and **2c**. In contrast, the freely rotating ethyl groups in **2b** as well as the sterically rigid spiromenthyl substituent in **2d** increase the angles between the planes to 51°, which correlates with the emission properties and kinetics (see below).

### 2.2 Optoelectronic Properties

**FIGURE 2:**
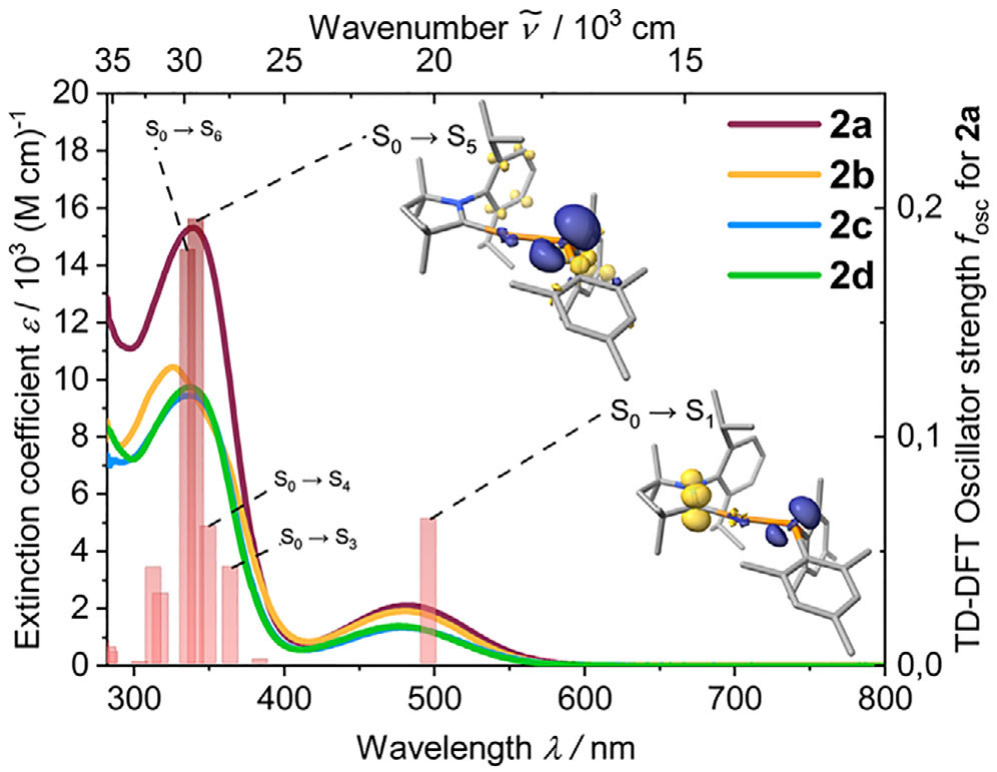
Absorption spectra of compounds **2a–d** in toluene solution (solid lines), TD‐DFT (D3BJ‐PBE0/ZORA/def2‐TZVP) calculated oscillator strengths of the first 11 vertical S_0_→S_n_ transitions (red bars) and depiction of the electron density difference between the S_0_ and S_1_/S_5_ states for **2a** in geometry optimized ground state structure.

The compounds appear as bright yellow (**2b**, **d**) to orange red (**2a**, **c**) solids depending on the cAAC ligand, and their solutions in THF, benzene or toluene are deep orange. The UV/vis absorption spectra of **2a‐d** in toluene solution are dominated by two major broad bands (Figure 2). The weakly allowed low energy absorption at λ_max_ = 478‐484 nm with extinction coefficients of *ε* = 1,400 – 2,000 M^−1^ cm^−1^ can be assigned to a LLCT p_P_+π_Mes_ → π*_CN_(cAAC) transition with some MLCT (σ_PCu_ → π*_CN_(cAAC)) admixture according to our TD‐DFT calculations performed for **2a**. We note that the experimental values for *ε* represent an average over all available conformational geometries due to rotation around the Cu–L σ bonds. In comparison to previously reported [Cu(^Ment^cAAC)(NPh_2_)] (**A**) absorbing at λ_max_ = 440 nm [44], the lowest energy absorption is bathochromically shifted in **2a–d**. The more intense high energy bands at λ_max_ = 326‐339 nm (*ε* = 9,900–15,300 M^−1^ cm^−1^ for **2b–d**) are the result of allowed LLCT and ILCT(phosphide) (S_0_ → S_5_ and S_0_ → S_6_) transitions overlapping with less allowed MLCT and LLCT transitions (S_0_ → S_3_ and S_0_ → S_4_). Interestingly, the less sterically encumbered **2a** exhibits a much higher extinction coefficient (*ε* = 15,300 M–^1^ cm–^1^) for this band in comparison to the other derivatives.

**FIGURE 3:**
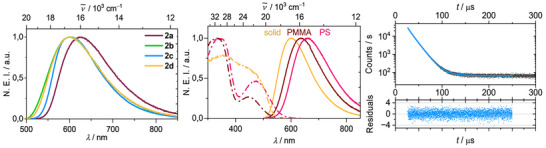
Normalized emission intensity (NEI) of compounds **2a‐d** in the solid state at room temperature (left), emission and excitation spectra of **2d** in different media (middle) and exemplary excited state lifetime analysis (in counts per second) of compound **2c** (λ_ex_ = 450 nm, λ_em_ = 600 nm) in the solid state at 297 K showing a multiexponential decay (right).

The phosphide complexes exhibit weak near‐IR photoluminescence with *λ*_max_ = 784 nm–818 nm upon irradiation at 480 nm in toluene solution, which is drastically bathochromically shifted by > 200 nm in direct comparison with the lighter *N*‐congener **A** (Figure S104 and Table S2) [44]. However, bright orange emission at *λ*_max_ = 625 and 600 nm for **2a** and **2b‐d**, respectively, with photoluminescence quantum yields of *ϕ* = 0.30–0.52 is observed in the solid state at room temperature (Figure 3 and Table 1). The large Stokes shift of Δ*ν̃*≈4200 cm^−1^ in combination with the broad spectral appearance indicate pronounced structural reorganization in the excited state of dominantly LLCT character. Interestingly, the emission of **2a** is slightly red shifted in comparison to the other derivatives.

**TABLE 1:** Selected photoluminescence data of the phosphide complexes **2a‐d** in the solid state, PS and PMMA (2 wt %) and toluene solution upon excitation at 480 nm (steady state) or 450 nm (time resolved).

Cpd.	Medium	*T* / K	*λ*_em_ /nm	*Φ*	<*τ*>^a^ /µs	<*k*_r_>^a^ /10^3^ s^−1^	Cpd.	Medium	*T* / K	*λ*_em_ / nm	*Φ*	<*τ*>^a^ /µs	<*k*_r_>^a^ / 10^3^ s–^1^
**2a**	solid state	297	626	0.47	7.91	59.0	**2c**	solid state	297	600	0.52	12.0	43.3
		77	612	0.35	1085^b^	0.32			77	610	0.39	1079^b^	0.36
	PS	297	682	0.04	1.51	26.5		PS	297	677	0.05	2.55	19.6
	PMMA	297	635	0.07	2.56	27.3		PMMA	297	655	0.07	2.79	25.1
	toluene	297	785	n/d	n/d	n/d		toluene	297	804	0.001	n/d	n/d
**2b**	solid state	297	601	0.30	16.5	18.8	**2d**	solid state	297	602	0.45	13.5	31.1
		77	652	0.14	264	0.53			77	650	0.31	433	0.72
	PS	297	679	0.01	1.62	6.17		PS	297	660	0.05	5.99	8.3
	PMMA	297	655	0.07	1.63	42.9		PMMA	297	636	0.07	8.15	8.5
	toluene	297		n/d	n/d	n/d		Toluene	297	789	0.001	n/d	n/d

^a^ Amplitude weighted values as the result of a multiexponential decay (Table S2 for contributions).

^b^ Monoexponential decay observed.

**FIGURE 4:**
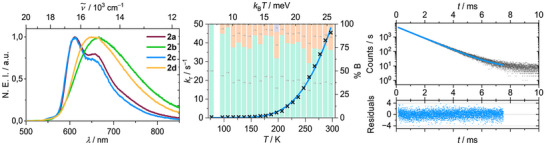
Normalized emission of compounds **2a‐d** in the solid state at 77 K (left), tr‐VT data of **2c** showing the temperature dependence of the average *k*_r_ and relative contributions of the observed lifetime components to the multiexponential transient decays (middle, values not obtained for 87 K, see also Supporting Information), and mono‐exponential decay (in counts per second) observed for **2c** (λ_ex_ = 450 nm, λ_em_ = 600 nm) at 77 K (right).

The transient luminescence decays are of bi‐ or tri‐exponential nature and their deconvolution gives averaged lifetimes in the µs regime, which suggests that spin‐forbidden processes are involved (Figure 4 and Table S3). The mean radiative rate constants <*k*_r_> are similar for complexes **2a** (5.9⋅10^4^s^−1^), **2c** (4.3⋅10^4^s^−1^) and **2d** (3.1⋅10^4^s^−1^). However, **2b** exhibits a much lower radiative efficiency (*ϕ* = 0.30, *k*_r_ = 1.9⋅10^4^s^−1^), which may be due to the bend structure in the single crystalline solid state (Figure 1), reducing orbital overlap between the donor and acceptor ligands and, consequently, reducing the oscillator strength *f*. The emission in different environments was investigated for compound **2d**, showing a bathochromic shift in comparison to the solid state when doped in polar PMMA (*λ*_max_ = 636 nm), and even more in non‐polar poly(styrene) (PS) (*λ*_max_  =  660 nm) films (Figure 3 and Table 1). A similar relative trend is observed for the excitation spectra, which suggests that the ground state of **2d** is more polar than the emitting excited state. Again, we notice that the luminescence of **2d** in PS is bathochromically shifted by 130 nm (3645 cm^−1^) compared to **A**, highlighting the influence of the heavier pnictogen [36, 37, 38, 39, 40, 41, 42]. Surprisingly, nonradiative decay in the polymer films is very facile and *k*_r_ is significantly reduced in comparison to the solid‐state measurements for all compounds due to a less efficient TADF process (Table 1). In the solid state, the phosphide complexes are very densely packed and the conformation fixed, providing optimal prerequisites for TADF. However, the higher conformational flexibility of **2a‐d** in viscous polymers and intermolecular interactions between the emitter complexes and the environment apparently influence the excited state coupling and, according to the energy gap law at lower emission energies, enhance vibrational coupling of the S_1_ state with the ground state.

As d^10^ coinage metal complexes can emit from both their S_1_ and T_1_ states, we were curious whether TADF or phosphorescence is the major radiative path and thus we carried out steady‐state and time‐resolved variable temperature luminescence measurements (Figure 4). Upon cooling **2a‐d** to 77 K in the solid state, the emission onset of all compounds is bathochromically shifted to 550 nm indicating a different lower energy state to be responsible for the luminescence than at 297 K. However, the spectral appearance reveals for **2a** and **2c** a vibrational progression, but remains broad and featureless for **2b** and **2d**. We attribute this to a more rigid solid‐state structure favoring certain vibrational modes for compounds **2a, c**. For all complexes, a significant increase of the luminescence lifetime up to the millisecond range with concomitant decrease of *ϕ* is observed, resulting in a drastic decrease of <*k*_r_> between 297 K and 77 K (Table 1). Such a behavior hints at TADF as the dominant emission mechanism at room temperature. Fitting *k*_r_ to the Boltzmann distribution function for a three‐state‐model (Equation (1)) [44] indeed reveals a sigmoidal temperature dependence:

(1) $$k_{r}=\frac{3k_{P}+k_{F}\cdot \exp\left(-\frac{\Delta_{\mathrm{ST}}E}{k_{b}T}\right)\;}{3+\exp\left(-\frac{\Delta_{\mathrm{ST}}E}{k_{b}T}\right)}$$

with the beginning of a plateau at room temperature, while at T < 150 K, no significant further decrease of *k*_r_ is observed, indicating pure phosphorescence from the T_1_ state. The relatively large Δ*E*_ST_ = 139 ± 5 meV (1119 cm^−1^) nicely coincides with the values obtained from the energetic shift of the emission onset at 297 and 77 K (Figure S107) and can be explained by the spatial proximity and more localized nature of the frontier orbitals involved in the vertical transitions (Figure 2), in contrast to structurally related carbazolate and diarylamide complexes, where the HOMO is delocalized over the entire π system [44].

### 2.3 Chemiluminescence

**FIGURE 5:**
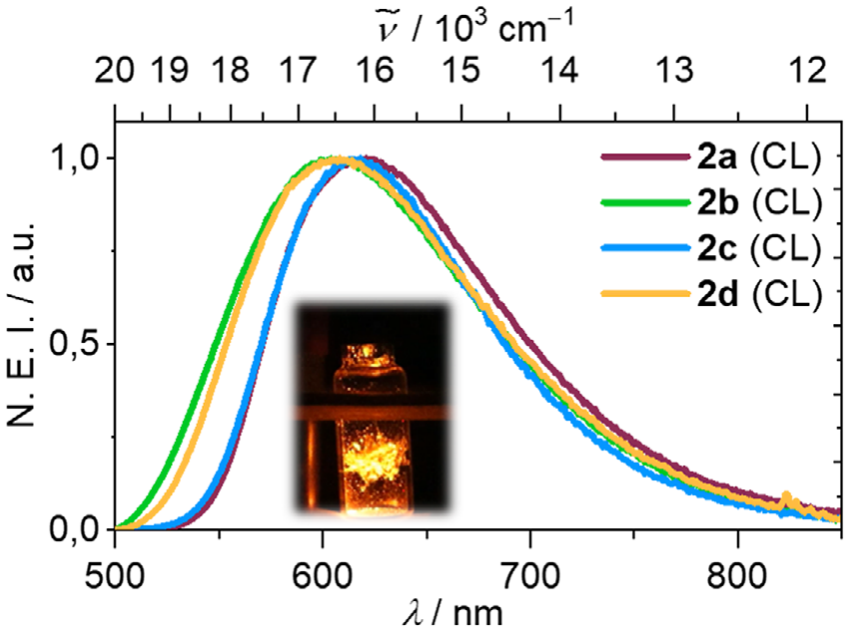
Normalized chemiluminescence spectra of **2a‐d** in the solid state upon reaction with atmospheric oxygen.

During the preparation and photophysical investigation of **2a‐d**, we inadvertently discovered orange chemiluminescence in the solid state as a result of a chemical reaction upon contact with air (Figure 5 and Video SI). Further studies showed that **2a‐d** do not react with N_2_O as an oxidizing agent even after several days, but exposure to dry molecular O_2_ gave immediate intense luminescence and yielded a white solid, which is insoluble in most standard solvents. ^31^P NMR spectroscopic studies in dry 1,2‐difluorobenzene showed one narrow singlet at –5.73 ppm, and HR‐MS analysis of the reaction product indicates the formation of, Cu(I)‐diarylphosphinites and Cu(I)‐diarylphosphinates supported by a cAAC ligand, reprotonated carbene and tetramesityldiphosphine (Figure S95). Unfortunately, attempts to isolate or crystallize the reaction products for further characterization were not successful. However, it is important to note that the chemiluminescence also occurs when PMMA or PS films doped with **2a‐d** are exposed to air and is not related to differences in the steric protection of the copper(I) center as **2d** with the highest *V*_Bur_ is equally reactive as the other derivatives (Figure 1). These properties are highly unusual, as we are not aware of any previously reported copper(I) amide analogs or copper(I) phosphide clusters displaying chemiluminescence upon oxidation, which appears to be due to the higher reactivity of the phosphide ligands coordinated to the electron‐rich copper(I) cAAC fragment in comparison to other typically employed donor ligands.

### 2.4 Chiroptical Properties

**FIGURE 6:**
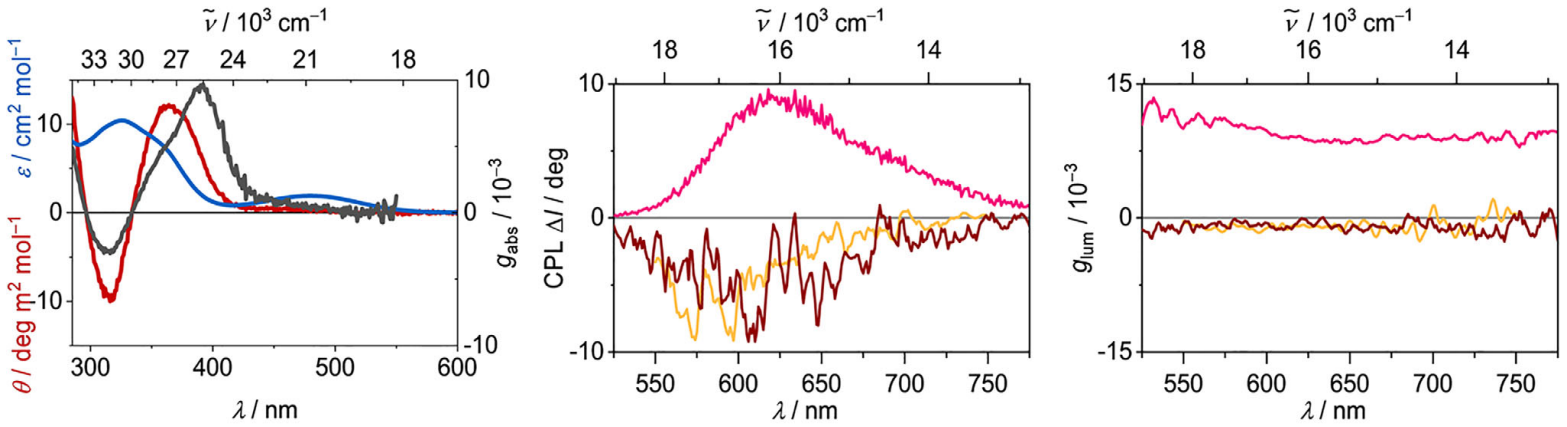
Left: CD (red) and absorption spectrum (blue) of compound **2d** in toluene at room temperature with absorption dissymmetry (grey). Middle: CPL‐spectrum of compound **2d** in solid state (yellow), in PMMA matrix (brown) and in PS matrix (pink). Right: Emission dissymmetry of the compound **2d** (*λ*_ex_ = 450 nm).

Given the current interest in chiroptical properties for photonic applications, we further investigated in this regard the chiral and enantiopure complex [Cu(^Menth^cAAC)(PMes_2_)] (**2d**) in different media. The CD spectrum in toluene (Figure 6) shows a relatively small absorption dissymmetry for the lowest energy transition (*g*_abs_≈10^−3^) and enhanced dissymmetry values of *g*_abs_ = 10 ^−2^ for higher energy transitions with small extinction coefficients ε. According to Equation (2) [51], the dissymmetry for the transition from state i to state f maximizes when the electronic (***μ***) and magnetic (***m***) transition dipole moment vectors are in antiparallel orientation. Our TD‐DFT calculations suggest an angle of 79° between ***μ*** and ***m*** for the S_0_→S_1_ transition, similar to related [Cu(cAAC)(Cz)] derivatives, [50] while the prominent CD bands at higher energy are due to or for symmetry‐forbidden electronic transitions with small ***μ*** and high ***m***.

(2) $$g_{i\to f\;}=4\;\frac{\left|\mu \right|\left|m\right|\;}{{\left|\mu \right|}^{2}+{\left|m\right|}^{2}}\cdot \cos\left(\theta \right),\theta =∠\left(\mu ,m\right)$$

**FIGURE 7:**
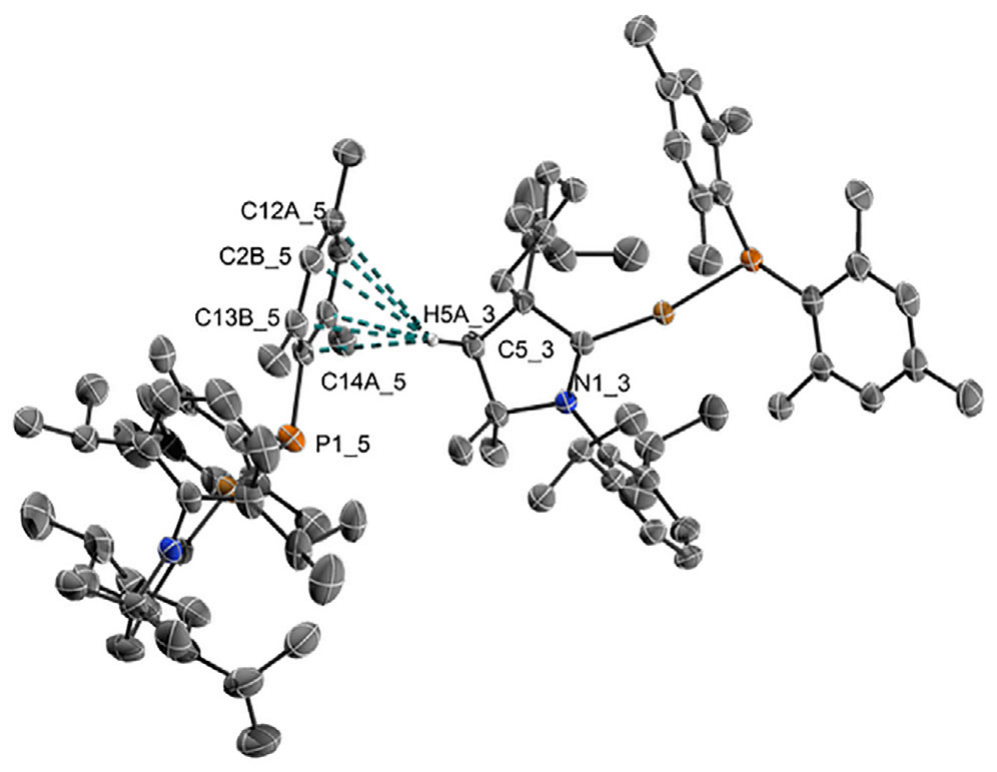
Csp^3–^H^…^π interactions between H5 from the cAAC backbone and the π system of the mesityl substituent; bond distances (in Å) d(C13B, H5) = 2.9059(22), d(C2B,H5) = 3.1030(24).

The luminescence dissymmetry of **2d** in the solid state is weak with *g*_lum_≈1.2·10^−3^, comparable to other structurally related CPL‐TADF complexes [50, 51]. As **2d** exhibits intermolecular non‐polar C(sp^3^)–H**^…^** π *London* dispersion interactions in the crystals similar to previously reported [Cu(BINAP)(Cz)] (Figure 7), it is not surprising that the chiroptical properties in polar and rigid PMMA capable of hydrogen bonding are very similar. However, more flexible and nonpolar PS not only leads to a bathochromic emission shift, but we also observe a drastic increase of the dissymmetry by a factor of 10 (*g*_lum_≈10–^2^) as well as a sign change. We attribute this finding to stabilization of a different excited state geometry and interactions between **2d** and the matrix. For **2d** in PS we calculated a CPL‐brightness [80] of *B*_CPL_ = 1.31 M^−1^ cm^−1^ according to Equation (3):

(3) $$B_{\mathrm{CPL}\;}=\varepsilon_{\lambda }\;\cdot \phi \;\frac{\left|g_{lum}\right|}{2}$$

and using *k*_CPL_ = (*k*_r_·*g*_lum_)/2 as a new way to quantify the efficiency of CPL emission [51], we obtained *k*_CPL_  =  41.5 s^−1^ and 15.6 s^−1^ for PS and the solid state, respectively. The comparatively high CPL‐brightness is probably due to a reduced *k*_nr_ compared to similar complexes (e.g. [Cu(BINAP)(Cz)]: *B*_CPL_ = 2 M^−1^ cm^−1^ [51], [Cu(^Ment^cAAC)(PZN)]: *B*_CPL_ = 1.9 M^−1^ cm^−1^ [50], Pt^II^: *B*_CPL_ = 0.01‐4.7 or Ir^III^: *B*_CPL_ = 0.63‐1.1 [80]) but considerably less than in compounds with *Laporte*‐forbidden transitions, like some Cr^III^ complexes (*B*_CPL_ = 6.3·10^−5^ – 174 M^−1^ cm^−1^) [20, 80, 81], allowing high *Φ* with reduced *k*_r_.

### 2.5 Photocatalysis

The significant absorbance in the visible blue spectral region renders complexes **2** as suitable candidates for photocatalytic hydrophosphination reactions, which have been reported to occur under thermal conditions with complexes of several metals [82, 83, 84]. A few studies employing other earth‐abundant metal complexes based on Ti^IV^ [85], Zr^IV^ [86, 87], Fe (II [88, 89]) or Ni (II [90]) as catalysts suggest that UV or visible light irradiation in the presence of a HPR_2_ or HP(O)R_2_ species and unsaturated organic substrates can initiate radical reactions, which finally provide access to the desired hydrophosphination products. *Waterman* et al. have shown that Cu^I^ complexes are generally also suitable for this purpose [68, 75, 91] but do not require radical reactions. Instead, high energy UV excitation with 360 nm light, for example of [Cu(NHC)(PPh_2_)], populates an LMCT state weakening the Cu–P bond, which allows for 2,1‐insertion of the alkene or nucleophilic attack [75]. Considering the low energy absorption of **2**, the previous reports motivated us to investigate the potential of the cAAC based Cu^I^ complexes.

**SCHEME 2:**
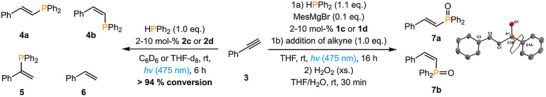
Photocatalyzed hydrophosphination reaction of phenylacetylene with HPPh_2_ employing **2c** or **2d** as precatalysts (left), or using **1c** and **1d** for in situ formation of catalytically active phosphide complexes with subsequent isolation of **7a/b** after oxidative work‐up (right), and molecular structure of **7a** determined by SC‐XRD (right, ellipsoids given for 50% probability; hydrogen atoms are omitted and one phenyl ring is depicted as wireframe for clarity). Selected bond lengths (Å) and angles (°): P1‐O1: 1.491(3), C1A‐P1: 1.807(5), C1B‐P1 1.810(4), C1‐P1: 1.774(4), C1‐C2: 1.342(6), C2‐C3: 1.472(6), C1‐P1‐O1: 114.9(2), P1‐C1‐C2: 119.9(4), C1‐C2‐C3: 125.2(4). For the Full ORTEP/PLATON plot and unit cell, see Figure S32.

Irradiation of a C_6_D_6_ or THF‐d_8_ solution containing phenyl acetylene and diphenylphosphine with 480 nm light for 6 h in the presence of 2–10 mol % of **2c** gave nearly quantitative conversion, forming both (*E*) and (*Z*) hydrophosphination products **4a** and **4b** as detected by ^1^H and ^31^P{^1^H} NMR spectroscopy together with traces of styrene, while the *gem*‐product is not observed (Scheme 2, left, and Table 2). Further addition of substrates continues the photocatalytic hydrophosphination reaction (see Supporting Information). Under the same reaction conditions in the dark, only minor product formation < 5% is observed even after 12 h.

**TABLE 2:** Yields of the photocatalyzed hydrophosphination of phenylacetylene (**3**) with diphenylphosphine (HPPh_2_) using [Cu(^R^cAAC)(PMes_2_)] (**2c**/**d**) as photocatalyst, or [Cu(^R^cAAC)Cl] (**1c/d**) as precatalysts according to Scheme 2. Species present were identified by ^1^H and ^31^P NMR spectroscopy and compared to literature values or values obtained from a commercial sample of styrene (**6**).

Cat.	Loading	Solvent	Conversion (HPPh_2_) / %^a^ ^)^	NMR‐Yield / %^a^ ^)^			
				4a (*E*)^b^ ^)^	4b (*Z*)	5 (*gem*)	6
**2c**	10 mol‐%	THF‐d_8_	98	**80**	10	0	5
	10 mol‐%	C_6_D_6_	98	**95**	2	0	3
	5 mol‐%	THF‐d_8_	94	**61**	31	0	4
	2 mol‐%	THF‐d_8_	99	28	**66**	0	3
	2 mol‐%	C_6_D_6_	98	28	**63**	0	3
**2d**	2 mol‐%	THF‐d_8_	97	11	**82**	1	2
				**7a** (*E*)	**7b** (*Z*)		
**1c**	10 mol‐%	THF		**70**	21		
	5 mol‐%	THF		**63**	25		
	2 mol‐%	THF		43	43		
**1d**	10 mol‐%	THF		21	**62**		

^a^ Determined by ^1^H NMR spectroscopy using 1,3,5‐trimethoxybenzene as internal standard.

^b^ ^1^H NMR resonance is strongly overlapping, yield thus determined by ^31^P NMR spectroscopy referenced to **4b**.

Several observations suggest that the mechanism of this photocatalytic transformation is significantly different than observed for prominent photoredox catalysis or bond homolysis mechanisms. At high catalyst loading of 10 mol % **2c**, the formation of **4a** is generally more favored than **4b**, but the yield of the *E*‐isomer is significantly higher in nonpolar C_6_D_6_ with 95:2 than observed for THF‐d_8_ (80:10) (Table 2). While with 5 mol % of **2c** the *E/Z*‐ratio is reduced to 66:34 in THF, further lowering of the catalyst loading to 2 mol % surprisingly favors the *Z*‐isomer independent of the solvent. The catalyst concentration dependent product distribution indicates that formation of the *E*‐isomer **4a** requires aggregation of the copper(I) species, while **4b** appears to be the result of a mono‐copper(I) complex acting as photocatalyst. In line with this interpretation is the fact that **2d** bearing a sterically more demanding carbene with a higher *V*_bur_ (Figure 1) gives a higher yield of **4b** at 2 mol % than **2c**. This interpretation is supported by the finding that **2d** bearing the bulky ^Menth^cAAC with a higher *V*_bur_ of 55% than ^Cy^cAAC with 45% (Figure 1) further shifts the *E/Z*‐ratio towards the latter with 11:82 in comparison to **2c** even at low catalyst loading of 2 mol %. We note that the photocatalytic hydrophosphination does not occur with sterically more demanding HPMes_2_ as a substrate under the same conditions, which rules out an outer‐sphere product forming step in the catalytic cycle or radical reactions (Figure S20).

Deeper insight into the mechanism is provided by ^31^P NMR spectroscopic measurements of **2c** in the presence of free HCCPh in the dark, suggesting fast equilibrium between the copper(I) phosphide and the related Cu–acetylide complex of 0.75:1.00 (Figure S24). However, structurally related and [Cu(^Ad^cAAC)(CCPh)] has been reported to not absorb light at the irradiation wavelength and such a species is thus not relevant for the photoinitiation, but could be a resting state [77]. Furthermore, TD‐DFT calculations of [Cu(cAAC)(η^1^‐CCPh)(PHMes_2_)] reveal only absorption in the UV, but [Cu(cAAC)(η^2^‐HCCPh)(PPh_2_)] exhibits S_0_ → S_1_/S_2_ excitation of (ML)LCT (p_P_+d_Cu_ → π*(HC≡CPh)) character for S_1_ and LLCT (p_P_+π_Mes_ → π*_CN_(cAAC)) character for S_2_ at 474 and 461 nm, respectively (Tables S24, S25, and S31). As fast exchange of ^−^PMes_2_ for ^−^PPh_2_ will occur under the reaction conditions, we therefore infer either [Cu(cAAC)(PPh_2_)] as the derivative of original **2c/d** to be the decisive photocatalytically active species via Cu–P bond weakening due to LLCT excitation and concomitant distortion, or a trigonal Cu^I^ complex with both phosphide and side‐on π‐bond acetylene to be involved as the critical light absorbing and product forming species. As we have described above, the copper(I) carbene diphenylphosphide complex could not be isolated due to its high reactivity and decomposition upon purification, which is the reason why we investigated the PMes_2_ analogues. However, the equilibrium reaction with free phenylacetylene provides a resting state for stabilization to continue the photocatalytic reaction cycle upon addition of further substrate to the reaction mixture (see above).

**FIGURE 8:**
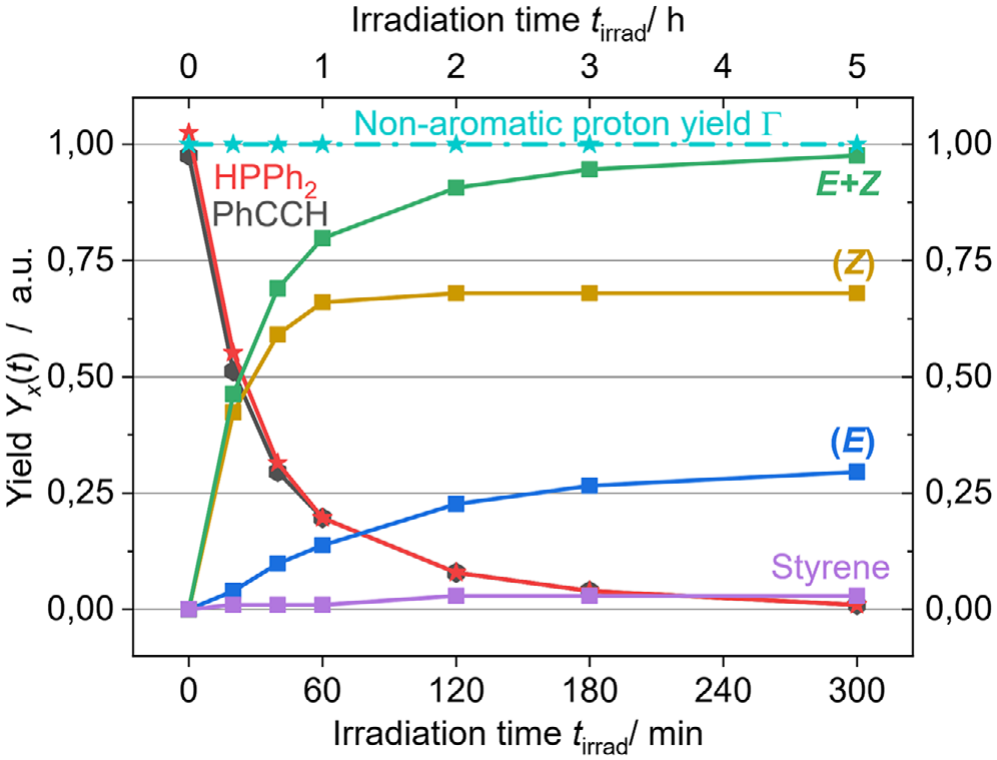
Time vs. product yield plot for the reaction of diphenylacetylene (**3**) with diphenylphosphine catalyzed by 2 mol‐% of **2c** under irradiation with 475 nm light. For additional details see Figure S8.

Investigation of the time‐dependence of the product yield using 2 mol % **2c** reveals very fast formation of the *Z*‐isomer **4b**, which is nearly finished after 60 min, while *E*‐isomer **4a** requires an induction period within the first 30–45 min (Figure 8). Apparently, the assumed aggregation of the photocatalytically active metal complexes is time‐dependent and after 1 h continues the formation of **4a** as the dominant process. We note that styrene (**6**) is observed as a by‐product. Interestingly, its formation is increased after 60 min when the *Z*‐isomer **4b** is barely produced anymore, indicating that **6** is the result of the aggregation process, which is complete after 120 min. These findings further support our hypothesis of inner‐sphere mechanisms of the hydrophosphination, of which the product distribution is under kinetic control of an equilibrium between a photocatalytically active mono‐copper(I) species and its catalytically active aggregates.

Furthermore, **1c/d** can be used as precatalysts by in situ formation of catalytically active phosphide complexes with HPPh_2_ in the presence of a mild mesityl *Grignard* reagent and subsequent addition of phenylacetylene. After 16 h and oxidative work‐up using H_2_O_2_ / NaOH, the desired vinylphosphine oxide could be isolated in 91% yield based on phenylacetylene (Scheme 2, right). For the (*E*)‐vinylphosphine oxide **7a**, the molecular structure was analyzed by SC‐XRD further confirming the double bond configuration [79]. Again, the steric requirements of the carbene ligands and the photocatalyst concentration appear to control the degree of copper(I) complex aggregation and thus the *E/Z*‐product distribution. Employing **2d** in high concentration of 10 mol % inverts the *E/Z*‐ratio, highlighting the potential reaction control for future application. Further studies of other substrate classes are beyond the scope of this work and are currently being studied in our ongoing projects.

## 3 Conclusion

The new copper(I) complexes demonstrate that stable monomeric diarylphosphide coordination compounds can selectively be obtained in pure form, thus expanding the chemical space of donor ligands for new luminescent materials. However, the unexpected chemoluminescence of **2c** in the presence of molecular oxygen shows that they are sensitive to O_2_. We anticipated that introduction of the diarylphosphide ligand in cAAC‐copper(I) complexes should lead to smaller energy gaps between the ground state and the excited states based on the higher polarizability of phosphorous in comparison to well‐known diarylamide complexes, and indeed pronounced bathochromic shifts of the emission (Δλ≈130 nm) have been observed. Although an unexpected large *ΔE*_ST_ reduces the efficiency of TADF, the generally promising photophysical results suggest that further optimization by chemical modification of either the phosphide or the carbene moiety can lead to efficient deep red to NIR emitters. While there are reported cases of CPL active compounds with the *C*_1_ symmetrical carbene [^Menth^Caac] [92], most of its carbene‐metal‐amide (CMA) derivatives do not show this technologically relevant property [50]. The fact that structurally related **2d** does exhibit for small molecules high emission dissymmetry up to *g*_lum_  =  10^−2^ demonstrates that not only the chiral ligand, but the entirety of the ligand system has direct influence on the chiroptical properties, although also environmental effects are relevant as shown by our matrix dependent luminescence studies. Finally, the phosphide complexes **2c** and **2d** proved to be efficient visible‐light photocatalysts for the hydrophosphination of terminal alkynes. In contrast to previous copper based hydrophosphination photocatalysts, the cAAC ligand allows lower energy excitation in the blue spectral region. The observed direct influence of the carbene sterics on the diastereoselectivity of the reaction suggests the prevalence of an inner‐sphere product forming step, of which formation of the E‐isomer requires metal complex aggregation and the Z‐isomer appears to be formed by a mono‐copper(I) species. Further investigation of this reaction regarding functional group tolerance, selectivity and mechanism may render this reaction a synthetically useful tool.

## Conflicts of Interest

The authors declare no conflicts of interest.

## Supporting information




**Supporting File 1**: The authors have cited additional references within the Supporting Information [1–22, 44, 51].

**Supporting File 2**: anie71068‐sup‐0002‐SuppMat2.pdf.

**Supporting File 3**: anie71068‐sup‐0003‐VideoS1.mp4.

**Supporting File 4**: anie71068‐sup‐0004‐Data.zip.

## Data Availability

The data that support the findings of this study are available in the supplementary material of this article.

## References

[1] O. S. Wenger , “Photoactive Complexes With Earth‐Abundant Metals,” Journal of the American Chemical Society 140 (2018): 13522–13533, 10.1021/jacs.8b08822. 30351136

[2] B. M. Hockin , C. Li , N. Robertson , and E. Zysman‐Colman , “Photoredox Catalysts Based on Earth‐Abundant Metal Complexes,” Catalysis Science & Technology 9 (2019): 889–915, 10.1039/C8CY02336K.

[3] C. Wegeberg and O. S. Wenger , “Luminescent First‐Row Transition Metal Complexes,” Journal of the American Chemical Society Au 1 (2021): 1860–1876. 34841405 10.1021/jacsau.1c00353 PMC8611671

[4] G. Giobbio , R. D. Costa , and S. Gaillard , “Earth‐Abundant Transition Metal Complexes in Light‐Emitting Electrochemical Cells: Successes, Challenges and Perspectives,” Dalton Transactions 54 (2025): 3573–3580, 10.1039/D4DT03210A. 39835838

[5] N. Kumar , T. Sharma , N. Thakur , R. Jain , and N. Sinha , “Abundant Transition Metal Based Photocatalysts for Red Light‐Driven Photocatalysis,” Chemistry—A European Journal 31 (2025): e202500365, 10.1002/chem.202500365. 40135511

[6] R. V. Eldik , P. C. Ford , V. W.‐W. Yam , et al., Advances in Inorganic Chemistry (Elsevier, 2024).

[7] V. W.‐W. Yam and W.‐K. Kwok , Advances in Inorganic Chemistry, ed. Rudi van Eldik , Peter C. Ford (Elsevier, 2024), S. 1–3.

[8] V. Ferraro , C. Bizzarri , and S. Bräse , “Thermally Activated Delayed Fluorescence (TADF) Materials Based on Earth‐Abundant Transition Metal Complexes: Synthesis, Design and Applications,” Advancement of Science 11 (2024): e2404866, 10.1002/advs.202404866. PMC11426009 38984475

[9] T. J. Penfold , E. Gindensperger , C. Daniel , and C. M. Marian , “Spin‐Vibronic Mechanism for Intersystem Crossing,” Chemical Reviews 118 (2018): 6975–7025, 10.1021/acs.chemrev.7b00617. 29558159

[10] M. Kleinschmidt , C. van Wüllen , and C. M. Marian , “Intersystem‐crossing and Phosphorescence Rates in Fac‐Ir *III* (ppy)3: A Theoretical Study Involving Multi‐Reference Configuration Interaction Wavefunctions,” Journal of Chemical Physics 142 (2015): 94301, 10.1063/1.4913513. 25747075

[11] E. Yu‐Tzu Li , T.‐Y. Jiang , Y. Chi , and P.‐T. Chou , “Semi‐Quantitative Assessment of the Intersystem Crossing Rate: An Extension of the El‐Sayed Rule to the Emissive Transition Metal Complexes,” Physical Chemistry Chemical Physics 16 (2014): 26184–26192, 10.1039/C4CP03540B. 25363371

[12] V. Ramu , L. S. Wijaya , N. Beztsinna , et al., “Cell Viability Imaging in Tumor Spheroids *via* DNA Binding of a Ruthenium( <scp>ii</scp> ) Light‐Switch Complex,” Chemical Communications 60 (2024): 6308–6311, 10.1039/D4CC01425A. 38818705 PMC11181008

[13] S. Bonnet , “Ruthenium‐Based Photoactivated Chemotherapy,” Journal of the American Chemical Society 145 (2023): 23397–23415, 10.1021/jacs.3c01135. 37846939 PMC10623564

[14] L. Bretin , Y. Husiev , V. Ramu , et al., “Red‐Light Activation of a Microtubule Polymerization Inhibitor via Amide Functionalization of the Ruthenium Photocage,” Angewandte Chemie International Edition 63 (2024), e202316425, 10.1002/anie.202316425. 38061013

[15] A. König , R. Naumann , C. Förster , J. Klett , and K. Heinze , “A Near‐Infrared‐II Luminescent and Photoactive Vanadium(II) Complex With a 760 Ns Excited State Lifetime,” Journal of the American Chemical Society 147 (2025): 20833–20842, 10.1021/jacs.5c04471. 40462271 PMC12186473

[16] M. Dorn , J. Kalmbach , P. Boden , et al., “A Vanadium(III) Complex With Blue and NIR‐II Spin‐Flip Luminescence in Solution,” Journal of the American Chemical Society 142 (2020): 7947–7955, 10.1021/jacs.0c02122. 32275150

[17] L. A. Büldt and O. S. Wenger , “Chromium (0), Molybdenum (0), and Tungsten (0) Isocyanide Complexes as Luminophores and Photosensitizers with Long‐Lived Excited States,” Angewandte Chemie International Edition 56, (2017), 5676–5682. 28317225 10.1002/anie.201701210

[18] F. Reichenauer , C. Wang , C. Förster , et al., “Strongly Red‐Emissive Molecular Ruby [Cr(bpmp) <sub>2</sub> ] <Sup>3+</Sup> Surpasses [Ru(bpy) <sub>3</sub> ] <Sup>2+</Sup>,” Journal of the American Chemical Society 143 (2021): 11843–11855, 10.1021/jacs.1c05971. 34296865

[19] N. Sinha , C. Wegeberg , D. Häussinger , A. Prescimone , and O. S. Wenger , “Photoredox‐active Cr(0) Luminophores Featuring Photophysical Properties Competitive With Ru(II) and Os(II) Complexes,” Nature Chemistry 15 (2023): 1730–1736, 10.1038/s41557-023-01297-9. PMC10695827 37580444

[20] J.‐R. Jiménez , M. Poncet , S. Míguez‐Lago , et al., “Bright Long‐Lived Circularly Polarized Luminescence in Chiral Chromium (III) Complexe,” Angewandte Chemie International Edition 60 (2021), 10095–10102; 33704880 10.1002/anie.202101158

[21] C. Wegeberg and O. S. Wenger , “Luminescent Chromium(0) and Manganese (<scp>i</scp>) Complexes,” Dalton Transactions 51 (2022): 1297–1302, 10.1039/D1DT03763C. 34908065 PMC8787763

[22] S. Kronenberger , R. Naumann , C. Förster , N. East , J. Klett , and K. Heinze , “A Manganese(I) Complex with a 190 ns Metal‐to‐Ligand Charge Transfer Lifetime,” Nature Communications 16 (2025): 7850. 10.1038/s41467-025-63225-4 PMC12373760 40846841

[23] N. R. East , R. Naumann , C. Förster , C. Ramanan , G. Diezemann , and K. Heinze , “Oxidative Two‐State Photoreactivity of a Manganese(IV) Complex Using Near‐Infrared Light,” Nature Chemistry 16 (2024): 827–834, 10.1038/s41557-024-01446-8. 38332331

[24] T. Huang , P. Du , X. Cheng , and Y.‐M. Lin , “Manganese Complexes with Consecutive Mn(IV) → Mn(III) Excitation for Versatile Photoredox Catalysis,” Journal of the American Chemical Society 146 (2024): 24515–24525, 10.1021/jacs.4c07084. 39079011

[25] P. Herr , C. Kerzig , C. B. Larsen , D. Häussinger , and O. S. Wenger , “Manganese(i) Complexes with Metal‐to‐ligand Charge Transfer Luminescence and Photoreactivity,” Nature Chemistry 13 (2021): 956–962, 10.1038/s41557-021-00744-9. 34341527

[26] B. C. Paulus , S. L. Adelman , L. L. Jamula , and J. K. McCusker , “Leveraging Excited‐State Coherence for Synthetic Control of Ultrafast Dynamics,” Nature 582 (2020): 214–218, 10.1038/s41586-020-2353-2. 32528090

[27] J. Steube , A. Kruse , O. S. Bokareva , et al., “Janus‐Type Emission from a Cyclometalated Iron(iii) Complex,” Nature Chemistry 15 (2023): 468–474, 10.1038/s41557-023-01137-w. PMC10070185 36849804

[28] J. Wellauer , F. Ziereisen , N. Sinha , et al., “Iron (III) Carbene Complexes with Tunable Excited State Energies for Photoredox and Upconversion,” Journal of the American Chemical Society 146 (2024): 11299–11318, 10.1021/jacs.4c00605. 38598280 PMC11046485

[29] R. J. Ortiz , R. Mondal , J. K. McCusker , and D. E. Herbert , “Leveraging Intramolecular π‐Stacking to Access an Exceptionally Long‐Lived <Sup>3</Sup> MC Excited State in an Fe(II) Carbene Complex,” Journal of the American Chemical Society 147 (2025): 1694–1708, 10.1021/jacs.4c12650. 39762138

[30] P. Chábera , Y. Liu , O. Prakash , et al., “A Low‐Spin Fe(iii) Complex With 100‐ps Ligand‐to‐Metal Charge Transfer Photoluminescence,” Nature 543 (2017): 695–699, 10.1038/nature21430. 28358064

[31] A. K. Pal , C. Li , G. S. Hanan , and E. Zysman‐Colman , “Blue‐Emissive Cobalt (Iii) Complexes and Their Use in the Photocatalytic Trifluoromethylation of Polycyclic Aromatic Hydrocarbons,” Angewandte Chemie International Edition 2018, 57, 8027–8031; 29726073 10.1002/anie.201802532

[32] L. A. Büldt , C. B. Larsen , and O. S. Wenger , “Luminescent Ni0 Diisocyanide Chelates as Analogues of CuI Diimine Complexes,” Chemistry—A European Journal 23 (2017): 8577–8580. 28295795 10.1002/chem.201700103

[33] S. Malzkuhn and O. S. Wenger , “Luminescent Ni(0) Complexes,” Coordination Chemistry Reviews 359 (2018): 52–56, 10.1016/j.ccr.2018.01.003.

[34] Y.‐S. Wong , M.‐C. Tang , M. Ng , and V. W.‐W. Yam , “Toward the Design of Phosphorescent Emitters of Cyclometalated Earth‐Abundant Nickel(II) and Their Supramolecular Study,” Journal of the American Chemical Society 142 (2020): 7638–7646, 10.1021/jacs.0c02172. 32275398

[35] D. Moreth , M. V. Cappellari , A. Müller , et al., “Luminescent N^C^N Pincer Ni(II), Pd(II), and Pt(II) Complexes With a Pendant Coumarin Group: The Role of Auxiliary Ligands and Environments,” Inorganic Chemistry 64 (2025): 4223–4235, 10.1021/acs.inorgchem.4c03773. 40008821

[36] J. A. Kübler , B. Pfund , and O. S. Wenger , “Zinc(II) Complexes With Triplet Charge‐Transfer Excited States Enabling Energy‐Transfer Catalysis, Photoinduced Electron Transfer, and Upconversion,” JACS Au 2022, 2, 2367–2380. 36311829 10.1021/jacsau.2c00442 PMC9597861

[37] Y. Ma , J. Shen , and J. Zhao , et al., “Multicolor Zinc (II)‐Coordinated Hydrazone‐Based Bistable Photoswitches for Rewritable Transparent Luminescent Labels,” Angewandte Chemie International Edition 61 (2022), e202202655; 35460581 10.1002/anie.202202655

[38] O. Mrózek , M. Gernert , A. Belyaev , et al., “Ultra‐Long Lived Luminescent Triplet Excited States in Cyclic (Alkyl)(amino)Carbene Complexes of Zn(II) Halides,” Chemistry—A European Journal 28 (2022): e202201114. 35583397 10.1002/chem.202201114 PMC9544448

[39] S. Koop , O. Mrózek , L. Janiak , et al., “Synthesis, Structural Characterization, and Phosphorescence Properties of Trigonal Zn(II) Carbene Complexes,” Inorganic Chemistry 63 (2024): 891–901, 10.1021/acs.inorgchem.3c03915. 38118184

[40] M. Mitra , O. Mrózek , M. Putscher , et al., “Structural Control of Highly Efficient Thermally Activated Delayed Fluorescence in Carbene Zinc (II) Dithiolates,” Angewandte Chemie International Edition 63 (2024), e202316300. 38063260 10.1002/anie.202316300

[41] D. A. Shariaty , P. I. Djurovich , and M. E. Thompson , “Structural and Electronic Tuning of Luminescent ZnII Complexes Based on an o‐Terphenyl Ligand Motif,” Journal of the American Chemical Society 147 (2025): 29065–29078. 40736152 10.1021/jacs.5c07162

[42] D. A. Shariaty , J. Schaab , E. McClure , et al., “Donor/Acceptor Ligands Based on an *o* ‐Terphenyl Motif to Achieve Thermally Activated Delayed Fluorescence in Zn(II) Complexes,” Inorganic Chemistry 64 (2025): 1228–1240, 10.1021/acs.inorgchem.4c04383. 39808071

[43] M. Gernert , U. Müller , M. Haehnel , J. Pflaum , and A. Steffen , “A Cyclic Alkyl(amino)Carbene as Two‐Atom π‐Chromophore Leading to the First Phosphorescent Linear Cu <Sup>I</Sup> Complexes,” Chemistry—A European Journal 23 (2017): 2206–2216, 10.1002/chem.201605412. 27911043

[44] R. Hamze , J. L. Peltier , D. Sylvinson , et al., “Eliminating Nonradiative Decay in Cu(I) Emitters: ≫99% Quantum Efficiency and Microsecond Lifetime,” Science 363 (2019): 601–606, 10.1126/science.aav2865. 30733411

[45] M. Gernert , L. Balles‐Wolf , F. Kerner , et al., “Cyclic (Amino)(aryl)Carbenes Enter the Field of Chromophore Ligands: Expanded π System Leads to Unusually Deep Red Emitting Cu <Sup>I</Sup> Compounds,” Journal of the American Chemical Society 142 (2020): 8897–8909, 10.1021/jacs.0c02234. 32302135

[46] R. Tang , S. Xu , T.‐L. Lam , et al., “Highly Robust CuI‐TADF Emitters for Vacuum‐Deposited OLEDs with Luminance up to 222 200 cd m− 2 and Device Lifetimes (LT90) up to 1300 hours at an Initial Luminance of 1000 cd m− 2,” Angewandte Chemie International Edition 61 (2022): e202203982; 35647660 10.1002/anie.202203982

[47] A. Ruduss , B. Turovska , S. Belyakov , K. A. Stucere , A. Vembris , and K. Traskovskis , “Carbene–Metal Complexes as Molecular Scaffolds for Construction of Through‐Space Thermally Activated Delayed Fluorescence Emitters,” Inorganic Chemistry 61 (2022): 2174–2185, 10.1021/acs.inorgchem.1c03371. 35038860

[48] C. N. Muniz , C. A. Archer , J. S. Applebaum , et al., “Two‐Coordinate Coinage Metal Complexes as Solar Photosensitizers,” Journal of the American Chemical Society 145 (2023): 13846–13857, 10.1021/jacs.3c02825. 37319428

[49] A. Steffen , A. Muthig , T. Ferschke , J. Pflaum , and E. Björn , “Emitter Material for OLEDs,” Patent EP4581037A1 (July 29 2025).

[50] A. M. T. Muthig , J. Wieland , C. Lenczyk , et al., “Towards Fast Circularly Polarized Luminescence in 2‐Coordinate Chiral Mechanochromic Copper(I) Carbene Complexes,” Chemistry—A European Journal 29 (2023): e202300946, 10.1002/chem.202300946. 37272620

[51] A. M. T. Muthig , O. Mrózek , T. Ferschke , et al., “Mechano‐Stimulus and Environment‐Dependent Circularly Polarized TADF in Chiral Copper(I) Complexes and Their Application in OLEDs,” Journal of the American Chemical Society 145 (2023): 4438–4449, 10.1021/jacs.2c09458. 36795037

[52] T.‐Y. Li , S.‐J. Zheng , P. I. Djurovich , and M. E. Thompson , “Two‐Coordinate Thermally Activated Delayed Fluorescence Coinage Metal Complexes: Molecular Design, Photophysical Characters, and Device Application,” Chemical Reviews 124 (2024): 4332–4392, 10.1021/acs.chemrev.3c00761. 38546341

[53] J. Beaudelot , S. Oger , S. Peruško , et al., “Photoactive Copper Complexes: Properties and Applications,” Chemical Reviews 122 (2022): 16365–16609, 10.1021/acs.chemrev.2c00033. 36350324

[54] Q. Zhang , N. Li , X. Wan , et al., “Harnessing of Cooperative Cu⋅⋅⋅H Interactions for Luminescent Low‐Coordinate Copper (I) Complexes Towards Stable OLEDs,” Angewandte Chemie International Edition 64 (2025): e202419290; 39641632 10.1002/anie.202419290

[55] A. S. Romanov , S. T. E. Jones , Q. Gu , et al., “Carbene Metal Amide Photoemitters: Tailoring Conformationally Flexible Amides for Full Color Range Emissions Including White‐emitting OLED,” Chemical Science 11 (2020): 435–446, 10.1039/C9SC04589A. 32190264 PMC7067249

[56] A. Steffen and B. Hupp , Comprehensive Coordination Chemistry III (Elsevier, 2021): 466–502.

[57] H. Yersin and U. Monkowius , “Thermally Activated Delayed Fluorescence and Beyond. Photophysics and Material Design Strategies,” Advanced Photonics Research 6 (2025): 2400111, 10.1002/adpr.202400111.

[58] J. M. Dos Santos , D. Hall , B. Basumatary , et al., “The Golden Age of Thermally Activated Delayed Fluorescence Materials: Design and Exploitation,” Chemical Reviews 124 (2024): 13736–14110, 10.1021/acs.chemrev.3c00755. 39666979 PMC12132800

[59] I. Sen , O. Mrózek , M. Mitra , et al., “Carbazolophane Enhances the Efficiency of Thermally Activated Delayed Fluorescence in Carbene Coinage Metal Amides,” ChemRxiv (2023), 10.26434/chemrxiv‐2023‐snwpg.

[60] C. Riley , W. Jones , N. L. E. Phuoc , M. Linnolahti , and A. S. Romanov , “Cyclic(amino)(barrelene)Carbene Metal Amide Complexes: Synthesis and Thermally Activated Delayed Fluorescence,” Organic Electronics 137 (2025): 107156, 10.1016/j.orgel.2024.107156.

[61] M. S. Kellogg , A. R. Mencke , C. N. Muniz , et al., “Intra‐ and Intermolecular Charge‐Transfer Dynamics of Carbene–Metal–Amide Photosensitizers,” Journal of Physical Chemistry C 128 (2024): 6621–6635, 10.1021/acs.jpcc.4c01994. PMC11056983 38690534

[62] A. M. T. Muthig , M. Krumrein , J. Wieland , et al., “Trigonal Copper(I) Complexes With Cyclic (Alkyl)(amino)Carbene Ligands for Single‐Photon Near‐IR Triplet Emission,” Inorganic Chemistry 61 (2022): 14833–14844, 10.1021/acs.inorgchem.2c02376. 36069727

[63] S. Shi , M. C. Jung , C. Coburn , et al., “Highly Efficient Photo‐ and Electroluminescence From Two‐Coordinate Cu(I) Complexes Featuring Nonconventional N‐Heterocyclic Carbenes,” Journal of the American Chemical Society 141 (2019): 3576–3588, 10.1021/jacs.8b12397. 30768250

[64] A. Jouaiti , L. Ballerini , H.‐L. Shen , et al., “Inside Back Cover: Binuclear Copper (I) Complexes for Near‐Infrared Light‐Emitting Electrochemical Cells,” Angewandte Chemie International Edition 62 (2023): e202305569; 37345993 10.1002/anie.202305569

[65] T. A. Annan , R. Kumar , and D. G. Tuck , “The Direct Electrochemical Synthesis of Metal–diphenylphosphido Complexes, and the Crystal Structure of Cu <Sub>4</Sub> (μ‐PPh <sub>2</sub>) <Sub>4</Sub> (Ph <sub>2</sub> PCH <sub>2</sub> PPh <sub>2</sub> ) <Sub>2</Sub>,” Journal of the Chemical Society, Chemical Communications 0 (1988): 446–448, 10.1039/C39880000446.

[66] T. A. Annan , R. Kumar , and D. G. Tuck , “Direct Electrochemical Synthesis and Crystallographic Characterization of Metal Diphenylphosphido and Diphenylthiophosphinato Compounds, and some Derivatives,” Journal of the Chemical Society, Dalton Transactions (1991): 11–18, 10.1039/dt9910000011.

[67] A. Eichhöfer , D. Fenske , and W. Holstein , “New Phosphido‐Bridging Copper Clusters,” Angewandte Chemie International Edition 32 (1993): 242–245;

[68] S. G. Dannenberg and R. Waterman , “Cyclo‐Tetrakis(μ‐diphenylphosphido)‐1,5‐bis(tri‐tert‐butylphosphine)‐Tetracopper,” Molbank 2022 (2022): M1334, 10.3390/M1334.

[69] C. Meyer , H. Grützmacher , and H. Pritzkow , “Copper Pnictogenides as Selective Reagents: A New Access to Functionalized Phosphanes and Arsanes,” Angewandte Chemie International Edition 36 (1997): 2471–2473.

[70] I. Abdellah , E. Bernoud , J.‐F. Lohier , et al., “Neutral Copper–phosphido‐borane Complexes: Synthesis, Characterization, and Use as Precatalysts in Csp–P Bond Formation,” Chemical Communications 48 (2012): 4088, 10.1039/c2cc30723e. 22430669

[71] G. C. Fortman , A. M. Z. Slawin , and S. P. Nolan , “A Versatile Cuprous Synthon: [Cu(IPr)(OH)] (IPr = 1,3 bis(diisopropylphenyl)Imidazol‐2‐ylidene),” Organometallics 29 (2010): 3966–3972, 10.1021/om100733n.

[72] B. Khalili Najafabadi and J. F. Corrigan , “Enhanced Thermal Stability of Cu–silylphosphido Complexes via NHC Ligation,” Dalton Transactions 44 (2015): 14235–14241, 10.1039/C5DT02040A. 26182889

[73] J. Yuan , L. Zhu , J. Zhang , J. Li , and C. Cui , “Sequential Addition of Phosphine to Alkynes for the Selective Synthesis of 1,2‐Diphosphinoethanes Under Catalysis. Well‐Defined NHC‐Copper Phosphides vs in Situ CuCl <Sub>2</Sub> /NHC Catalyst,” Organometallics 36 (2017): 455–459, 10.1021/acs.organomet.6b00854.

[74] T. M. Horsley Downie , J. W. Hall , T. P. Collier Finn , et al., “The First Ring‐Expanded NHC–copper( <scp>i</scp> ) Phosphides as Catalysts in the Highly Selective Hydrophosphination of Isocyanates,” Chemical Communications 56 (2020): 13359–13362, 10.1039/D0CC05694D. 33030162

[75] S. G. Dannenberg , D. M. Seth , E. J. Finfer , and R. Waterman , “Divergent Mechanistic Pathways for Copper(I) Hydrophosphination Catalysis: Understanding That Allows for Diastereoselective Hydrophosphination of a Tri‐Substituted Styrene,” ACS Catal 13 (2023): 550–562, 10.1021/acscatal.2c05221.

[76] R. Wei , S. Ju , and L. L. Liu , “Free Metallophosphines: Extremely Electron‐Rich Phosphorus Superbases That Are Electronically and Sterically Tunable,” Angewandte Chemie International Edition 61 (2022): e202205618; 35491966 10.1002/anie.202205618

[77] A. S. Romanov , C. R. Becker , C. E. James , et al., “Copper and Gold Cyclic (Alkyl)(amino)Carbene Complexes With Sub‐Microsecond Photoemissions: Structure and Substituent Effects on Redox and Luminescent Properties,” Chemistry—A European Journal 23 (2017): 4625–4637, 10.1002/chem.201605891. 28164390

[78] K. C. Mondal , S. Roy , B. Maity , D. Koley , and H. W. Roesky , “Estimation of σ‐Donation and π‐Backdonation of Cyclic Alkyl(amino) Carbene‐Containing Compounds,” Inorganic Chemistry 55 (2016): 163–169, 10.1021/acs.inorgchem.5b02055. 26675319

[79] , “Deposition numbers 2481679 (2a), 2481670 (2b), 2481647 (2c), 2481689 (2d) and 2481691 (5a) contain the supplementary crystallographic data for this paper. These data are provided free of charge by the joint Cambridge Crystallographic Data Centre and Fachinformationszentrum Karlsruhe Access Structures service.”.

[80] L. Arrico , L. Di Bari , and F. Zinna , “Quantifying the Overall Efficiency of Circularly Polarized Emitters,” Chemistry—A European Journal 27 (2021): 2920–2934, 10.1002/chem.202002791. 32725832

[81] J.‐R. Jiménez , B. Doistau , C. M. Cruz , et al., “Chiral Molecular Ruby [Cr(dqp)2]3+ With Long‐Lived Circularly Polarized Luminescence,” Journal of the American Chemical Society 141 (2019): 13244–13252. 31353904 10.1021/jacs.9b06524

[82] C. A. Bange and R. Waterman , “Challenges in Catalytic Hydrophosphination,” Chemistry—A European Journal 22 (2016): 12598–12605, 10.1002/chem.201602749. 27405918

[83] B. T. Novas and R. Waterman , “Metal‐Catalyzed Hydrophosphination,” Chemcatchem 14 (2022): e202200988, 10.1002/cctc.202200988.

[84] M. B. Reuter , D. M. Seth , D. R. Javier‐Jiménez , E. J. Finfer , E. A. Beretta , and R. Waterman , “Recent Advances in Catalytic Pnictogen Bond Forming Reactions *via* Dehydrocoupling and Hydrofunctionalization,” Chemical Communications 59 (2023): 1258–1273, 10.1039/D2CC06143K. 36648191

[85] D. M. Seth and R. Waterman , “Photo‐Initiated Radical Hydrophosphination at Titanium Compounds Capable of Ti–P Insertion,” Organometallics 42 (2023): 1213–1219, 10.1021/acs.organomet.3c00049.

[86] C. A. Bange , M. A. Conger , B. T. Novas , E. R. Young , M. D. Liptak , and R. Waterman , “Light‐Driven, Zirconium‐Catalyzed Hydrophosphination With Primary Phosphines,” ACS Catal 8 (2018): 6230–6238, 10.1021/acscatal.8b01002.

[87] C. A. Bange and R. Waterman , “Zirconium‐Catalyzed Intermolecular Double Hydrophosphination of Alkynes With a Primary Phosphine,” ACS Catal 6 (2016): 6413–6416, 10.1021/acscatal.6b01850.

[88] B. J. Ackley , J. K. Pagano , and R. Waterman , “Visible‐Light and Thermal Driven Double Hydrophosphination of Terminal Alkynes Using a Commercially Available Iron Compound,” Chemical Communications 54 (2018): 2774–2776, 10.1039/C8CC00847G. 29484325

[89] J. K. Pagano , C. A. Bange , S. E. Farmiloe , and R. Waterman , “Visible Light Photocatalysis Using a Commercially Available Iron Compound,” Organometallics 36 (2017): 3891–3895, 10.1021/acs.organomet.7b00499.

[90] H. Hou , B. Zhou , J. Wang , et al., “Stereo‐ and Regioselective Cis ‐Hydrophosphorylation of 1,3‐Enynes Enabled by the Visible‐Light Irradiation of NiCl <Sub>2</Sub> (PPh <sub>3</sub> ) <Sub>2</Sub>,” Organic Letters 23 (2021): 2981–2987, 10.1021/acs.orglett.1c00626. 33784463 10.1021/acs.orglett.1c00626

[91] S. G. Dannenberg and R. Waterman , “A Bench‐Stable Copper Photocatalyst for the Rapid Hydrophosphination of Activated and Unactivated Alkenes,” Chemical Communications 56 (2020): 14219–14222, 10.1039/D0CC06570F. 33112298

[92] M. Deng , N. F. M. Mukthar , N. D. Schley , and G. Ung , “Yellow Circularly Polarized Luminescence from C1‐Symmetrical Copper (I) Complexes,” Angewandte Chemie International Edition 59 (2020): 1228–1231; 31778290 10.1002/anie.201913672

