# Supplementary material for: Organophosphides: A New Class of Luminophore Ligands for Copper(I) Carbene Based TADF Emitters and Photocatalysts
Source: Angew Chem Int Ed Engl. 2026 Feb 25;65(13):e18530. doi: 10.1002/anie.202518530 (PMC13007583; doi:10.1002/anie.202518530)
Supplement: Supplementary file 1 — Supporting File 1: The authors have cited additional references within the Supporting Information [1–22, 44, 51]. [file ANIE-65-e18530-s002.pdf]

Table of Contents

1. Synthetic part 2

1.1. General considerations 2

1.2. Synthesis of precursors compounds 3

1.2.1. Synthesis of copper(I) carbene chlorido complexes 3

1.2.2 Synthesis of the secondary phosphine Mes_2PH 6

1.3. Synthesis of phosphide complexes 9

1.4. Photocatalysis 15

2. X-Ray Structure Determination 40

3. 1- and 2-D NMR Spectra 48

NMR spectra of $H(O)PMes_2$ and $HPMes_2$ 49

NMR spectra of 2a [Cu(^{Me}cAAC)($PMes_2$)] 55

NMR spectra of 2b [Cu(^{Et}cAAC)($PMes_2$)] 64

NMR spectra of 2c [Cu(^{Cy}cAAC)($PMes_2$)] 73

NMR spectra of 2d [Cu(^{Ment}cAAC)($PMes_2$)] 86

NMR spectra Photocatalysis products 95

4. MIR-absorption-spectra 103

MIR-Spectra of complexes 2a – 2d 103

MIR-Spectrum of Photocatalysis product 5a 107

5. HR-Mass-Spectra 108

HRMS of Complexes 2a – 2d 108

HR-mass spectra of the oxidation product 111

HR-mass spectra of Photocatalysis products (PC-1, PC-2) 114

6. References 121

1. Synthetic part

1.1. General considerations

Unless otherwise specified, all reactions were conducted in oven dried glassware under an argon atmosphere using standard Schlenk techniques or inside an argon filled glove box. Solids were weighted in air or inside the glove box, transferred to the reaction vessel and the vessel atmosphere was flushed with argon. Liquids were added via dry syringes and oven dried needles and cannulas. THF, CH_2Cl_2, toluene and Et_2O and *n*-Pentane were used after drying with an *Inert Systems PureSolv MD7* solvent purification system, degassed before use and stored over thoroughly activated molecular sieves (4 Å). Reactions were monitored by TLC analysis using the same eluent mixture as for the analytical TLC. Flash column chromatography was performed on silica gel (40-70 µm mesh) purchased from *Acros Organics* under positive air pressure. Analytical thin layer chromatography (TLC) was performed on silica gel 60 F_{254} aluminum plates (*Macherey-Nagel*). TLC plates were visualized at 254 nm and/or $KMnO_4$- or *p*-anisaldehyde staining solution followed by heating with a hot air fan. Commercially available chemicals were purchased from *ThermoFisher, Acros Organics*, *Sigma-Aldrich*, *Alfa Aesar*, *ABCR* and *TCI Europe*, *BLD Pharm* and used as received unless otherwise stated. Commercial and synthesized *Grignard* reagents were titrated using iodine and LiCl according to a method published by *Knochel*.[1] Organolithium reagents were titrated using diphenylacetic acid.[2] For the titration of iPrMgCl·LiCl, no LiCl was added. Molecular sieves (3 – 4 Å) were activated by heating under vacuum ($p < 1$ mbar) at 100 °C for 2 h and further 6 h at 300 °C. ^{1}H-, ^{13}C- and ^{31}P NMR spectra were recorded at room temperature on a *Bruker Avance III HD NanoBay* (400 MHz), *Bruker Avance NEO*, *Bruker Avance NEO II* (500 MHz, DFG-funded project No. 452669591) or a *Bruker Avance III HD* (600 MHz, DFG-funded project No. 452669688) NMR spectrometer. Chemical shifts (δ scale, in ppm) were referenced as follows: $CDCl_3$ (referenced to solvent signal: 7.26 ppm for ^{1}H NMR and 77.16 ppm for ^{13}C NMR); C_6D_6 (referenced to solvent signal: 7.16 ppm for ^{1}H NMR and 128.06 ppm for ^{13}C NMR); THF-d_8 (referenced to solvent signal: 3.58 ppm for ^{1}H NMR and 67.21 ppm for ^{13}C NMR). ^{31}P NMR spectra were not calibrated by an internal reference and shifts are reported relative to 85 % H_3PO_4 in water. If present, residual dimesitylphosphine was used as internal reference with $\delta = -93.6$ ppm.[3] ^{15}N NMR data were recorded via a (^{1}H,^{15}N)-HMBC experiment referenced to liquid ammonia. ^{15}N NMR chemical shifts were converted to nitromethane reference standard by subtraction of 380.2 ppm. Deuterated solvents for NMR spectroscopy were purchased from *Eurisotop* and *Deutero*. For NMR spectroscopy of oxygen and moisture sensitive substances, the solvents were dried chemically (THF, Benzene: $LiAlH_4$; $CDCl_3$: CaH_2), distilled under inert atmosphere and stored over thoroughly activated molecular sieves (4 Å). The multiplicities of the signals are reported as s (singlet), brs (broad singlet), d (doublet), t (triplet), q (quartet), p (pentet), sept (septet), septd (septet of doublets), m (multiplet or overlap of non-equivalent resonances), dd (doublet of doublets), dt (doublet of triplets), tt (triplet of triplets), ddd (doublet of doublets of doublets), ddq (doublet of doublets of quadruplets) and dddd (doublet of doublets of doublets of doublets). The subscript "app" refers to an apparent multiplet. Coupling constants (*J*) are given in Hz. Structural assignments were made with additional information from COSY, HSQC, HMBC, NOESY experiments. (^{1}H,^{13}C)-HSQC and HMBC spectra were recorded using the NOAH supersequence.[4] Infrared spectra (ATR) of air-stable compounds were recorded using a Spectrum 3 FTIR spectrometer in a range from 4000 to 500 cm^{-1} and IR spectra of air sensitive compounds were recorded inside an argon-filled glove box using a Bruker Alpha II IR spectrometer in a range from 4000 to 500 cm^{-1}, both used in reflection mode with a diamond ATR (attenuated total reflectance) unit. Wavenumbers are reported in cm^{-1}. High resolution mass spectra (HRMS) were performed on a mass spectrometer equipped with an *LTQ Orbitrap or LTQ Orbitrap XL* (*Thermo Scientific*) mass analyzer and an electrospray ionization (ESI) or atmospheric pressure chemical ionization (APCI) ion source and were performed at the Center for Mass Spectrometry at TU Dortmund University. Photocatalytic reactions were performed using an *EvoluChem LED 475PF* (475 nm, 230 V, 206 mW cm^{-2}) inside an *EvoluChem PhotoRedOx Box* photoreactor setup.

1.2. Synthesis of precursors compounds

1.2.1. Synthesis of copper(I) carbene chlorido complexes

General procedure GP-1: Complex formation with CuCl

The respective iminium tetrafluoroborate (1.0 equiv) was suspended in toluene ($c = 1.0$ mol L^{-1}) and a solution of $NaN(SiMe_3)_2$ (0.95 equiv) in toluene ($c = 2.0$ mol L^{-1}) was added at rt. After the mixture was stirred overnight, the volatiles were removed *in vacuo*. The residue was suspended in *n*-pentane, filtered through Celite® under inert atmosphere to remove insoluble salts, and the solvent was removed *in vacuo*. The residue containing the free carbene ligand was dissolved in THF ($c = 1.0$ mol L^{-1}) and CuCl (1.1 equiv) was added. The mixture was stirred for 48 h at rt and then filtered through Celite® and basic alumina under inert atmosphere. The solution was concentrated to half its original volume and the crude product was precipitated by addition of *n*-pentane. After filtration, the product was washed with *n*-pentane and dried *in vacuo*.

Chlorido(1-(2,6-diisopropylphenyl)-4,4-dimethyl-2,2-dimethyl-3,4 dihydro-2*H*-pyrrol-1-ylidene)copper(I) [Cu(^{Me}cAAC)Cl]

According to general procedure **GP-1**, [^{Me}cAACH][BF_4] (0.75 g, 2.00 mmol, 1.0 equiv) was reacted with $NaN(SiMe_3)_2$ (367 mg, 2.00 mmol, 1.0 equiv) in toluene (10 mL). The crude carbene was reacted with CuCl (218 mg, 2.2 mmol, 1.1 equiv). Complex **1a** was obtained as a white powder (461 mg, 1.20 mmol, 60 %).

Chemical Formula: $C_{20}H_{31}ClCuN$
Exact Mass: 383,1441

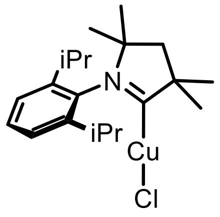

1a

(^1H,^{15}N)-HMBC: (500, 51 MHz, THF-d$_8$): δ / ppm (1.40, –152.2)

The ^1H- and {^1H}^{13}C NMR spectroscopic data match with those reported in the literature.[5]

Chlorido(1-(2,6-diisopropylphenyl)-4,4-diethyl-2,2-dimethyl-3,4-dihydro-2*H*-pyrrol-1-ylidene)copper(I) [Cu(^{Et}cAAC)Cl]

According to general procedure **GP-1**, [^{Et}cAACH][BF_4] (1.00 g, 2.50 mmol, 1.0 equiv) was reacted with $NaN(SiMe_3)_2$ (498 mg, 2.50 mmol, 1.0 equiv) in toluene (12 mL). The crude carbene was reacted with CuCl (297 mg, 3.0 mmol, 1.2 equiv). Complex **1b** was obtained as a white powder (0.73 g, 1.77 mmol, 71 %).

Chemical Formula: $C_{22}H_{35}ClCuN$
Exact Mass: 411,1754

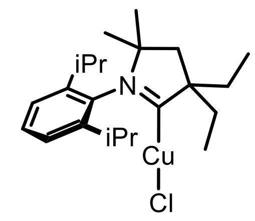

1b

^{1}H NMR: (600.3 MHz, THF-d$_8$): δ / ppm: 7.42 (dd, J = 8.3, 7.2 Hz, 1H, H_{ar}), 7.33 (d, J = 7.8 Hz, 2H, H_{ar}), 2.91 (sept, J = 6.8 Hz, 2H, H_{iPr}), 2.04 (s, 2H, H_{CH2}), 1.91 (dq, $^2J_{AB}$ = 13.7 Hz, J = 7.5 Hz, 2H, H_{Et}), 1.80 (dq, $^2J_{AB}$ = 13.7 Hz, J = 7.5 Hz, 2H, H_{Et}), 1.37 (s, 6H, H_{Me}), 1.32 (d, J = 6.8 Hz, 6H, H_{Me}), 1.29 (d, J = 6.7 Hz, 6H, H_{Me}), 1.11 (t, J = 7.5 Hz, 6H, H_{Me}).

^{13}C{^{1}H} NMR: (151.0 MHz, THF-d$_8$) 145.9 (2C, $C_{q,ar}$), 135.7 ($C_{q,ar}$), 130.3 (C_{ar}), 125.3 (2C, C_{ar}), 81.5 (C_q), 63.0 (C_q), 43.1, 31.6 (2C, C_{Et}), 29.8 (2C, C_{iPr}), 29.2 (2C, C_{Me}), 27.1 (2C, C_{Me}), 22.4 (2C, C_{Me}), 9.6 (2C, C_{Me}).

(^{1}H,^{15}N)-HMBC: (500, 51 MHz, THF-d$_8$): δ / ppm (1.40, –152.2)

Chlorido(2-(2,6-diisopropylphenyl)-3,3-dimethyl-azaspiro[4.5]dec-1-ylidene)] copper(I) [Cu(^{Cy}cAAC)Cl]

According to general procedure **GP-1**, [^{Cy}cAACH][BF_4] (4.00 g, 9.68 mmol, 1.0 equiv) was reacted with $NaN(SiMe_3)_2$ (1.77 g, 9.68 mmol, 1.0 equiv) in toluene (50 mL). The crude carbene was reacted with CuCl (0.96 g, 9.68 mmol, 1.0 equiv). Complex **1c** was obtained as a white powder (2.25 g, 5.30 mmol, 55 %).

Chemical Formula: $C_{23}H_{35}ClCuN$
Exact Mass: 423,1754

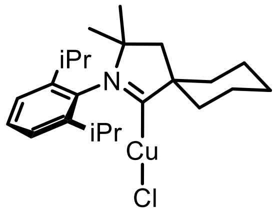

1c

^{1}H NMR: (500 MHz, $CDCl_3$): δ / ppm 7.41 (t, J = 7.7 Hz, 1H, H_{ar}), 7.32 (d, J = 7.7 Hz, 2H, H_{ar}), 2.89 (sept, J = 6.7 Hz, 2H, H_{iPr}), 2.13 (m, 2H, H_{Cy}), 2.13 (s, 2H, H_{CH2}), 1.91 – 1.84 (m, 2H, H_{Cy}), 1.76 – 1.69 (m, 1H, H_{Cy}), 1.60 – 1.54 (m, 2H, H_{Cy}), 1.53 – 1.44 (m, 3H, H_{Cy}), 1.36 (s, 6H, H_{Me}), 1.31 (d, J = 6.8 Hz, 6H, H_{Me}), 1.28 (d, J = 6.7 Hz, 6H, H_{Me}).

$^{13}C\{^{1}H\}$ NMR: (126 MHz, THF-d$_8$): δ / ppm 250.0 (C$_q$), 145.9 (2C, C$_{q,ar}$), 135.4 (C$_{q,ar}$), 130.3 (C$_{ar}$), 125.3 (2C, C$_{ar}$), 81.2 (C$_q$), 59.8 (C$_q$), 46.2, 36.7 (2C), 29.7 (2C), 29.5 (2C), 27.1 (2C), 26.1, 22.7 (2C), 22.5 (2C).

(^{1}H,^{15}N)-HMBC: (500, 51 MHz, THF-d$_8$): δ / ppm (1.40, –152.2)

Chlorido((6*S*,9*R*)-2-(2,6-diisopropylphenyl)-6-isopropyl-3,3,9-trimethylazaspiro[4.5]dec-1-en-1-ylidene)copper(I)

Chemical Formula: $C_{27}H_{43}ClCuN$
Exact Mass: 479,2380

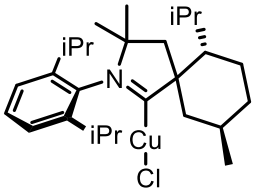

2b

According to general procedure **GP-1**, [M^{enth}CAACH][BF$_4$] (0.7 g, 1.49 mmol, 1.0 equiv) was reacted with NaN(SiMe$_3$)$_2$ (273 mg, 1.49 mmol, 1.0 equiv) in toluene (15 mL). The crude carbene was reacted with CuCl (162 mg, 1.64 mmol, 1.0 equiv). Complex **1d** was obtained as a white powder (428 mg, 0.90 mmol, 60 %).

^{1}H NMR: (500 MHz, THF-d$_8$): δ / ppm 7.41 (t, J = 7.7 Hz, 1H, H$_{ar}$), 7.33 (m, 1H, H$_{ar}$), 7.31 (m, 1H, H$_{ar}$), 2.94 (m, 1H), 2.88 (m, 1H), 2.84 – 2.68 (m, 2H), 2.33 (d, ^{2}J = 13.6 Hz, 1H), 2.08 (dp$_{app}$, J = 12.8, J = 3.4 Hz, 1H), 2.00 (m, 1H), 1.97 (m, 1H), 1.82 (d, J = 13.6 Hz, 1H), 1.81 (dd, J = 14.0, J = 3.5 Hz, 1H), 1.44 – 1.41 (m, 3H), 1.41 – 1.39 (m, 4H), 1.37 (s, 3H), 1.35 – 1.30 (m, 9H), 1.27 (d, J = 6.6 Hz, 3H), 1.12 (ddd, J = 12.4, 12.2, 3.2 Hz, 1H), 1.06 (d, J = 6.97 Hz, 1H), 1.03 (d, J = 6.9 Hz, 3H), 0.90 (d, J = 6.4 Hz, 3H).

$^{13}C\{^{1}H\}$ NMR: (126 MHz, THF-d$_8$): δ / ppm 146.4 (C$_{q,ar}$), 145.9 (C$_{q,ar}$), 136.2 (C$_{q,ar}$), 130.2 (C$_{ar}$), 125.4 (C$_{ar}$), 125.3 (C$_{ar}$), 78.3 (C$_q$), 65.8 (C$_q$), 53.1, 51.9, 48.8, 36.5, 31.5, 29.9, 29.8, 29.6, 29.3, 28.4, 27.8, 26.9, 25.5, 24.5, 23.0, 22.6, 22.5, 20.3.

(^{1}H,^{15}N)-HMBC: (500, 51 MHz, THF-d$_8$): δ / ppm (1.40, –152.2)

The analytical data match with those reported in the literature.[6]

1.2.2 Synthesis of the secondary phosphine Mes_2PH

Bis(2,4,6-trimethylphenyl)phosphine oxide (10)

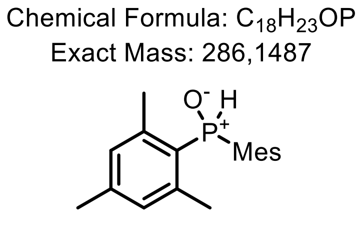

10

Analogous to a known procedure:[7] In a 250 mL two-necked round bottom flask, a solution of mesitylmagnesium bromide[8] (c = 0.32 mol L^{-1}, 42 mL, 13.4 mmol, 1.0 equiv) was cooled to 0 °C and a solution of diethyl phosphonate (0.63 mL, 0.68 g, 0.36 equiv) in THF (15 mL) was added dropwise by means of a dropping funnel. The ice bath was removed and the solution was stirred at rt for 16 h. Aqueous HCl (c = 1.0 mol L^{-1}, 20 mL) was added carefully at 0 °C and the system was stirred vigorously for 1 h. CH_2Cl_2 (30 mL) was added to the mixture, the phases were separated and the aqueous phase was extracted with CH_2Cl_2 (2 × 20 mL). The combined organic phases were washed with brine, dried over $MgSO_4$, and filtered. The solvent was removed under reduced pressure and the crude product was washed with cold *n*-pentane. The product was isolated as an off-white solid containing trace amounts of mesitylene and was used without further purification.

^{1}H NMR: (400 MHz, $CDCl_3$): δ / ppm 8.54 (d, $^1J_{HP}$ = 477 Hz, 1H), 6.86 (d, $^4J_{HP}$ = 3.9 Hz, 4H), 2.39 (s, 12H), 2.28 (s, 6H).

^{13}C{^{1}H} NMR: (101 MHz, $CDCl_3$): δ / ppm 142.1 (d, $^4J_{CP}$ = 2.7 Hz, 2C, $C_{q,ar}$), 141.9 (d, $^2J_{CP}$ = 10.6 Hz, 4C, $C_{q,ar}$), 130.6 (d, $^3J_{CP}$ = 10.7 Hz, 4C, C_{ar}), 126.4 (d, $^1J_{CP}$ = 100 Hz, 2C, $C_{q,ar}$), 21.3 (d, $^5J_{CP}$ = 1.1 Hz, 2C, $C_{p\text{-Me}}$), 20.9 (d, $^3J_{CP}$ = 7.8 Hz, 4C, $C_{o\text{-Me}}$).

^{31}P{^{1}H} NMR: (162 MHz, $CDCl_3$): δ / ppm 10.4.

The analytical data match with those reported in the literature.[7]

Bis(2,4,6-trimethylphenyl)phosphine (11)

Caution: Triisobutylaluminium is highly pyrophoric and will ignite in contact with air!

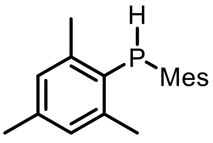

11

In analogy to a known procedure:[7] A solution of the crude phosphine oxide (**10**) in toluene (50 mL) was treated with neat triisobutylaluminium (9.9 mL, 7.76 g, 39.1 mmol, *assuming* 8.0 equiv) and the solution was heated to 75 °C for 24 h. Degassed NaOH (c = 1.0 mol L^{-1}) and degassed saturated *Rochelle* salt solution were added carefully at −78 °C while stirring the mixture vigorously. The mixture was diluted with Et_2O and the organic phase was filtrated over $MgSO_4$ and SiO_2 under inert atmosphere and the solvent was removed *in vacuo*. The solid was dissolved in *n*-pentane and filtered over a plug of basic alumina under inert atmosphere. The solvent was removed *in vacuo* and the product was obtained as a white waxy solid (0.78 g, 2.89 mmol, 59 % over 2 steps).

^{1}H NMR: (500 MHz, $CDCl_3$): δ / ppm 6.82 (d, $^4J_{HP}$ = 2.6 Hz, 4H), 5.25 (d, $^1J_{HP}$ = 232.4 Hz, 1H), 2.26 (d, $^4J_{HP}$ = 1.1 Hz, 12H), 2.25 (s, 6H).

^{13}C{^{1}H} NMR (126 MHz, $CDCl_3$): δ / ppm 142.4 (d, $^2J_{CP}$ = 12.4 Hz, 4C, $C_{q,ar}$), 137.9 (s, 2C, $C_{q,ar}$), 129.4 (d, $^1J_{CP}$ = 15.8 Hz, 2C, $C_{q,ar}$), 129.2 (d, $^3J_{CP}$ = 2.9 Hz, 2C, C_{ar}), 22.9 (d, $^3J_{CP}$ = 10.7 Hz, 4C, $C_{o\text{-Me}}$), 21.1 (s, 2C, $C_{p\text{-Me}}$).

^{31}P NMR: (162 MHz, $CDCl_3$): δ / ppm −93.1 (d, $^1J_{HP}$ = 232.6 Hz, 1P).

The analytical data match with those reported in the literature.[7]

(Phenylmethyl)potassium

Caution: Benzylpotassium is highly pyrophoric and will ignite immediately in contact with air!

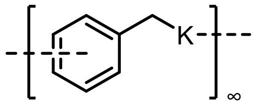

Analogous to a known procedure:[9] *t*-BuOK (2.34 g, 20.9 mmol, 1.0 equiv) was dissolved in toluene (30 mL) and the clear solution was cooled to –10 °C. At this temperature, a solution of *n*-BuLi in Hexanes (c = 2.5 mol L^{-1}, 8.50 mL, 21.3 mmol, 1.0 equiv) was added dropwise and the solution turned bright orange-red and after a few minutes, an orange precipitate formed. After 40 min at –5 °C, the mixture was warmed to room temperature and the orange suspension was filtered through a glass frit under inert atmosphere. The orange solid was copiously washed with toluene followed by *n*-pentane and dried *in vacuo* to yield (phenylmethyl)potassium as a dark orange-red pyrophoric solid which was used without further characterization (2.61 g, 20.0 mmol, 96%).

1.3. Synthesis of phosphide complexes

(Bis(2,4,6-trimethylphenyl)phosphido)(1-(2,6-diisopropylphenyl)-4,4-dimethyl-2,2-dimethyl-3,4-dihydro-2*H*-pyrrol-1-ylidene)copper(I) [Cu(^{Me}cAAC)($PMes_2$)]

Chemical Formula: $C_{38}H_{53}CuNP$
Exact Mass: 617,3212

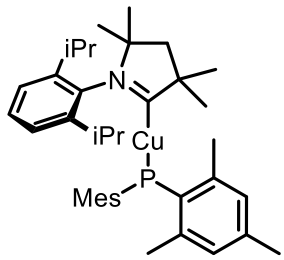

2a

In an argon filled glovebox, $HPMes_2$ (111 mg, 410 µmol, 1.05 equiv) and $KN(SiMe_3)_2$ (77.8 mg, 390 µmol, 1.0 equiv) were dissolved in THF (2.0 mL) and the deep orange solution was stirred for 2 h at rt. This solution was added dropwise to a stirred solution of [Cu(^{Me}cAAC)Cl] (**1a**, 150 mg, 390 µmol, 1.0 equiv) at rt and the mixture was stirred at rt for 48 h. After filtration of the deep red reaction mixture over a plug of basic alumina and Celite®, and removal of all volatiles *in vacuo*, the remaining orange powder was dissolved in a mixture of Et_2O (2.5 mL) and THF (0.5 mL) and deep red-orange single crystals of **2a** suitable for X-ray diffraction experiments were obtained by vapor diffusion of a mixture of *n*-pentane (3.0 mL) and cyclohexane (3.0 mL) at −30 °C. For analytical purity and spectroscopy, the crystals were washed with small amounts of *n*-pentane and dried *in vacuo*. (162 mg, 262 µmol, 67 %).

IR: (ATR): 3070, 3009, 2968, 2949, 2912, 2722, 2285, 2237, 2211, 2185, 2164, 2150, 1998, 1986, 1952, 1721, 1677, 1601, 1588, 1513, 1456, 1422, 1385, 1367, 1343, 1323, 1265, 1232, 1205, 1177, 1135, 1114, 1053, 1028, 1006, 953, 905 cm^{-1}.

^{1}H NMR: (500 MHz, THF-d_8): δ / ppm 7.42 (t, J = 7.8 Hz, 1H, H_{ar}), 7.27 (d, J = 7.7 Hz, 2H, H_{ar}), 6.53 (d, $^4J_{HP}$ = 2.3 Hz, 4H, $H_{ar,Mes}$), 2.84 (sept, J = 6.8 Hz, 2H, H_{iPr}), 2.10 (s, 6H, $H_{Mes,Me}$), 2.02 (s, 2H), 1.97 (s, 12H, $H_{Mes,Me}$), 1.36 (s, 6H, H_{Me}), 1.31 (s, 6H, H_{Me}), 1.26 (d, J = 6.7 Hz, 6H, H_{Me}), 1.10 (d, J = 6.7 Hz, 6H, H_{Me}).

^{13}C{^{1}H} NMR: (125 MHz, THF-d_8): δ / ppm 252.1 (d, $^2J_{CP}$ = 34.9 Hz, $C_{q,carbene}$), 146.3 (2C, $C_{q,ar}$), 143.2 (d, $^1J_{CP}$ = 28.4 Hz, 2C, $C_{q,ar}$), 141.2 (d, $^2J_{CP}$ = 10.4 Hz, 4C, $C_{q,ar}$), 136.1 ($C_{q,ar}$), 132.3 (2C, $C_{q,ar}$), 130.5 (C_{ar}), 128.4 (d, $^3J_{CP}$ = 28.4 Hz, 4C, C_{ar}), 125.8 (2C, C_{ar}), 82.2 (C_q), 55.1 (C_q), 50.6, 30.1 (2C), 29.6 (2C), 28.7 (2C), 27.2 (2C), 25.9 (2C), 23.3 (2C), 21.2 (4C).

^{1}H-^{15}N-HMBC: (500 MHz, THF-d_8) δ / ppm −156.2.

^{31}P{^{1}H} NMR: (162 MHz, THF-d_8) δ / ppm −75.5.

ESI-HRMS *m/z*: $[M + H]^+$ Calcd. for $[C_{38}H_{53}CuNP + H]^+$: 618.3284. Found: 618.3284.

Elemental Analysis: Calc.: 73.81 % C; 8.64 % H; 2.27 % N. Exp.: 73.85 % C; 8.80 % H, 2.50 % N.

UV/VIS: (Toluene) λ / nm (*ε* / M^{-1} cm^{-1}): 339 (15293), 482 (2122)

(Bis(2,4,6-trimethylphenyl)phosphido)(1-(2,6-diisopropylphenyl)-4,4-diethyl-2,2-dimethyl-3,4-dihydro-2*H*-pyrrol-1-ylidene)copper(I) [Cu(^{Et}cAAC)(PMes2)]

Chemical Formula: $C_{40}H_{57}CuNP$
Exact Mass: 645,3525

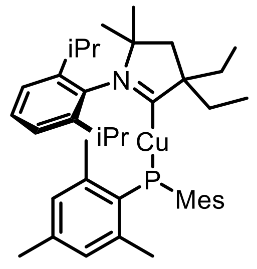

2b

In an argon filled glovebox, $HPMes_2$ (**SI-2,** 196.6 mg, 0.73 mmol, 1.0 equiv) and benzyl potassium (94.7 mg, 0.73 mmol, 1.0 equiv), were placed together in a glass tube with a *J-Young*-valve and cooled to –78 °C. Pre-cooled THF (–78 °C, 3 mL) was added *via* stainless-steel cannula and the mixture was stirred for 1 h at –78 °C and 1 h at room temperature. The deep orange mixture was added to a solution of [Cu(^{Et}cAAC)Cl] (**1b**, 300 mg, 0.73 mmol, 1.0 equiv) in THF (2 mL) and the mixture was stirred for 48 h at room temperature. After filtration over a plug of basic alumina and Celite® and concentration of the solution *in vacuo*, the product was crystallized by vapor diffusion of Et_2O / *n*-pentane affording the compound as deep-yellow plates suitable for single crystal X-ray diffraction experiments. Analytical purity was achieved after washing **2b** with *n*-pentane and drying *in vacuo* (242 mg, 374 µmol, 51 %).

IR: (ATR): 2963, 2869, 1602, 1516, 1460, 1365, 1319, 1261, 1176, 1136, 1051, 1023, 953, 934, 900 cm^{-1}.

^{1}H NMR: (500 MHz, THF-d_8): *δ* / ppm 7.42 (t, J = 7.8 Hz, 1H, H_{ar}), 7.29 (d, J = 7.8 Hz, 2H, H_{ar}), 6.53 (d, $^4J_{HP}$ = 2.3 Hz, 4H, H_{Mes}), 2.88 (sept, J = 6.7 Hz, 2H, H_{iPr}), 2.11 (s, 6H, H_{Mes}), 1.96 (s, 12H, H_{Mes}), 1.69 (dq, $^2J_{AB}$ = 14.1 Hz, J = 7.3 Hz, 2H, H_{Et}), 1.59 (dq, $^2J_{AB}$ = 14.5 Hz, J = 7.4 Hz, 2H, H_{Et}), 1.38 (s, 6H, H_{Me}), 1.26 (d, J = 6.8 Hz, 6H, H_{Me}), 1.15 (d, J = 6.7 Hz, 6H, H_{Me}), 0.94 (t, J = 7.4 Hz, 6H, H_{Me}).

^{13}C{^{1}H} NMR: (126 MHz, THF-d_8): *δ* / ppm 253.9 (d, $^2J_{CP}$ = 37.9 Hz, $C_{q,carbene}$). 146.3 (2C, $C_{q,ar}$), 143.21 (d, $^1J_{CP}$ = 29.1 Hz, 2C, $C_{q,ar}$), 141.19 (d, $^2J_{CP}$ = 10.4 Hz, 2C, $C_{q,ar}$), 136.6 ($C_{q,ar}$), 132.3 (2C, $C_{q,ar}$), 130.5 (C_{ar}), 128.41 (d, $^3J_{CP}$ = 2.4 Hz, 4C, C_{ar}), 125.8 (2C, C_{ar}), 81.9 (C_q), 63.7 (C_q), 43.0, 32.0 (2C), 30.2 (2C), 29.7 (2C), 27.4 (2C), 25.8 (4C), 23.2 (2C), 21.2 (2C), 10.0 (2C).

^{1}H-^{15}N-HMBC: (500 MHz, THF-d_8) *δ* / ppm −153.2.

$^{31}P\{^{1}H\}$ NMR: (203 MHz, THF-d_8) δ / ppm −76.1.

ESI-HRMS *m/z*: $[M + H]^+$ Calcd for $[C_{40}H_{57}CuNP + H]^+$: 646.3597. Found: 646.3607.

Elemental Analysis: Calcd.: 74.3 % C; 8.89 % H; 2.17 % N. Exp.: 74.30 % C; 9.00 % H, 2.15 % N.

UV/VIS: (Toluene) λ / nm (ε / M^{-1} cm^{-1}): 337 (9729), 475 (1382).

(Bis(2,4,6-trimethylphenyl)phosphido)(2-(2,6-diisopropylphenyl)-3,3-dimethylazaspiro[4.5]dec-1-en-1-ylidene)copper(I) [Cu(^{Cy}cAAC)(PMes$_2$)]

Chemical Formula: $C_{41}H_{57}CuNP$
Exact Mass: 657,3525

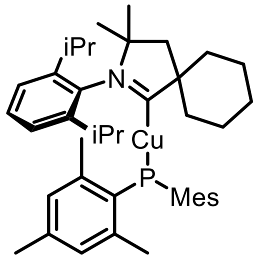

2c

In an argon filled glovebox, [Cu(^{Cy}cAAC)Cl] (**1c**, 300 mg, 0.73 mmol, 1.0 equiv), $HPMes_2$ (196.6 mg, 0.73 mmol, 1.0 equiv) and *t*-BuOK (94.7 mg, 0.73 mmol, 1.0 equiv) were dissolved in THF (7.0 mL) and the deep red solution was stirred for 2 d at rt. After filtration over a plug of basic alumina and Celite®, and removal of the volatiles *in vacuo*, the crude product was dissolved in Et_2O (2 mL) and filtered through a PTFE syringe tip filter (5 µm). Crystallization by slow vapor diffusion of *n*-pentane at −25 °C resulted in the formation of deep red-orange plates of **2c** suitable for X-Ray diffraction experiments and of sufficient purity for spectroscopic investigations (278 mg, 422 µmol, 60 %).

IR: (ATR): 2935, 2857, 1517, 1456, 1386, 1367, 1344, 1323, 1246, 1180, 1148, 1112, 1053, 1028, 1007, 935 cm^{-1}.

^{1}H NMR: (400 MHz, Benzene-d_6): δ / ppm 7.19 (d_{app}, J = 7.8 Hz, 1H, H_{ar}), 7.00 (d, J = 7.8 Hz, 2H, H_{ar}), 6.90 (d, $^{4}J_{HP}$ = 2.4 Hz, 4H, H_{Mes}), 2.65 (sept, J = 6.6 Hz, 2H, H_{iPr}), 2.47 (s, 12H, H_{Mes}), 2.27 (s, 6H, H_{Mes}), 1.87 (td_{app}, J = 12.8, 3.4 Hz, 3H, H_{Cy}), 1.52 (brd_{app}, J = 11.9 Hz, 4H, H_{Cy}), 1.38 (s, 2H), 1.27 (d, J = 6.8 Hz, 6H, H_{Me}), 1.12 – 1.00 (m, 2H, H_{Cy}), 1.08 (d, J = 6.8 Hz, 6H, H_{Me}), 0.82 (s, 6H, H_{Me}).

(600 MHz, THF-d_8): δ / ppm 7.43 (t, J = 7.7 Hz, 1H, H_{ar}), 7.30 (d, J = 7.8 Hz, 2H, H_{ar}), 6.55 (d, J = 2.4 Hz, 4H, H_{Mes}), 2.87 (sept, J = 6.8 Hz, 2H, H_{iPr}), 2.11 (s, 6H, H_{Mes}), 2.04 (s, 2H), 1.98 (s, 12H, H_{Mes}), 1.80 (m, 2H, H_{Cy}), 1.65 – 1.55 (m, 3H, H_{Cy}), 1.40 – 1.35 (m, 2H, H_{Cy}), 1.36 (s, 6H, H_{Me}), 1.27 (d, J = 6.7 Hz, 6H, H_{Me}), 1.18 (d, J = 6.8 Hz, 6H, H_{Me}), 1.13 (m, 1H, H_{Cy}).

$^{13}C\{^1H\}$ NMR: (101 MHz, Benzene-d_6): δ / ppm. δ / ppm 146.2 (2C, C_q), 143.2 (d, $^1J_{CP}$ = 30.1 Hz, 2C, $C_{q,ar}$), 141.2 (d, $^2J_{CP}$ = 10.6 Hz, 4C, $C_{q,ar}$), 136.4 (C_q), 132.3 (2C, C_{ar}), 130.4, 128.5 (d, $^3J_{CP}$ = 2.5 Hz, 4C, C_{ar}), 125.8 (2C, C_{ar}) 81.6 (C_q) , 60.5 (C_q), 46.6, 36.4 (2C), 30.1 (2C), 30.08 (2C), 27.4 (2C), 26.1 (C), 25.8 (4C), 23.3 (2C), 23.0 (2C), 21.2 (2C).

(151 MHz, THF-d_8): 251.9 (d, $^2J_{CP}$ = 37.2 Hz, C_q), 145.9 (2C, $C_{q,ar}$), 142.9 (d, $^1J_{CP}$ = 30.1 Hz, 2C, $C_{q,ar}$), 140.8 (d, $^2J_{CP}$ = 10.4 Hz, 4C, $C_{q,ar}$), 136.1 ($C_{q,ar}$), 131.9 (2C, $C_{q,ar}$), 130.1 (C_{ar}), 128.1 (d, $^3J_{CP}$ = 2.3 Hz, 4C, C_{ar}), 125.5 (2C, $C_{q,ar}$), 81.2 (C_q), 60.2 (C_q), 46.2, 36.0, 29.74, 29.7, 27.03, 27.0, 25.7 (2C), 25.6 (2C), 25.4 (2C), 25.3 (2C), 23.0 (2C), 22.6 (2C), 20.8 (2C).

^{15}N NMR: (600 MHz, Benzene-d_6) δ / ppm −157.8.

(600 MHz, THF-d_8) δ / ppm −158.1.

$^{31}P\{^1H\}$ NMR: (162 MHz, Benzene-d_6) δ / ppm −75.6.

(243 MHz, THF-d_8) δ / ppm −75.6.

ESI-HRMS *m/z*: $[M + H]^+$ Calcd. for $[C_{41}H_{57}CuNP + H]^+$: 658.3597. Found: 658.3625.

Elemental Analysis: Calcd.: 74.79 % C; 8.73 % H; 1.91 % N Exp.: 74.60 % C; 8.55 % H, 2.15 % N.

UV/VIS: (Toluene) λ / nm (ε / M^{-1} cm^{-1}): 337 (9600), 479 (1500).

In the $^{13}C\{^1H\}$ NMR spectrum recorded in THF-d_8, two resonances (26.1 ppm, 25.8 ppm) corresponding to five carbon atoms are partially superimposed with the upfield signal of THF-d_8.

(Bis(2,4,6-trimethylphenyl)phosphido)((6*S*,9*R*)-2-(2,6-diisopropylphenyl)-6-isopropyl-3,3,9-trimethylazaspiro[4.5]dec-1-en-1-ylidene)copper(I) [Cu(Ment cAAC)(PMes2)]

Chemical Formula: $C_{45}H_{65}CuNP$
Exact Mass: 713,4151

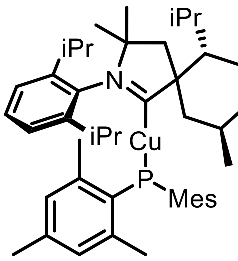

2b

In an argon filled glovebox, $HPMes_2$ (84.4 mg, 312 µmol, 1.0 equiv) and $KN(SiMe_3)_2$ (62.3 mg, 312 µmol, 1.0 equiv) were dissolved in THF (2.0 mL) and the deep orange solution was stirred for 2 h at rt. This solution was added dropwise to a stirred solution of [Cu(^{Ment}cAAC)Cl] (**1d**, 150 mg, 312 µmol, 1.0 equiv) at rt and the mixture was stirred at rt for 48 h. After filtration of the deep red reaction mixture over a plug of basic alumina and Celite®, the volatiles were removed *in vacuo*, the orange powder was dissolved in a minimal volume of *n*-pentane (3.0 mL) and stored at −30 °C to yield crystals suitable for X-Ray diffraction experiments. For analytical purity and spectroscopy, the crystals of **2d** were washed with small amounts of *n*-pentane and dried *in vacuo*. (136 mg, 190 µmol, 61 %).

IR: (ATR): 2954, 2918, 2866, 2848, 1495, 1456, 1385, 1368, 1342, 1320, 1262, 1203, 1174, 1141, 1103, 1052, 1023, 1052, 1023, 954 cm^{-1}.

^{1}H NMR: (600 MHz, THF-d_8): δ / ppm 7.50 (t, J = 7.8 Hz, 1H, H_{ar}), 7.40 (d, J = 7.8 Hz, 2H, H_{ar}), 6.53 (d, $^4J_{HP}$ = 2.4 Hz, 4H, H_{Mes}), 3.03 (dt$_{app}$, J = 13.9, 6.9 Hz, 1H, H_{iPr}), 2.98 (dt$_{app}$, J = 13.5, 7.0 Hz, 1H, H_{iPr}), 2.26 (d, J = 13.6 Hz, 1H, H_{Ment}), 2.11 (s$_{app}$, 7H), 1.95 – 1.89 (m, 1H), 1.88 (m, 13H), 1.81 (dt$_{app}$ (ddd), J_{AB} = 13.5 Hz, J = 2.9 Hz 1H H_{Ment}), 1.74 (d, J_{AB} = 13.4 Hz , 1H H_{Ment}), 1.43 – 1.38 (m, 12H), 1.37 – 1.29 (m, 10H), 1.29 – 1.23 (m, 3H), 1.21 – 1.15 (m, 2H), 1.03 (d, J = 6.9 Hz, 3H, H_{Me}), 0.93 (d, J = 6.9 Hz, 3H, H_{Me}), 0.89 (t$_{app}$ (dd), J = 7.1 Hz, 6H H_{Me}), 0.64 (d, J = 6.4 Hz, 3H, $H_{Pentane}$), 0.55 (qd$_{app}$ (dddd), J_{AB} = 12.3, 3.3 Hz, 1H, H_{Ment}).

^{13}C{^{1}H} NMR: (151 MHz, THF-d_8): δ / ppm 253.4 (d, $^2J_{CP}$ = 38.3 Hz, C_q), 146.1 ($C_{q,ar}$), 145.8 ($C_{q,ar}$), 143.4 (d, $^1J_{CP}$ = 27.5 Hz, 2C, $C_{q,ar}$), 141.1 (d, $^2J_{CP}$ = 10.1 Hz, 4C, $C_{q,ar}$), 137.1 ($C_{q,ar}$), 132.1 (2C, $C_{q,ar}$), 130.3 (C_{ar}), 128.1 (d, $^3J_{CP}$ = 2.5 Hz, 4C, C_{ar}), 126.1 (C_{ar}), 126.0 (C_{ar}), 78.6 (d, $^3J_{CP}$ = 1.8 Hz, C_{spiro}), 53.4, 52.4, 49.2, 36.1, 34.8 (*n*PeH), 31.6, 30.2, 30.0, 29.8, 29.7, 28.5, 28.04, 28.0, 27.2, 27.1, 25.8 (d, $^4J_{CP}$ = 1.8 Hz) 25.1 (2C), 25.0 (2C) , 24.3, 23.4, 23.03, 23.01 (2C, *n*PeH), 22.5, 20.8 (2C) 14.6 (2C, *n*PeH).

^{15}N NMR: (60.8 MHz, THF-d_8) δ –153.9.

$^{31}P\{^{1}H\}$ NMR: (243 MHz, THF-d_8) δ / ppm–75.8.

ESI-HRMS *m/z*: $[M + H]^+$ Calcd for $[C_{45}H_{65}CuNP + H]^+$: 714.4223. Found: 714.4221.

Elemental Analysis: Calcd.: 75.64 % C; 9.17 % H, 1.96 % N Exp.: 76.0 % C; 9.6 % H; 2.2 % N.

UV/VIS: (Toluene) λ / nm (ε / M^{-1} cm^{-1}): 326 (10500), 481 (1900).

NMR spectra were recorded of the single crystals containing one molecule of *n*-pentane (*n*PeH) per molecule of complex. By fine grinding of the complex and thorough drying *in vacuo*, removal of *n*-pentane is possible, as elemental analysis indicates no presence of pentane.

1.4. Photocatalysis

General procedure for NMR scale photocatalysis reactions:

Photocatalytic reactions were performed using an *EvoluChem LED 475PF* (475 nm, 230 V, 206 mW cm^{-2}) inside an *EvoluChem PhotoRedOx Box* photoreactor setup inside a closed black painted box. [Cu(^{R}cAAC)(PMes$_2$)] (0.02 – 0.10 equiv), the respective phosphine (HPPh$_2$ or HPMes$_2$, 1.0 equiv) and the respective alkyne (1.0 equiv) were dissolved in dry THF-d$_8$ or C$_6$D$_6$ and NMR spectra were recorded immediately after. Finally, the solution was irradiated with a 475 nm LED for the indicated period and NMR spectra were recorded again. Yields were calculated using the integral ratio between the product resonances and the starting material resonances calibrated against aromatic proton resonance of 1,3,5-trimethoxybenzene (0.33 equiv, ^{1}H NMR (500 MHz, THF-d$_8$): δ / ppm = 6.04 (s, 3H), 3.70 (s, 9H).

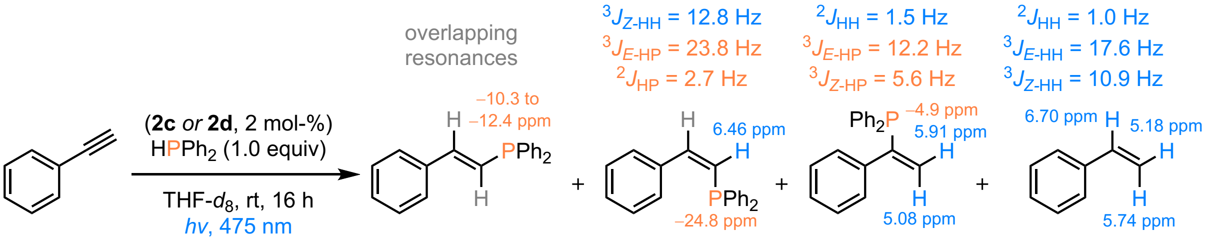

3 4a 4b 5 6

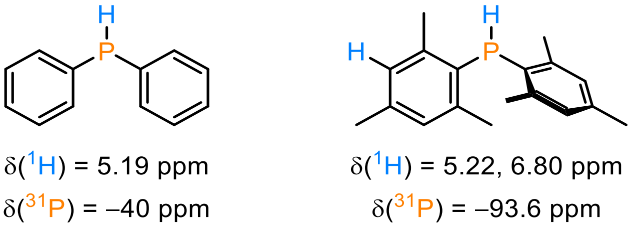

Scheme S 1: NMR scale hydrophosphination of phenylacetylene (**3**) with diphenylphosphine (HPPh$_2$) using compounds [Cu(^{Cy}cAAC)(PMes$_2$)] (**2c**) or [Cu(^{Ment}cAAC)(PMes$_2$)] (**2d**) and important ^{1}H- and ^{31}P NMR resonance values used for assignment of signals: **4b** in CDCl$_3$[10] and **5** in CDCl$_3$.[11] Styrene (**6**) was measured in THF-d$_8$ and ^{1}H NMR literature data for the vinylic protons of **4a** is inconsistent due to overlap of the signal with the aromatic protons of the compound.[11,12]

(E,Z)-P,P-Diphenyl(*ω*-styryl)phosphine (4a + 4b) with 10 mol-% 2c

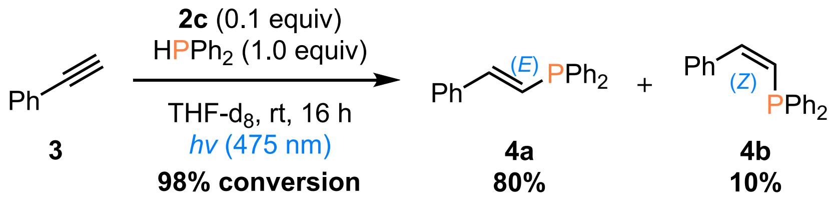

Scheme S 2: NMR scale photocatalyzed hydrophosphination of phenylacetylene (**3**) with diphenylphosphine ($HPPh_2$) using 10 mol-% [Cu(^{Cy}cAAC)($PMes_2$)] (**2c**) in THF-d_8. . Conversion based on consumption of diphenylphosphine

According to the general procedure, **2c** (10 mol-%), 1,3,5-trimethoxybenzene (TMB, 0.33 equiv), $HPPh_2$ (1.0 equiv) and PhCCH (1.0 equiv) were combined in anhydrous THF-d_8.

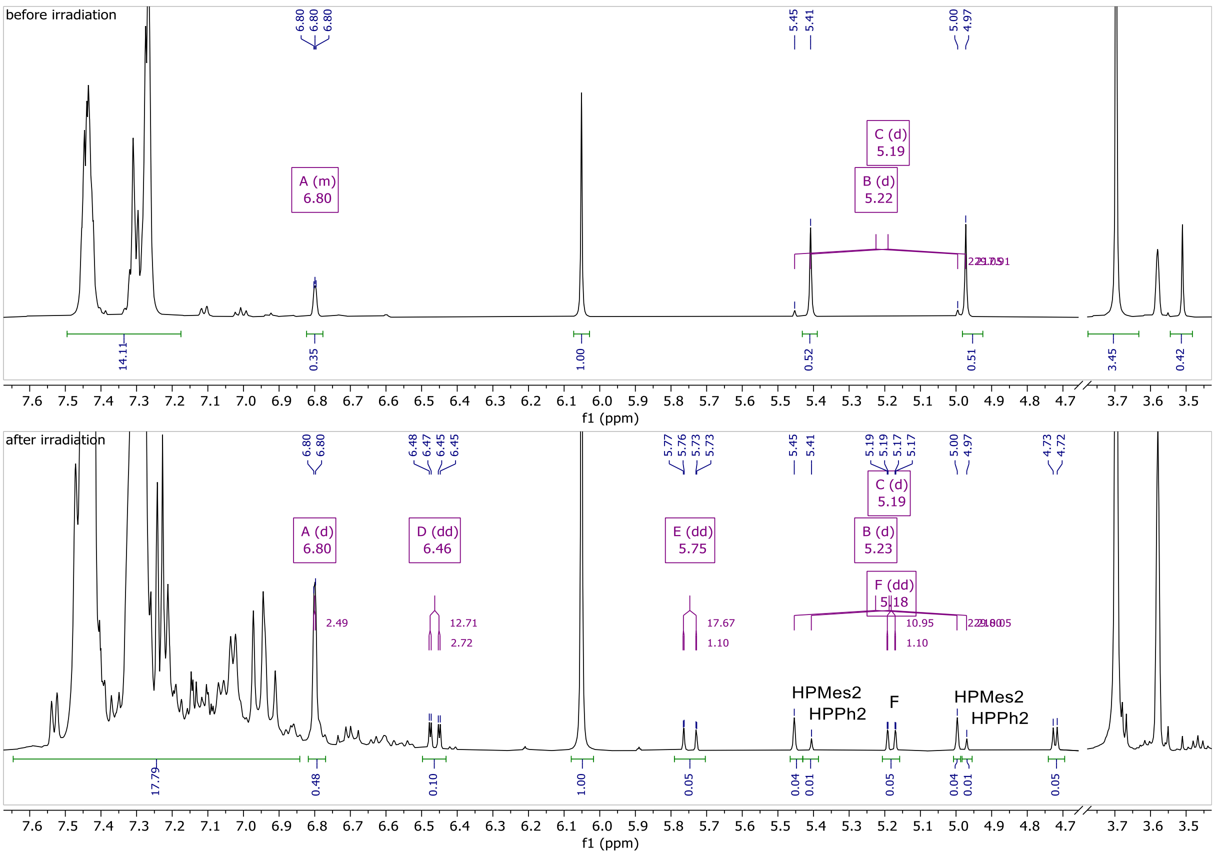

Figure S 1: ^{1}H NMR spectra of $HPPh_2$, TMB, PhCCH and **2c** (10 mol-%) in THF-d_8 (500 MHz) before and after 16 h irradiation: Signal assignment **A** + **B**: *H*-$PMes_2$ (5.23 ppm, d, *J* = 229 Hz, product of ligand exchange, 10 %), **C**: *H*-PPh_2 (5.19 ppm, d, *J* = 218 Hz), **D**: (*Z*)-**4** (**4b**, 10%), **E** – **F**: $H_{Styrene}$ (**6**, 5%). ^{1}H-resonances of (*E*)-**4** (**4a**) are overlapping with catalyst signals and other impurities. Yield determined by ^{31}P NMR spectroscopy (see below).

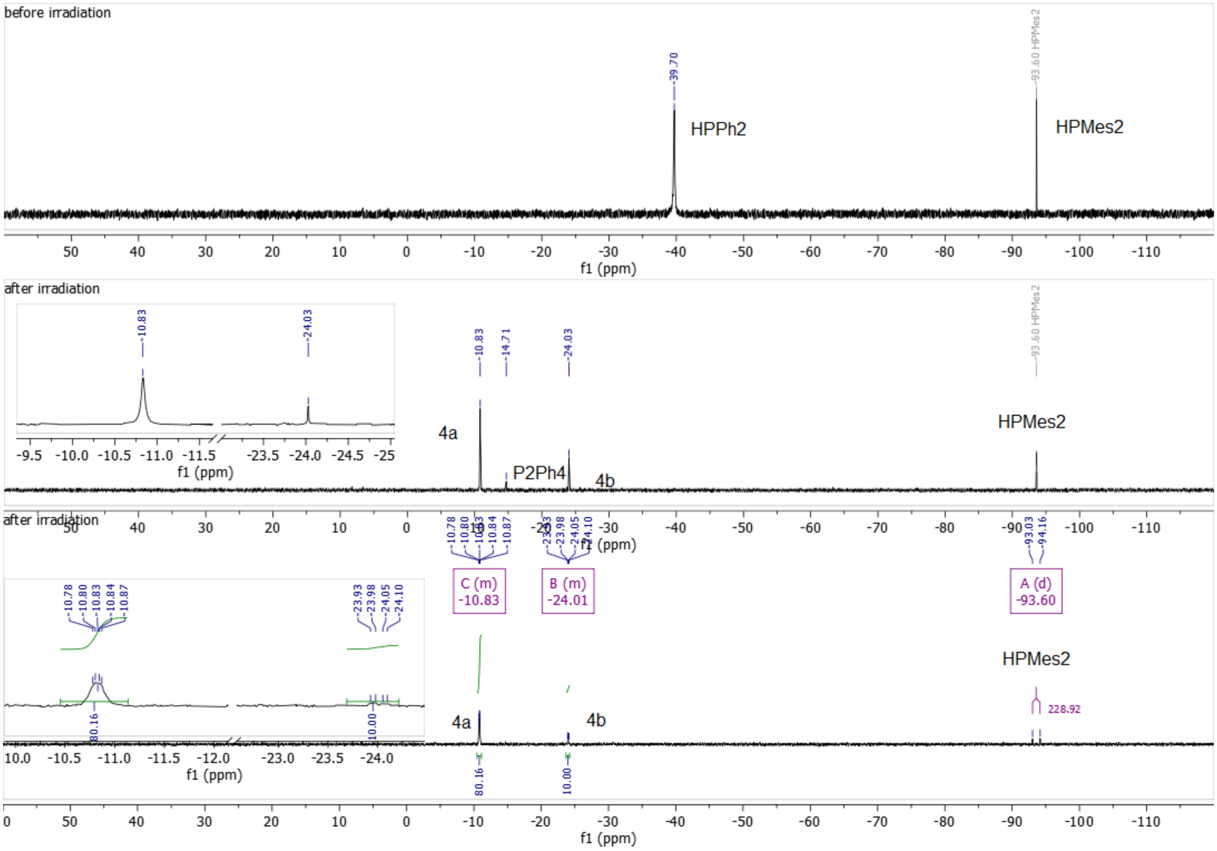

Figure S 2: $^{31}P\{^{1}H\}$ NMR spectra (top and mid) and ^{31}P NMR spectrum (bottom) of $HPPh_2$, TMB, PhCCH and **2c** (10 mol-%) in THF-d_8 (500 MHz) before and after 16 h irradiation and ^{31}P NMR spectrum after irradiation: **A**: $HPMes_2$, **B**: (*Z*)-**4** (**4b**, 10%) and **C**: (*E*)-**4** (**4a**, 80%). The resonance at –14.7 ppm is assigned to trace amounts of Ph_4P_2.[13]

Integration of the ^{31}P NMR resonances indicates a *E*/*Z*-ratio of 8:1 which, with an ^{1}H NMR yield of 10 % for **4b**, indicates the formation of **4b** in 80 % yield.

***(E,Z)-P,P*-Diphenyl(*ω*-styryl)phosphine (4a + 4b) with 5 mol-% 2c**

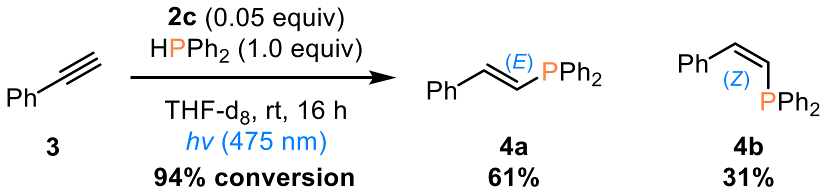

Scheme S 3: NMR scale photocatalyzed hydrophosphination of phenylacetylene (**3**) with diphenylphosphine ($HPPh_2$) using 5 mol-% [Cu(^{Cy}cAAC)($PMes_2$)] (**2c**) in THF-d_8. Conversion based on consumption of diphenylphosphine.

According to the general procedure, **2c** (**5 mol-%**), 1,3,5-trimethoxybenzene (0.33 equiv), $HPPh_2$ (1.0 equiv) and PhCCH (1.0 equiv) were combined in anhydrous THF-d_8.

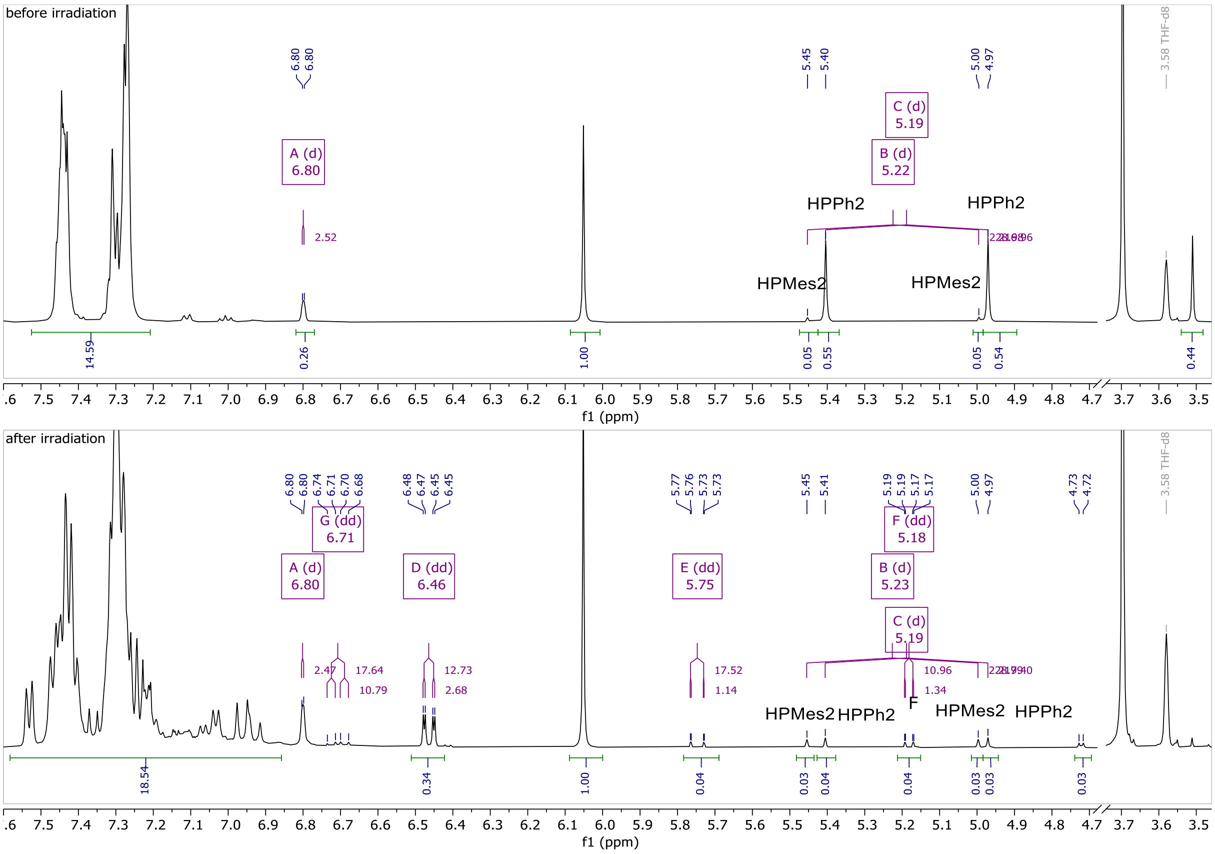

Figure S 3: ^{1}H NMR spectra of $HPPh_2$, PhCCH and **2c** (5 mol-%) in THF-d_8 before and after 16 h irradiation: Signal assignment **A** + **B**: *H*-$PMes_2$ (5.23 ppm, d, J = 229 Hz, product of ligand exchange, 10 %), **C**: *H*-PPh_2 (5.19 ppm, d, J = 218 Hz), **D**: (*Z*)-**4** (**4b**, 31%), **E** – **G**: $H_{Styrene}$ (**6**, 4%). ^{1}H-resonances of (*E*)-**4** (**4a**) are overlapping with catalyst signals and other impurities. Yield determined by ^{31}P NMR spectroscopy (see below).

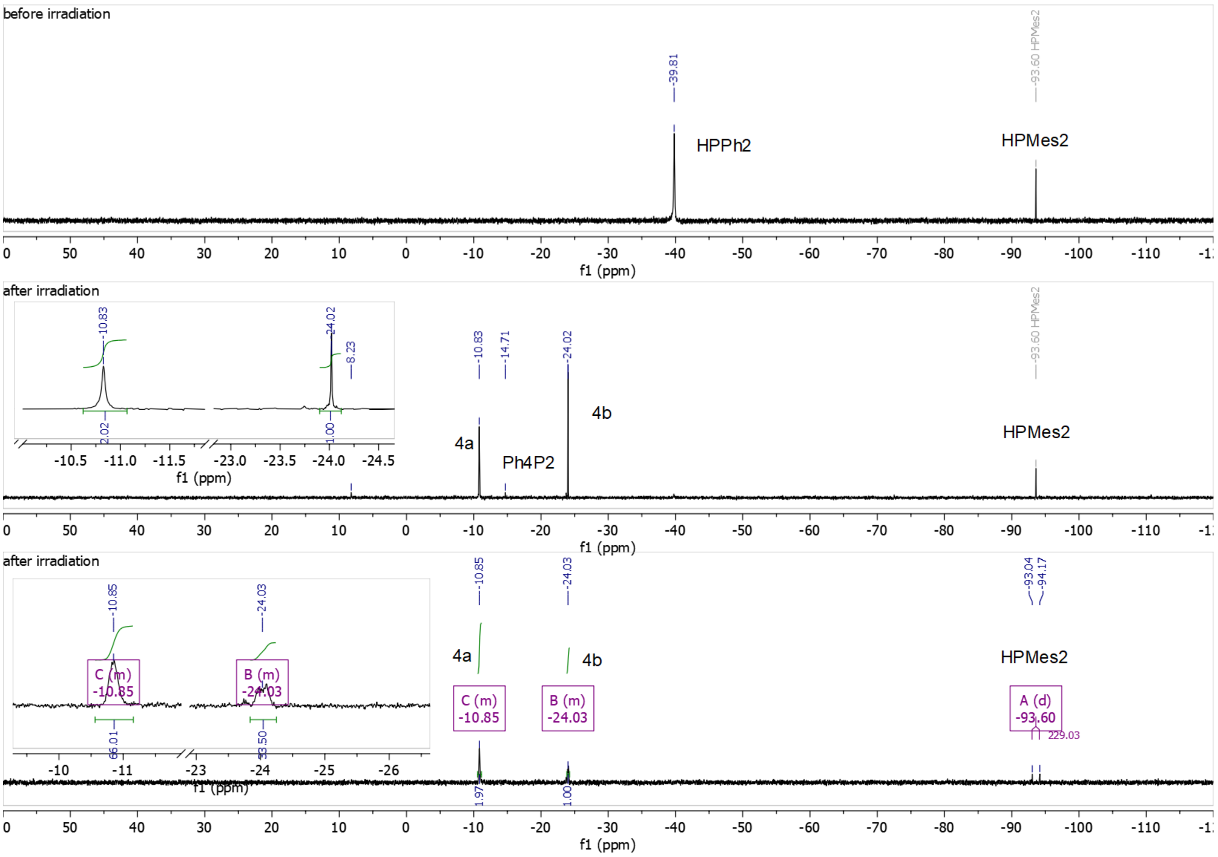

Figure S 4: $^{31}P\{^{1}H\}$ NMR spectra (top and mid) and ^{31}P NMR spectrum (bottom) of $HPPh_2$, PhCCH (**3**) and **2c** (5 mol-%) in THF-d_8 before and after 16 h irradiation. **A**: $HPMes_2$, **B**: (*Z*)-**4** (**4b**, 34%) and **C**: (*E*)-**4** (**4a**, 65%). The resonance at –14.7 ppm is assigned to trace amounts of Ph_4P_2.[13]

Integration of the ^{31}P NMR resonances indicates a *E*/*Z*-ratio of 2:1 which, with an ^{1}H NMR yield of 31 % for **4b**, indicates the formation of **4a** in 61 % yield.

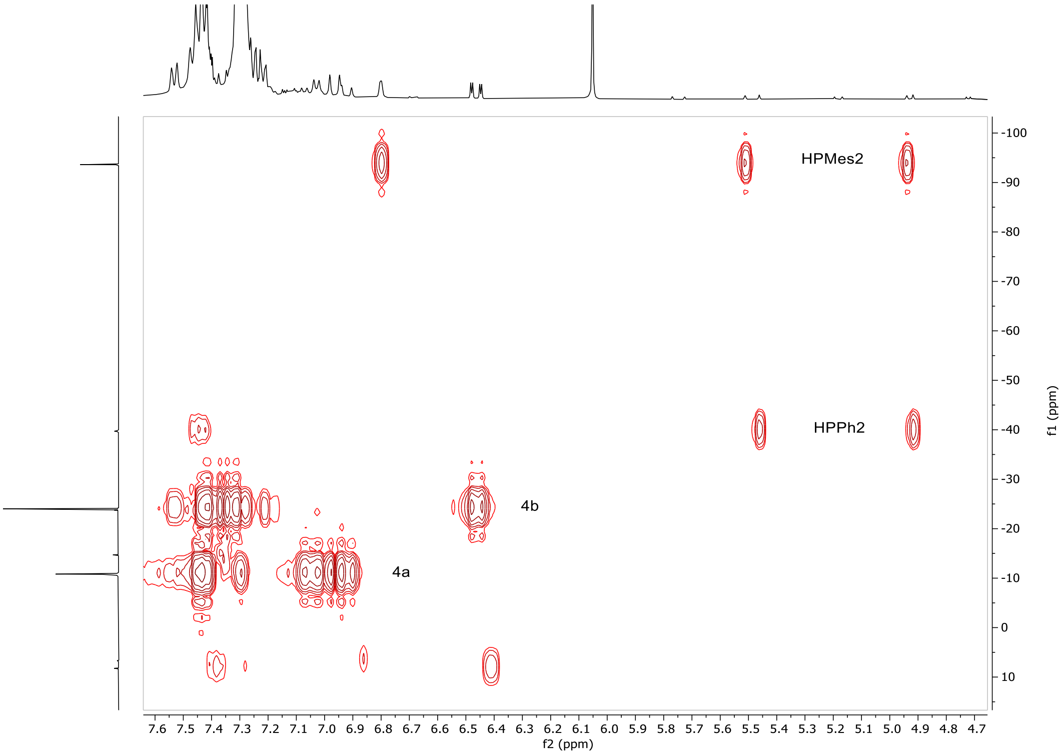

Figure S 5: (^{1}H,^{31}P)-HMBC NMR spectrum of $HPPh_2$, PhCCH (**3**) and **2c** (5 mol-%) in THF-d_8 after 16 h irradiation in THF-d_8.

(E,Z)-P,P-Diphenyl(*ω*-styryl)phosphine (4a + 4b) with 2 mol-% 2c

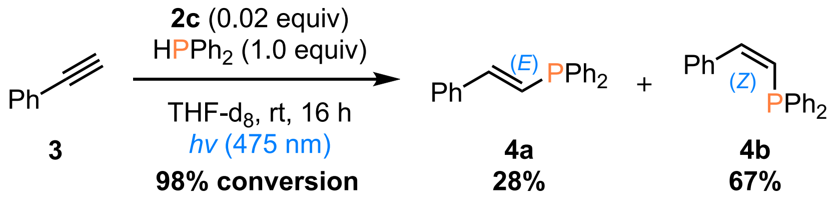

Scheme S 4: NMR scale photocatalyzed hydrophosphination of phenylacetylene (**3**) with diphenylphosphine ($HPPh_2$) using 2 mol-% [Cu(^{Cy}cAAC)($PMes_2$)] (**2c**) in THF-d_8. . Conversion based on consumption of diphenylphosphine

According to the general procedure, **2c** (**2 mol-%**), 1,3,5-trimethoxybenzene (0.33 equiv), $HPPh_2$ (1.0 equiv) and PhCCH (1.0 equiv) were combined in anhydrous THF-d_8.

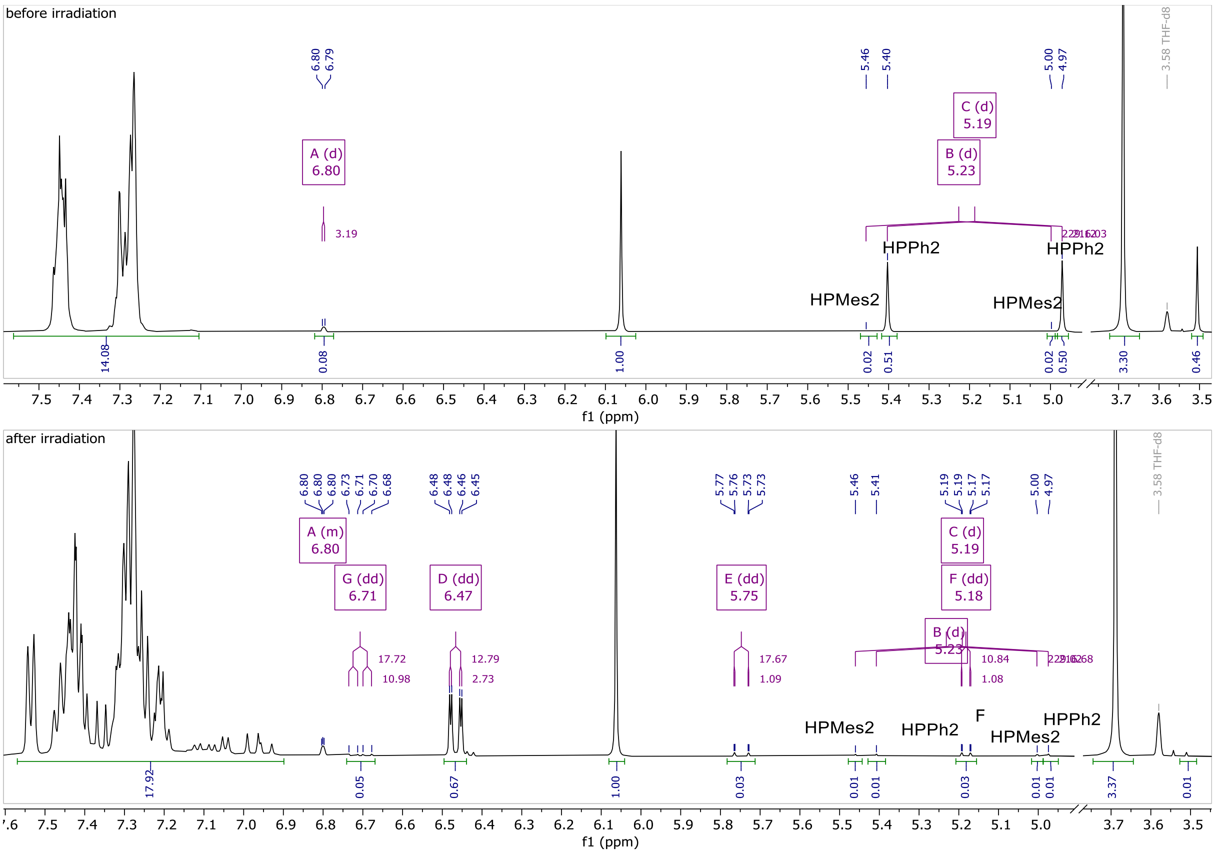

Figure S 6: ^{1}H NMR spectra of $HPPh_2$, PhCCH and **2c** (2 mol-%) in THF-d_8 before and after 16 h irradiation Signal assignment **A** + **B**: *H*-$PMes_2$ (5.23 ppm, d, J = 229 Hz, product of ligand exchange, 10 %), **C**: *H*-PPh_2 (5.19 ppm, d, J = 218 Hz), **D**: (*Z*)-**4** (**4b**, 67%), **E** – **G**: $H_{Styrene}$ (**6**, 5%). ^{1}H-resonances of (*E*)-**4** (**4a**) are overlapping with catalyst signals and other impurities. Yield determined by ^{31}P NMR spectroscopy (see below).

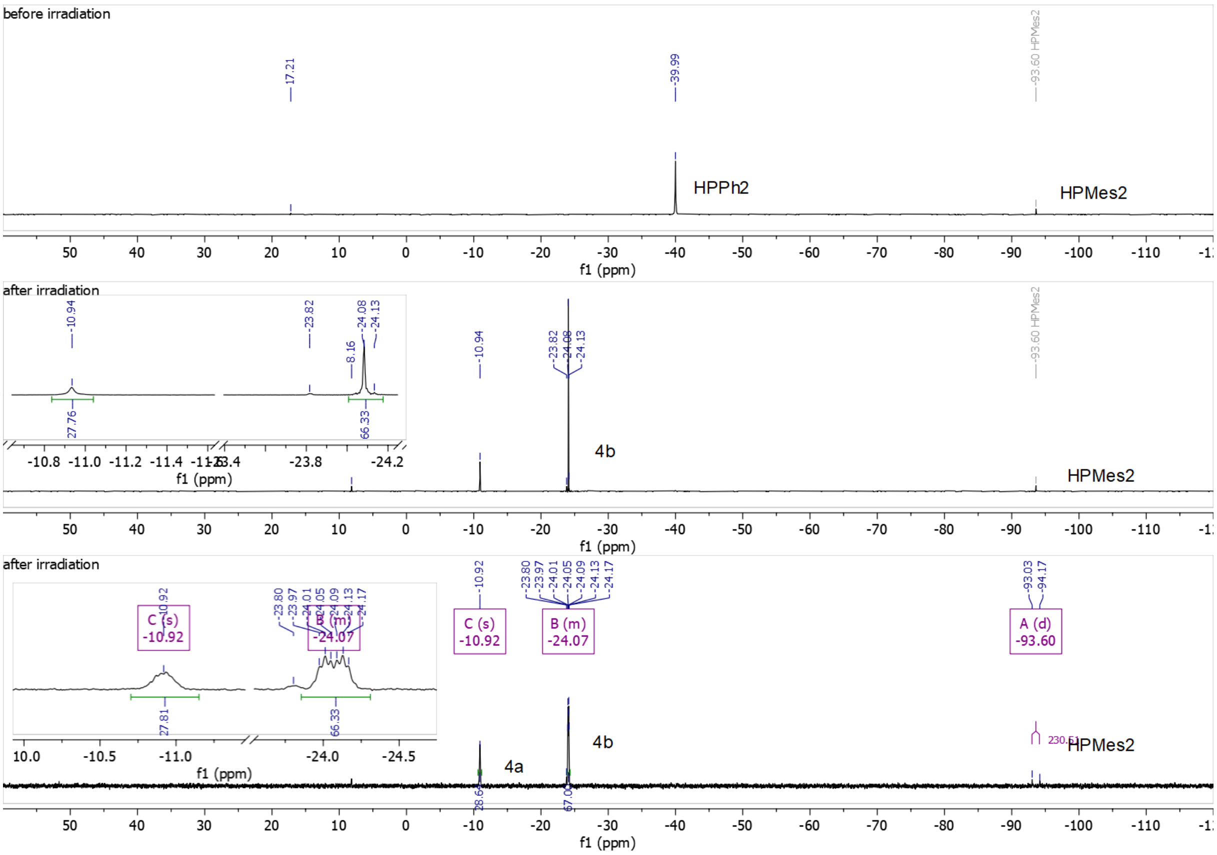

Figure S 7: $^{31}P\{^{1}H\}$ NMR spectra (top and mid) and ^{31}P NMR spectrum (bottom) of $HPPh_2$, PhCCH (**3**) and **2c** (2 mol-%) in THF-d_8 before and after 16 h irradiation. **A**: $HPMes_2$, **B**: (*Z*)-**4** (**4b**, 66%) and **C**: (*E*)-**4** (**4a**, 28%). The resonance at –14.7 ppm is assigned to trace amounts of Ph_4P_2.[13]

Integration of the ^{31}P NMR resonances indicates a *E*/*Z*-ratio of 2:1 which, with an ^{1}H NMR yield of 67 % for **4b**, indicates the formation of **4a** in 28 % yield.

Kinetic investigation of the reaction:

Kinetic studies were conducted with 2 mol-% of **2c** and an internal standard (trimethoxybenzene, $I_{TMB} \equiv 1.000$).

The conversion of diphenylphosphine $C(t)$ was determined by:

$$C(t) = 1 - \frac{I(t)}{I(0)}$$

The yield Y_x of compound x was calculated *via* the quotient of half of the sum of the integrals of the starting materials I_{PhCCH} and I_{HPPh2} and the integral of the compound I_x:

$$Y_x = 2\frac{I_x}{I_{PhCCH} + I_{HPPh2}}$$

The yield of the (*E*)-isomer could not be determined bei ^{1}H NMR spectroscopy due to severe overlap with other resonances and was therefore calculated by the integral ratio of the ^{31}P resonances calibrating the signal of the (*Z*)-isomer to the value determined in the ^{1}H NMR spectrum.

The total non-aromatic proton yield Γ was used as a checksum to verify the integrity of all assumptions. Γ is calculated according to:

$$\Gamma = \frac{\sum_x I_x}{I_{PhCCH} + I_{HPPh2}}$$

Γ should always be equal to 1 since the total count of non-aromatic protons should not change during the reaction.

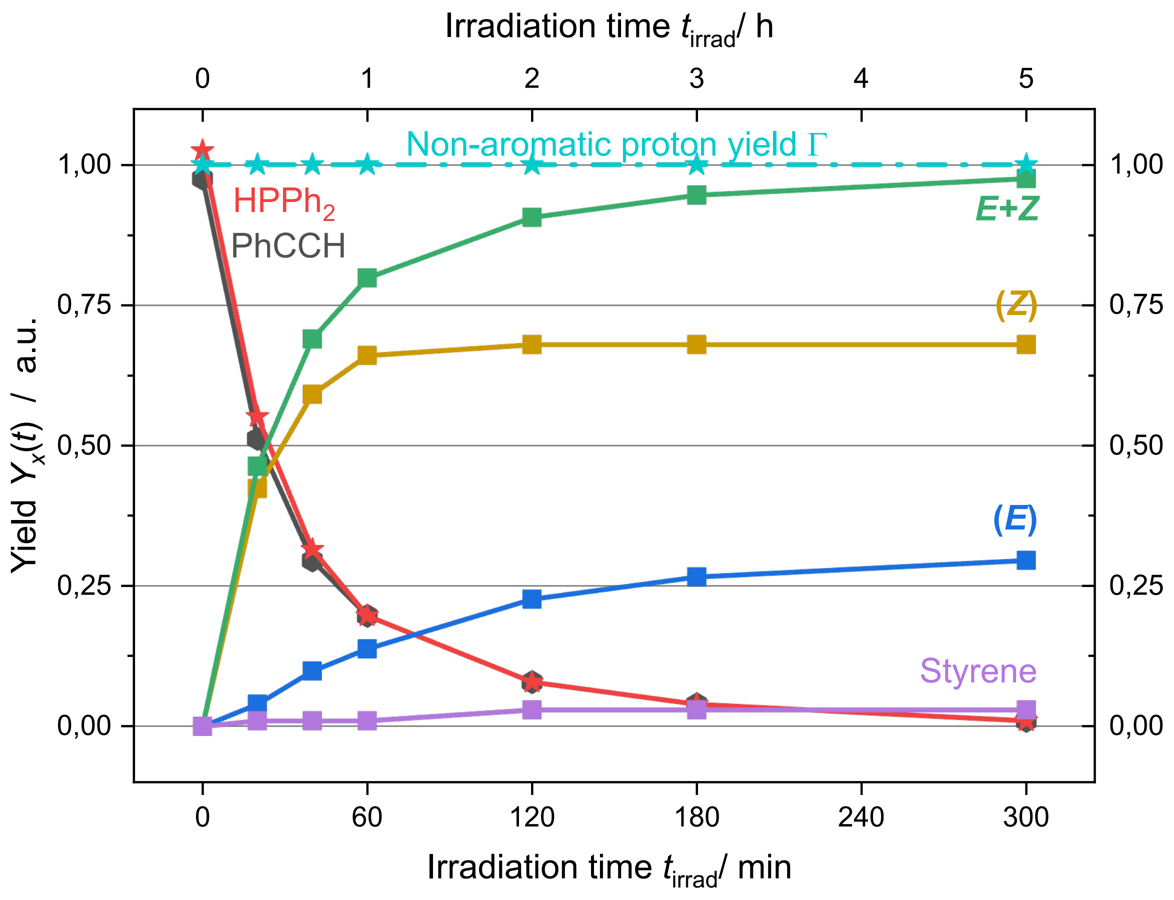

Figure S 8: Time-conversion plot showing the NMR yield of the different species observed (see above) during the reaction of diphenylphosphine ($HPPh_2$) and phenylacetylene (**3**) catalyzed by **2c** (2 mol-%) under irradiation of light (475 nm). The amount of non-aromatic protons should be equal at all times.

(*E*,*Z*) -*P*,*P*-Diphenyl(*ω*-styryl)phosphine (4a + 4b) with 2 mol-% 2c in two cylces:

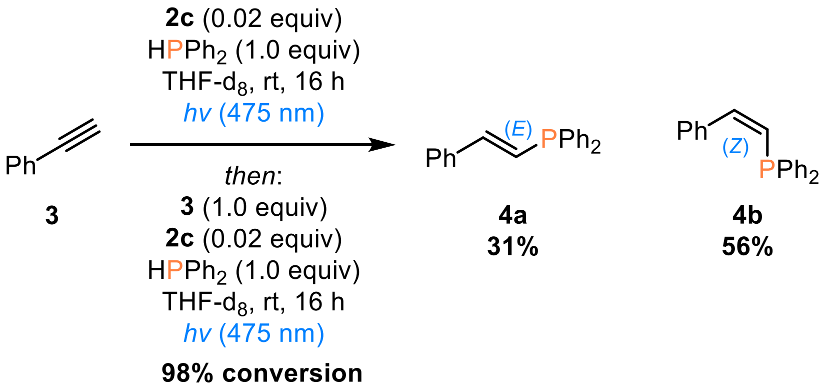

Scheme S 5: NMR scale photocatalyzed hydrophosphination of phenylacetylene (**3**) with diphenylphosphine ($HPPh_2$) using 2 mol-% [Cu(^{Cy}cAAC)($PMes_2$)] (**2c**) in THF-d_8. And readdition of substrates to the reaction mixture. Conversion based on consumption of diphenylphosphine and yields are referring to the total amount of starting material added.

First, according to the general procedure, **2c** (**2 mol-%**), 1,3,5-trimethoxybenzene (0.33 equiv), $HPPh_2$ (1.0 equiv) and PhCCH (1.0 equiv) were combined in anhydrous THF-d_8. After irradiation for 16 h, NMR spectra were recorded and PhCCH (1.0 equiv) and $HPPh_2$ (1.0 equiv) were added. The solution was irradiated again for 16 h and NMR spectra were recorded:

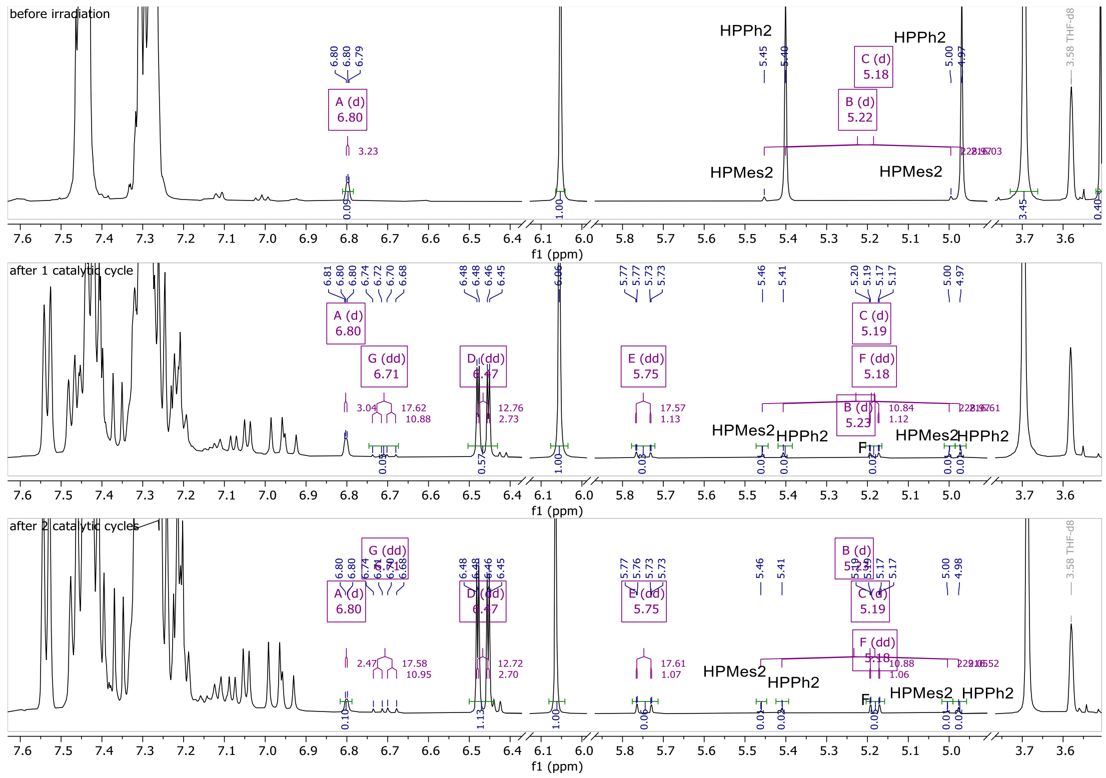

Figure S 9: ^{1}H NMR spectra of $HPPh_2$, PhCCH and **2c** (2 mol-%) in THF-d_8 before irradiation (above) and after one catalytic cycle (mid) and after two catalytic cycles (below Signal assignment **A** + **B**: *H*-$PMes_2$ (5.23 ppm, d, J = 229 Hz, product of ligand exchange, 10 %), **C**: *H*-PPh_2 (5.19 ppm, d, J = 218 Hz), **D**: (*Z*)-**4** (**4b**, 67%), **E** – **G**: $H_{Styrene}$ (**6**, 5%). ^{1}H-resonances of (*E*)-**4** (**4a**) are overlapping with catalyst signals and other impurities. Yield determined by ^{31}P NMR spectroscopy (see below).

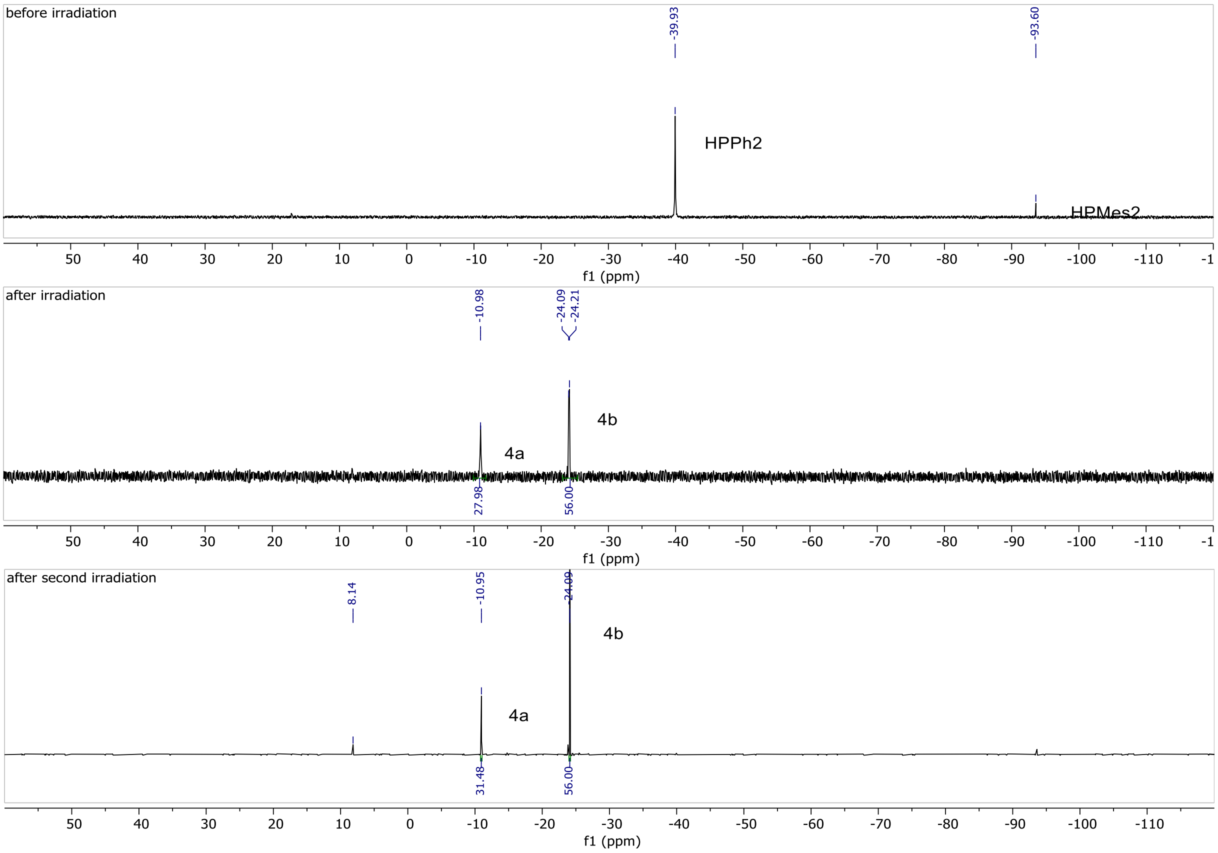

Figure S 10: $^{31}P\{^{1}H\}$ NMR spectra of $HPPh_2$, PhCCH (**3**) and **2c** (2 mol-%) in THF-d_8 before and after 16 h irradiation. **A**: $HPMes_2$, **B**: (*Z*)-**4** (**4b**, 56%) and **C**: (*E*)-**4** (**4a**, 32%).

(E,Z)-P,P-Diphenyl(*ω*-styryl)phosphine (4a + 4b) with 2 mol-% 2d

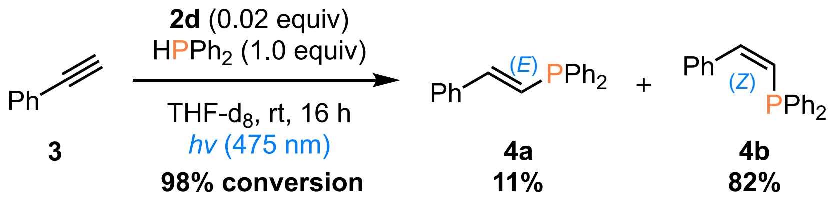

Scheme S 6: NMR scale photocatalyzed hydrophosphination of phenylacetylene (**3**) with diphenylphosphine ($HPPh_2$) using 2 mol-% [Cu(^{Ment}cAAC)($PMes_2$)] (**2d**) in THF-d_8. . Conversion based on consumption of diphenylphosphine.

According to the general procedure, **2d** (**2 mol-%**), 1,3,5-trimethoxybenzene (0.33 equiv), $HPPh_2$ (1.0 equiv) and PhCCH (1.0 equiv) were combined in anhydrous THF-d_8.

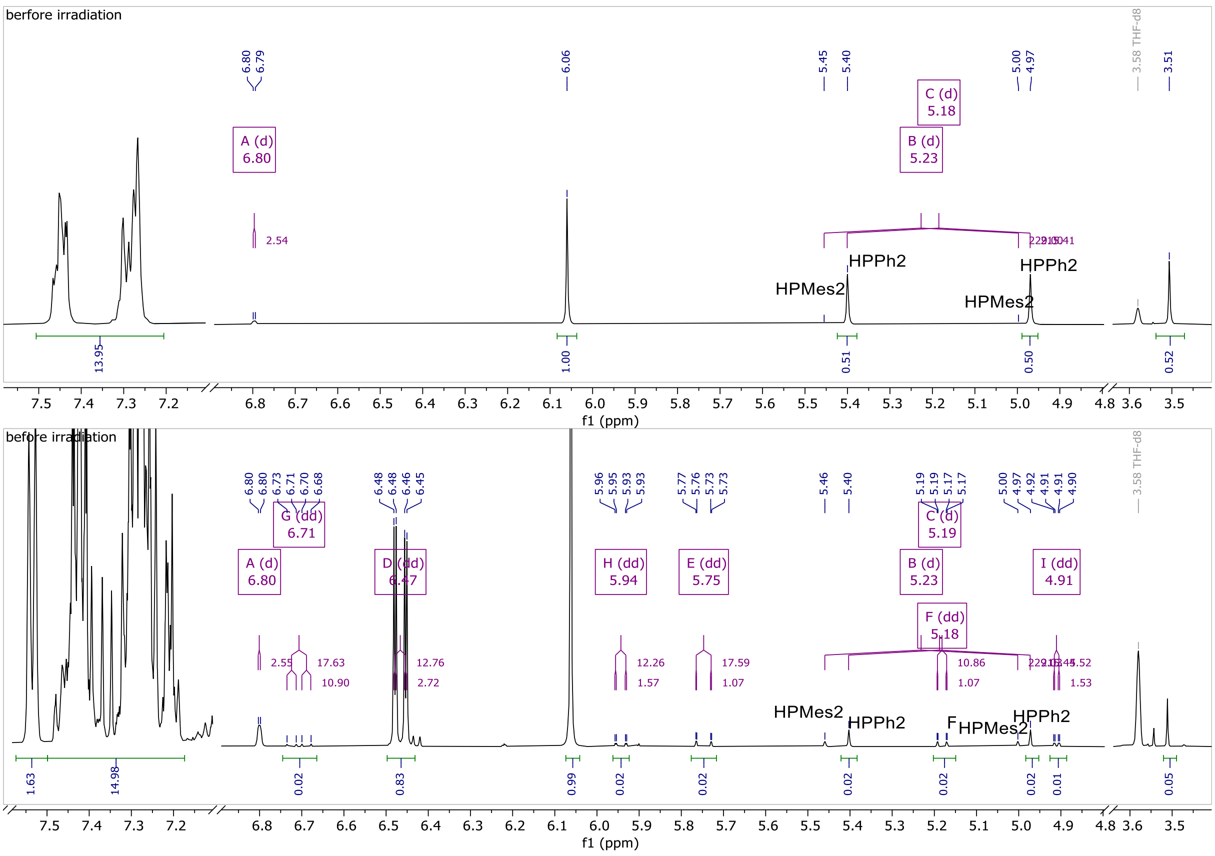

Figure S 11: ^{1}H NMR spectra of $HPPh_2$, PhCCH and **2d** (2 mol-%) in THF-d_8 before and after 16 h irradiation: Signal assignment **A** + **B**: *H*-$PMes_2$ (5.23 ppm, d, J = 229 Hz, product of ligand exchange, 10 %), **C**: *H*-PPh_2 (5.19 ppm, d, J = 218 Hz), **D**: (*Z*)-**4** (**4b**, 82%), **E** – **G**: $H_{Styrene}$ (**6**, 2%), **H** – **I**: **5** (1 %). ^{1}H-resonances of (*E*)-**4** (**4a**) are overlapping with catalyst signals and other impurities. Yield determined by ^{31}P NMR spectroscopy (see below).

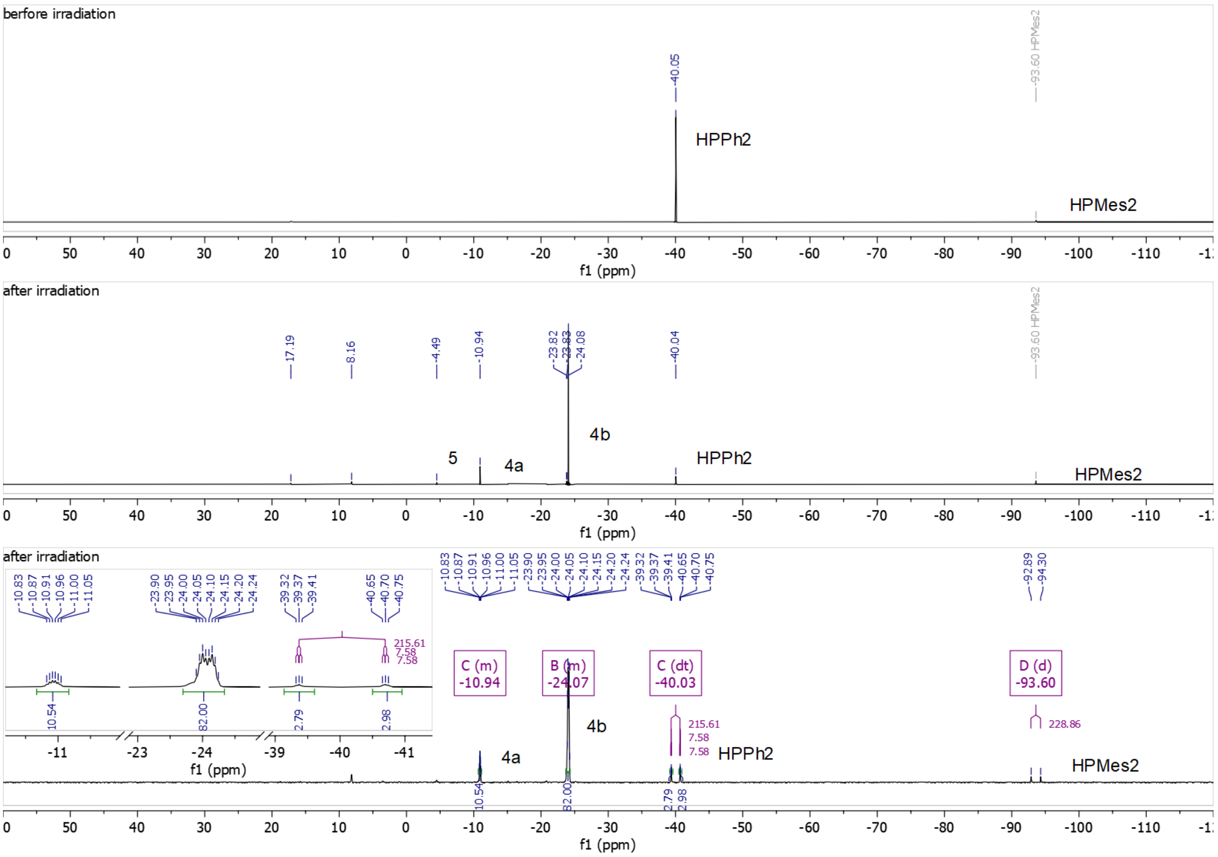

Figure S 12: $^{31}P\{^{1}H\}$ NMR spectra (top and mid) and ^{31}P NMR spectrum (bottom) of $HPPh_2$, PhCCH (**3**) and **2d** (2 mol-%) in THF-d_8 before and after 16 h irradiation. **A**: $HPMes_2$, **B**: (*Z*)-**4** (**4b**, 11%) and **C**: (*E*)-**4** (**4a**, 82%). The resonance at $\delta = -4.5$ ppm is assigned to the *gem*-addition product **5** and the resonance at $\delta = -14.7$ ppm is assigned to trace amounts of Ph_4P_2.[13]

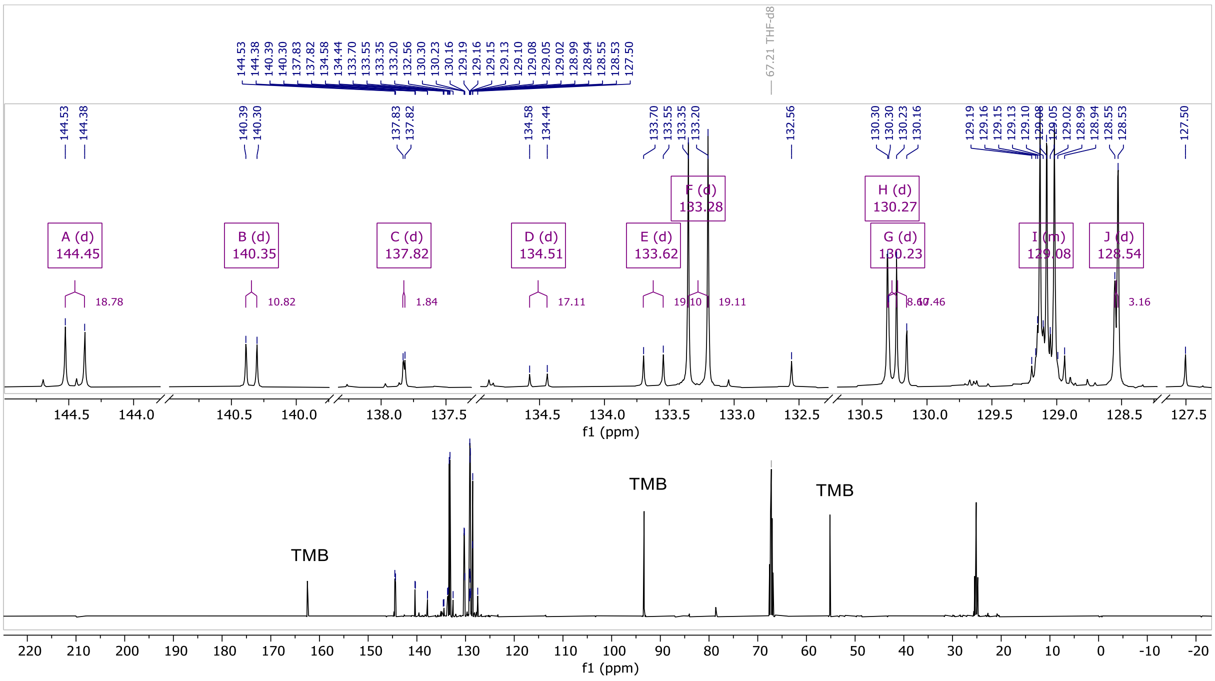

Figure S 13: $^{13}C\{^{1}H\}$ NMR spectra of $HPPh_2$, PhCCH and **2d** (2 mol-%) in THF-d_8 after 16 h irradiation. TMB = 1,3,5-trimethoxybenzene (internal standard).

Signal assignment to the species **4 – 6** was done by (^{1}H,^{1}H)-COSY and (^{1}H,^{31}P)-HMBC experiments:

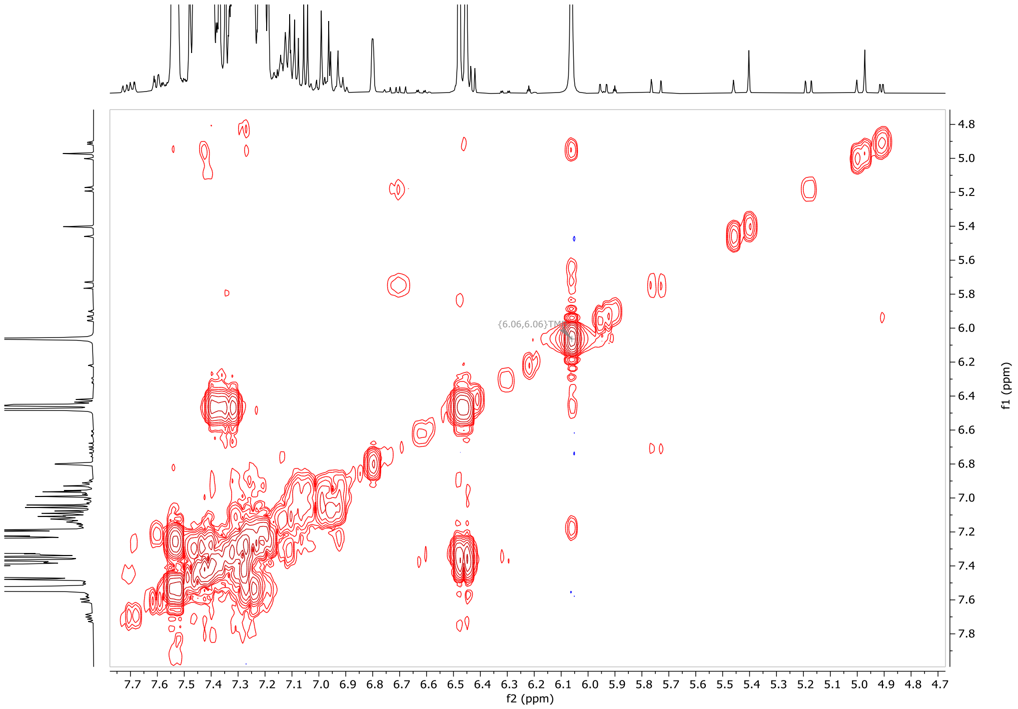

Figure S 14: (^{1}H,^{1}H)-COSY NMR spectrum of $HPPh_2$, PhCCH (**3**) and **2d** (2 mol-%) in THF-d_8 after 16 h irradiation in THF-d_8.

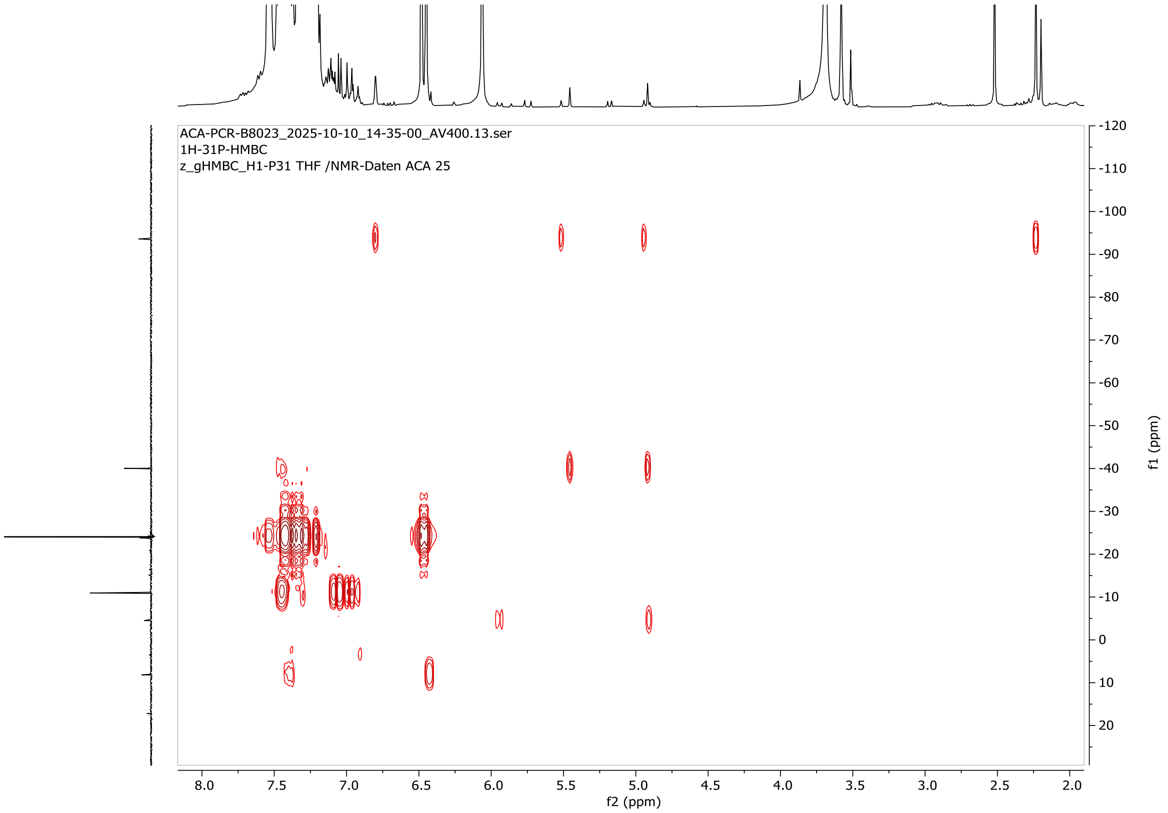

Figure S 15: (^{1}H,^{31}P)-HMBC NMR spectrum of $HPPh_2$, PhCCH (**3**) and **2d** (2 mol-%) in THF-d_8 after 16 h irradiation in THF-d_8.

(E,Z)-P,P-Diphenyl(*ω*-styryl)phosphine (4a + 4b) with 10 mol-% 2c in C_6D_6

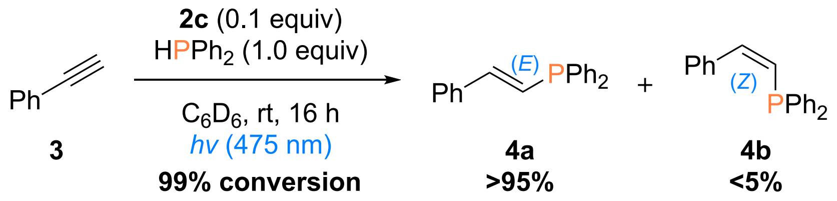

Scheme S 7: NMR scale photocatalyzed hydrophosphination of phenylacetylene (**3**) with diphenylphosphine ($HPPh_2$) using 10 mol-% [Cu(^{Cy}cAAC)($PMes_2$)] (**2c**) in C_6D_6.. Conversion based on consumption of diphenylphosphine.

According to the general procedure, **2c** (**10 mol-%**), 1,3,5-trimethoxybenzene (0.33 equiv), $HPPh_2$ (1.0 equiv) and PhCCH (1.0 equiv) were combined in anhydrous C_6D_6.

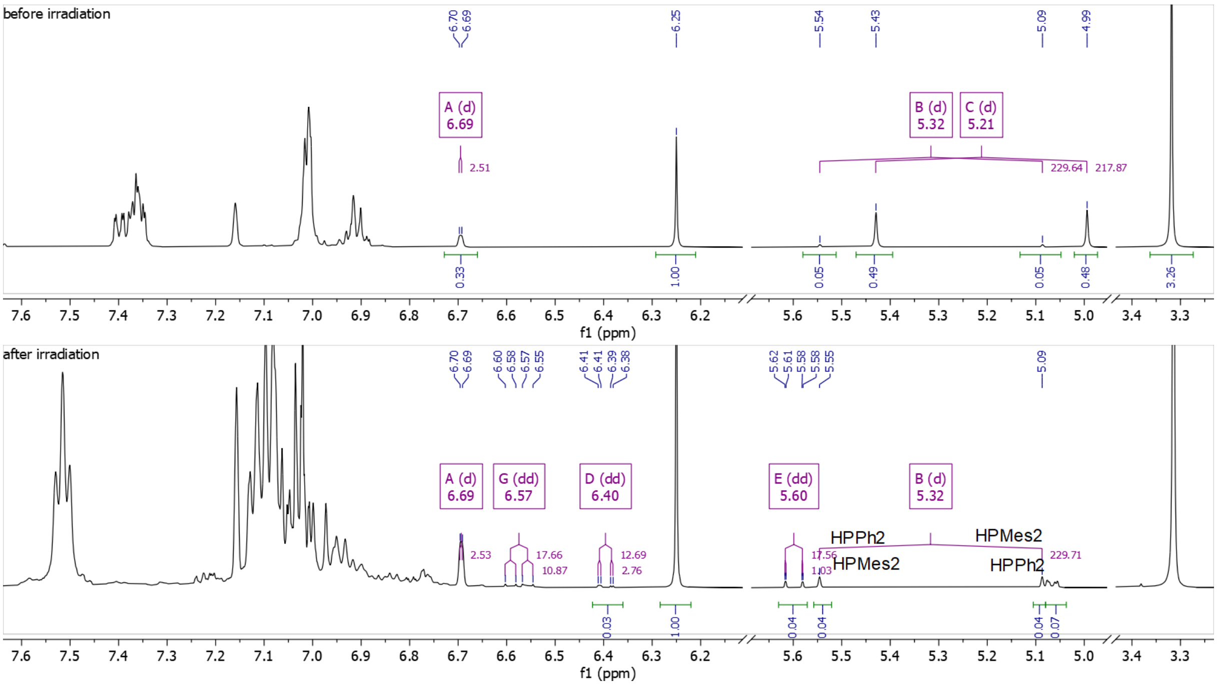

Figure S 16: ^{1}H NMR spectra of $HPPh_2$, PhCCH and **2c** (10 mol-%) in C_6D_6 before and after 16 h irradiation: Signal assignment **A** + **B**: *H*-$PMes_2$ (5.23 ppm, d, J = 229 Hz, product of ligand exchange, 10 %), **C**: *H*-PPh_2 (5.19 ppm, d, J = 218 Hz), **D**: (*Z*)-**4** (**4b**, <5%), **E** + **G**: $H_{Styrene}$ (**6**, 3%). ^{1}H-resonances of (*E*)-**4** (**4a**) are overlapping with catalyst signals and other impurities. Yield determined by ^{31}P NMR spectroscopy (see below).

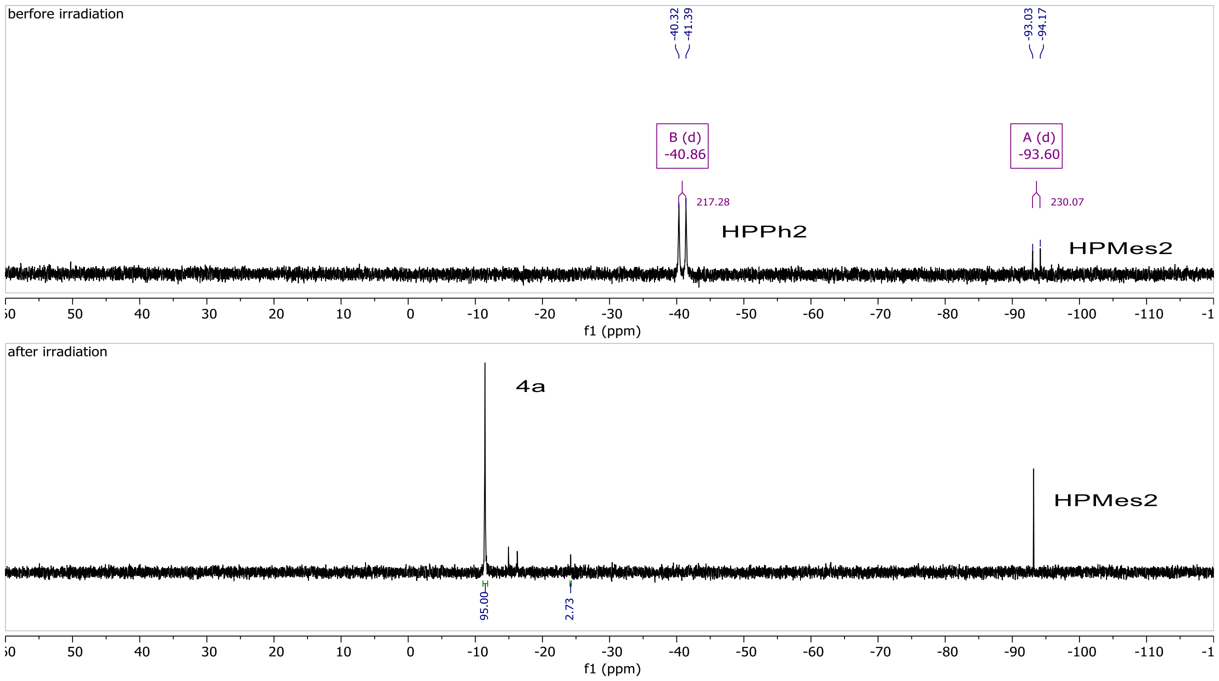

Figure S 17: ^{31}P NMR spectra of $HPPh_2$, PhCCH (**3**) and **2c** (10 mol-%) in C_6D_6 before and after 16 h irradiation. **A**: $HPMes_2$, **B**: (*Z*)-**4** (**4b**, <5%) and **C**: (*E*)-**4** (**4a**, >95%).

(E,Z)-P,P-Diphenyl(*ω*-styryl)phosphine (4a + 4b) with 2 mol-% 2c in C_6D_6:

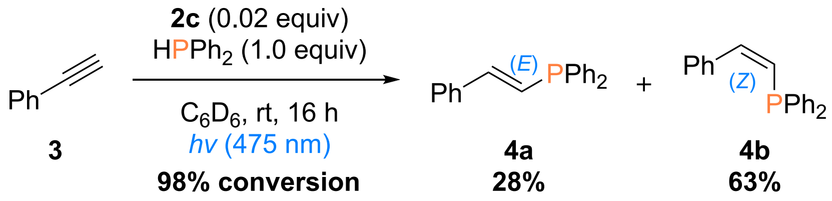

Scheme S 8: NMR scale photocatalyzed hydrophosphination of phenylacetylene (**3**) with diphenylphosphine ($HPPh_2$) using 2 mol-% [Cu(^{Cy}cAAC)($PMes_2$)] (**2c**) in C_6D_6. . Conversion based on consumption of diphenylphosphine.

According to the general procedure, **2c** (**2 mol-%**), 1,3,5-trimethoxybenzene (0.33 equiv), $HPPh_2$ (1.0 equiv) and PhCCH (1.0 equiv) were combined in anhydrous C_6D_6.

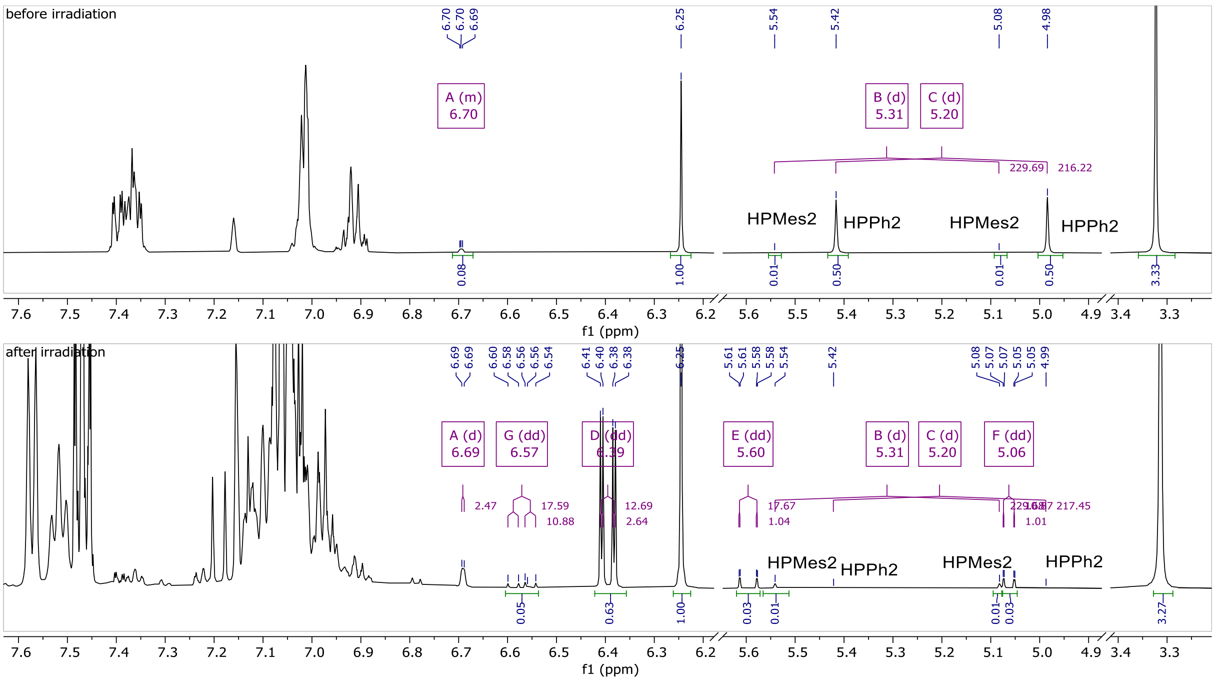

Figure S 18: ^{1}H NMR spectra of $HPPh_2$, PhCCH and **2c** (2 mol-%) in C_6D_6 before and after 16 h irradiation: Signal assignment **A** + **B**: *H*-$PMes_2$ (5.23 ppm, d, J = 229 Hz, product of ligand exchange, 10 %), **C**: *H*-PPh_2 (5.19 ppm, d, J = 218 Hz), **D**: (*Z*)-**4** (**4b**, 63%), **E** – **G**: $H_{Styrene}$ (**6**, 3%). ^{1}H-resonances of (*E*)-**4** (**4a**) are overlapping with catalyst signals and other impurities. Yield determined by ^{31}P NMR spectroscopy (see below).

^{31}P NMR spectroscopy (203 MHz, THF-d_8) of the reaction mixture before and after irradiation:

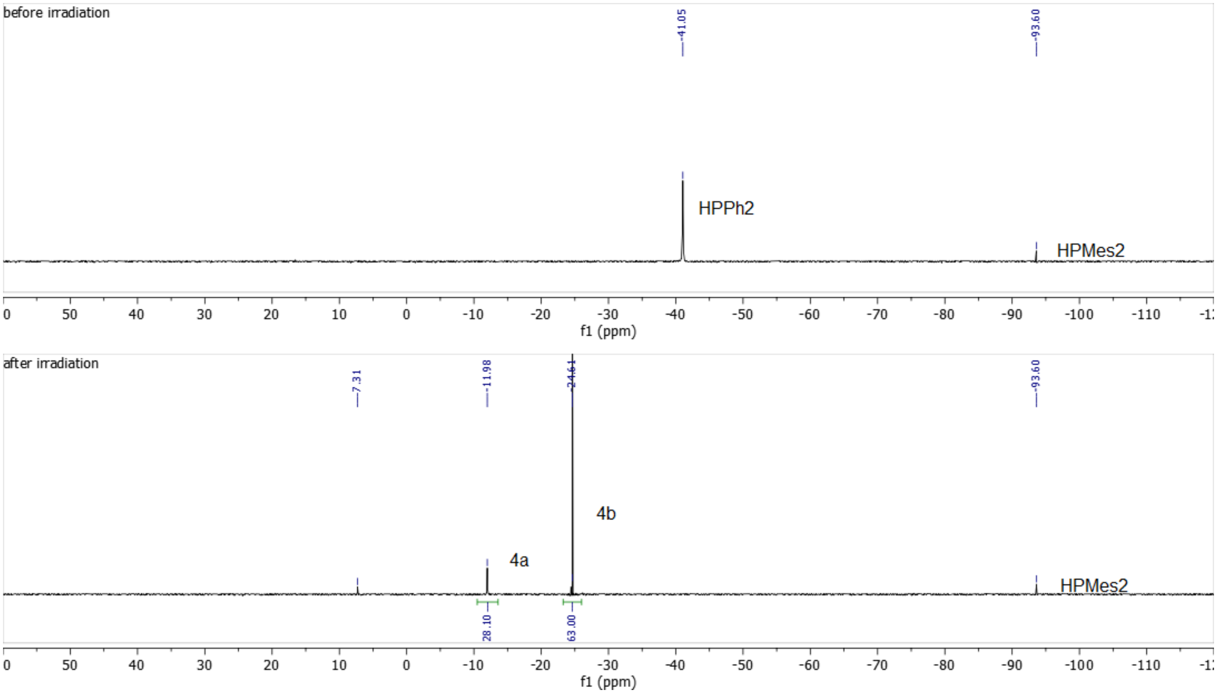

Figure S 19: $^{31}P\{^{1}H\}$ NMR spectra of $HPPh_2$, PhCCH (**3**) and **2c** (2 mol-%) in C_6D_6 before and after 16 h irradiation. **A**: $HPMes_2$, **B**: (*Z*)-**4** (**4b**, 63%) and **C**: (*E*)-**4** (**4a**, 28%).

Reaction with dimesitylphosphine:

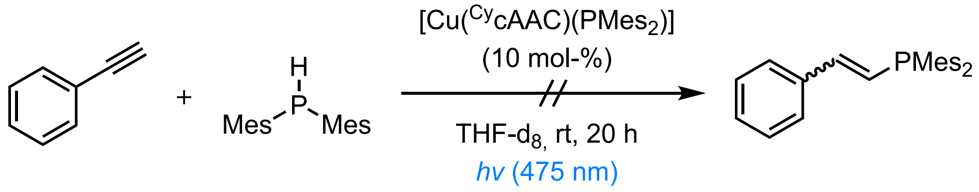

3 **11** **12**

Scheme S 9: NMR scale photocatalyzed hydrophosphination of phenylacetylene (**3**) with dimesitylphosphine ($HPMes_2$) using 10 mol-% [Cu(^{Cy}cAAC)($PMes_2$)] (**2d**) in THF-d_8. . Conversion based on consumption of dimesitylphosphine.

According to the general procedure, **2c**, $HPMes_2$ and phenylacetylene were dissolved in dry THF-d_8 and the mixture was irradiated for 20 h and NMR spectra were recorded.

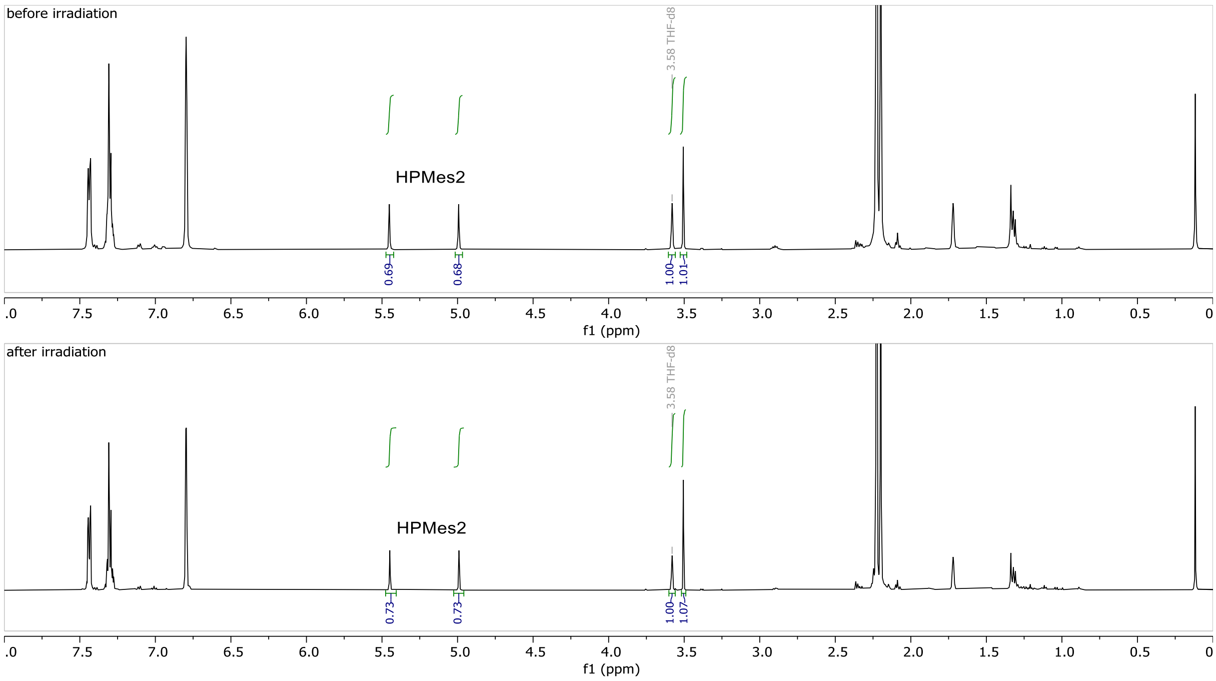

Figure S 20: ^{1}H NMR spectra of $HPMes_2$, PhCCH and **2c** in THF-d_8 before and after 20 h irradiation.

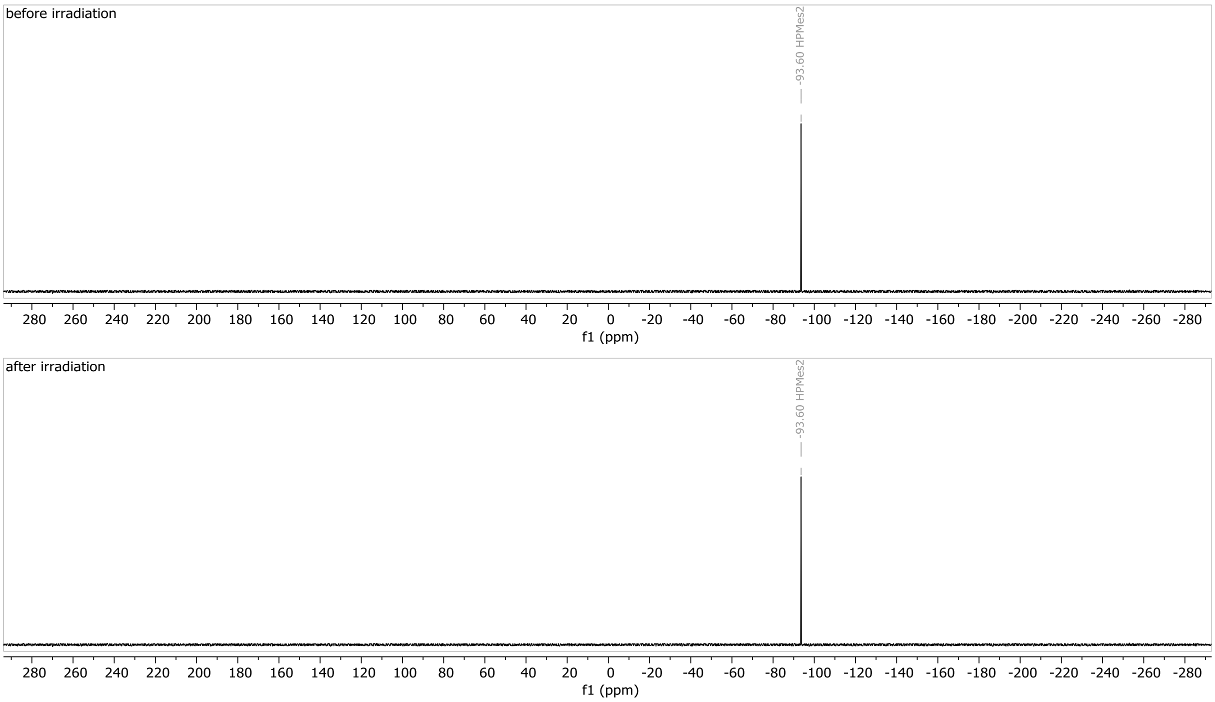

Figure S 21: $^{31}P\{^{1}H\}$ NMR spectra of $HPMes_2$, PhCCH and **2c** in THF-d_8 before and after 20 h irradiation.

Reaction with catalyst under exclusion of light:

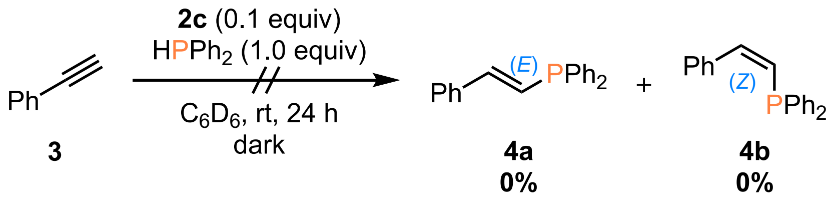

Scheme S 10: NMR scale reaction of phenylacetylene (**3**) with dimesitylphosphine ($HPMes_2$) using 10 mol-% [Cu(^{Cy}cAAC)($PMes_2$)] (**2d**) in C_6D_6 under exclusion of light for 24 h.. Conversion based on consumption of dimesitylphosphine. No onversion was observed.

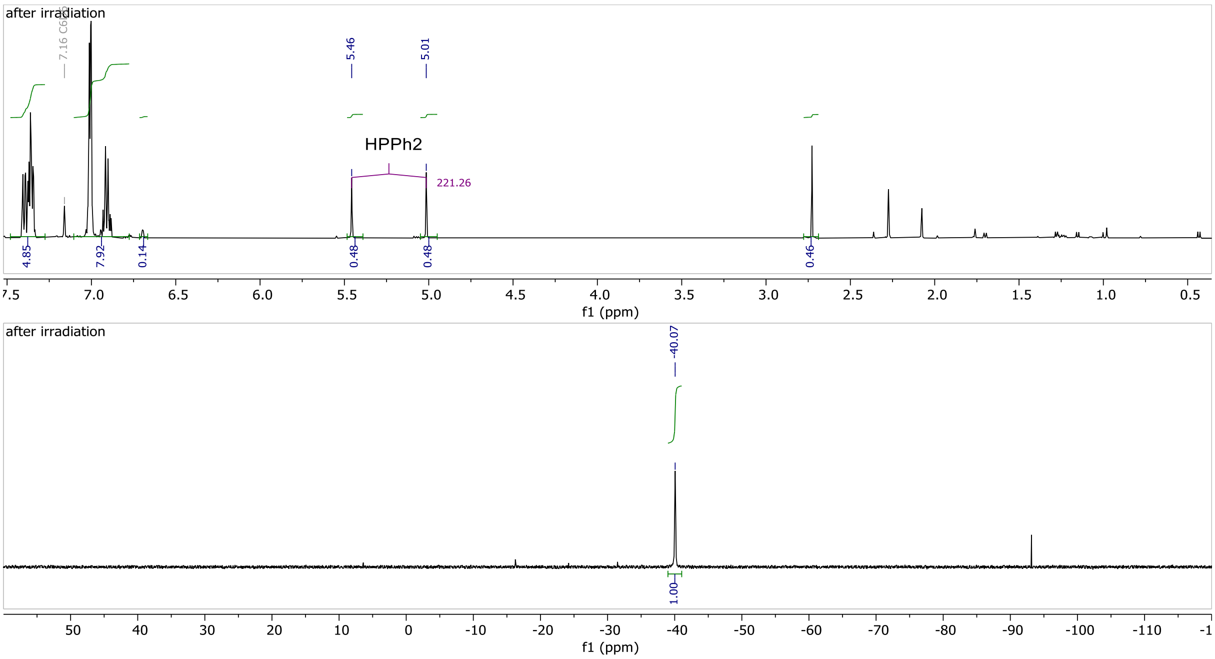

Figure S 22: ^{1}H NMR and ^{31}P{^{1}H} NMR spectra of $HPMes_2$, PhCCH and **2c** (10 mol-%) in C_6D_6 after 24 h under exclusion of light.

Irradiation in absence of a catalyst:

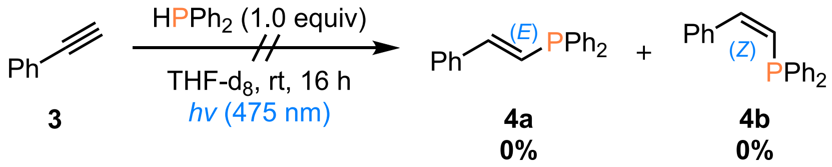

Scheme S 11: NMR scale reaction of phenylacetylene (**3**) with dimesitylphosphine ($HPMes_2$) without any catalyst in THF-d_8.

$HPPh_2$ (1.0 equiv) and phenylacetylene (**3,** 1.0 equiv) were dissolved in dry THF-d_8. The solution was irradiated for 16 h. No conversion was observed.

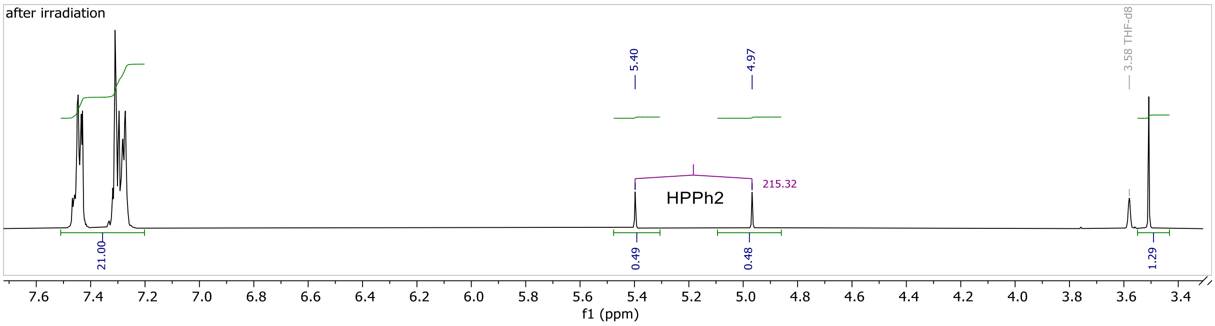

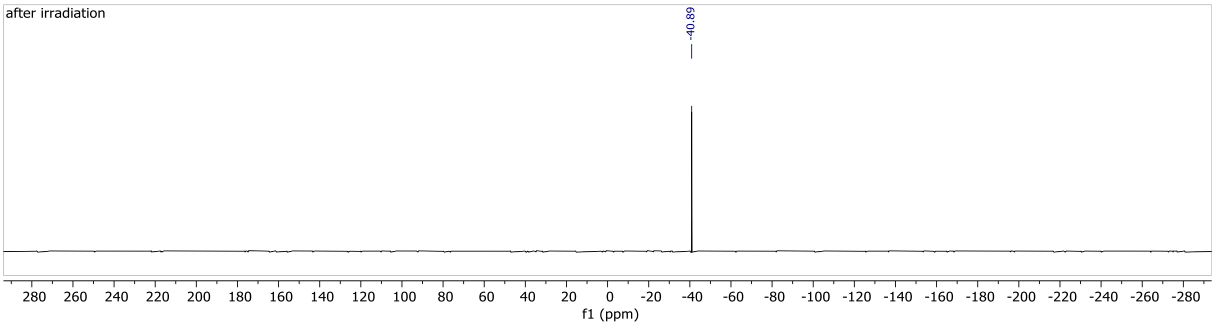

Figure S 23: ^{1}H NMR (top) and $^{31}P\{^{1}H\}$ NMR (bottom) spectra of $HPMes_2$, PhCCH and in THF-d_8 after 16 h irradiation.

Reaction between 2c and phenylacetylene (3):

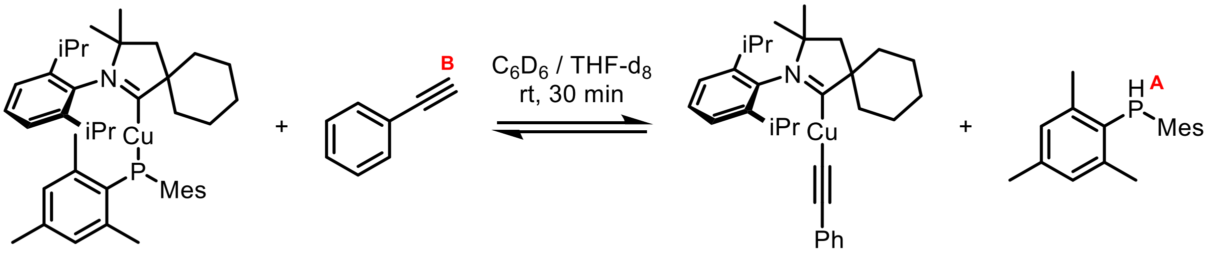

2c **3** **13** **11**

Scheme S 12: Protolysis of **2c** by phenylacetylene (**3**)

In an argon filled glovebox, [Cu(^{Cy}cAAC)($PMes_2$)] (**2c**, 10.0 mg, 15.2 µmol, 1.0 equiv) was dissolved in dry THF-d_8 (0.5 mL). A freshly prepared solution of phenylacetylene in C_6D_6 (c = 0.98 mol L^{-1}, v/v, 16 µL, 15.2 µmol, 1.03 equiv) was added. After 30 min under exclusion of light, NMR spectra (^{1}H and {^{1}H}^{31}P) were recorded revealing a 1:0.75 ratio of free phosphine (**SI-2**) vs. phenylacetylene (**3**).

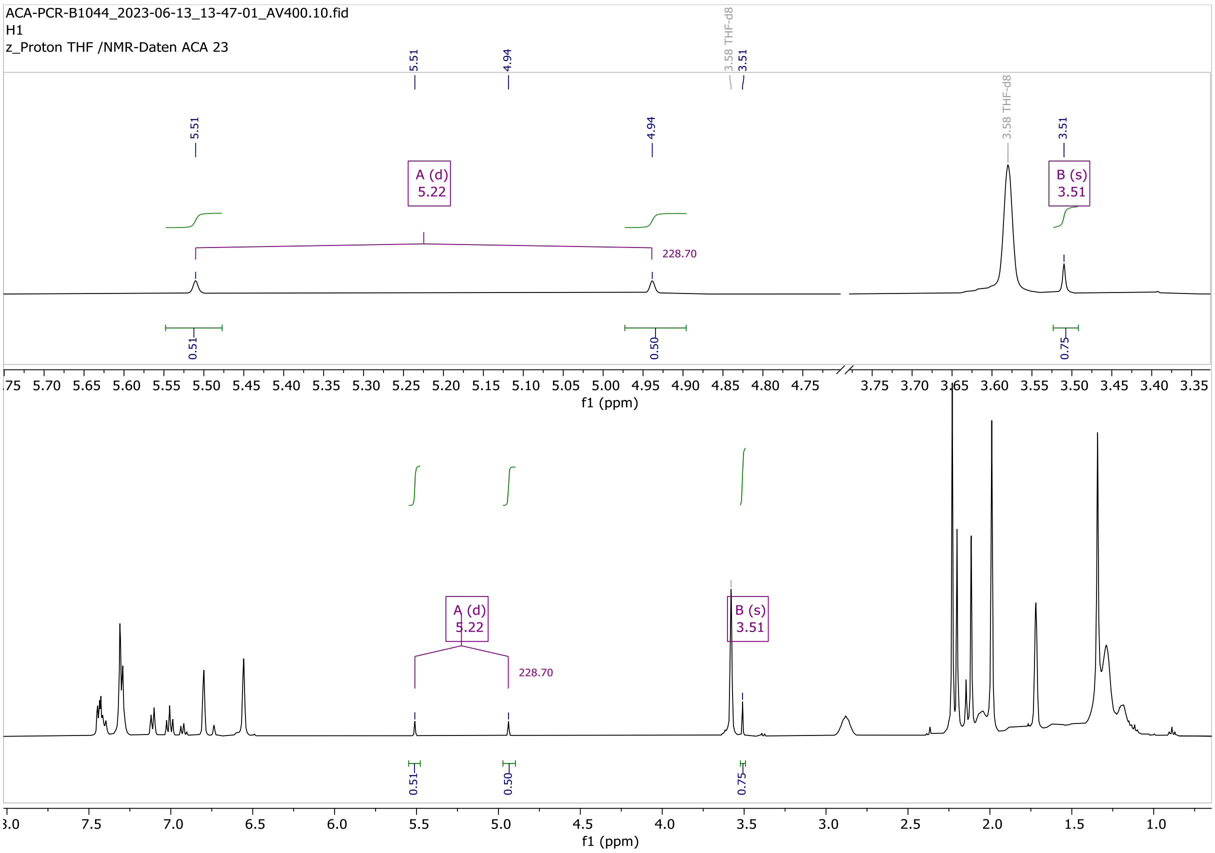

Figure S 24: ^{1}H NMR spectrum of the ligand protolysis reaction (see scheme **S1**).

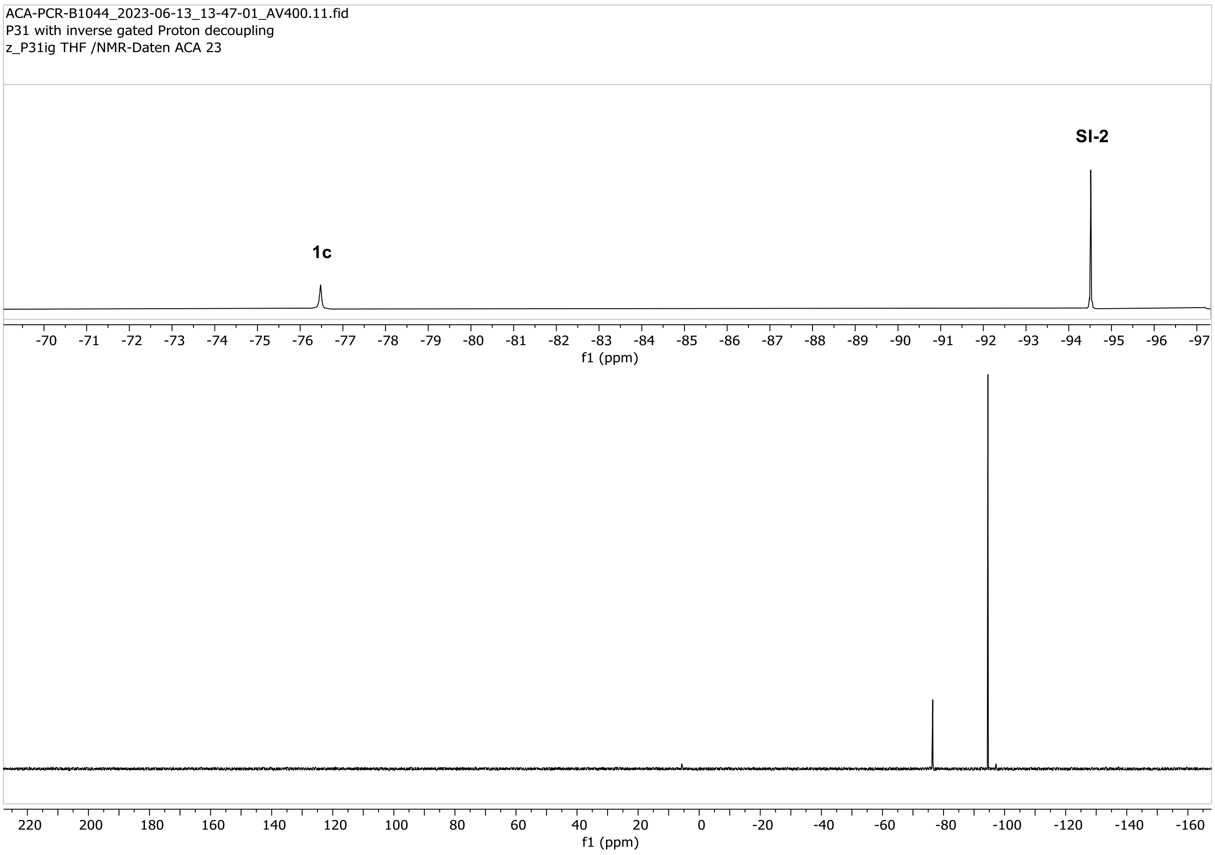

Figure S 25: $\{^1H\}^{31}P$ NMR spectra of the ligand protolysis reaction (see scheme **S1**).

(E,Z)-*P,P*-Diphenyl(*ω*-styryl)phosphine oxide (7a + 7b) by photocatalysis with an *in-situ* formed Carbene Cu(I) phosphide

Chemical Formula: $C_{20}H_{17}OP$
Exact Mass: 304,1017

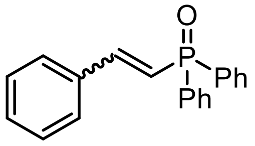

7a (*E*) / **7b** (*Z*)

[Cu(^{Cy}cAAC)Cl] (**1c,** 125 mg, 294 µmol, 0,1 equiv) and $HPPh_2$ (579 µL, 602 mg, 3.23 mmol, 1.1 equiv) were dissolved in THF (20 mL) and a commercial solution of 1-(2,4,6-trimethylphenyl)magnesium bromide in 2-methyltetrahydrofurane (c = 1.0 mol L^{-1}, 294 µL, 294 µmol, 0.1 equiv) was added at 0 °C. After 20 min at this temperature, phenylacetylene (323 µL, 300 mg, 2.66 mmol, 1.0 equiv) was added and the mixture was stirred and irradiated with blue LED light (480 nm) at room temperature for 16 h. After cooling to 0 °C, a solution of H_2O_2 in H_2O (β = 30 wt%, 2 mL) was added dropwise and the mixture was dissolved with CH_2Cl_2 and NaOH (c = 1.0 mol L^{-1}, 20 mL). The phases were separated and the aqueous phase was extracted with CH_2Cl_2 (3 × 20 mL). The combined organic phases were washed with NaOH (c = 1.0 mol L^{-1}, 20 mL) and brine, and dried over $MgSO_4$. After filtration, Celite® was added the organic filtrate and the solvents were removed under reduced pressure. The crude product adsorbed on Celite was purified by flash column chromatography on silica gel (gradient of elution: CH_2Cl_2/MeOH = 1:0 → 99:1) to obtain the product as an inseparable mixture of (*E*)- and (*Z*)-isomers ((*E*)/(*Z*) = 39:10, 0.81 g, 2.66 mmol, 91% combined yield). The (*E*)/(*Z*)

ratio was determined by ^{1}H NMR spectroscopy of the crude product in $CDCl_3$. Crystals suitable for single-crystal x-ray diffraction experiments were obtained in shape of colorless needles by slow vapor diffusion of pentane into a saturated solution of the title compound in THF.

TLC: R_f = 0.15 (CH_2Cl_2/MeOH = 99:1).

FT-IR: (ATR): 3054, 2999, 1673, 1607, 1574, 1496, 1484, 1448, 1437, 1326, 1234, 1176, 1165, 1103, 1071, 1028, 925 cm^{-1}.

^{1}H NMR: (400 MHz, $CDCl_3$, (*E*)-major diastereomer): δ / ppm 7.82 – 7.71 (m, 4H), 7.58 – 7.44 (m, 9H), 7.37 (m, 3H), 6.84 (dd, $^{1}J_{HP}$ = 22.8, J = 17.5 Hz, 1H).

^{13}C{^{1}H} NMR: (101 MHz, $CDCl_3$): δ / ppm 147.6 (d, J = 3.6 Hz, $C_{q,ar}$), 135.3 (d, J = 17.8 Hz), 133.1 (d, J = 105.9 Hz, 2C, $C_{q,ar}$), 132.0 (d, J = 2.8 Hz), 131.6 (d, J = 9.9 Hz, 4C, C_{ar}), 130.3 (2C, $C_{q,ar}$), 129.0 (d, J = 0.7 Hz, 2C, C_{ar}), 128.8 (d, J = 12.1 Hz, 4C, C_{ar}), 127.9 (d, J = 1.0 Hz, 2C, C_{ar}), 119.4 (d, J = 104.4 Hz).

ESI-HRMS: *m/z*: $[M + H]^+$ Calcd for $[C_{20}H_{17}OP + H]^+$: 305.1090. Found 305.1086.

The same procedure was applied with 2 mol-% and 5 mol-% of [Cu(^{Cy}cAAC)Cl] (**1c**) and with 10 mol-% of **1d**:

The reaction using **2** mol-% of **1c** yielded a combined yield of 86 % (0.62 g, 2.02 mmol, dr = 1.0 : 1).
The reaction using **5** mol-% of **1c** yielded a combined yield of 88 % (0.62 g, 2.05 mmol, dr = 2.5 : 1).
The reaction using **10** mol-% of **1d** yielded a combined yield of 83 % (0.26 g, 1.04 mmol, dr = 1 : 2.9).

2. X-Ray Structure Determination

Single crystals of **2a-2c** were immersed in a film of NVH oil, mounted onto a polyamide microloop (*MiTiGen*), transferred to a stream of cold nitrogen (*Bruker Kryoflex2/Cryostream 700* cooling system by *Oxford Cryosystems*) and measured at a temperature of 100 K (**2b-d**) or 150 K (**2a**). **2d** was crystallized from a saturated solution in *n*-pentane at -30 °C and single crystals were selected under a cold stream of nitrogen (–100 °C, *X-Temp 2*)[14] in perfluoroalkyl ether oil. Diffraction data were collected on a *Bruker D8 Venture* diffractometer with a *Bruker Photon II* CMOS detector. X-rays were generated by *microfocus IμS* Cu/Mo tubes from *Incoatec GmbH*. Data were collected and processed using APEX 4 Suite (SAINT/SADABS) from *Bruker*. Structure solution was performed with SHELXT.[15] Refinement of the structures was done with OLEX 2 (v1.5) or ShelXle3[16] using SHELXL.[17] All non-hydrogen atoms were refined with anisotropic displacement parameters. The hydrogen atoms were refined isotropically on calculated, idealized positions. Structure finalization was realized with FinalCIF (https://dkratzert.de/finalcif.html).

Table S 1: SC/XRD parameters of **2a** - **2d** and **7a**.

Parameter \ Compound	[Cu(^{Me}CAAC)(PMes$_2$)] **2a**	[Cu(^{Et}CAAC)(PMes$_2$)] **2b**	[Cu(^{Cy}CAAC)(PMes$_2$)] **2c**	[Cu(^{Ment}CAAC)(PMes$_2$)] **2d · *n*-pentane**	Ph(CH)$_2$P(O)Ph$_2$ **7a**
CCDC-Number	2481679	2481670	2481647	2481689	2481691
CIF-ID	PCRX1037-PCRB1037	PCRX1012-PCRB1024	PCRX1021-PCRB1031	PCRX1065-PCRB1043-JJH	PCRX1209-PCRB1051
Sum formula	$C_{38}H_{53}CuNP$	$C_{40}H_{57}CuNP$	$C_{41}H_{57}CuNP$	$C_{46.67}H_{69}CuNP$	$C_{20}H_{17}OP$
M / g mol^{-1}	618.32	646.425	658.436	738.53	304.31
T / K	150(2)	100.00	100.00	100.00	100(2)
Crystal system	monoclinic	monoclinic	monoclinic	monoclinic	Monoclinic
Space group (#)	$P2_1/c$ (14)	$P2_1/c$ (14)	$P2_1/c$ (14)	$P2_1$ (4)	$P2_1/n$ (14)
a / Å	16.6746(7)	12.6056(6)	17.5516(9)	12.2041(4)	16.4590(7)
b / Å	13.1698(5)	17.6043(8)	13.3084(6)	20.6661(7)	5.8049(3)
c / Å	16.0654(6)	17.3103(9)	15.9247(7)	27.1497(8)	33.8444(13)
α / °	90	90	90	90	90
β / °	93.351(2)	111.236(2)	90.881(2)	101.3170(10)	99.693(3)
γ / °	90	90	90	90	90
Volume V / Å^3	3521.9(2)	3580.5(3)	3719.3(3)	6714.3(4)	3187.4(2)
Z	4	4	4	6	4
ρ / g cm^{-3}	1.166	1.199	1.176	1.096	1.268
μ / mm^{-1}	1.473	0.682	0.658	1.230	1.504
F(000)	1328	1394.472	1418.486	2400	1280
Crystal size / mm^3	0.178 × 0.113 × 0.056	0.261×0.164×0.088	0.215×0.112×0.056	0.106×0.047×0.023	0.075×0.02×0.07
Color	reddish orange	yellow	clear orange	yellow	Colorless
Shape	block	plate	block	block	Needle
Radiation (λ / Å)	Cu K$_\alpha$ (1.54178)	Mo K_α (0.71073)	Mo K_α (0.71073)	Cu K_α (1.54178)	Cu K_α (1.54178)
2θ / ° (Resolution / Å)	5.31 – 133.85 (0.84)	4.16 – 67.00 (0.64)	3.84 – 60.08 (0.71)	5.41 – 158.76 (0.78)	5.30 – 132.99 (0.84)
Reflections collected	59778	284843	88254	185395	32485
Independent reflections	6243 (R_{int} = 0.0834, R_{sigma} = 0.0387)	14041 (R_{int} = 0.1169) R_{sigma} = 0.0423)	10851 (R_{int} = 0.0732) R_{sigma} = 0.0560)	28280 (R_{int} = 0.0404) R_{sigma} = 0.0252)	5432 (R_{int} = 0.1665) R_{sigma} = 0.1088
Parameters	384	402	409	1519	397
Completeness	99.6 %	99.9 %	99.6 %	99.9 %	97.1 %
Absorption correction	Multi-Scan	Multi-Scan	Multi-Scan	Multi-Scan	Multi-Scan
T_{min} / T_{max}	0.6148 / 0.7528	0.7052 / 0.7473	0.6261 / 0.7461	0.674 / 0.754	0.5922 / 0.7533
GooF	1.070	1.0303	1.0589	1.017	1.024
R_1 (wR_2) für [I≥2σ(I)]	0.0501 (0.1287)	0.0432 (0.0992)	0.0477 (0.0951)	0.0303 (0.0806)	0.0794 (0.1754)
R_1 (wR_2) (all data)	0.0683 (0.1414)	0.0647 (0.1103)	0.0710 (0.1049)	0.0319 (0.0819)	0.1214 (0.1971)
Largest peak/hole / e Å^{-3}	0.66 / −0.74	0.60 / −0.93	0.60 / –0.79	0.40 / –0.42	0.53 / –0.57
Flack X parameter		N/A[a]		0.022(10)[b]	N/A[a]

[a] not applicable [b] after twin refinement indicating *ee* = 99 %

Structure of 2a in the solid state

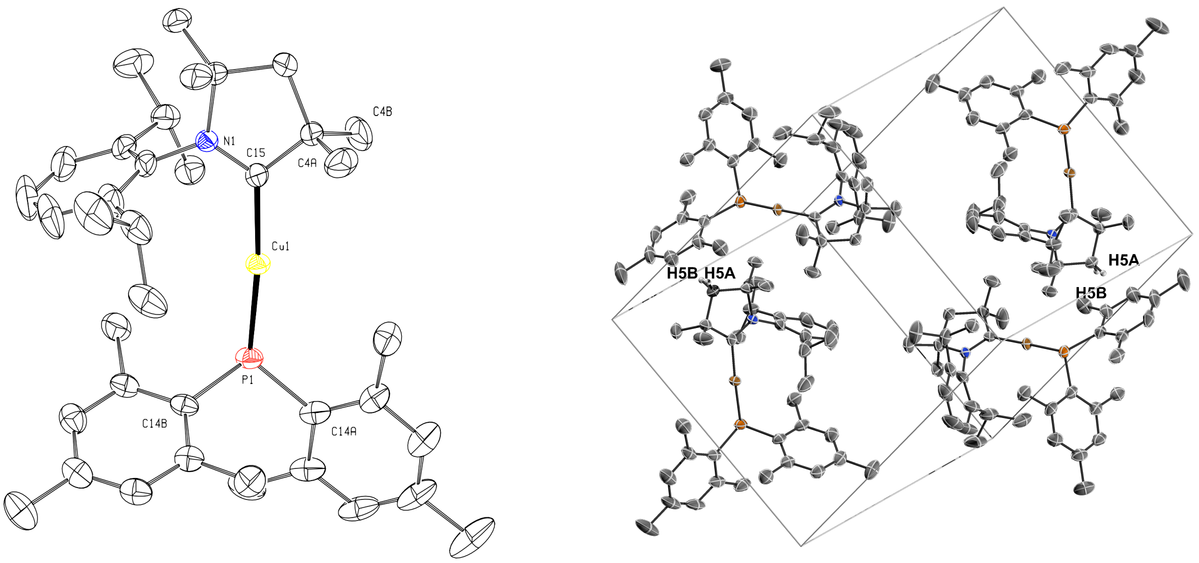

Figure S 26: Molecular structure of **2a** in crystalline state (ORTEP plot, left) and unit cell of the crystal of **2a** (right).

2a: (CCDC: 2481679) In total 16 sweeps with a detector distance of 37 mm and exposure times from 10s/° for low order ($2\Theta = 20°$) up to 20s/° for high order ($2\Theta = 106°$) were collected with an increment of 2° per image. The ORTEP/PLATON plots with 50% probability and relevant atom naming as well as the elementary cell are depicted above.

Structure of 2b in solid state

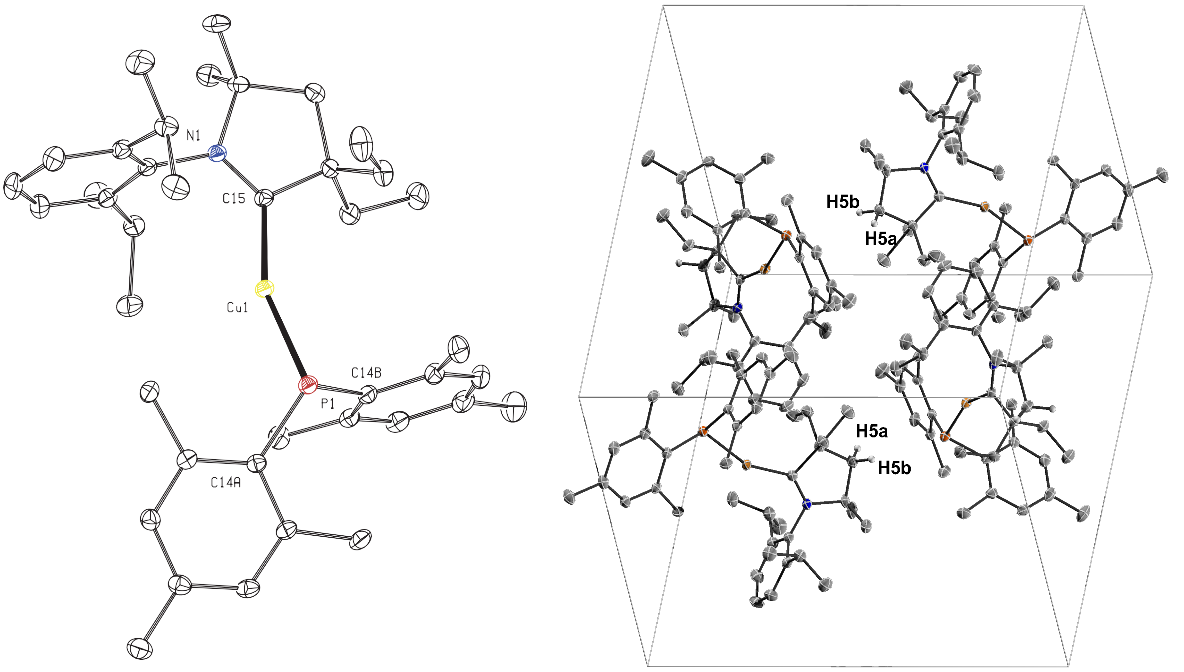

Figure S 27: Molecular structure of **2b** in crystalline state (ORTEP plot, left) and unit cell of the crystal of **2b** (right).

2b: (CCDC: 2481670) In total 16 sweeps with a detector distance of 37 mm and exposure times from 2s/° for low order ($2\Theta = 0°$) up to 18s/° for high order ($2\Theta = 44°$) were collected with an increment of 0.5° per image. The ORTEP/PLATON plots with 50% probability and relevant atom naming as well as the elementary cell are depicted above.

Structure of 2c in solid state

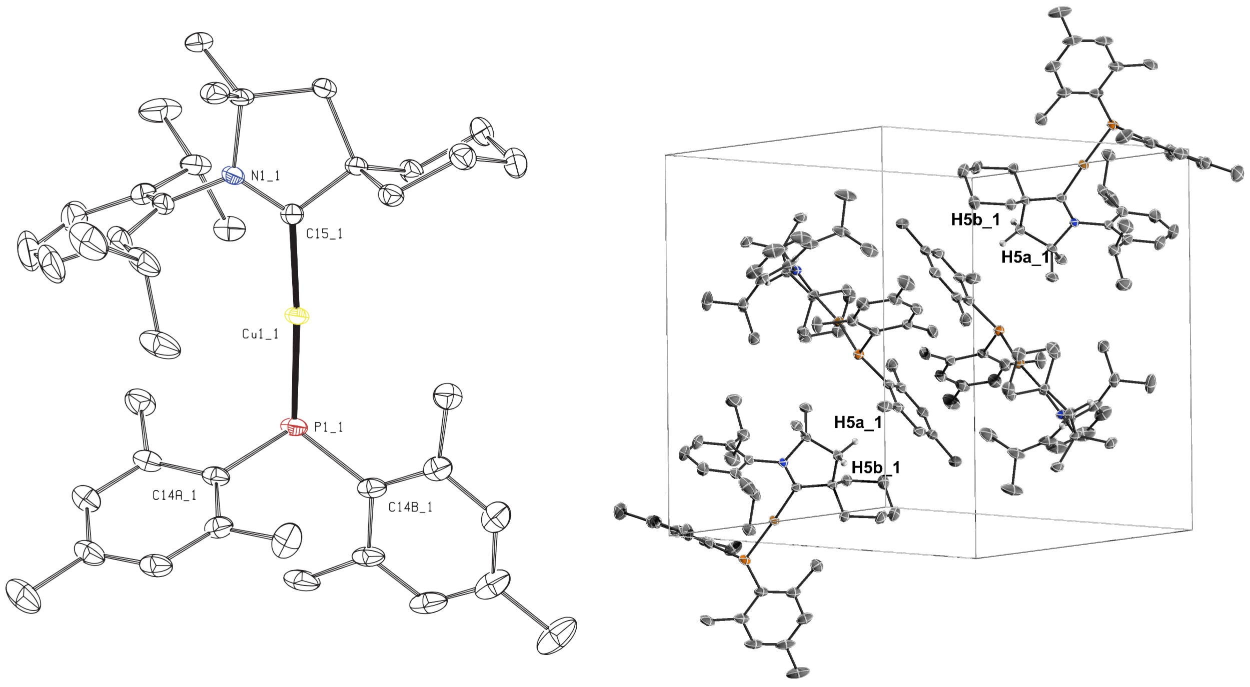

Figure S 28: Molecular structure of **2c** in crystalline state (ORTEP plot, left) and unit cell of the crystal of **2c** (right).

2c: (CCDC: 2481647) In total 16 sweeps with a detector distance of 37 mm and exposure times from 1s/° for low order ($2\Theta = 0°$) up to 40s/° for high order ($2\Theta = 9°$) were collected with an increment of 0.5° per image. The ORTEP/PLATON plots with 50% probability and relevant atom naming as well as the elementary cell are depicted above.

Structure of 2d in solid state

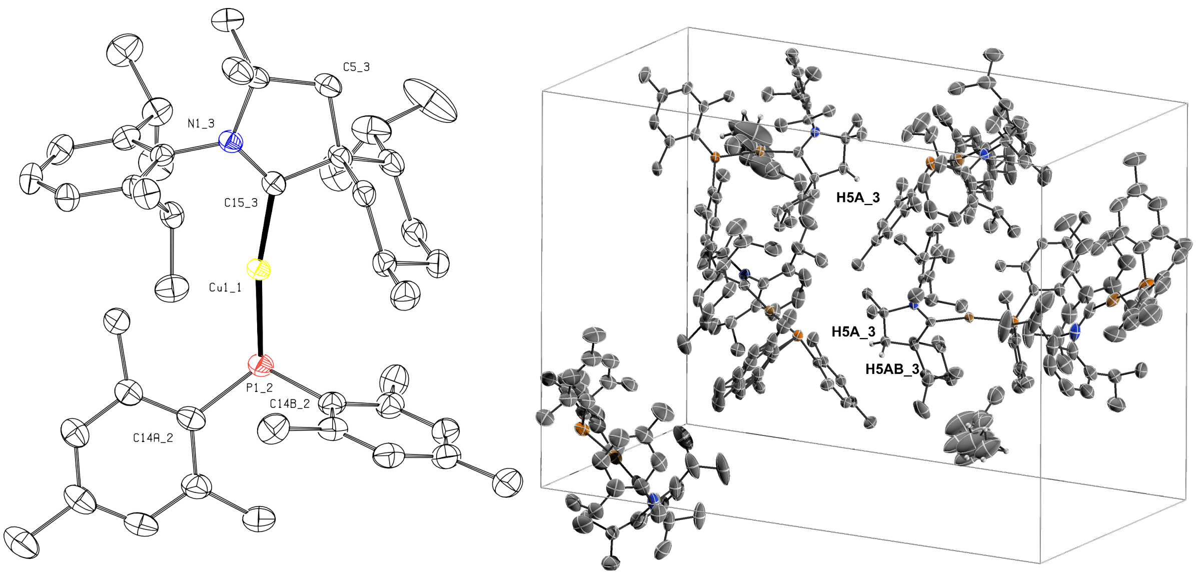

Figure S 29: Molecular structure of **2d** in crystalline state (ORTEP plot, left) and unit cell of the crystal of **2d** (right).

2d · C_5H_{12}: (CCDC: 2481689) The ORTEP/PLATON plots with 50% probability and relevant atom naming as well as the elementary cell are depicted above. To avoid collapse of the crystal lattice, the crystal was quickly mounted onto a 50 µm *Mitegen* dual thickness *MicroLoop LD*™. In total 21 sweeps with a detector distance of 45 mm and exposure times from 4s/° for low order ($2\Theta = 0°$) up to 140s/° for high order ($2\Theta = 104°$) were collected with an increment of 0.5° per image. All data were integrated with SAINT and a multi-scan absorption correction using SADABS was applied. The structure was solved by intrinsic phasing/direct methods using SHELXT[15] and refined with SHELXL[17] using full-matrix least-squares routines on F^2 and ShelXle[16] as a graphical user interface and the DSR program plugin was employed for modeling.[18]

The asymmetric unit contains three target molecules and one pentane solvent molecule. Disorder of one pentane solvent molecule and one 1,3,5-trimethylphenyl group were modeled using stereochemical restraints generated by the GRADE program using the GRADE Web Server (http://grade.globalphasing.org) and applied in the refinement. A GRADE dictionary for SHELXL contains target values and standard deviations for 1,2-distances (DFIX) and 1,3-distances (DANG), as well as restraints for planar groups (FLAT). All displacements for non-hydrogen atoms were refined anisotropically. The refinement of ADP's for carbon, nitrogen and oxygen atoms was enabled by a combination of similarity restraints (SIMU) and rigid bond restraints (RIGU).[19] The contribution of the electron density from disordered counterions and solvent molecules, which could not be modeled with discrete atomic positions, were handled using the SQUEEZE[20] routine in PLATON.[21] The solvent

mask file (.fab) computed by PLATON was included in the SHELXL refinement via the ABIN instruction leaving the measured intensities untouched.

We could only model one disordered pentane molecule with discrete atomic positions in the asymmetric unit, which results in a 3:1 ration (target molecule:pentane). However, the total squeezed volume of the unit cell is 478 Å³ (7%). With an estimate of 23 Å³ per non-hydrogen atom this volume perfectly corresponds to the volume of four pentane molecules (each 115 Å³) in the unit cell (two pentane molecules in the asymmetric unit). This is plausible and would perfectly fit the NMR findings of a 1:1 ratio (target molecule:pentane).

The *Flack x* was determined to be 0.018(3) using 12340 quotients [(*I*+)-(*I*-)]/[(*I*+)+(*I*-)] after method from *Parsons et al.*[22] The Flack parameter value deviates significantly from zero as estimated from the ratio of that value to the associated s.u. This indicates a borderline case of inversion twining, which is also indicated by CHACKCIF 987_ALERT_1_B Alert. We therefore refined inversion twinning using BASF/TWIN construct in SHELXL, yielding a refined BASF parameter of 0.022(10), which is reported as "Flack x" item in the CIF by SHELXL. This result corresponds well with the 99% *ee* of the chiral starting material used for synthesis. The squeezed organic solvent only contains light atoms, so that its contribution to the anomalous signal is expected to be low.

Structure of 5a in solid state

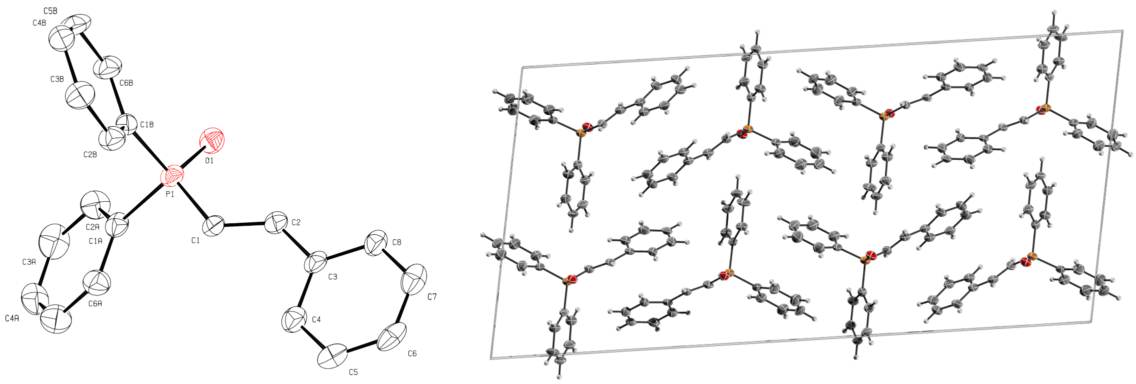

Figure S 30: Molecular structure of **7a** in crystalline state (ORTEP plot, left) and unit cell of the crystal of **7a** (right).

7a: (CCDC: 2481691) In total 7 sweeps with a detector distance of 37 mm and exposure times from 10s/° for low order ($2\Theta = 0°$) up to 96s/° for high order ($2\Theta = 106°$) were collected with an increment of 0.5° per image. The ORTEP/PLATON plots with 50% probability and relevant atom naming as well as the elementary cell are depicted above.

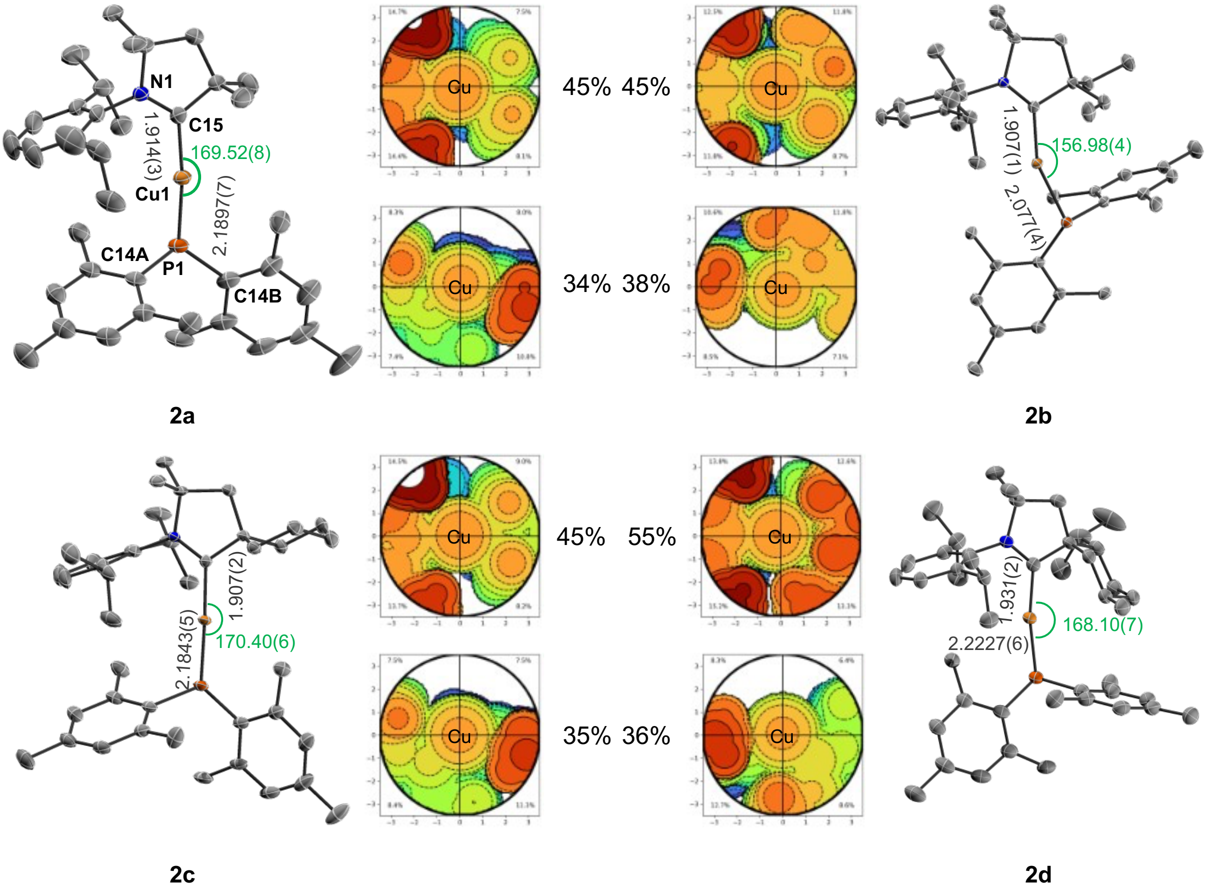

Figure S 31: Graphical representation of the buried volume (V_{bur} in %) of complexes **2a** – **2d** and respective molecular structures in solid state with important bond lengths and angles**.**

3. 1- and 2-D NMR Spectra

NMR spectra of $H(O)PMes_2$ and $HPMes_2$

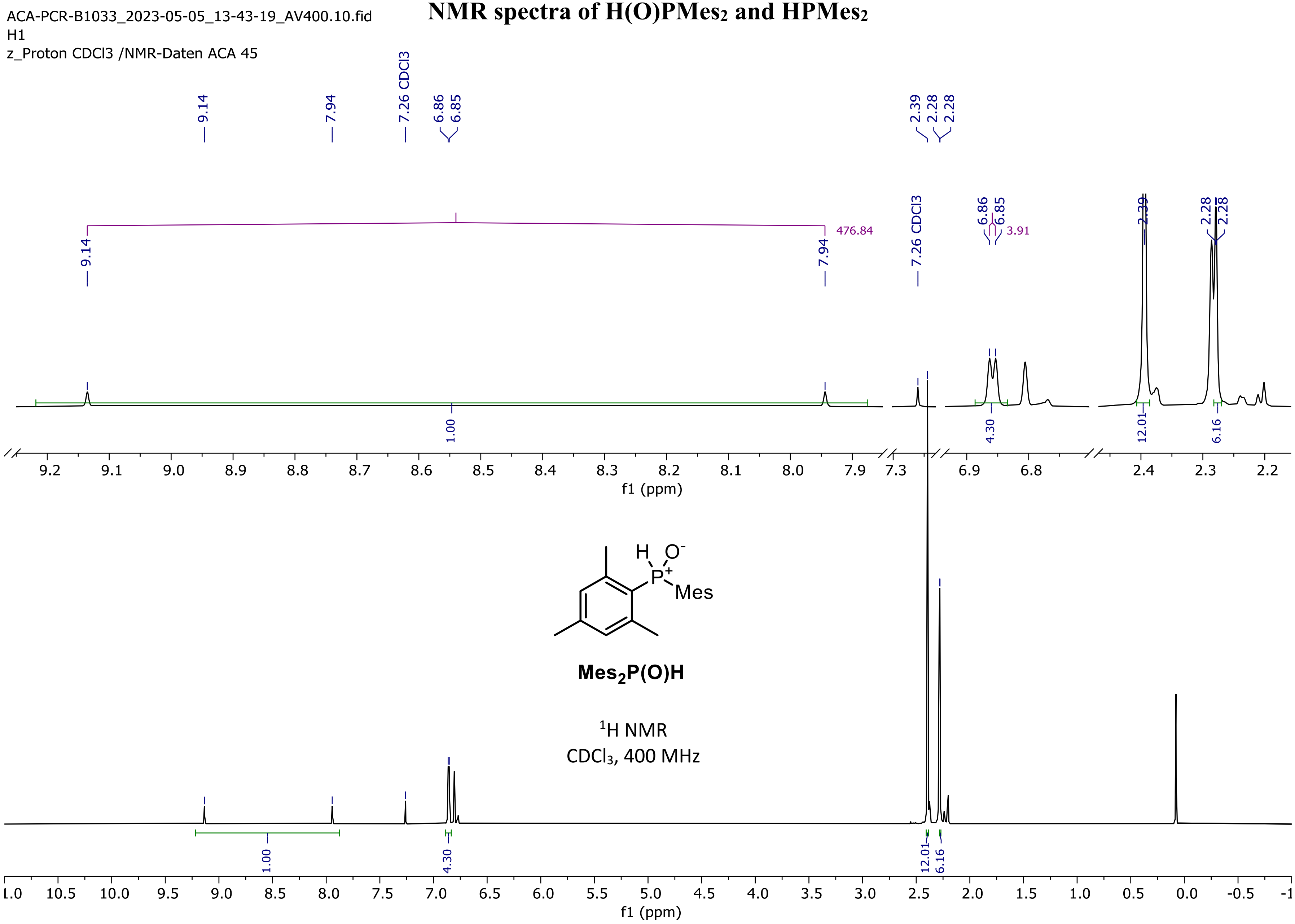

Figure S 32: ^{1}H NMR spectrum of dimesitylphosphine oxide (400 MHz, $CDCl_3$, 298 K).

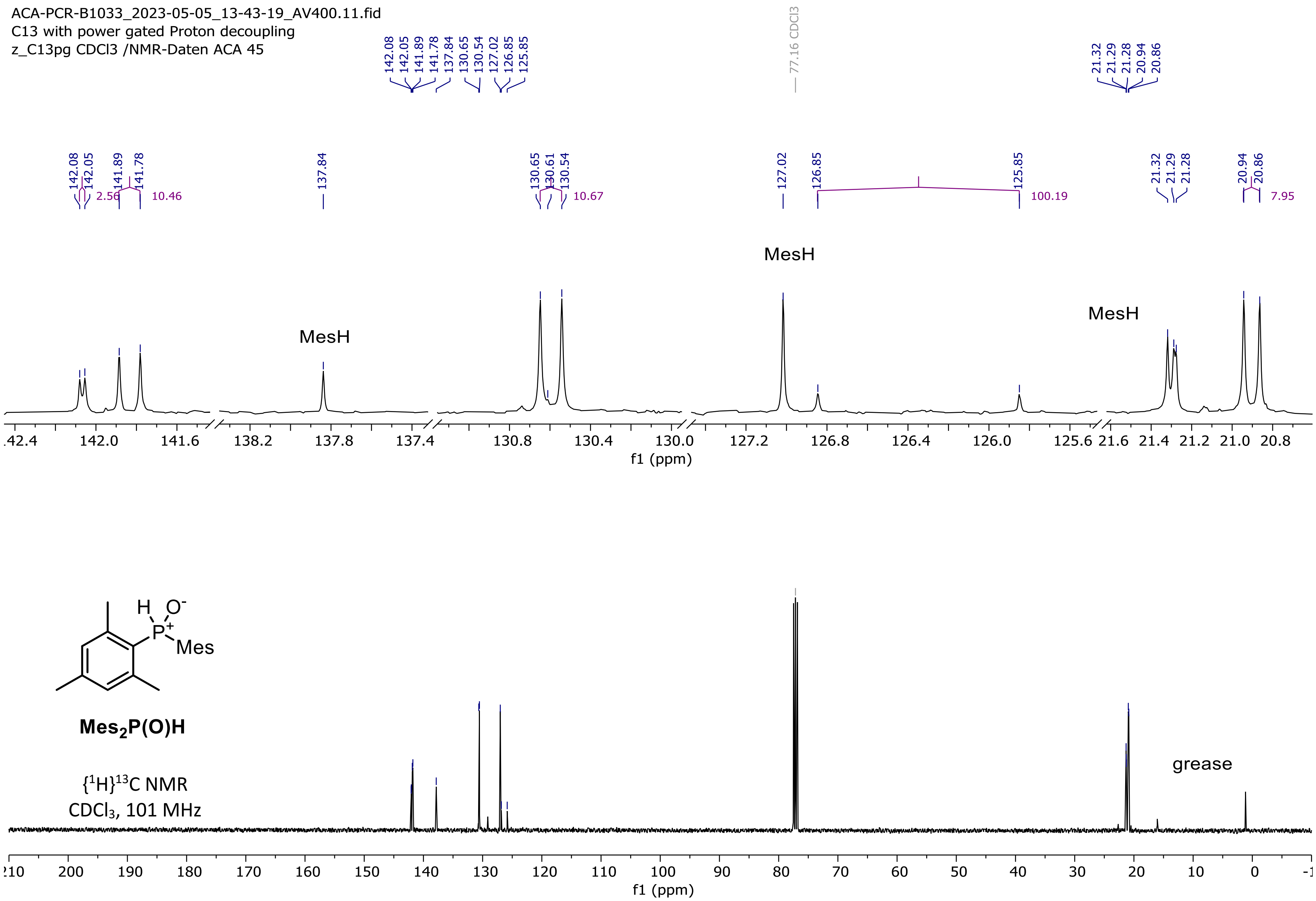

Figure S 33: $\{^1H\}^{13}C$ NMR spectrum of dimesitylphosphine oxide (400 MHz, $CDCl_3$, 298 K).

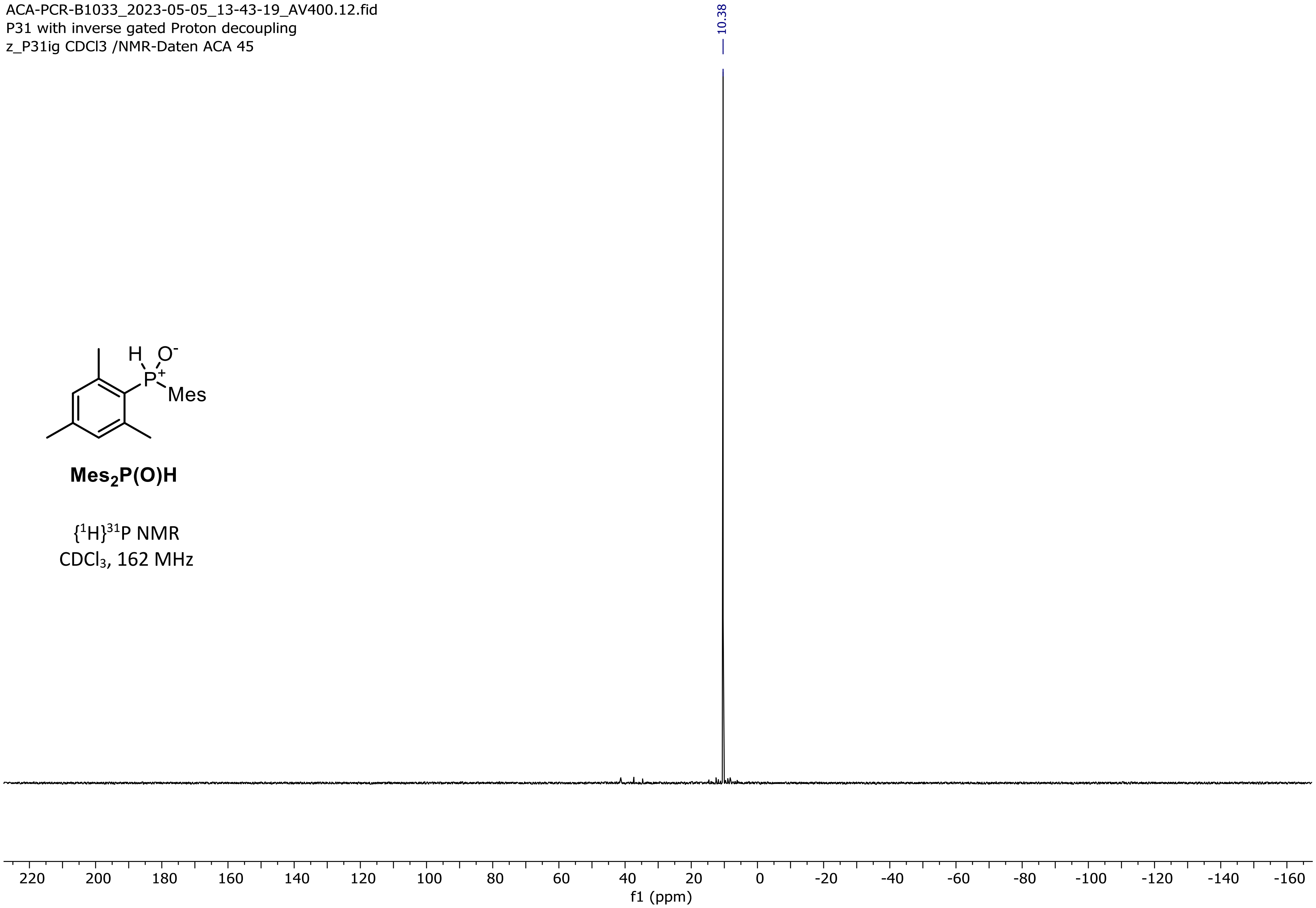

Figure S 34: $\{^1H\}^{31}P$ NMR spectrum of dimesitylphosphine oxide (162 MHz, $CDCl_3$, 298 K).

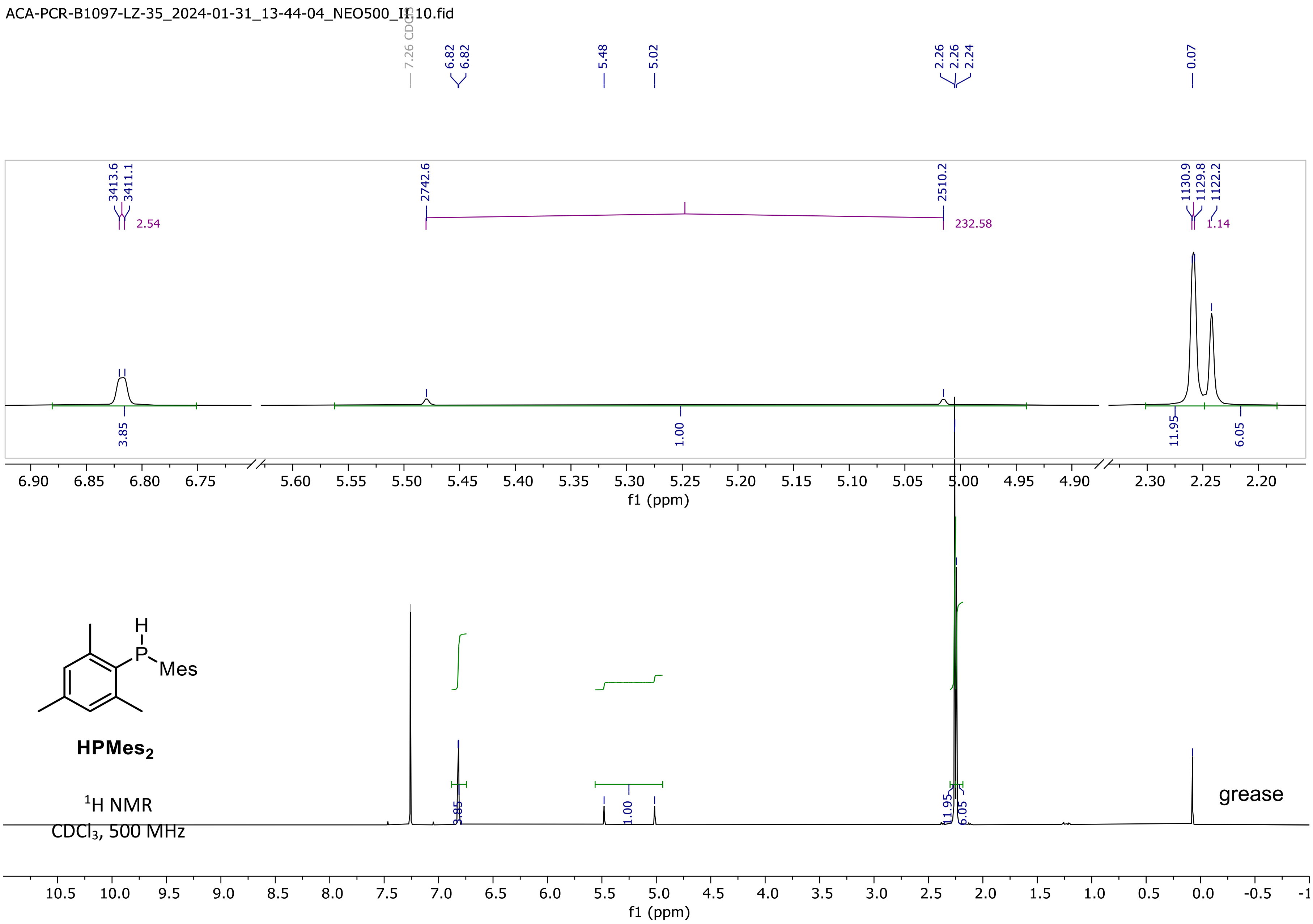

Figure S 35: ^{1}H NMR spectrum of dimesitylphosphine (500 MHz, $CDCl_3$, 298 K).

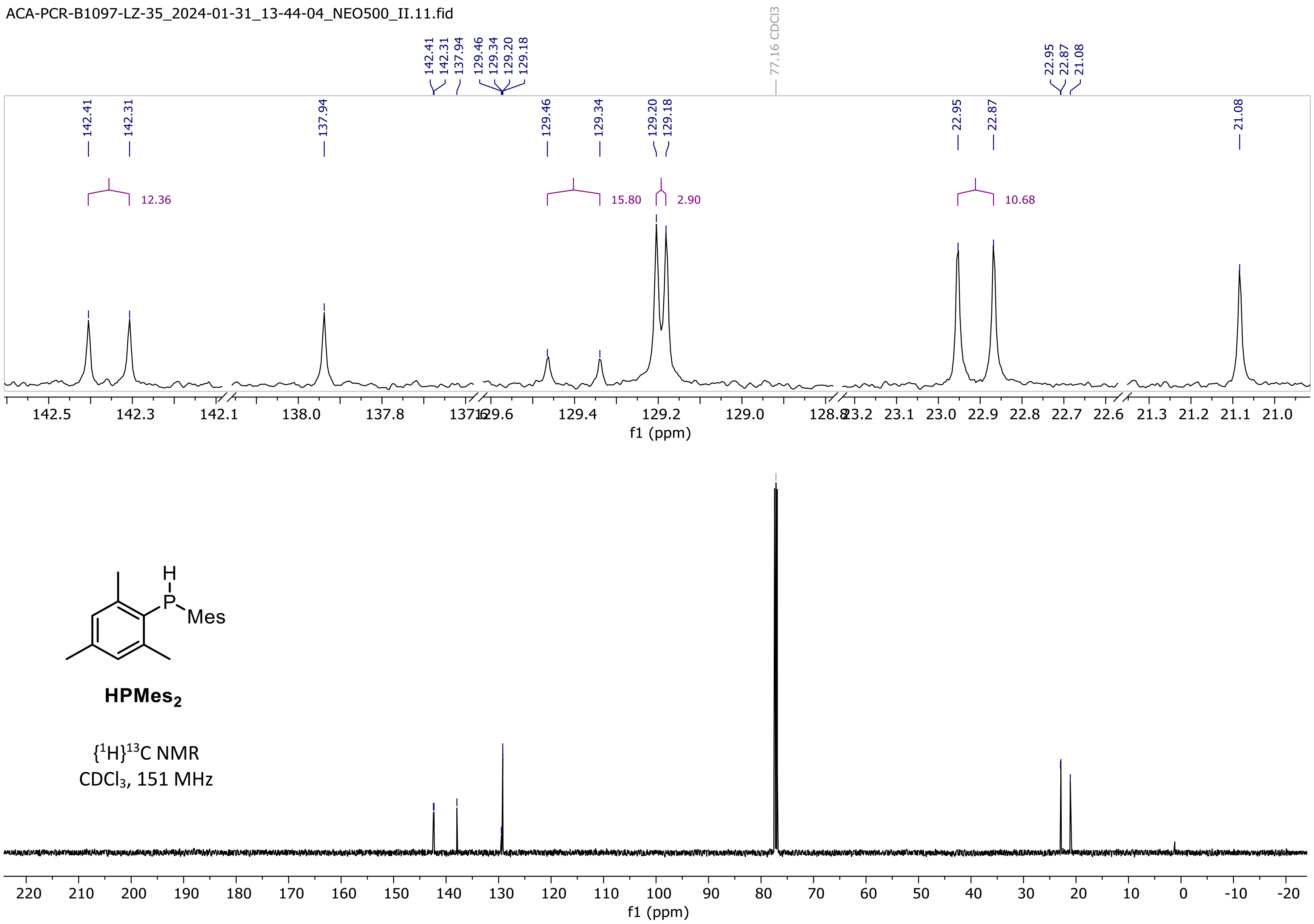

Figure S 36: {^{1}H}^{13}C NMR spectrum of dimesitylphosphine (151 MHz, $CDCl_3$, 298 K).

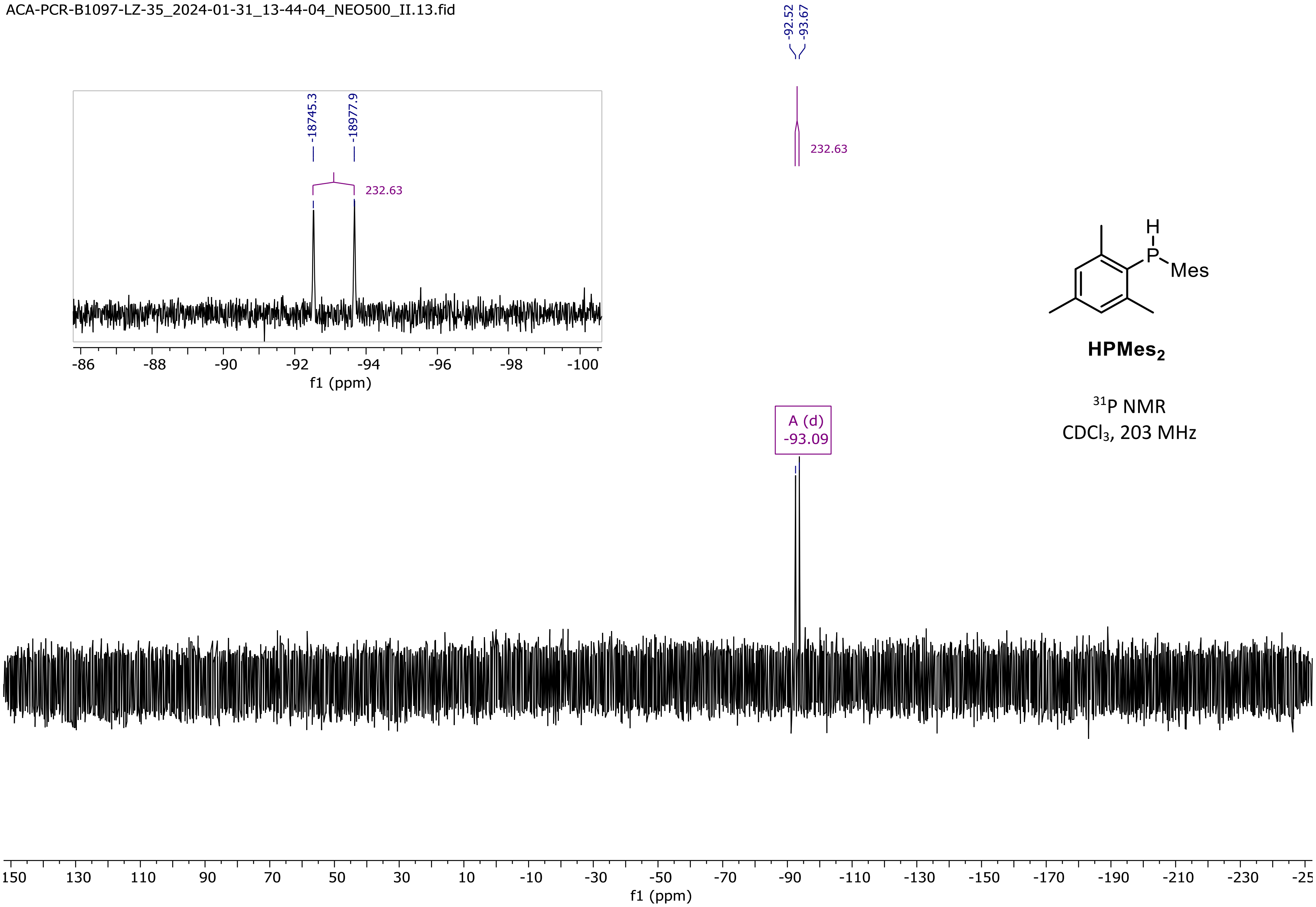

Figure S 37: ^{31}P NMR spectrum of dimesitylphosphine (203 MHz, $CDCl_3$, 298 K).

NMR spectra of 2a [Cu(^{Me}cAAC)($PMes_2$)]

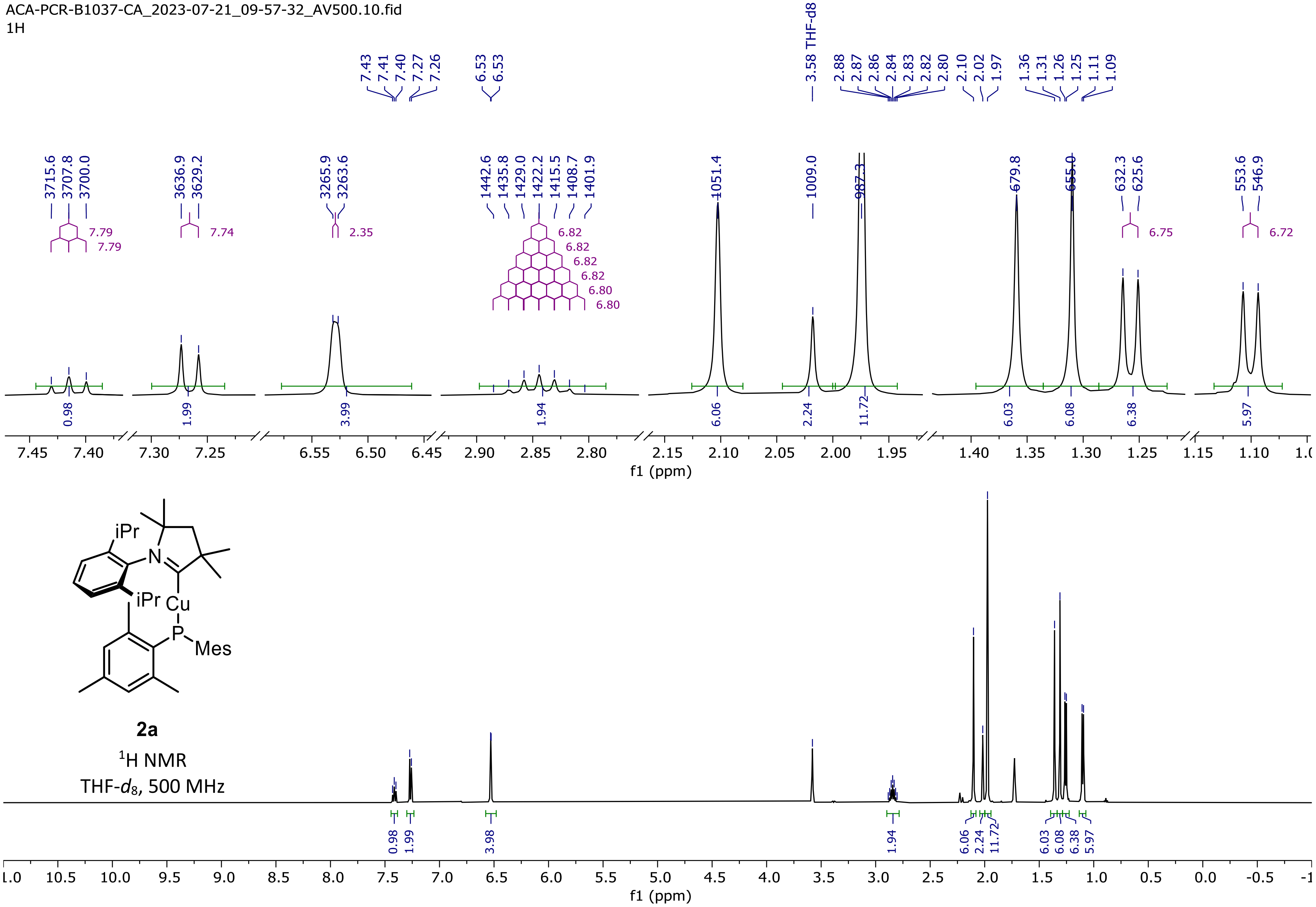

Figure S 38: ^{1}H NMR spectrum of [Cu(^{Me}cAAC)($PMes_2$)] (**2a**) (500 MHz, THF-d_8, 298 K).

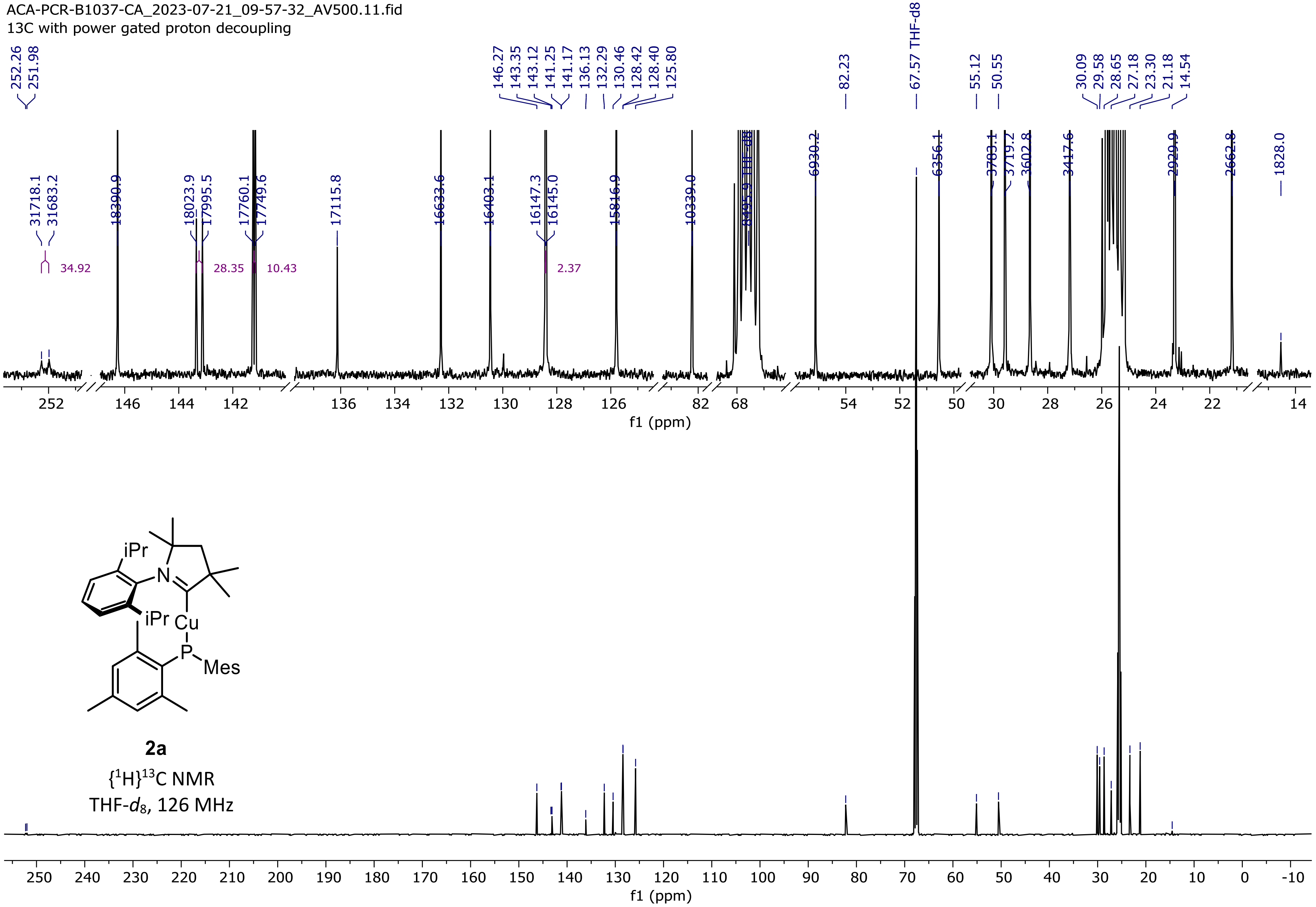

Figure S 39: {^{1}H}^{13}C NMR spectrum of [Cu(^{Me}cAAC)($PMes_2$)] (**2a**) (126 MHz, THF-d_8, 298 K).

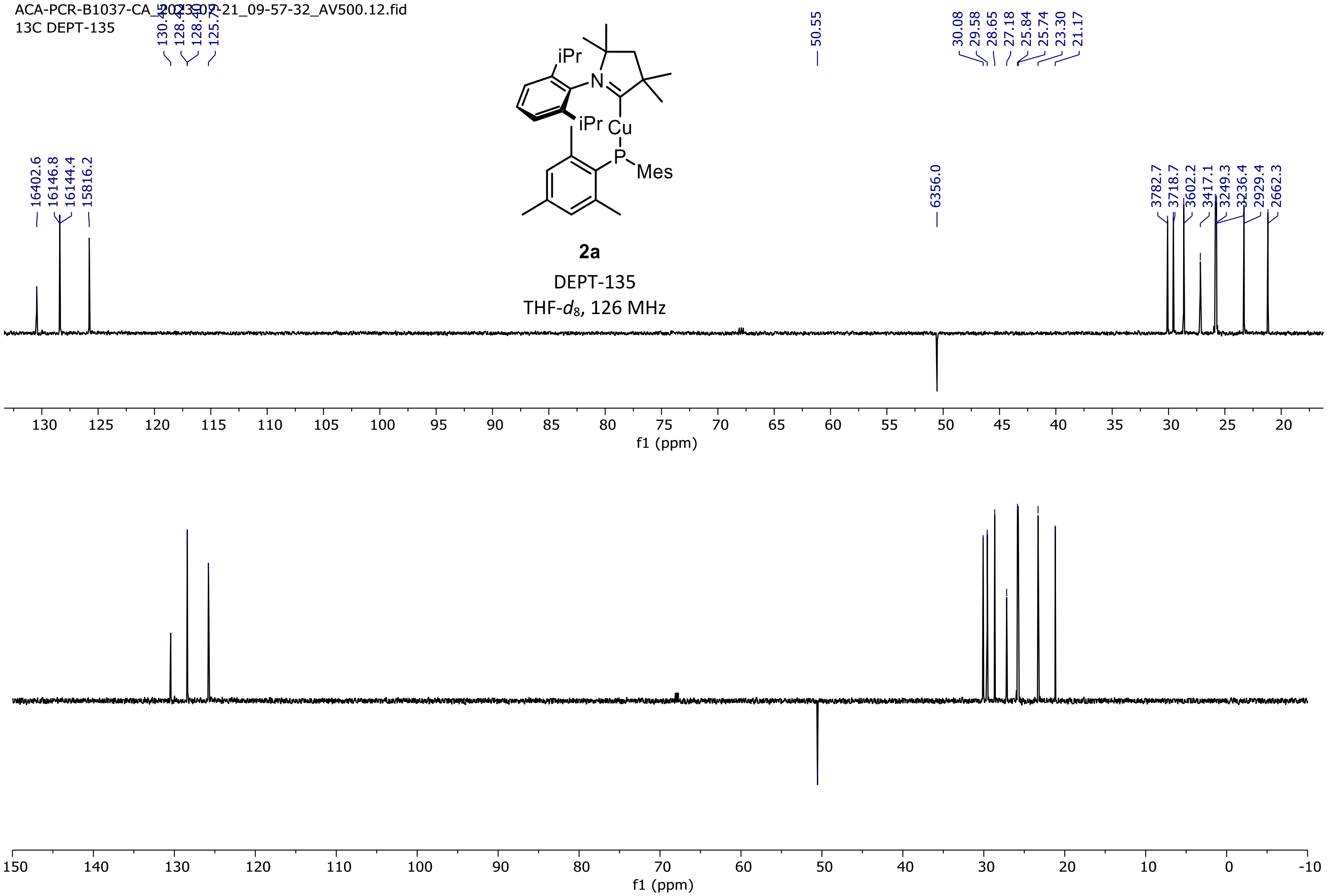

Figure S 40: DEPT-135 NMR spectrum of [Cu(^{Me}cAAC)(PMes$_2$)] (**2a**) (126 MHz, THF-d_8, 298 K).

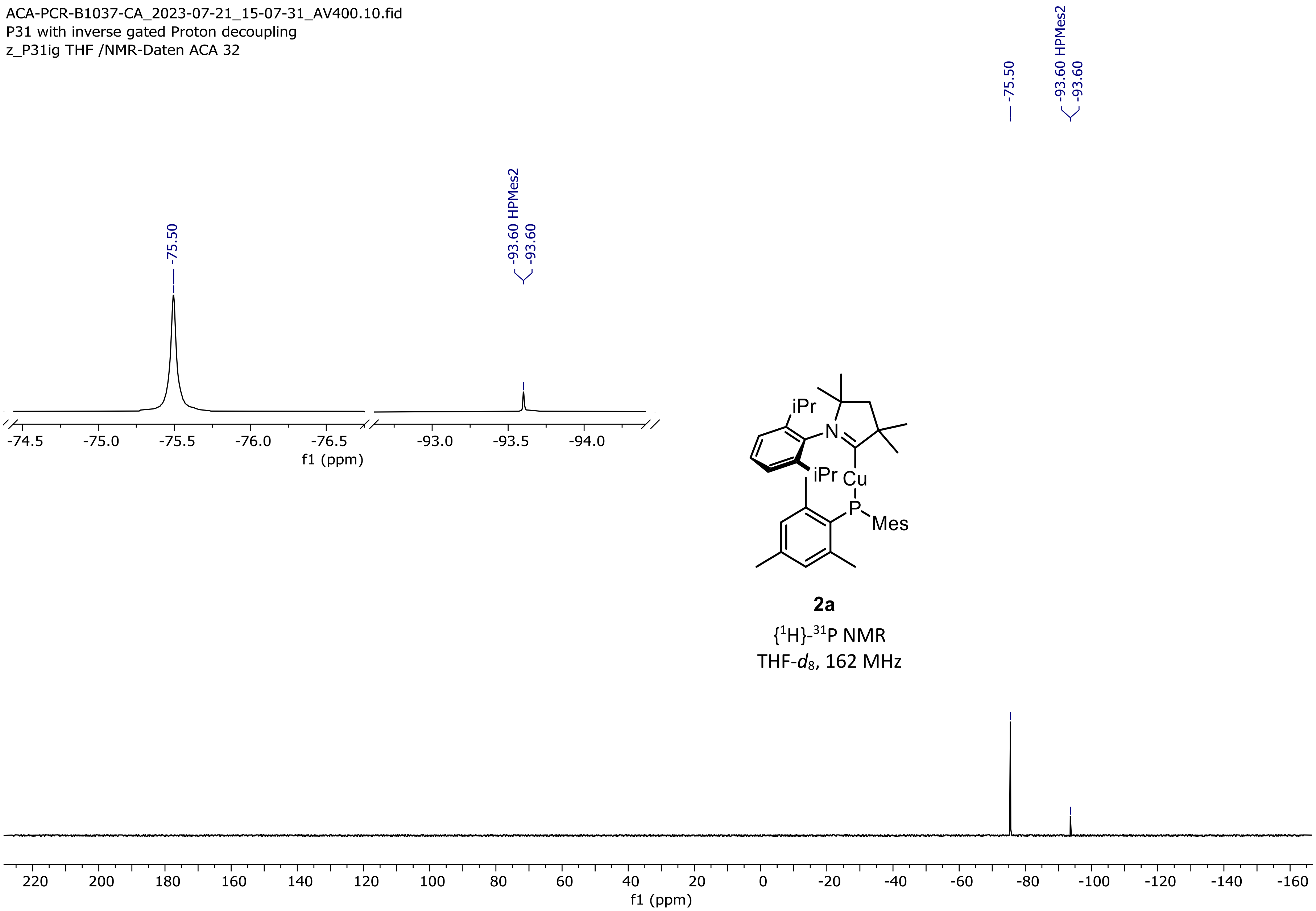

Figure S 41: $\{^1H\}^{31}P$ NMR spectrum of [Cu(^{Me}cAAC)($PMes_2$)] (**2a**) (162 MHz, THF-d_8, 298 K).

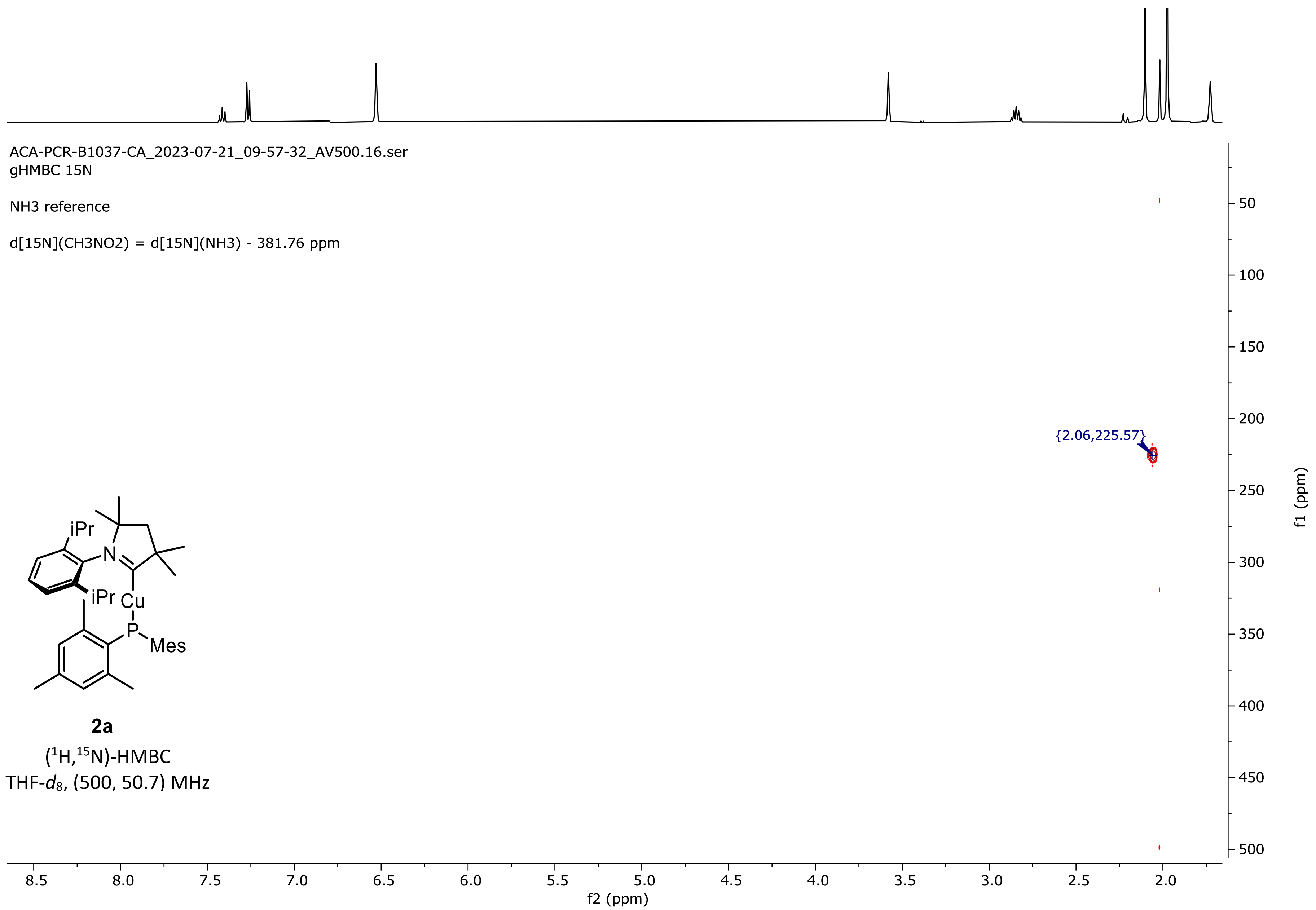

Figure S 42: (^{1}H,^{15}N)-HMBC spectrum of [Cu(^{Me}cAAC)(PMes$_2$)] (**2a**) (500 MHz, 51 MHz, THF-d_8, 298 K).

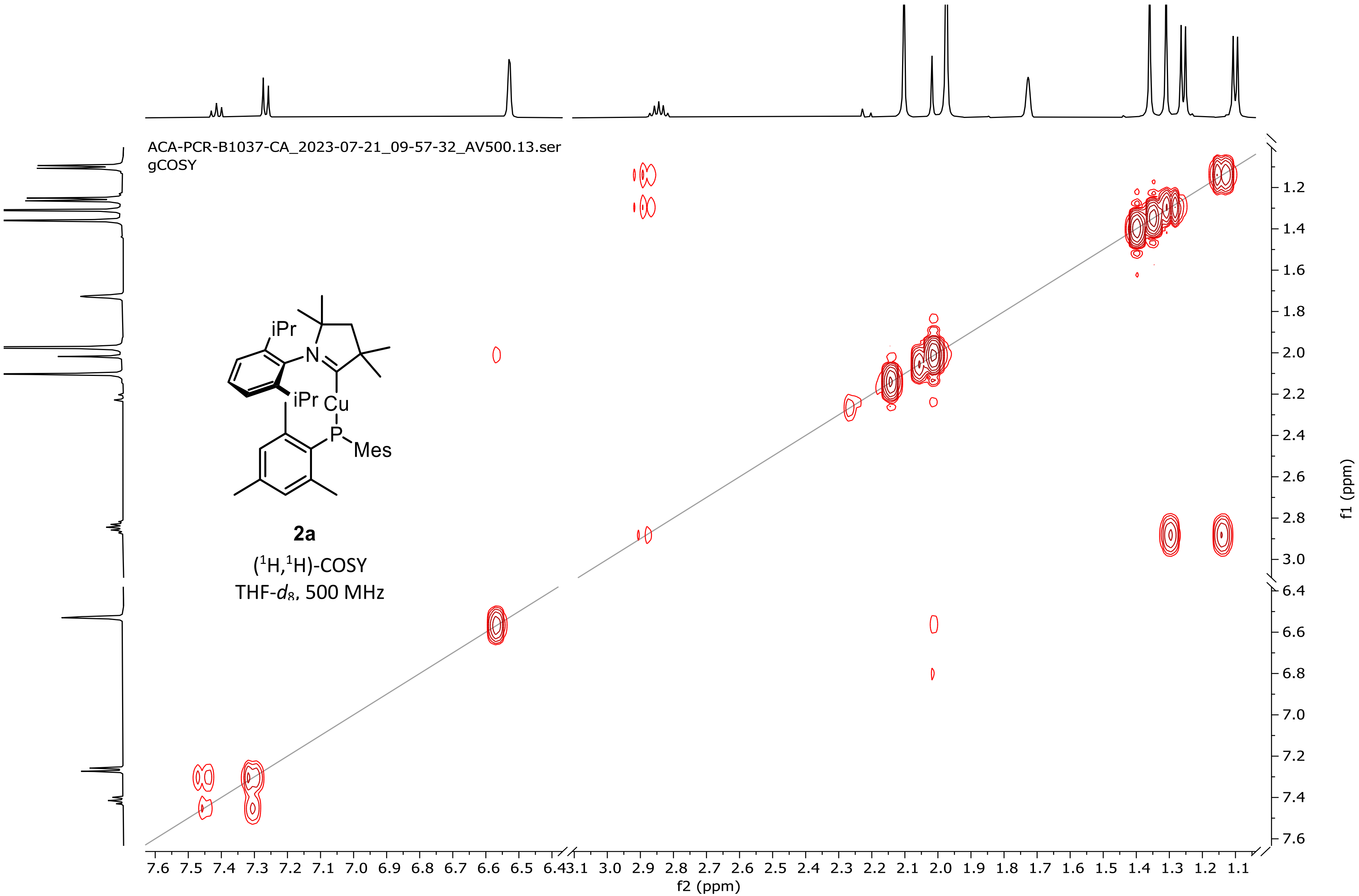

Figure S 43: (^{1}H,^{1}H)-COSY spectrum of [Cu(^{Me}cAAC)(PMes$_2$)] (**2a**) (500 MHz, THF-d_8, 298 K).

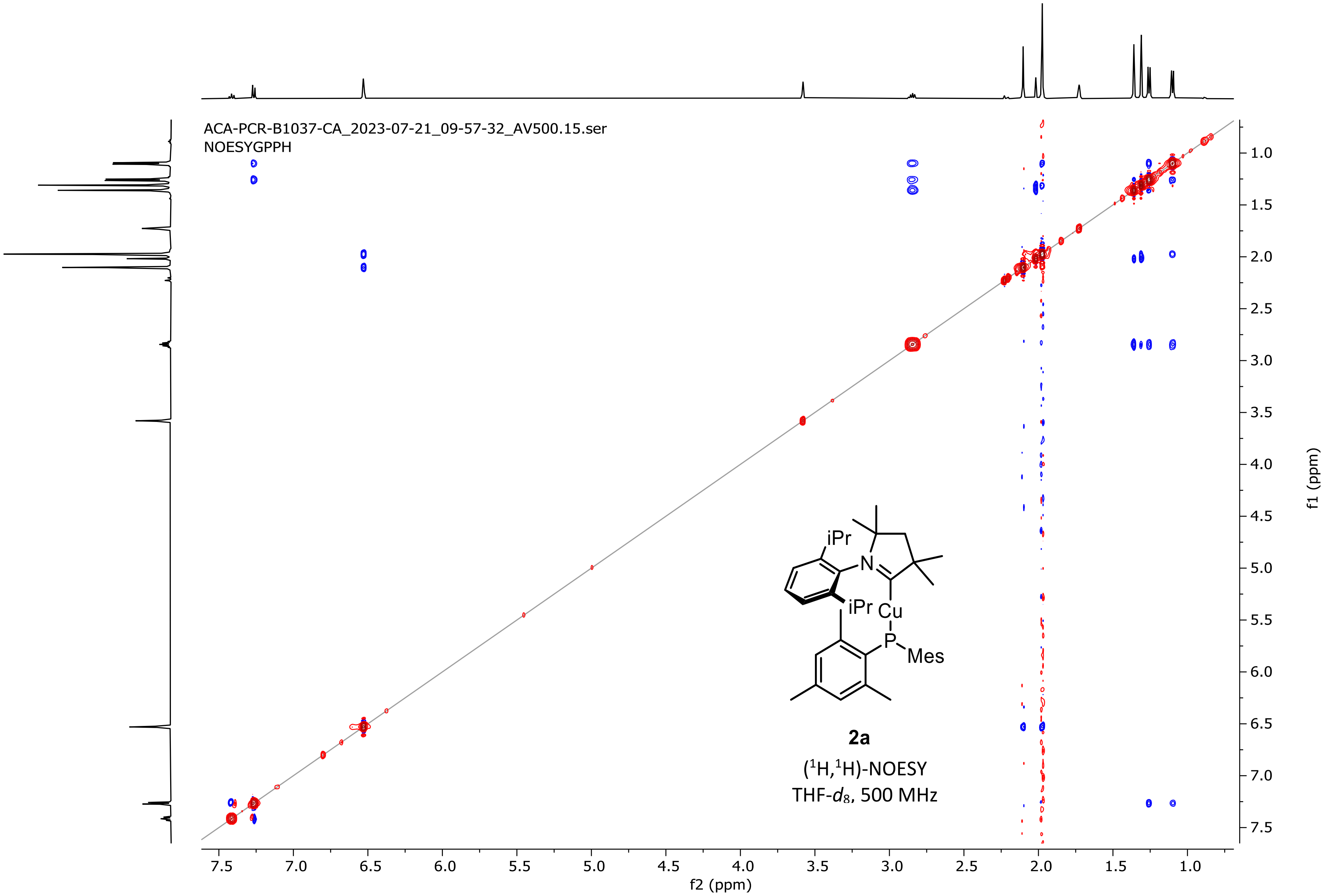

Figure S 44: (^{1}H,^{1}H)-NOESY spectrum of [Cu(^{Me}cAAC)(PMes$_2$)] (**2a**) (500 MHz, THF-d_8, 298 K).

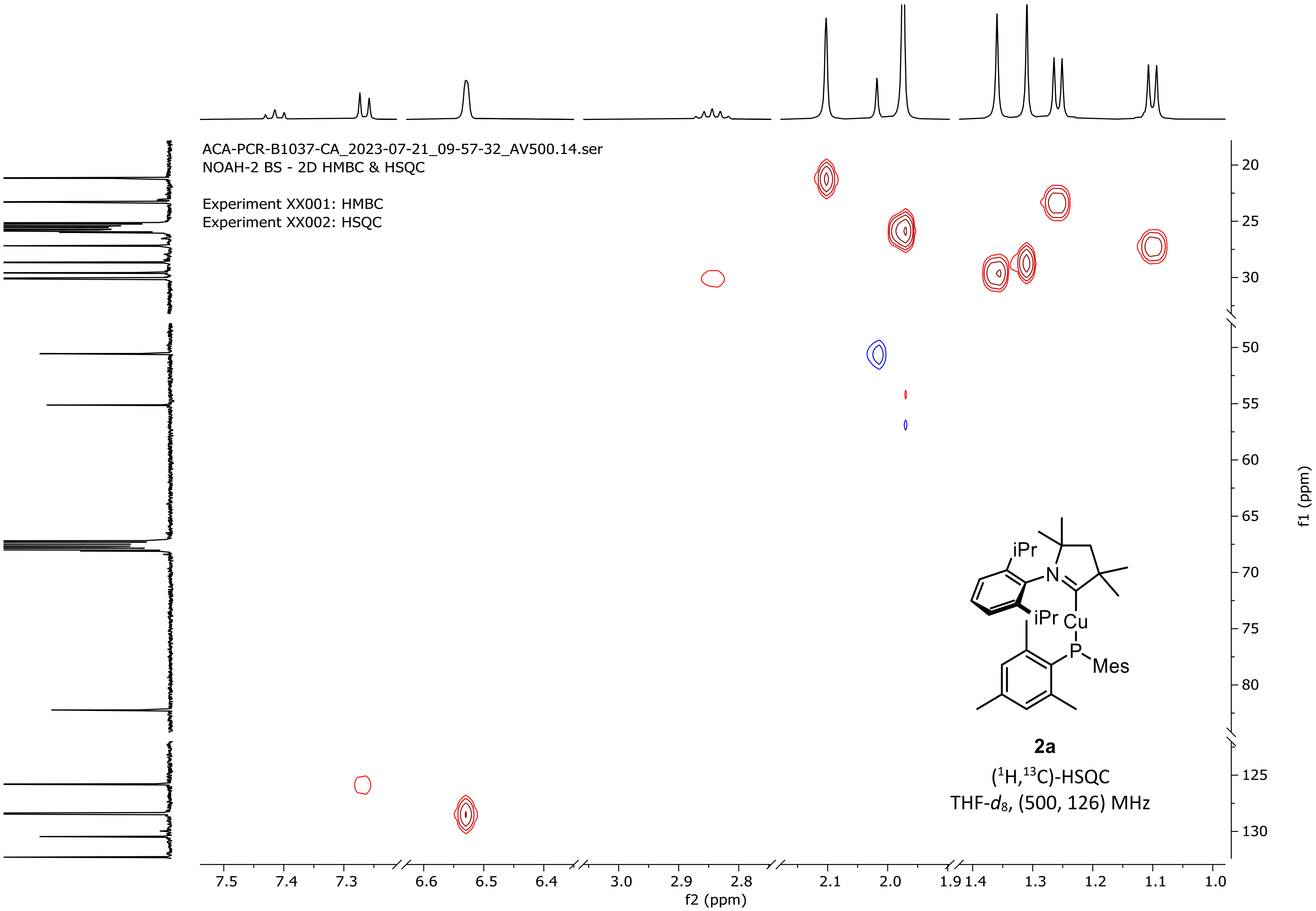

Figure S 45: (^{1}H,^{13}C)-HSQC spectrum of [Cu(^{Me}cAAC)(PMes$_2$)] (**2a**) (500 MHz, 126 MHz, THF-d_8, 298 K).

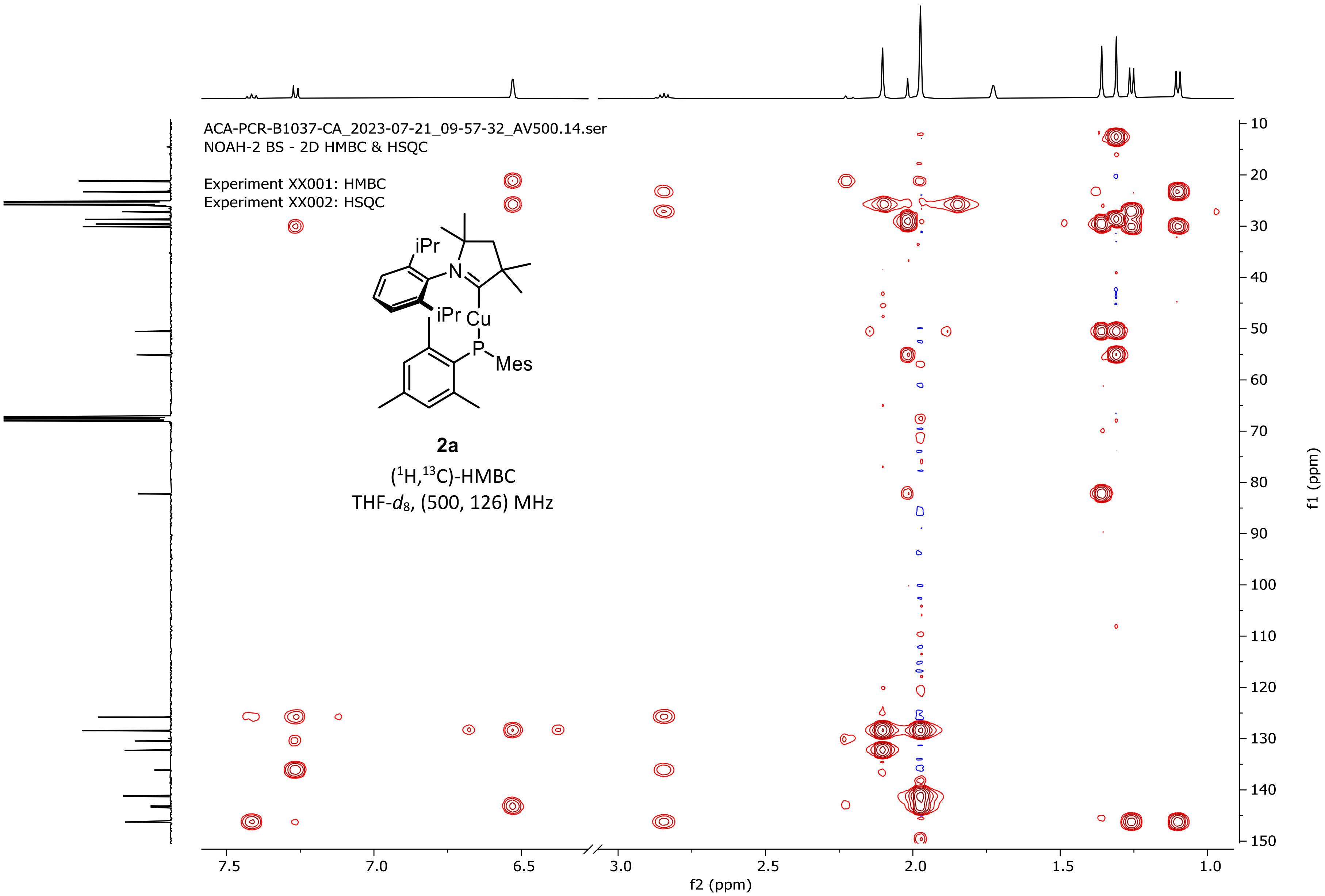

Figure S 46: (^{1}H,^{13}C)-HMBC spectrum of [Cu(^{Me}cAAC)(PMes$_2$)] (**2a**) (500 MHz, 126 MHz, THF-d_8, 298 K).

NMR spectra of 2b [Cu(^{Et}cAAC)(PMes$_2$)]

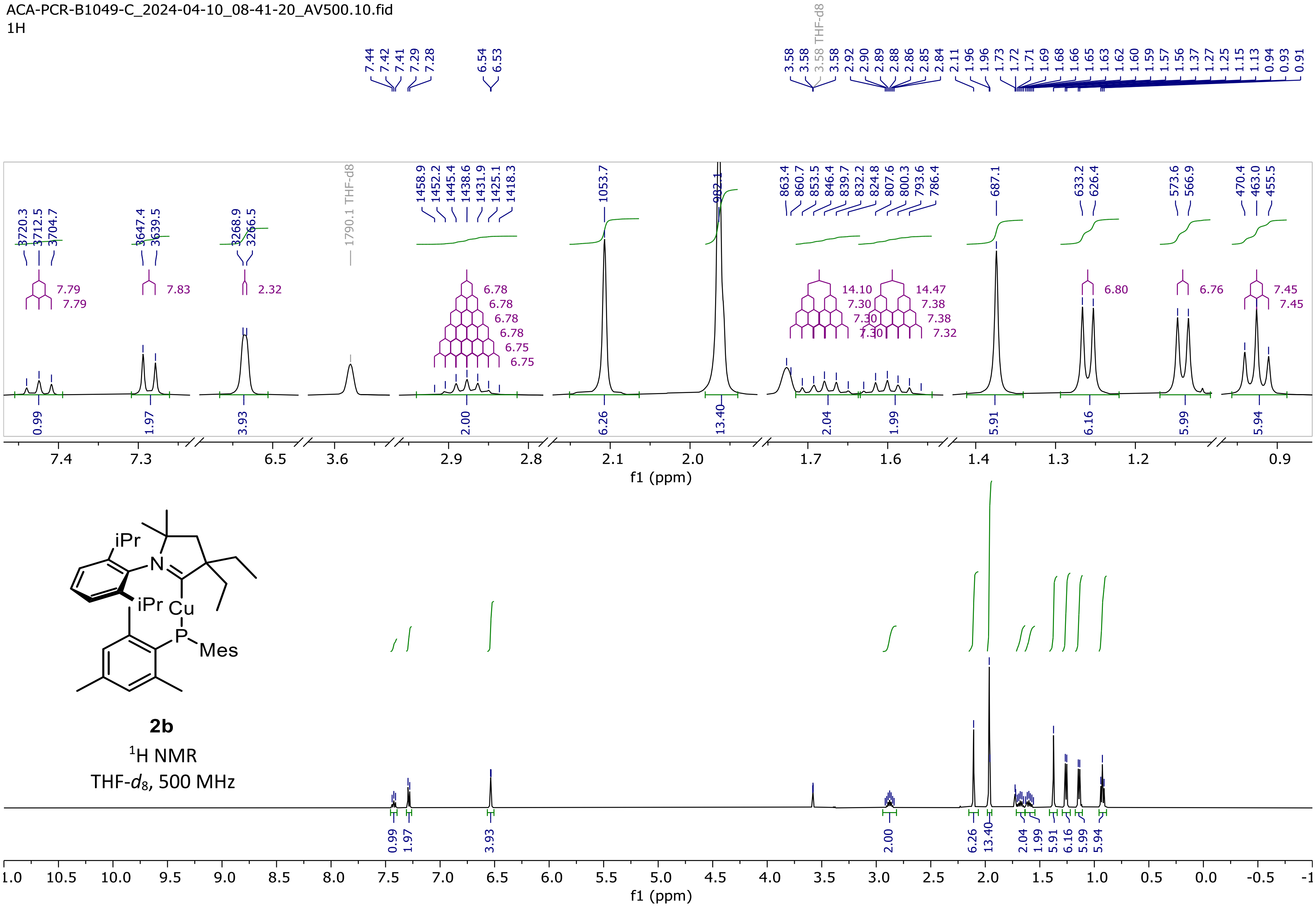

Figure S 47: ^{1}H NMR spectrum of [Cu(^{Et}cAAC)(PMes$_2$)] (**2b**) (500 MHz, THF-d_8, 298 K).

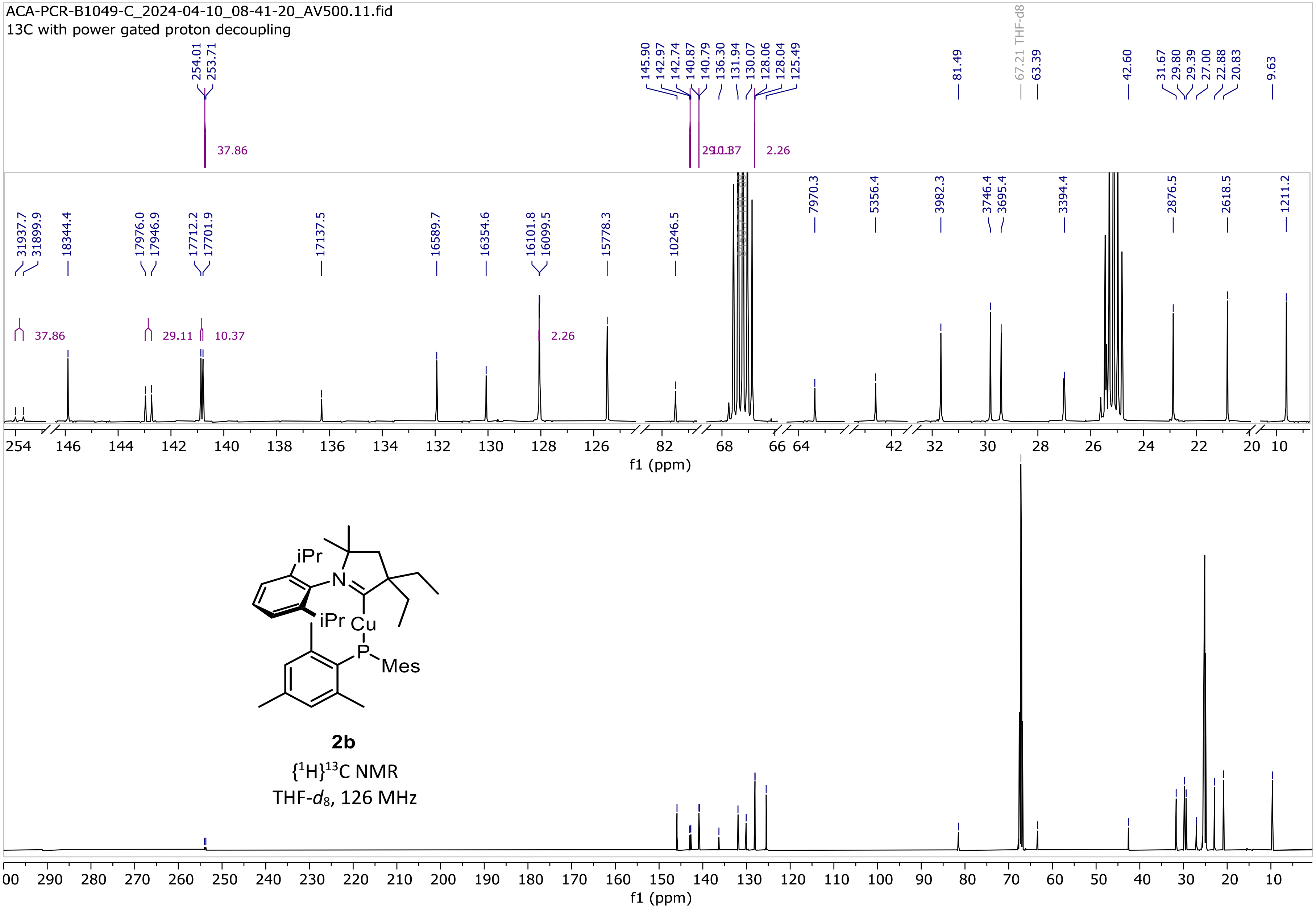

Figure S 48: $\{^1H\}^{13}C$ NMR spectrum of [Cu(^{Et}cAAC)($PMes_2$)] (**2b**) (126 MHz, THF-d_8, 298 K).

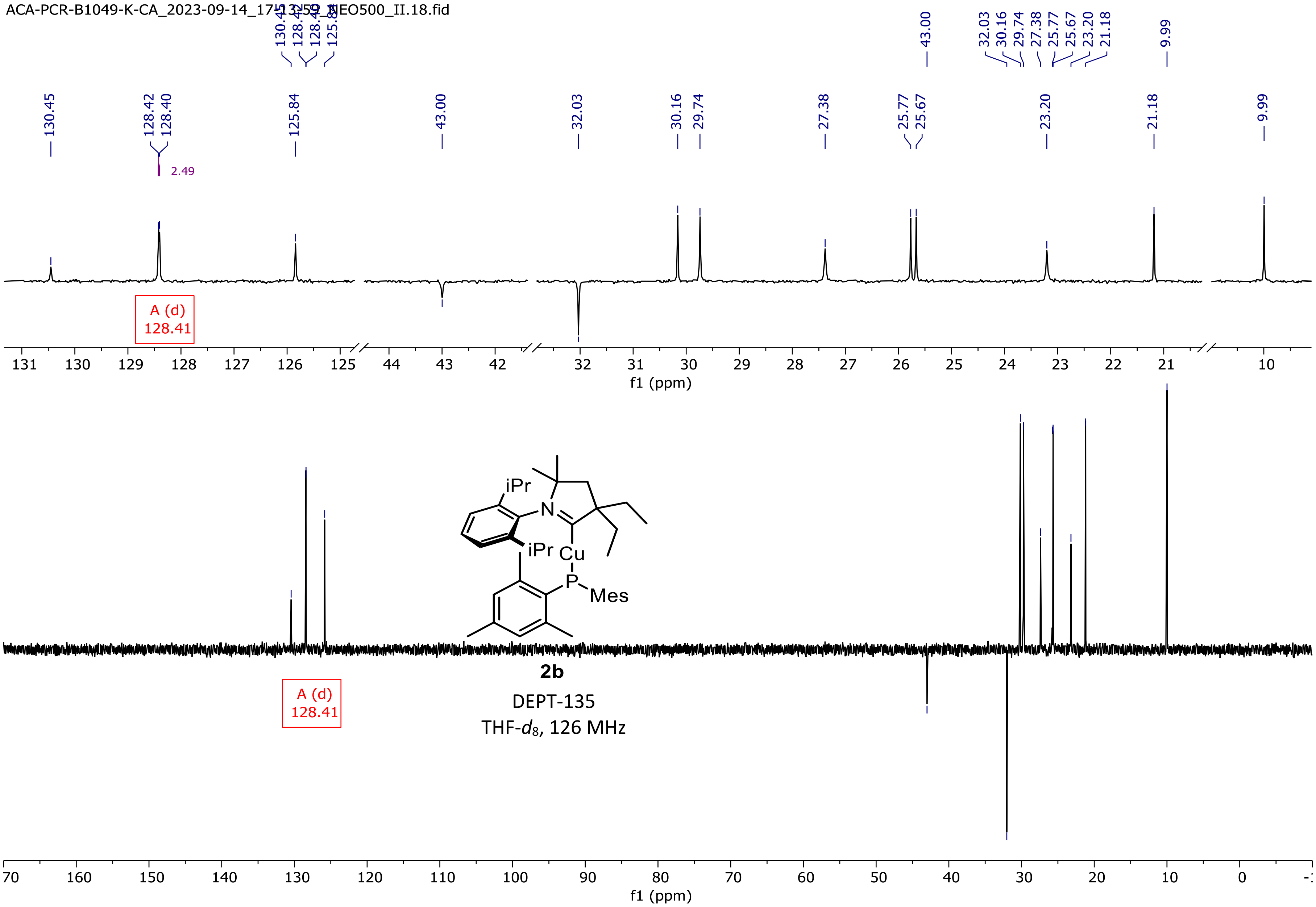

Figure S 49: DEPT-135 NMR spectrum of [Cu(^{Et}cAAC)(PMes$_2$)] (**2b**) (126 MHz, THF-d_8, 298 K).

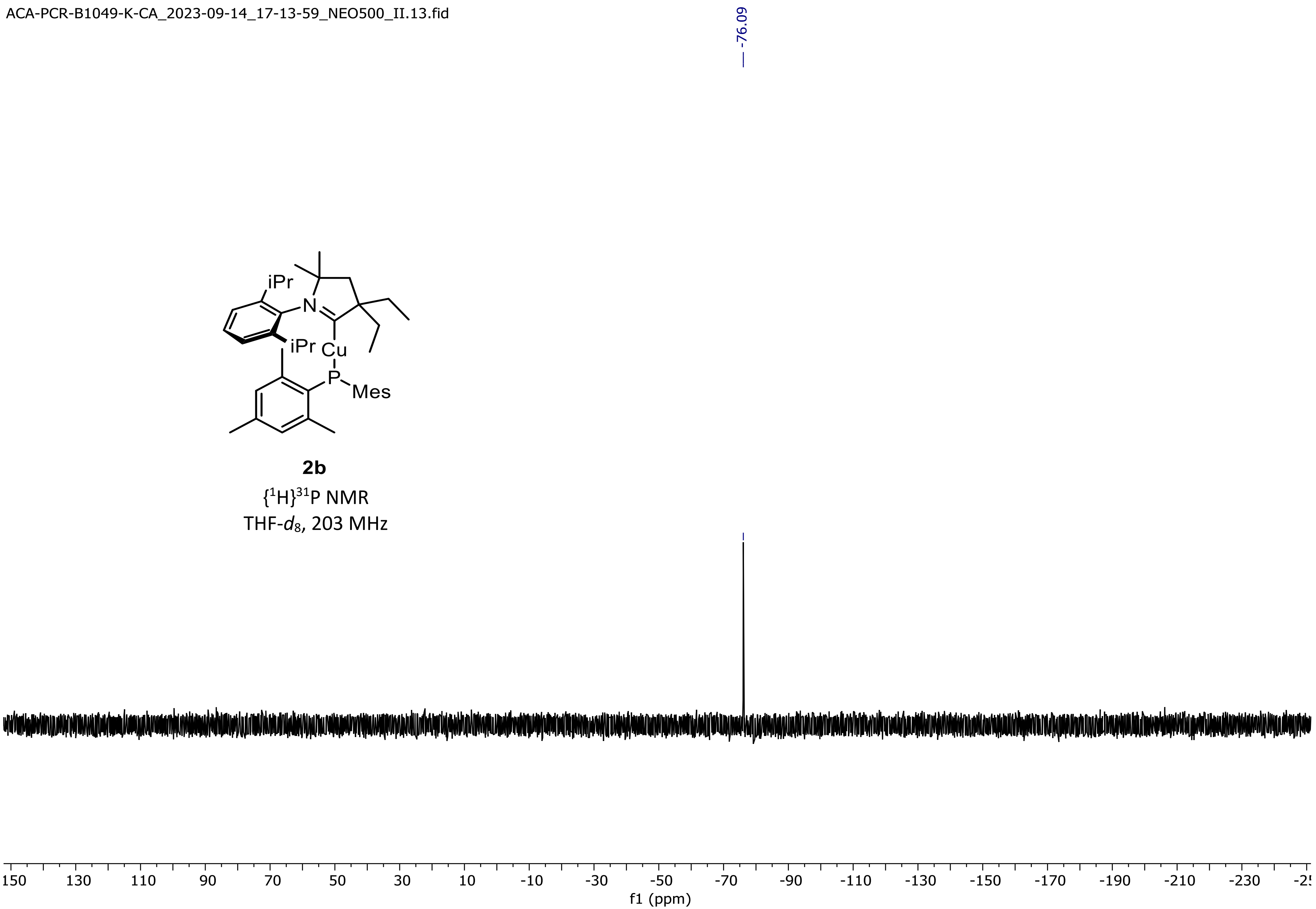

Figure S 50: $\{^1H\}^{31}P$ NMR spectrum of [Cu(^{Et}cAAC)($PMes_2$)] (**2b**) (162 MHz, THF-d_8, 298 K).

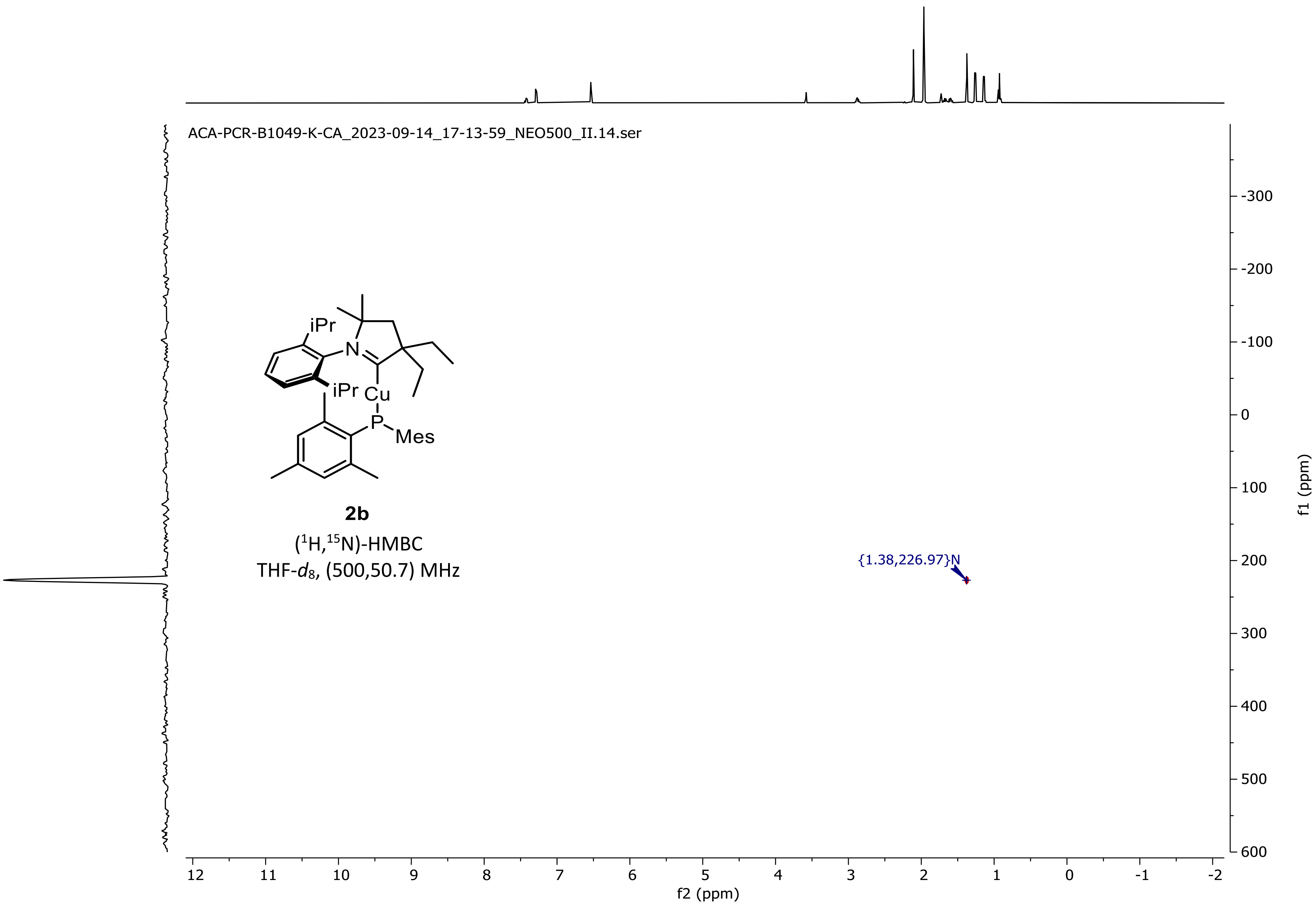

Figure S 51: (^{1}H,^{15}N)-HMBC NMR spectrum of [Cu(^{Et}cAAC)(PMes$_2$)] (**2b**) (500 MHz, 51 MHz, THF-d_8, 298 K).

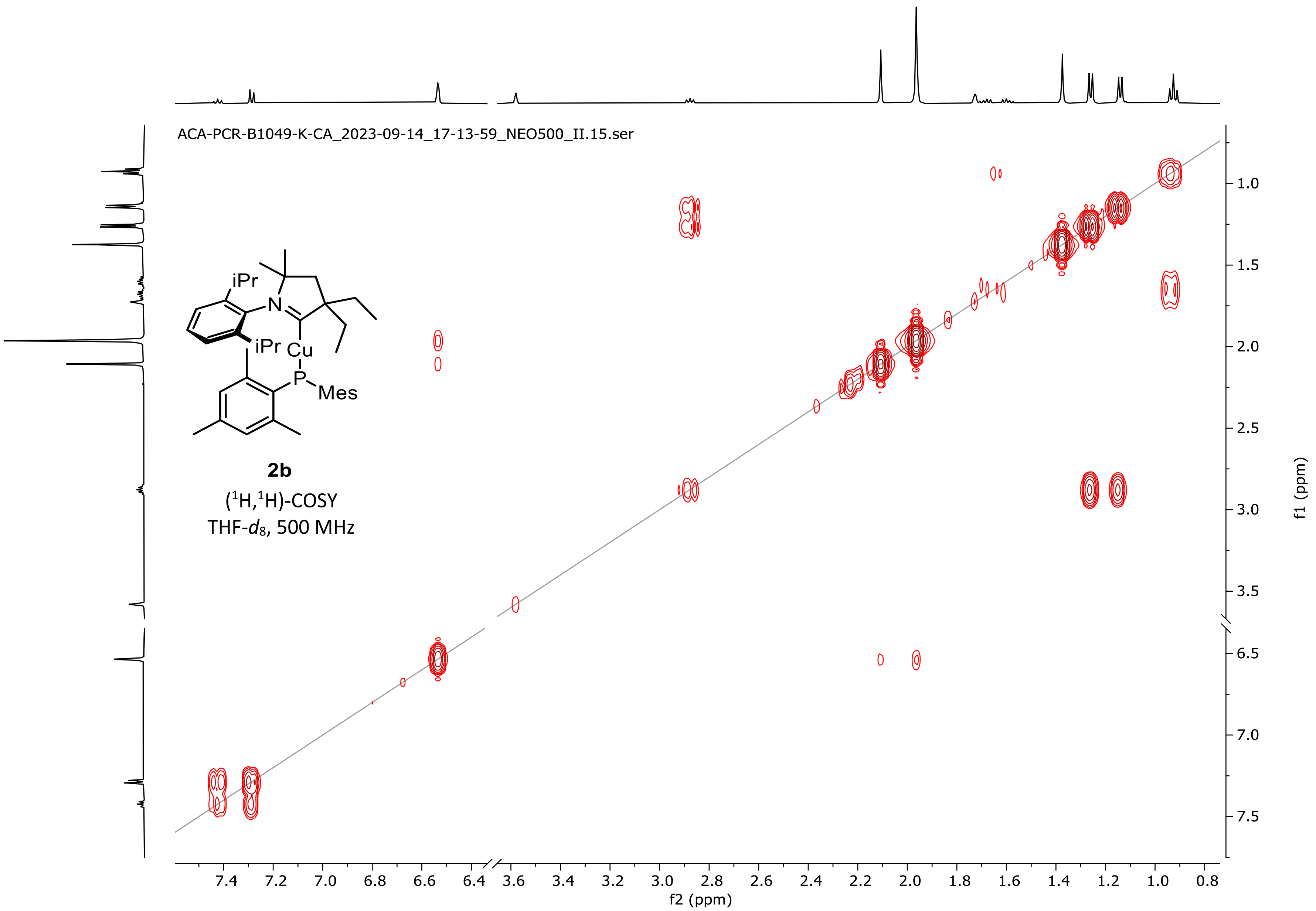

Figure S 52: (^{1}H,^{1}H)-COSY NMR spectrum of [Cu(^{Et}cAAC)(PMes$_2$)] (**2b**) (500 MHz, THF-d_8, 298 K).

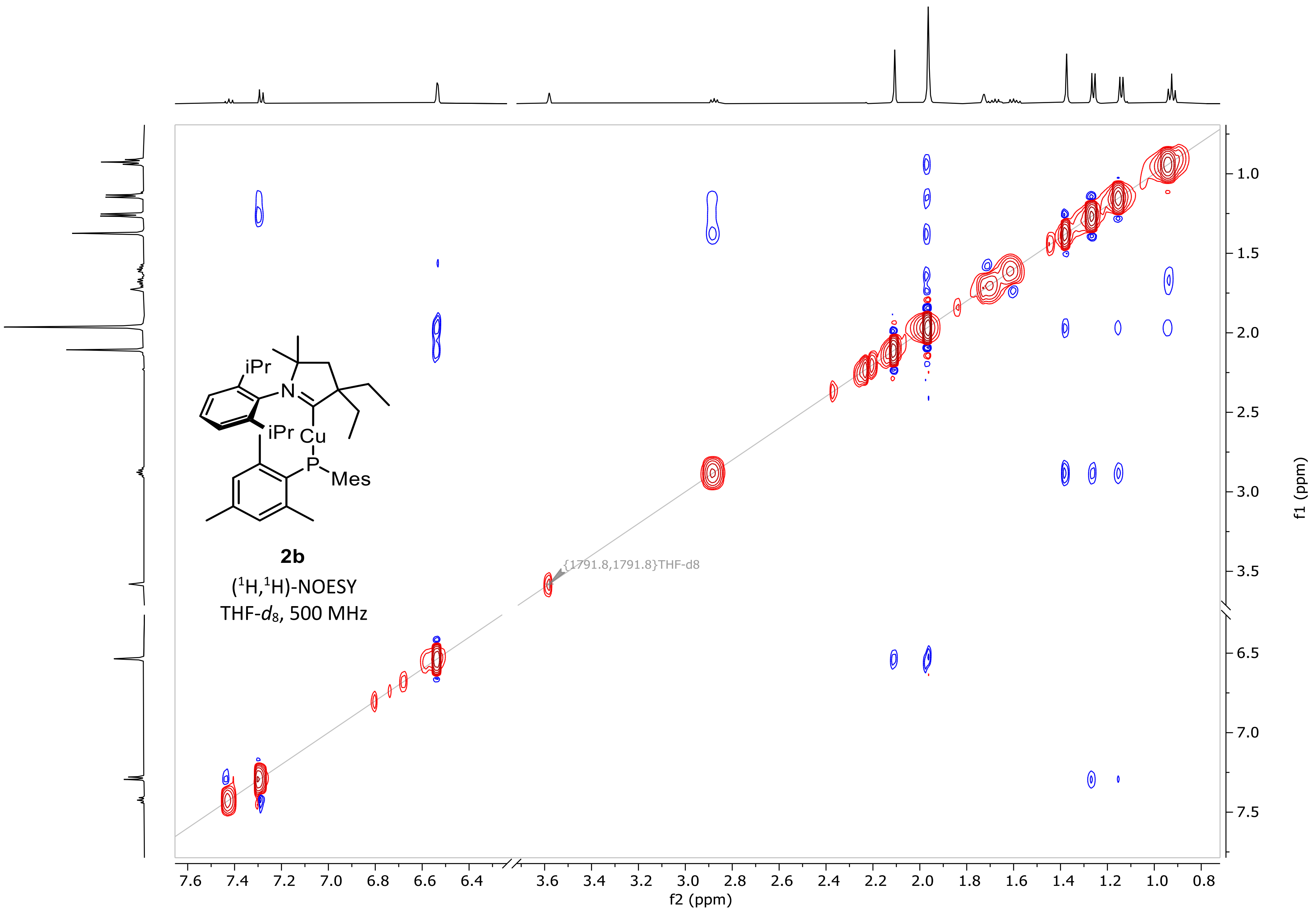

Figure S 53: (^{1}H,^{1}H)-NOESY NMR spectrum of [Cu(^{Et}cAAC)(PMes$_2$)] (**2b**) (500 MHz, THF-d_8, 298 K).

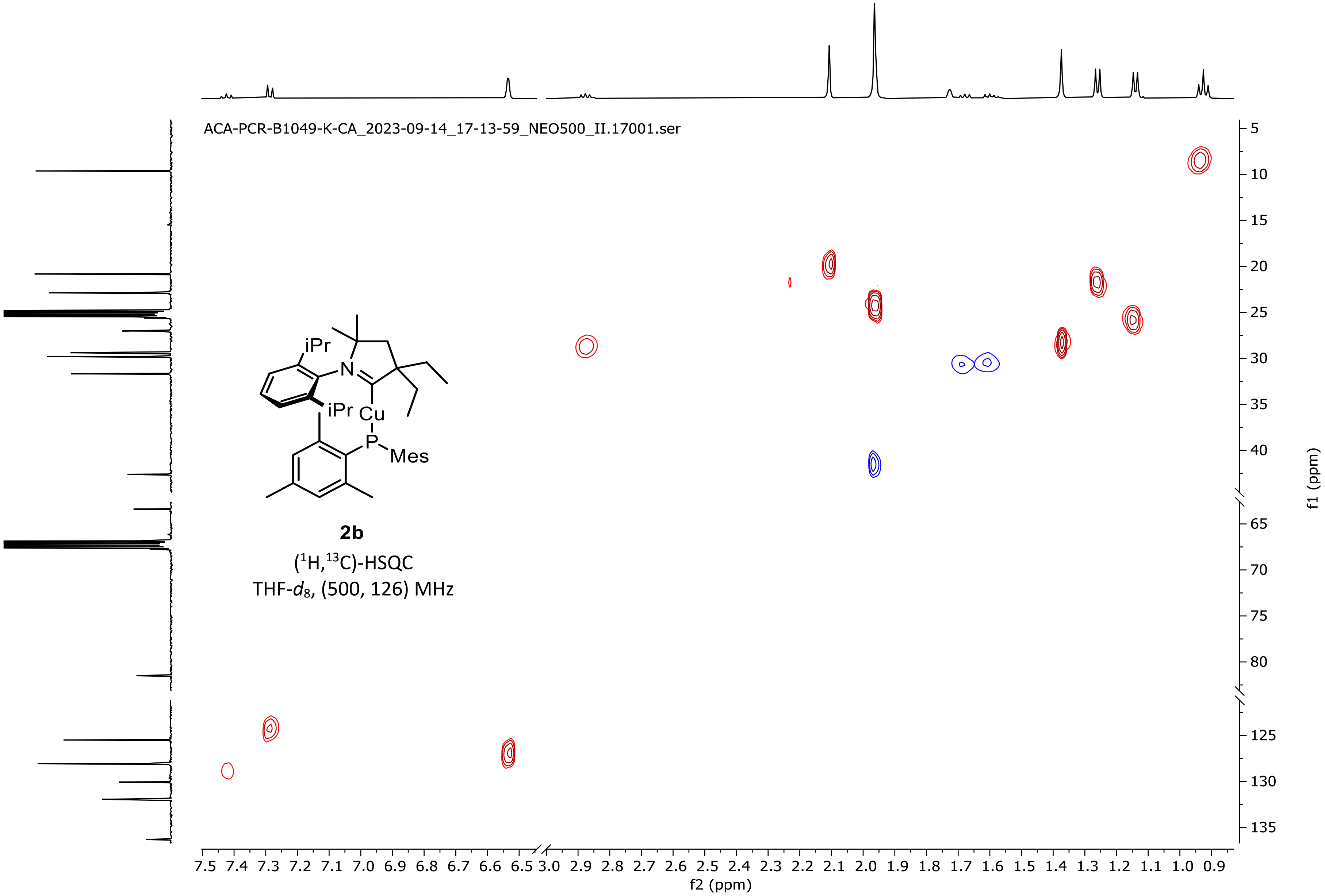

Figure S 54: (^{1}H,^{13}C)-HSQC NMR spectrum of [Cu(^{Et}cAAC)($PMes_2$)] (**2b**) (500 MHz, 126 MHz, THF-d_8, 298 K).

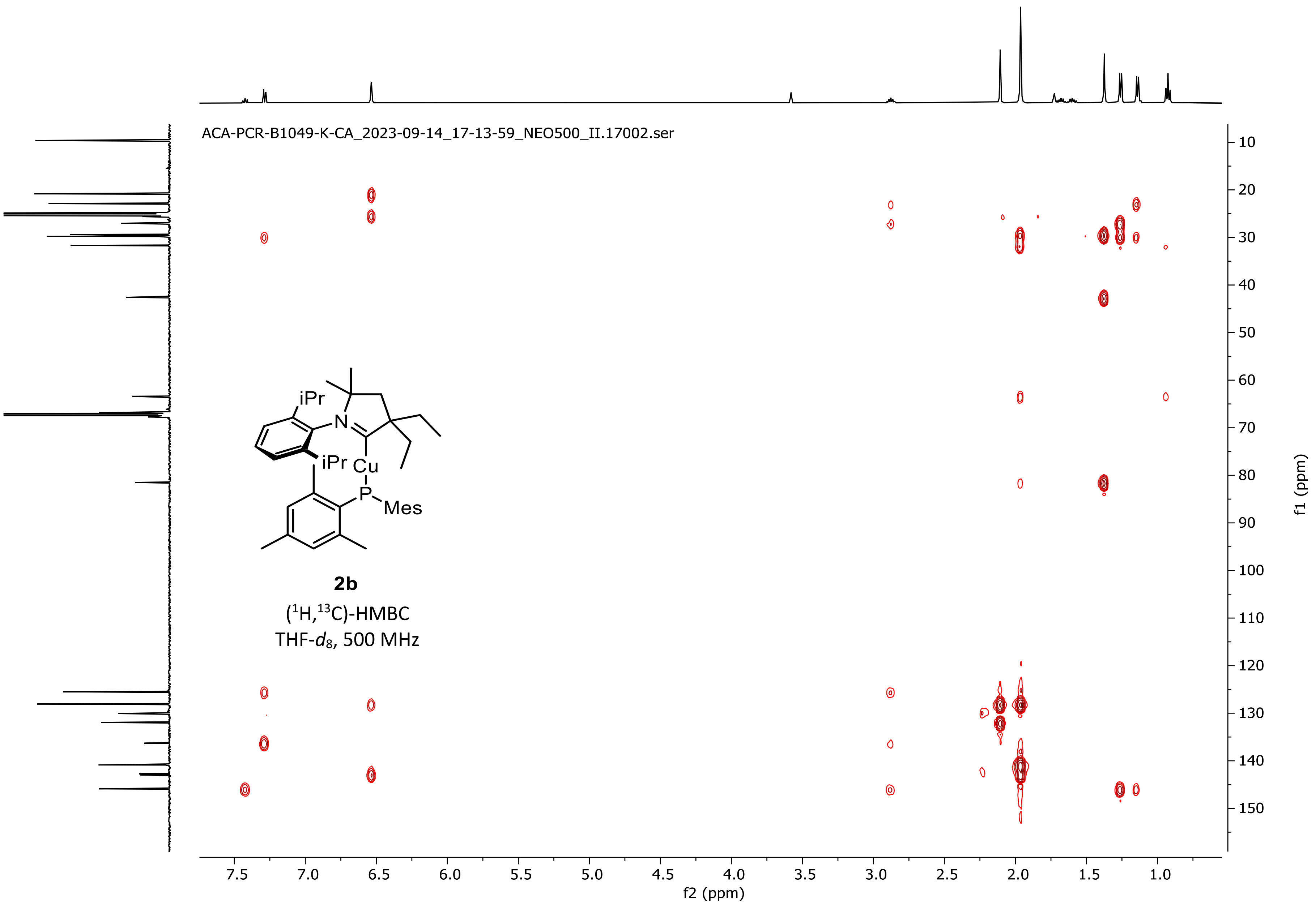

Figure S 55: (^{1}H,^{13}C)-HMBC NMR spectrum of [Cu(^{Et}cAAC)(PMes$_2$)] (**2b**) (500 MHz, 126 MHz, THF-d_8, 298 K).

NMR spectra of 2c [Cu(^{Cy}cAAC)(PMes$_2$)]

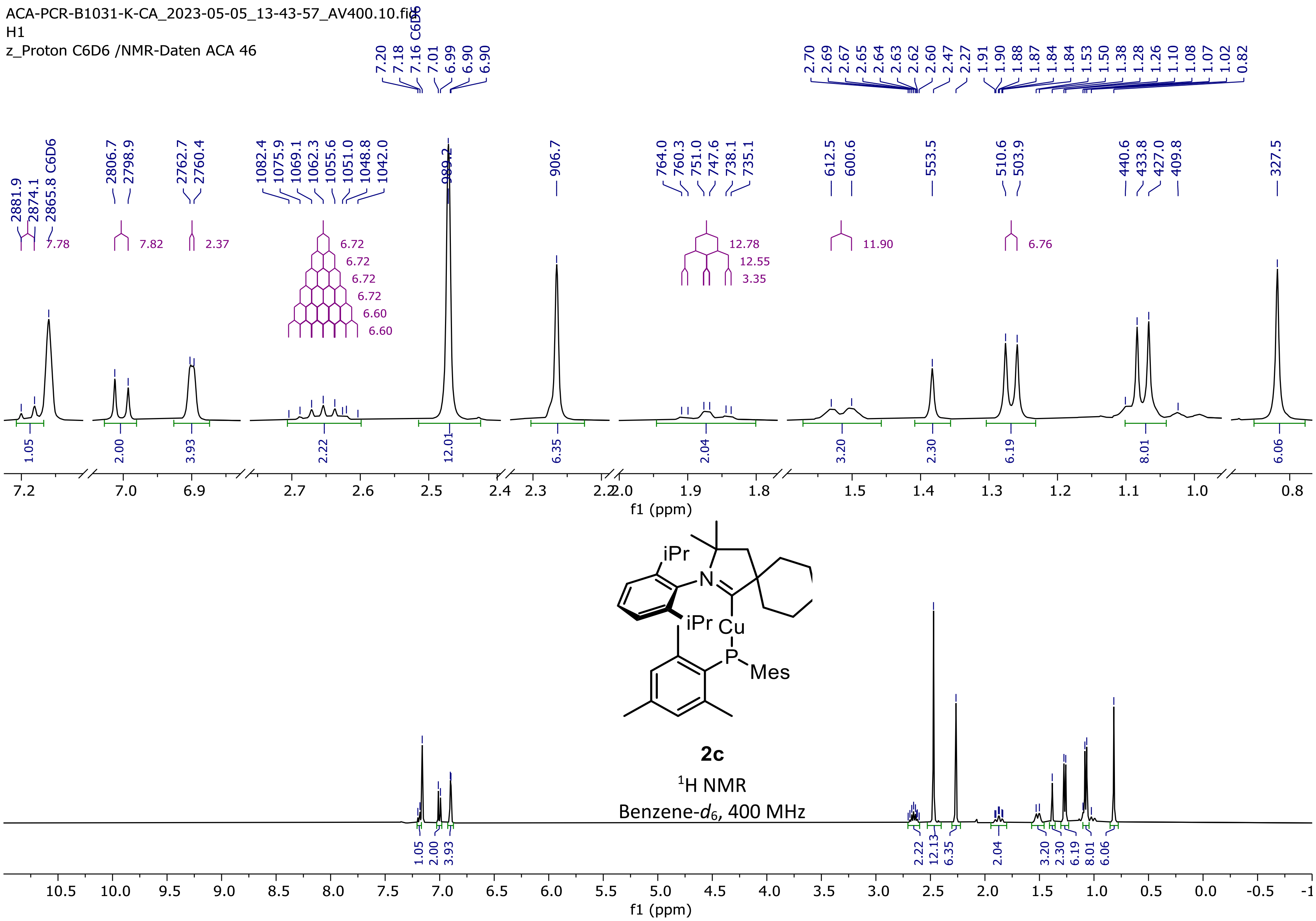

Figure S 56: ^{1}H NMR spectrum of [Cu(^{Cy}cAAC)(PMes$_2$)] (**2c**) (400 MHz, C_6D_6, 298 K).

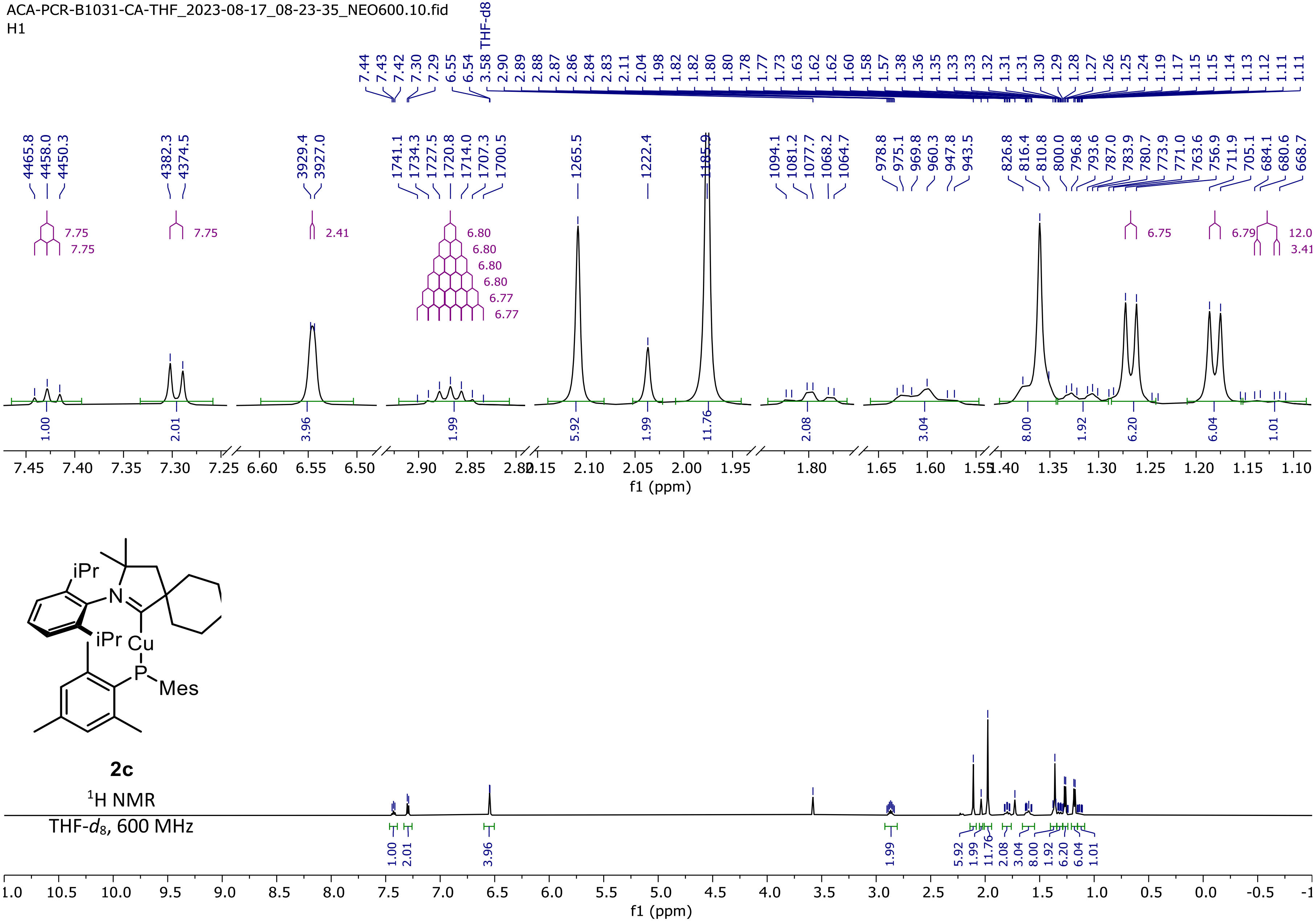

Figure S 57: ^{1}H NMR spectrum of [Cu(^{Cy}cAAC)(PMes$_2$)] (**2c**) (600 MHz, THF-d_8, 298 K).

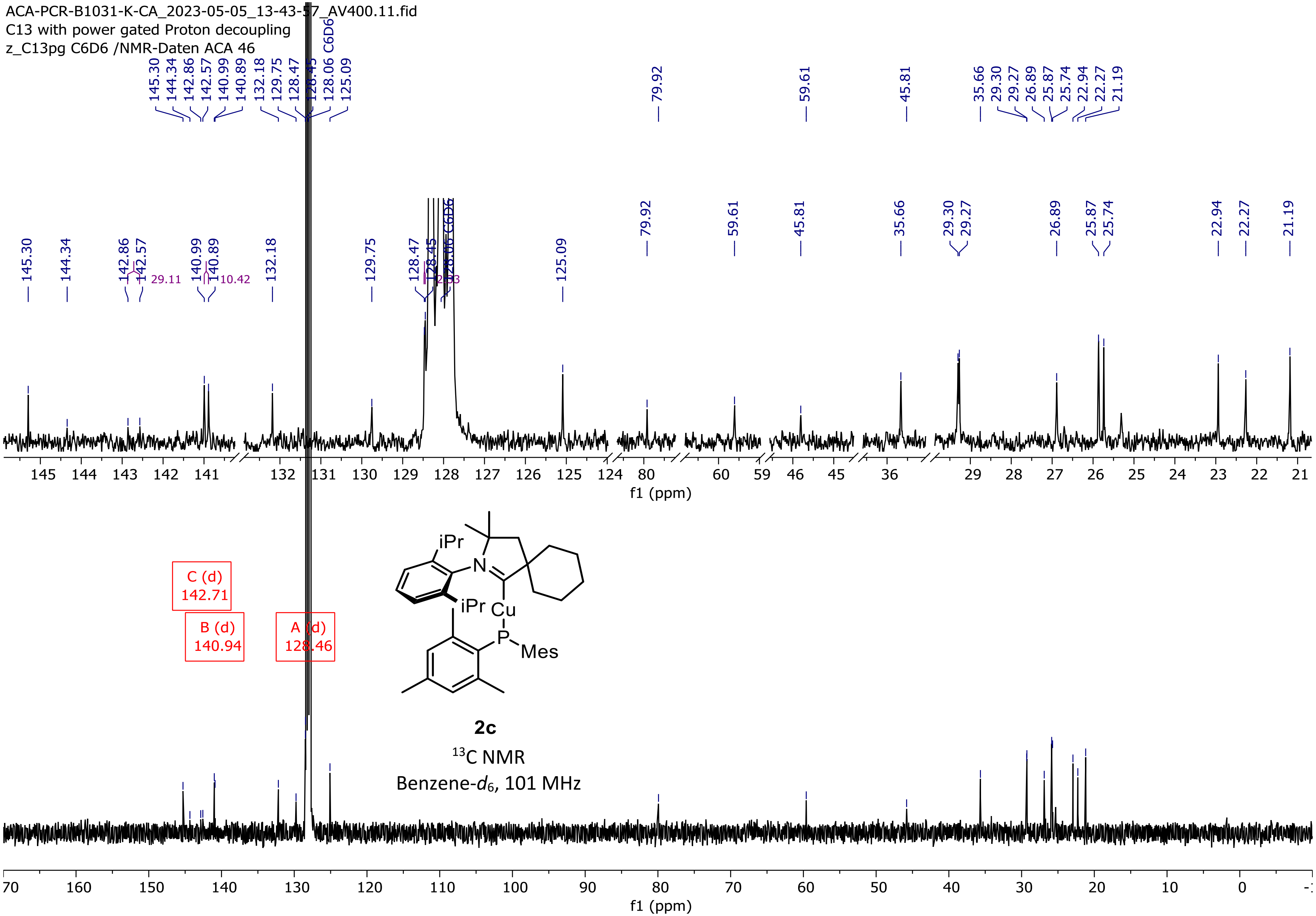

Figure S 58: {^{1}H}^{13}C NMR spectrum of [Cu(^{Cy}cAAC)(PMes$_2$)] (**2c**) (101 MHz, C_6D_6, 298 K).

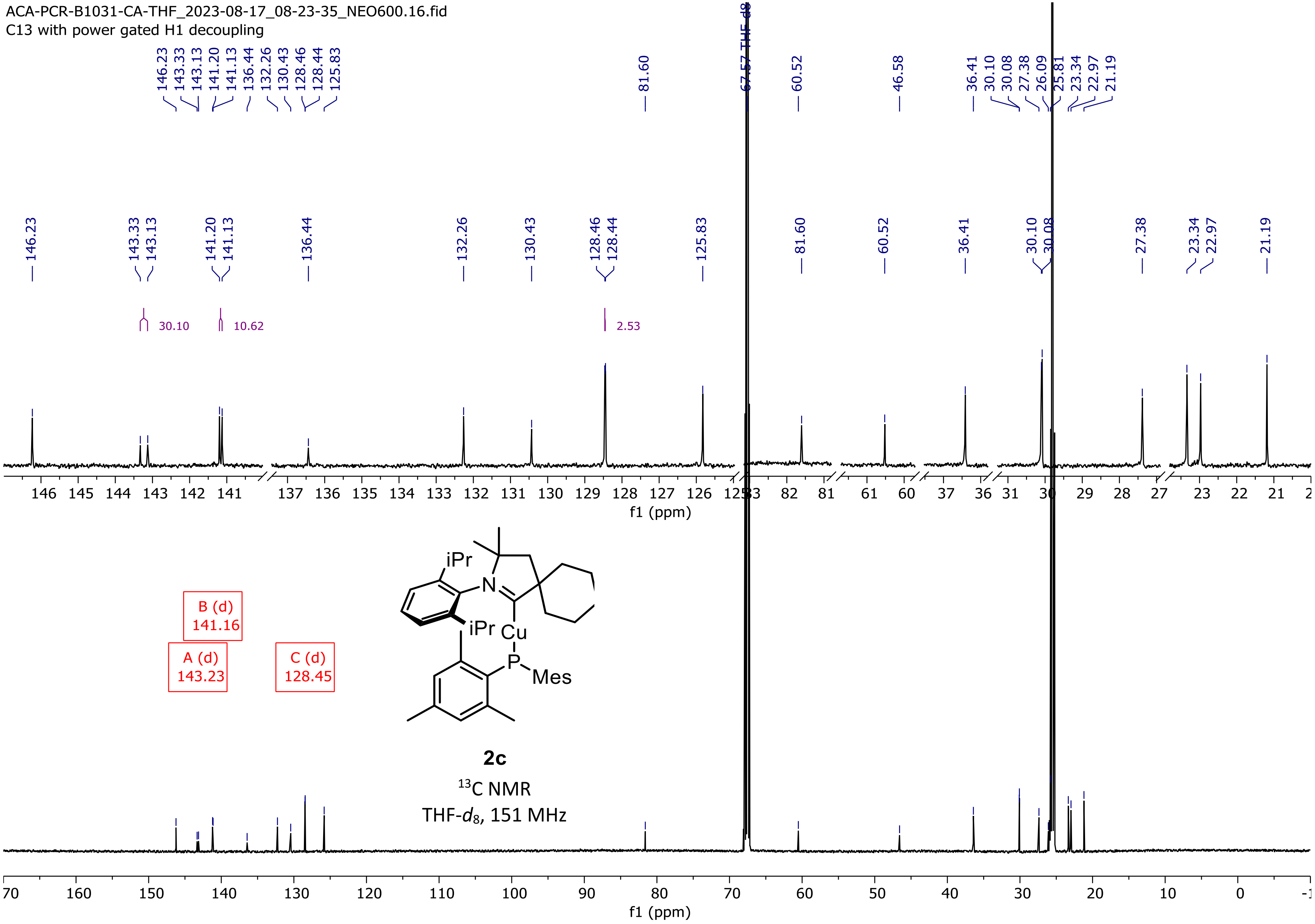

Figure S 59: {^{1}H}^{13}C NMR spectrum of [Cu(^{Cy}cAAC)(PMes$_2$)] (**2c**) (151 MHz, THF-d$_8$, 298 K).

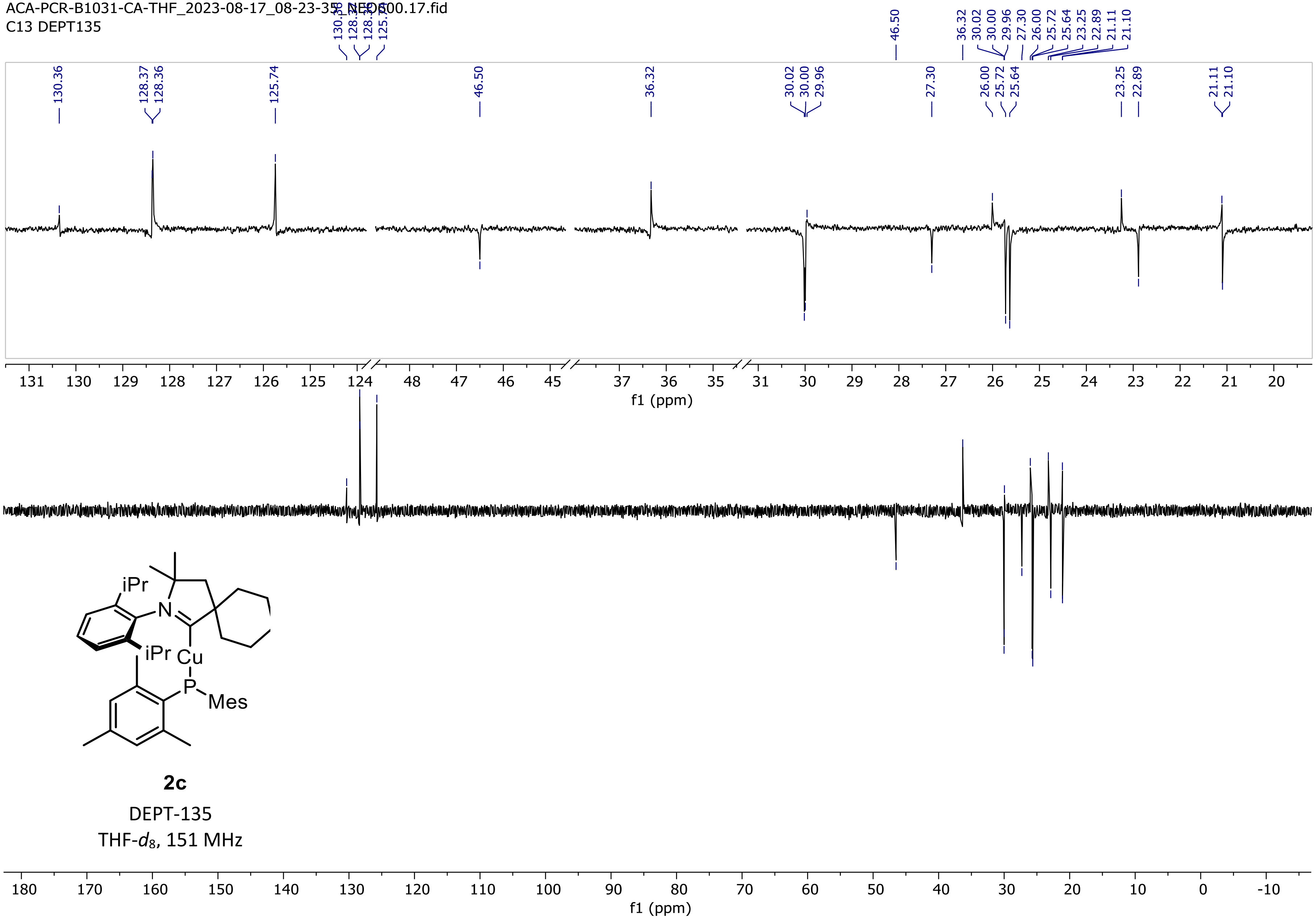

Figure S 60: DEPT-135 NMR spectrum of [Cu(^{Cy}cAAC)($PMes_2$)] (**2c**) (151 MHz, THF-d_8, 298 K).

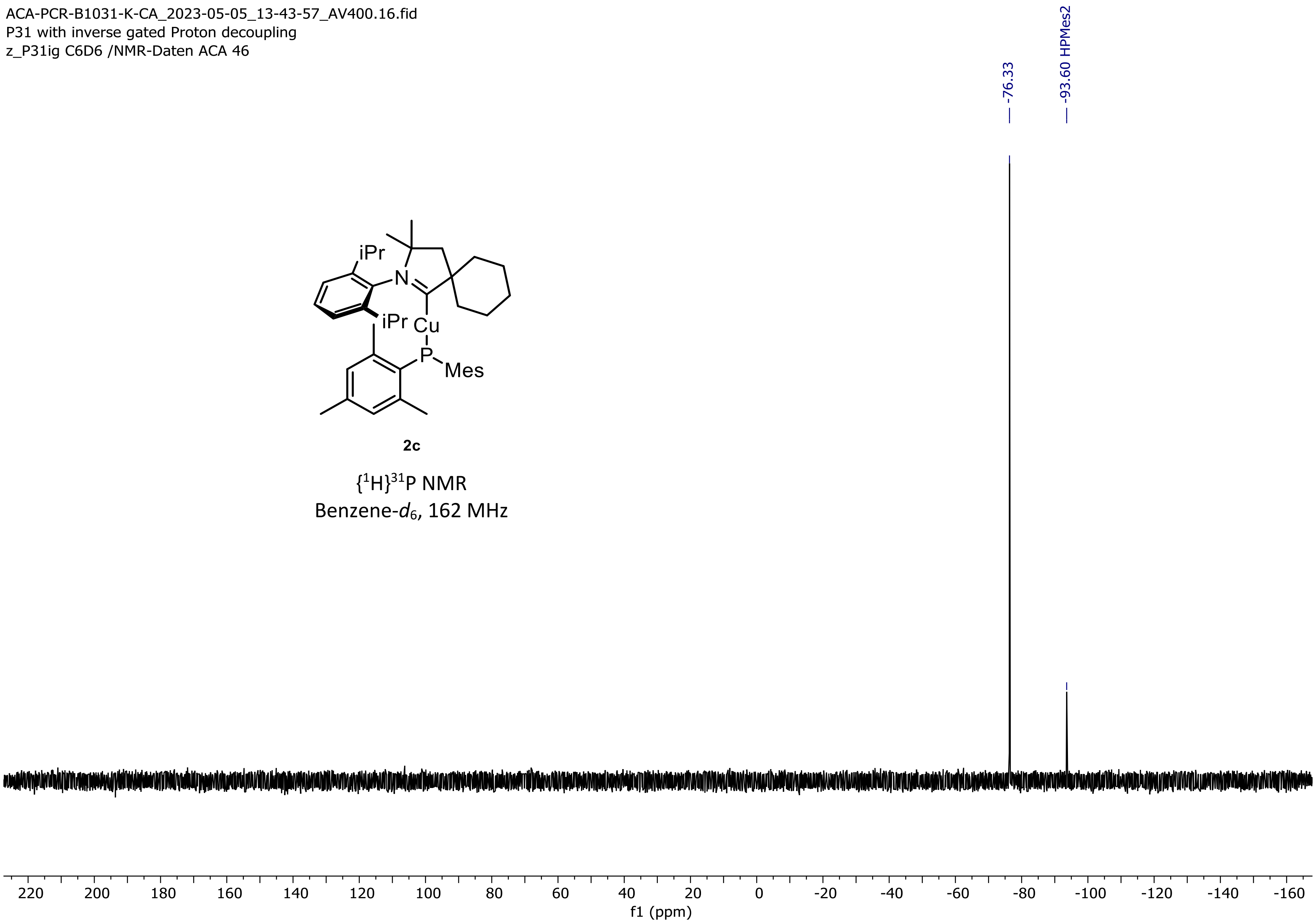

Figure S 61: $\{^{1}H\}^{31}P$ NMR spectrum of [Cu(^{Cy}cAAC)($PMes_2$)] (**2c**) (126 MHz, C_6D_6, 298 K).

ACA-PCR-B1031-CA-THF_2023-08-17_08-23-35_NEO600.11.fid
P31 with inverse gated H1 decoupling

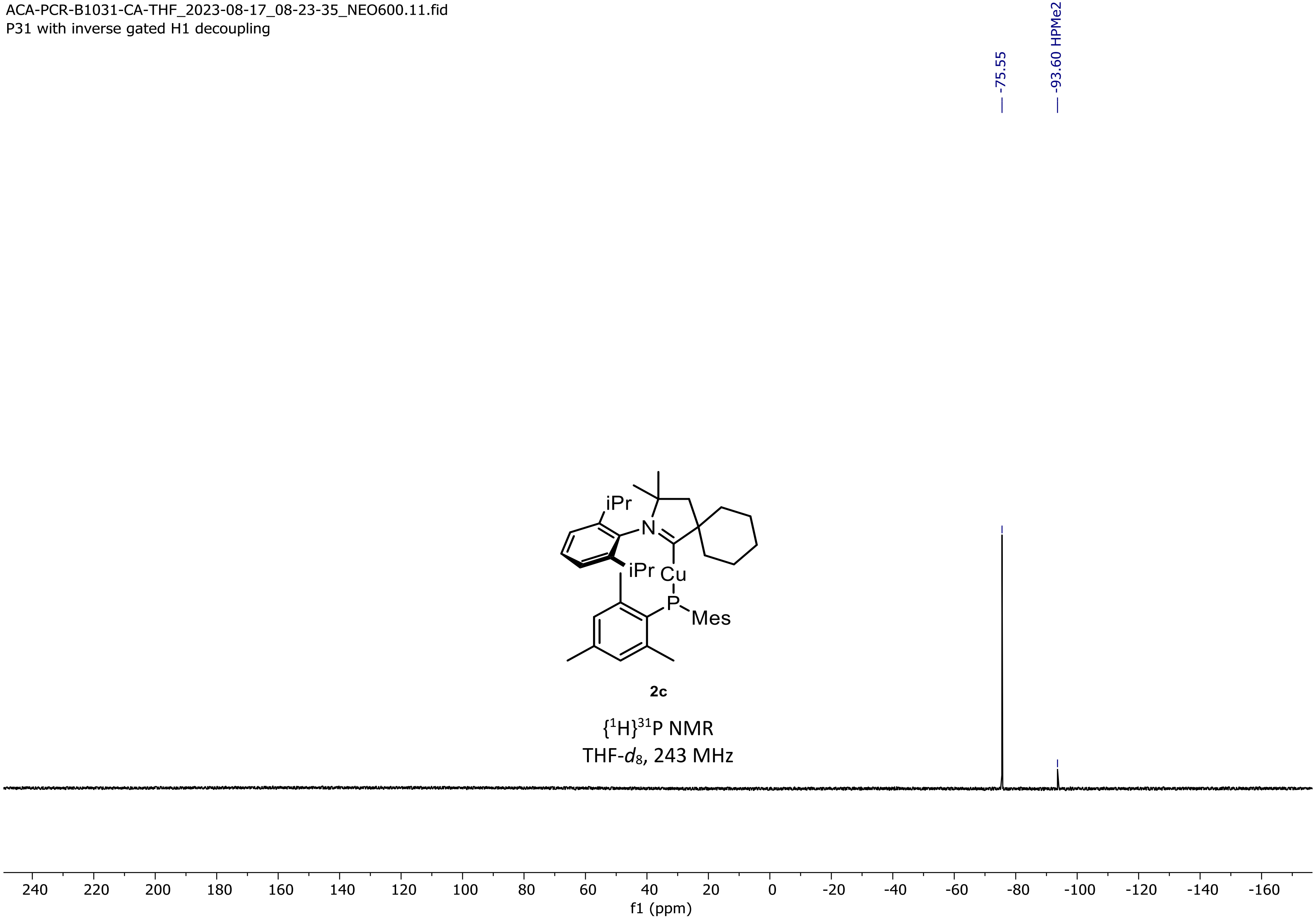

Figure S 62: $\{^1H\}^{31}P$ NMR spectrum of [Cu(^{Cy}cAAC)($PMes_2$)] (**2c**) (243 MHz, THF-d_8, 298 K).

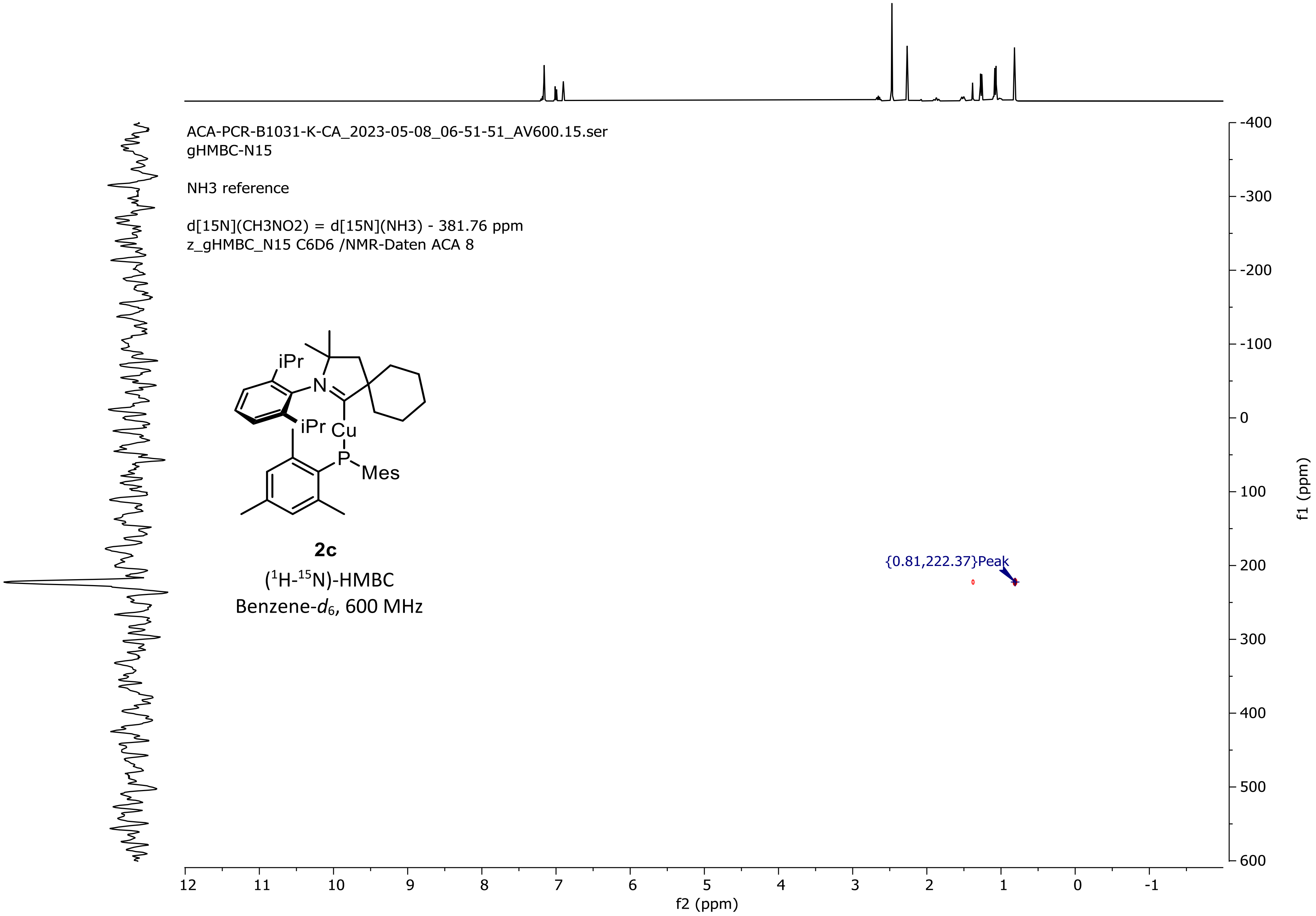

Figure S 63: (^{1}H,^{15}N)-HMBC NMR spectrum of [Cu(^{Cy}cAAC)(PMes$_2$)] (**2c**) (600 MHz, 61 MHz, C_6D_6, 298 K).

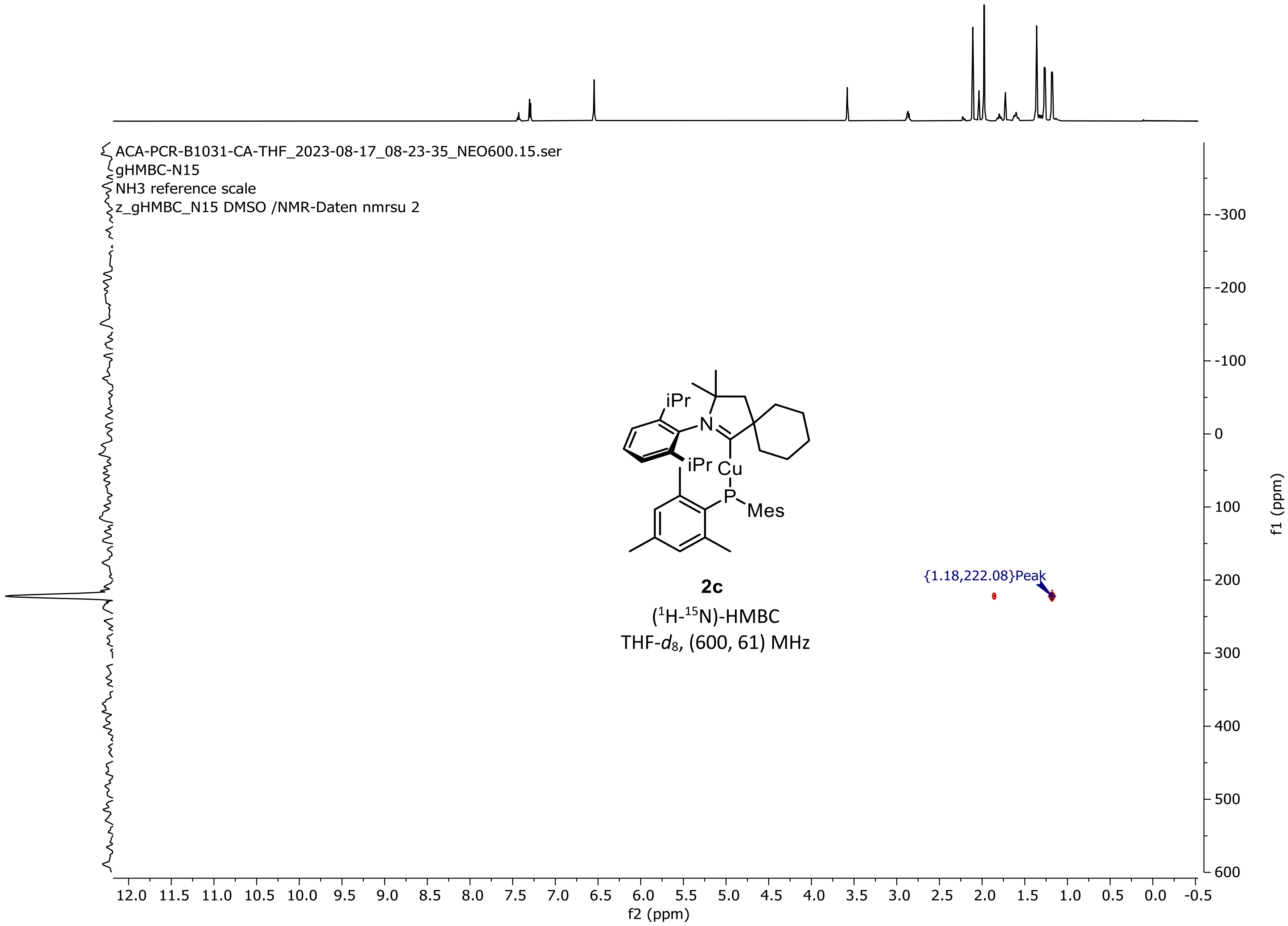

Figure S 64: (^{1}H,^{15}N)-HMBC NMR spectrum of [Cu(^{Cy}cAAC)(PMes$_2$)] (**2c**) (600 MHz, 61 MHz, THF-d_8, 298 K).

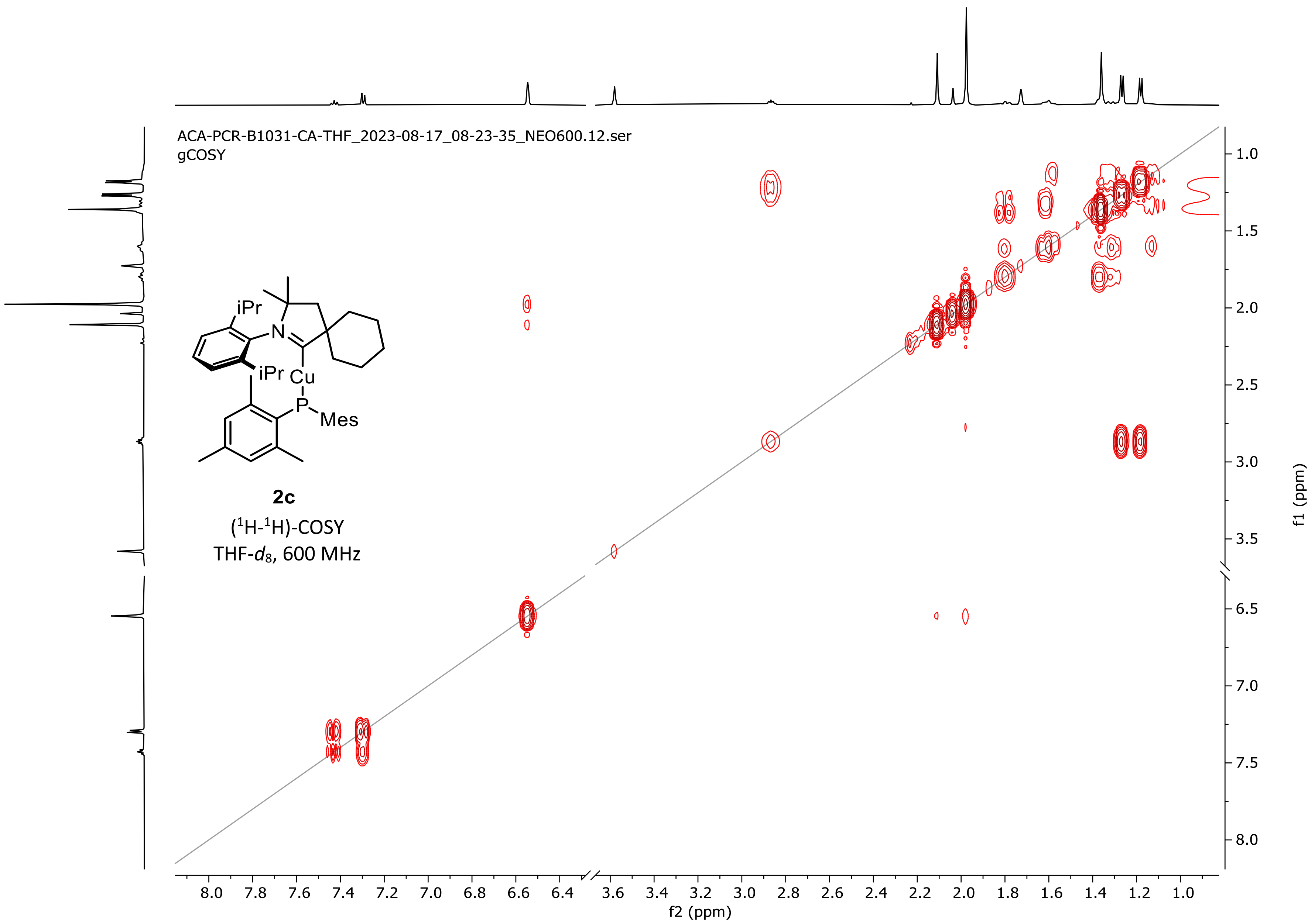

Figure S 65: (^{1}H,^{1}H)-COSY NMR spectrum of [Cu(^{Cy}cAAC)(PMes$_2$)] (**2c**) (600 MHz, THF-d_8, 298 K).

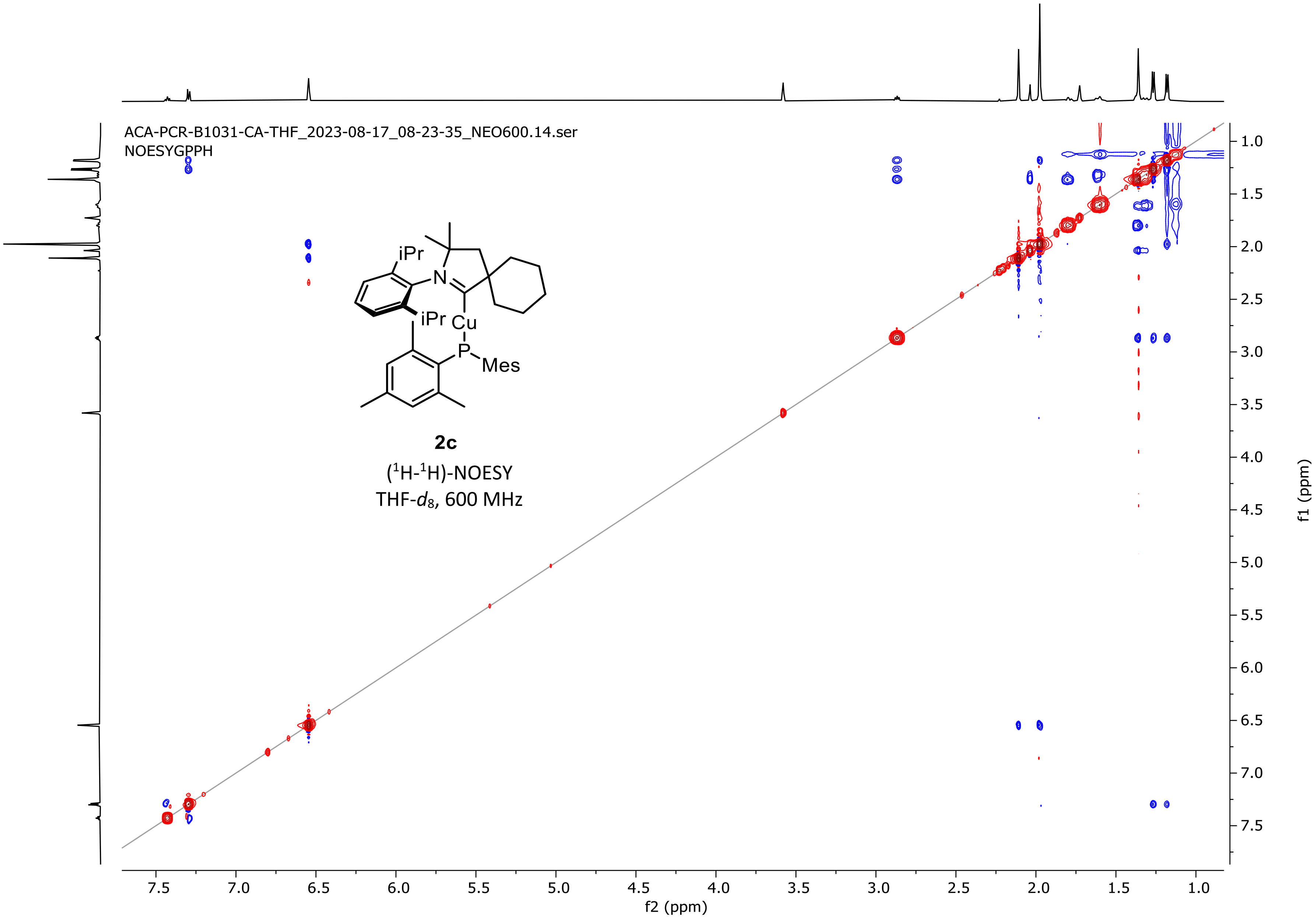

Figure S 66: (^{1}H,^{1}H)-NOESY NMR spectrum of [Cu(^{Cy}cAAC)(PMes$_2$)] (**2c**) (600 MHz, THF-d_8, 298 K).

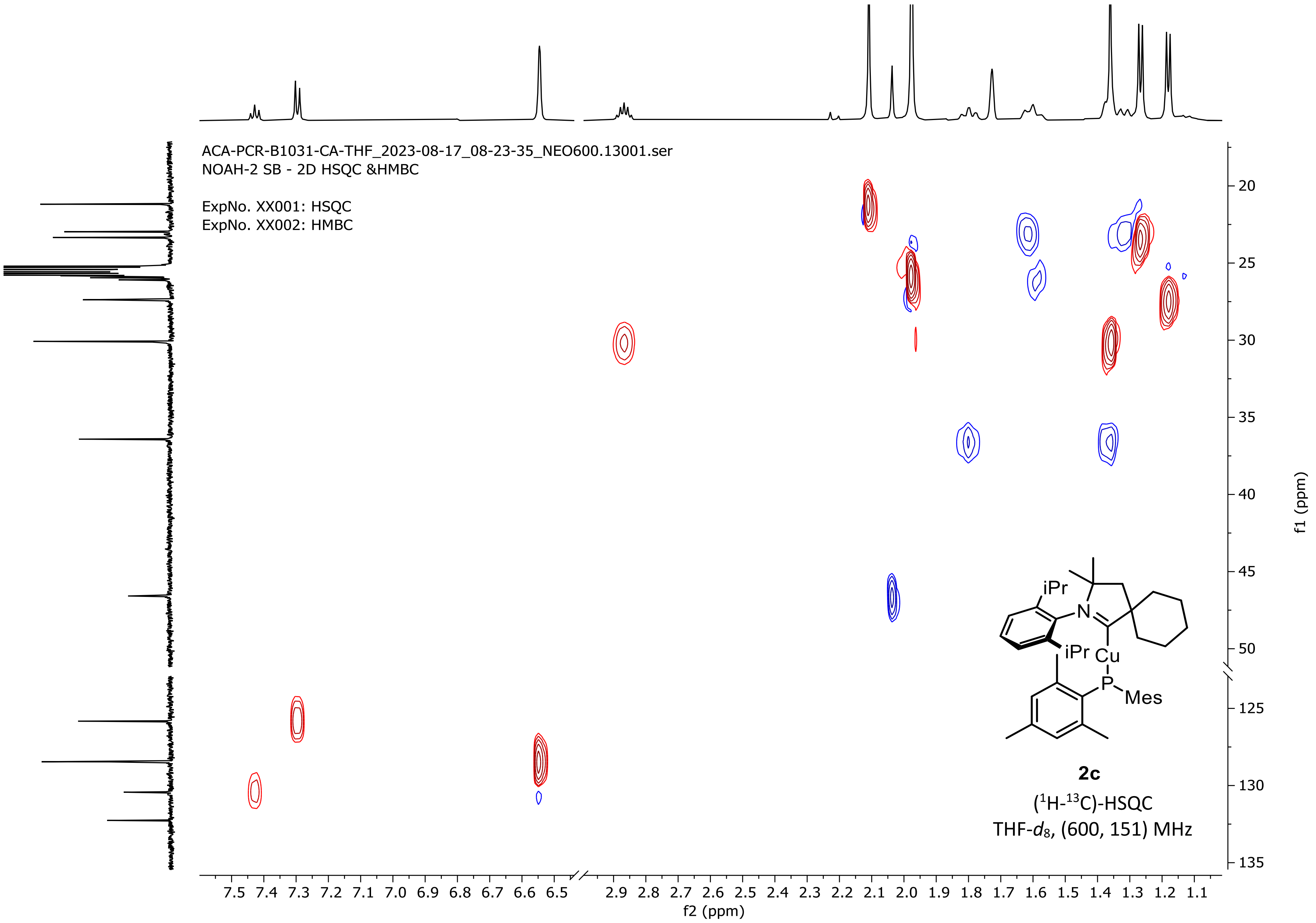

Figure S 67: (^{1}H,^{13}C)-HSQC NMR spectrum of [Cu(^{Cy}cAAC)(PMes$_2$)] (**2c**) (600 MHz, 151MHz, THF-*d*$_8$, 298 K).

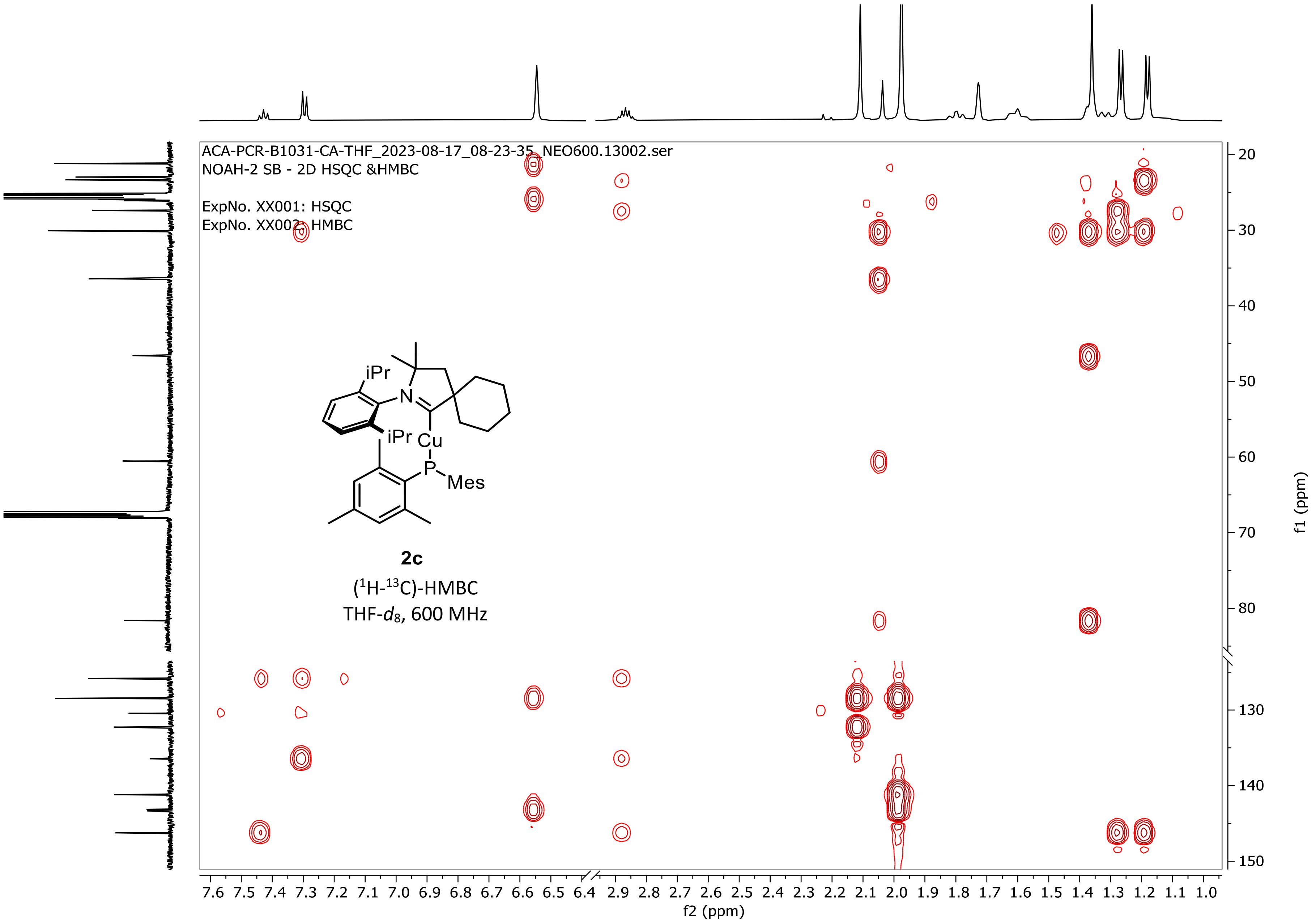

Figure S 68: (^{1}H,^{13}C)-HMBC NMR spectrum of [Cu(^{Cy}cAAC)(PMes$_2$)] (**2c**) (600 MHz, 151 MHz, THF-d_8, 298 K).

NMR spectra of 2d [Cu(^{Ment}cAAC)(PMes$_2$)]

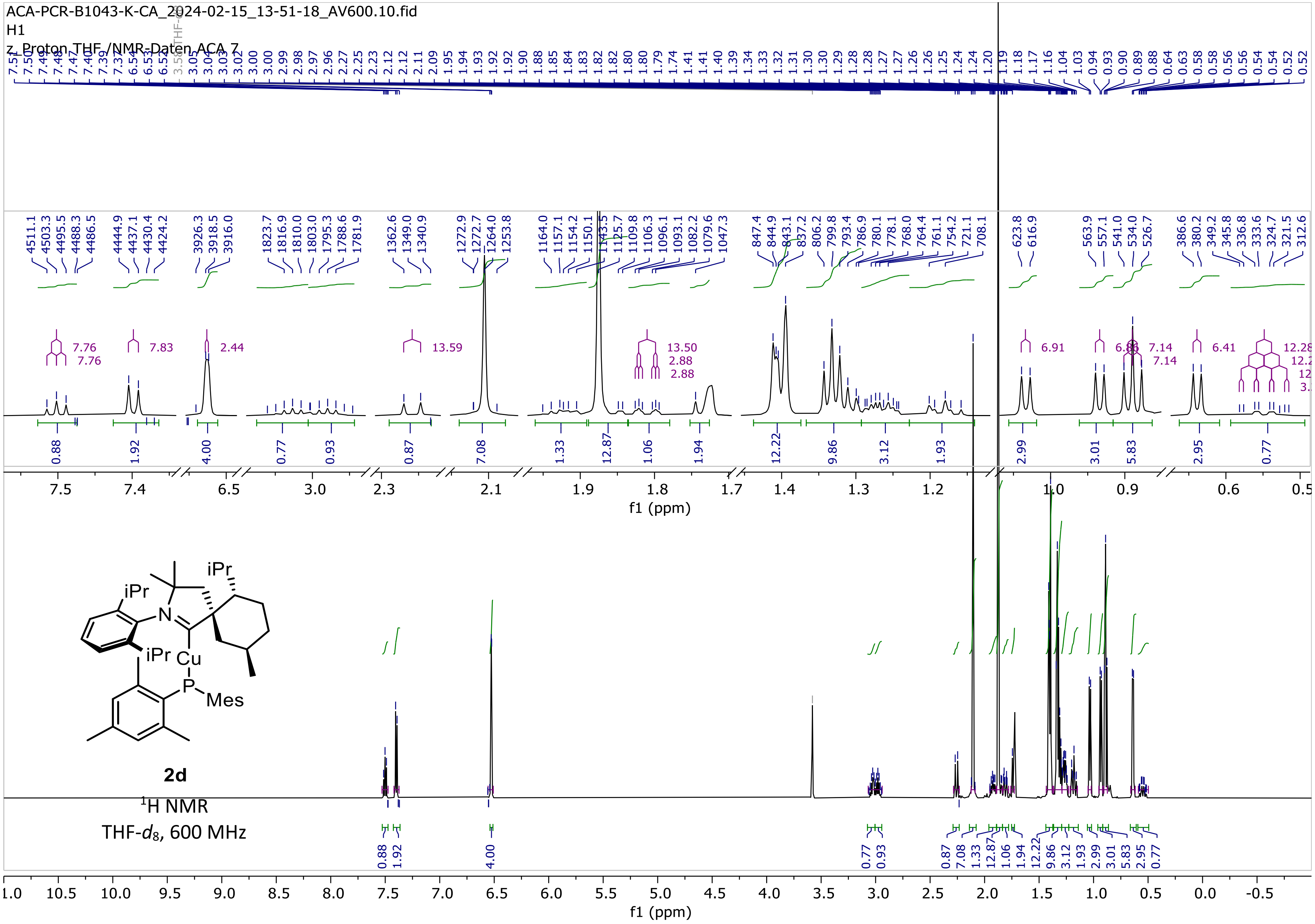

Figure S 69: ^{1}H NMR spectrum of [Cu(^{Ment}cAAC)(PMes$_2$)] (**2d**) (600 MHz, THF-d_8, 298 K).

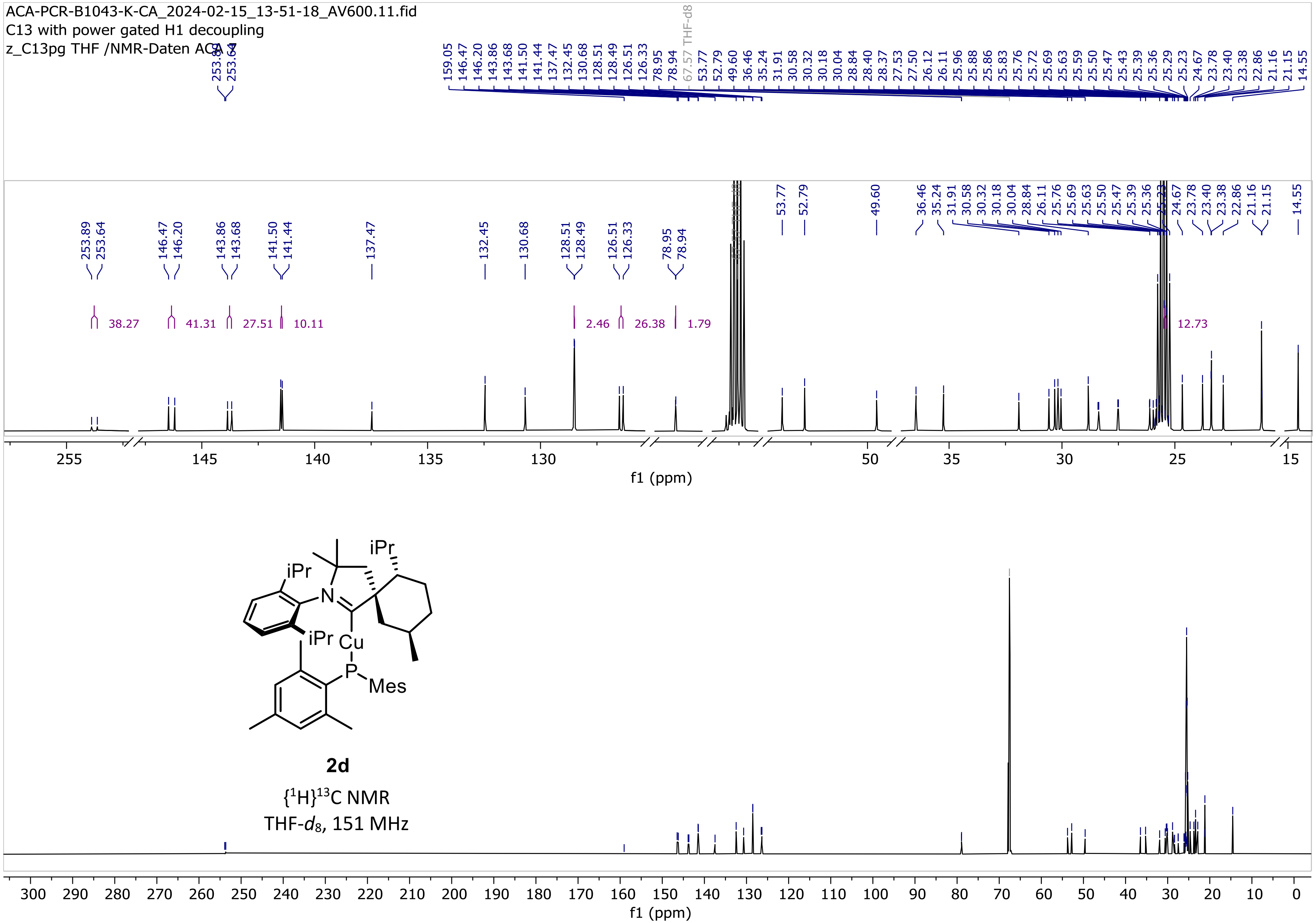

Figure S 70: {^{1}H}^{13}C NMR spectrum of [Cu(^{Ment}cAAC)($PMes_2$)] (**2d**) (151 MHz, THF-d_8, 298 K).

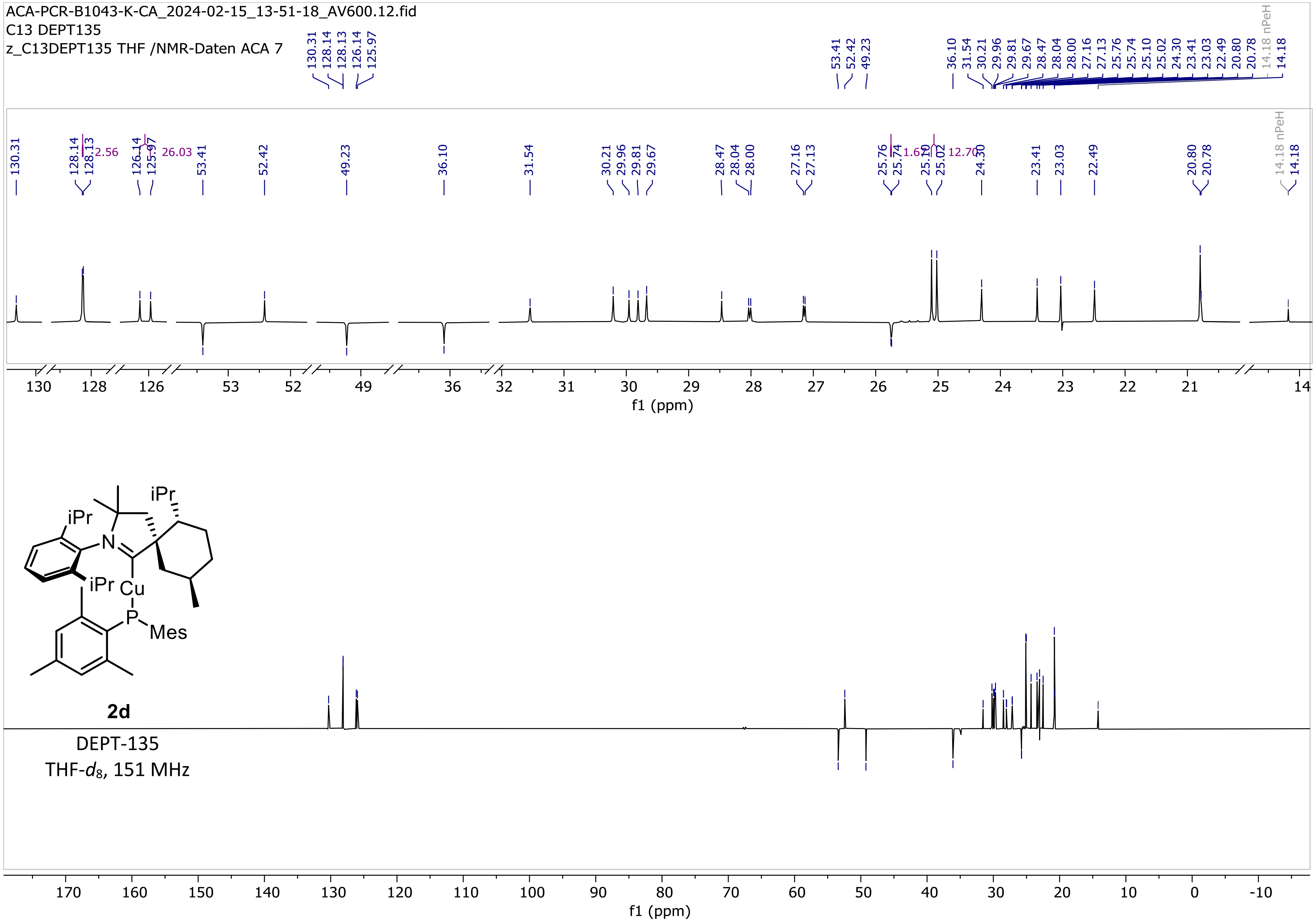

Figure S 71: DEPT-135 NMR spectrum of [Cu(^{Ment}cAAC)(PMes$_2$)] (**2d**) (126 MHz, THF-d_8, 298 K).

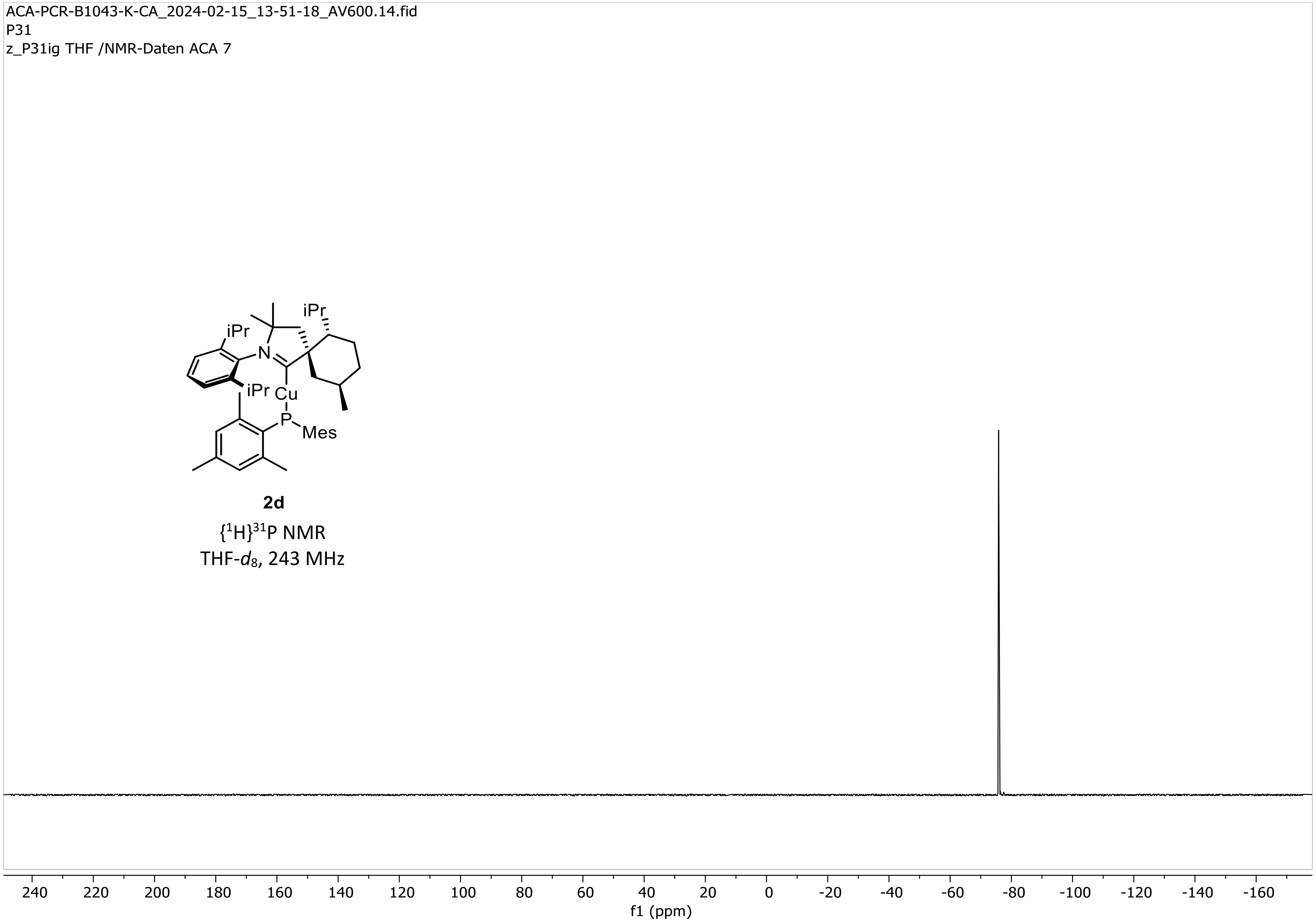

Figure S 72: $\{^1H\}^{31}P$ NMR spectrum of [Cu(^{Ment}cAAC)($PMes_2$)] (**2d**) (243 MHz, THF-d_8, 298 K).

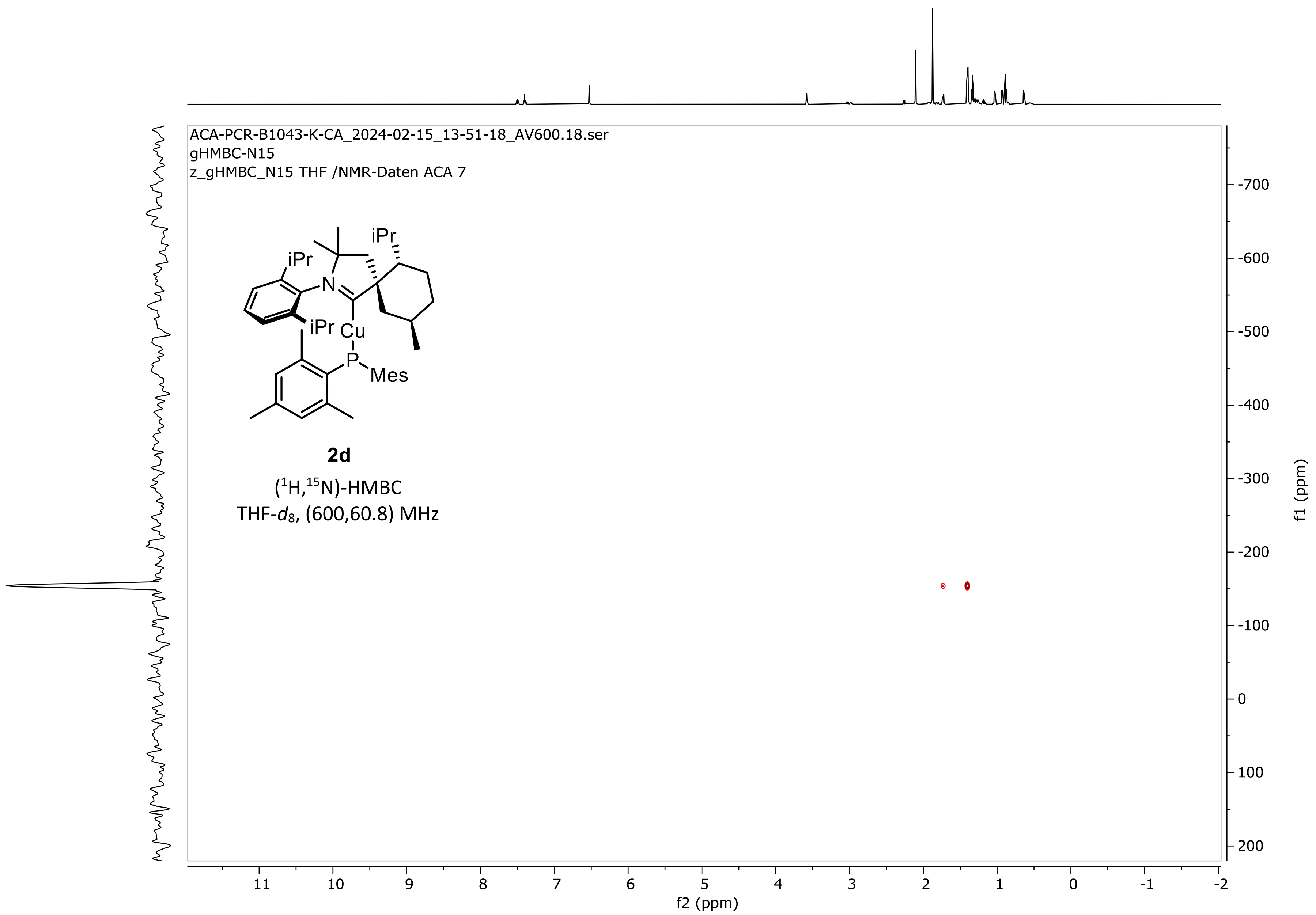

Figure S 73: (^{1}H,^{15}N)-HMBC NMR spectrum of [Cu(^{Ment}cAAC)(PMes$_2$)] (**2d**) (600 MHz, 61 MHz, THF-d_8, 298 K).

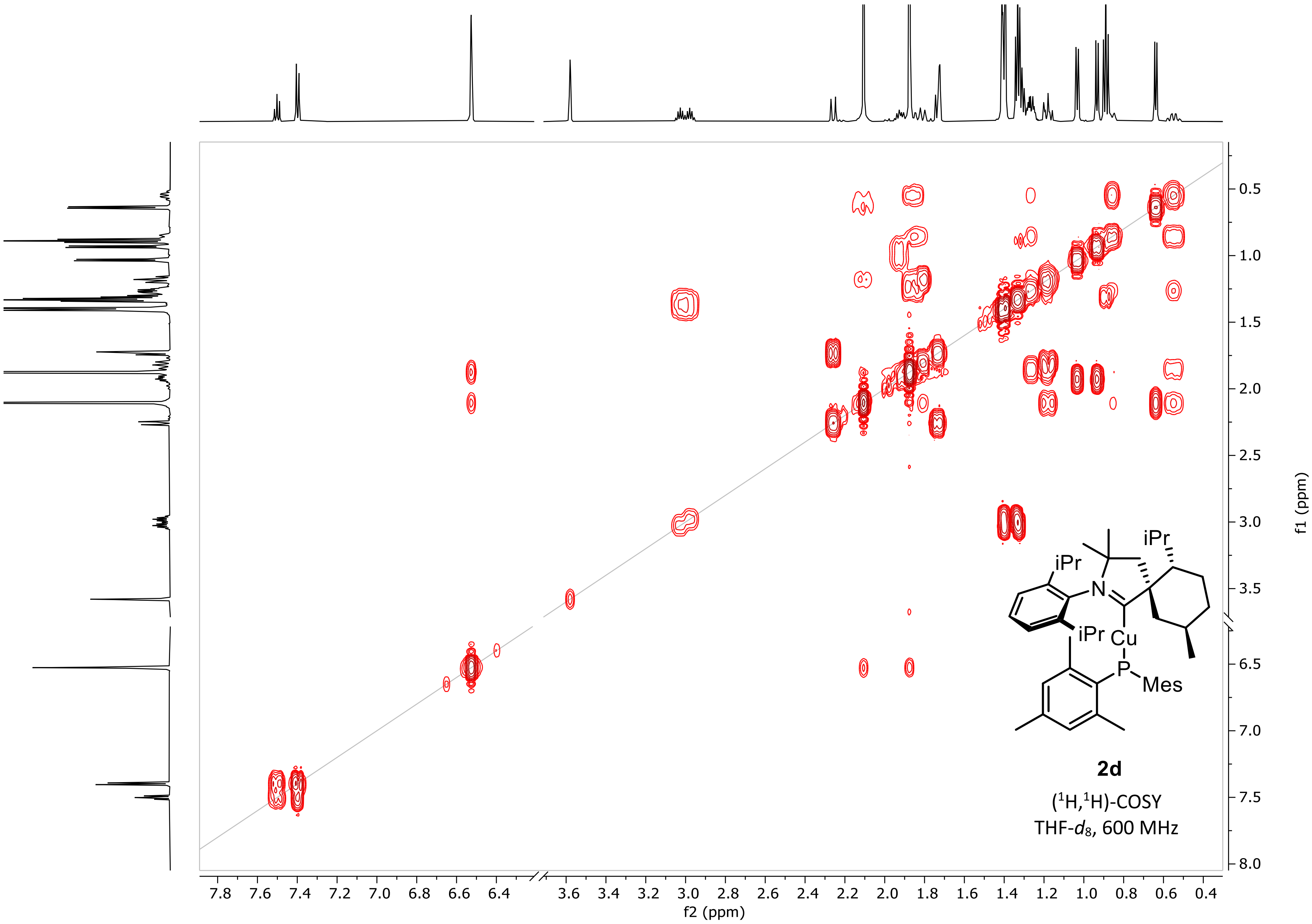

Figure S 74: (^{1}H,^{1}H)-COSY NMR spectrum of [Cu(^{Ment}cAAC)(PMes$_2$)] (**2d**) (600 MHz, THF-d_8, 298 K).

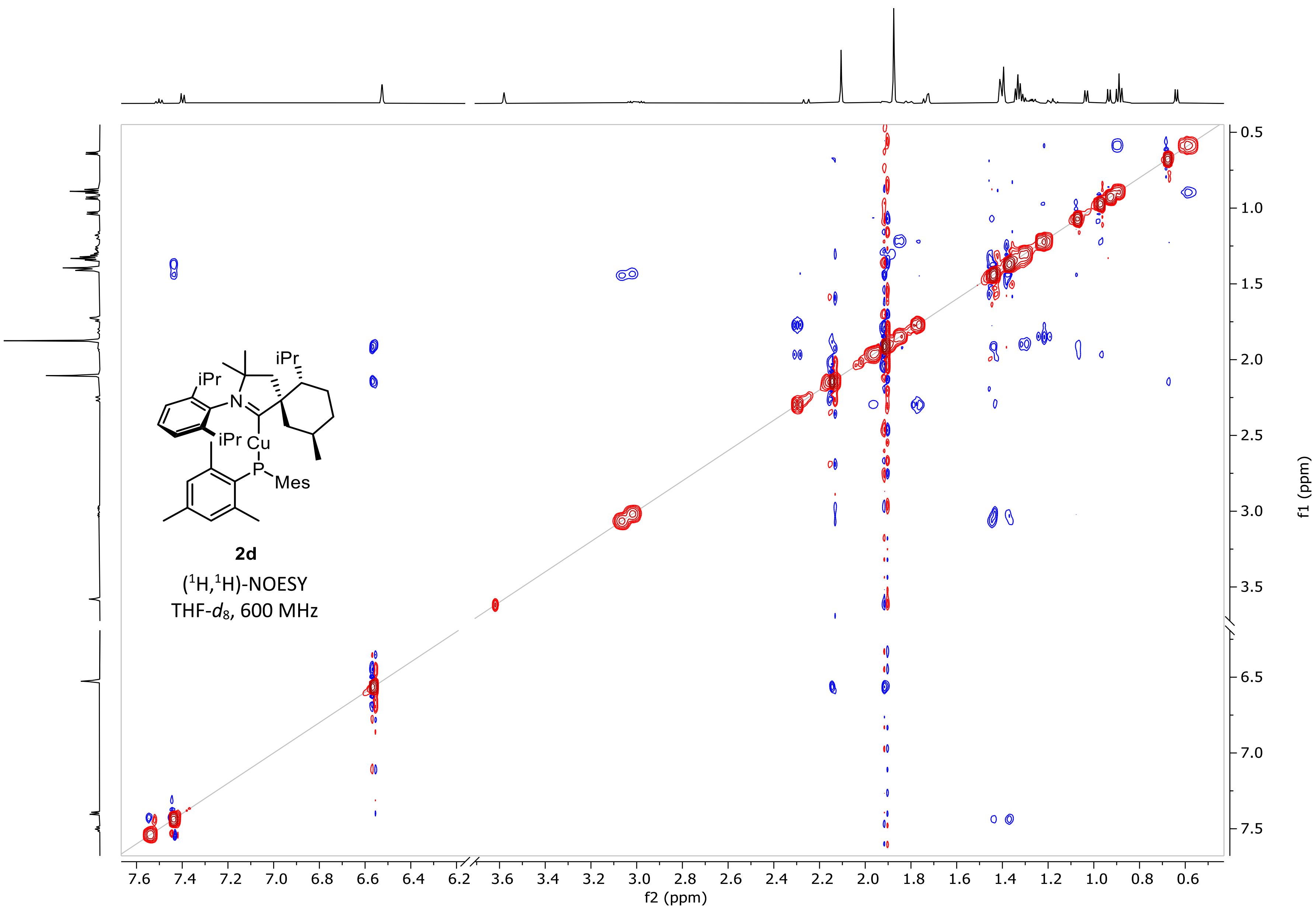

Figure S 75: (^{1}H,^{1}H)-NOESY NMR spectrum of [Cu(^{Ment}cAAC)(PMes$_2$)] (**2d**) (600 MHz, THF-d_8, 298 K).

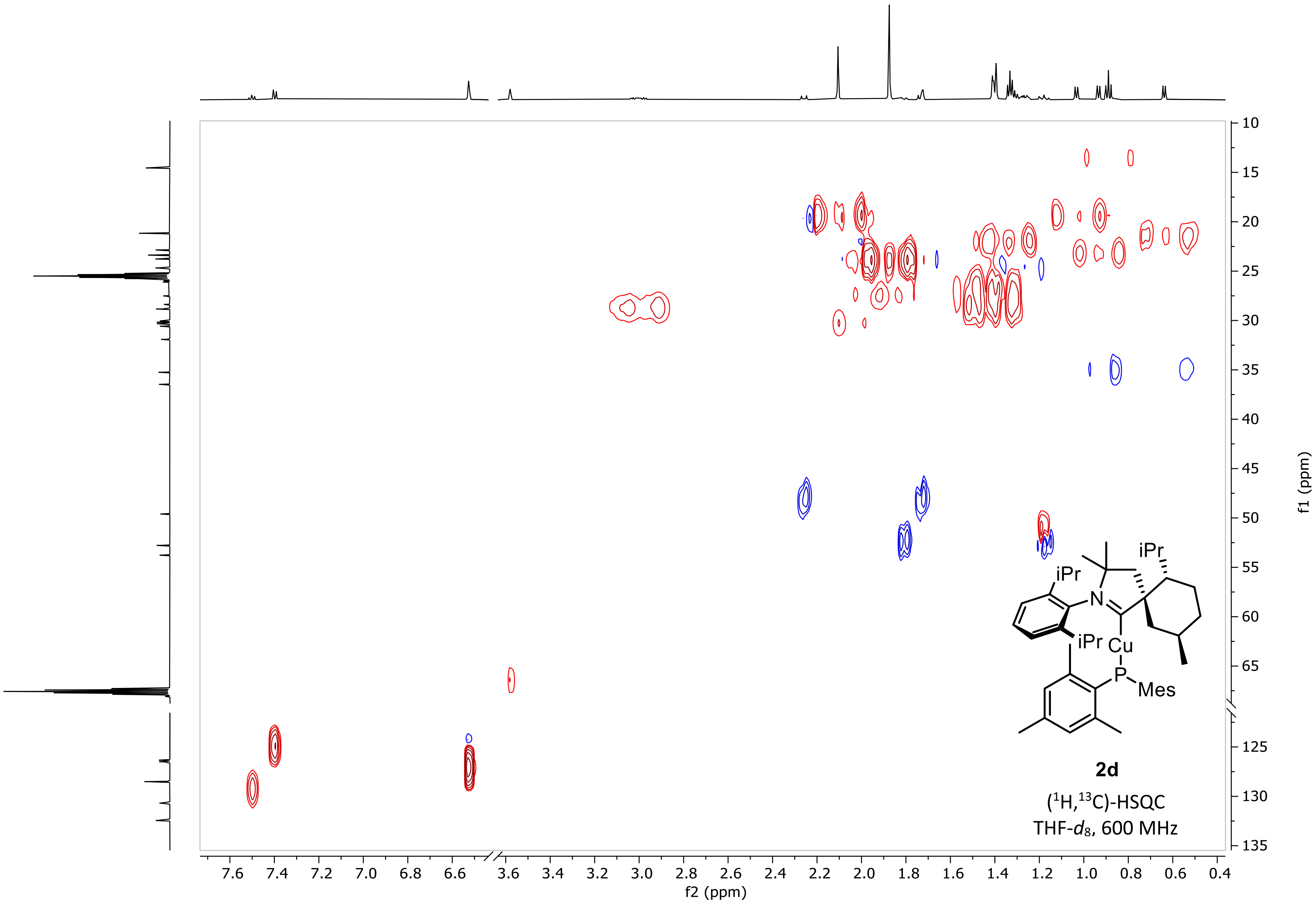

Figure S 76: (^{1}H,^{13}C)-HSCQ NMR spectrum of [Cu(^{Ment}cAAC)(PMes$_2$)] (**2d**) (600 MHz, 151 MHz, THF-d_8, 298 K).

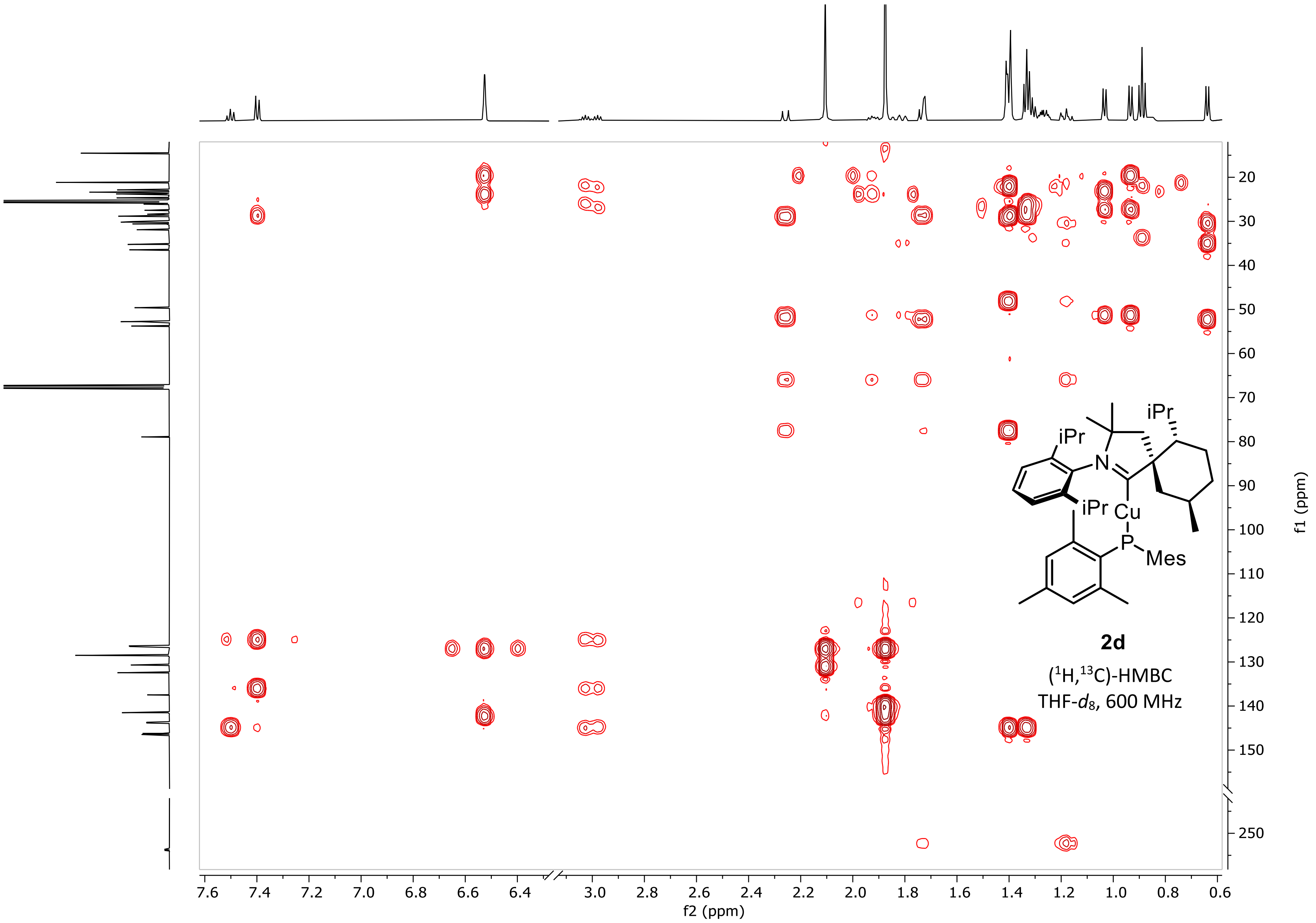

Figure S 77: (^{1}H,^{13}C)-HMBC NMR spectrum of [Cu(^{Ment}cAAC)(PMes$_2$)] (**2d**) (600 MHz, 151 MHz, THF-d_8, 298 K).

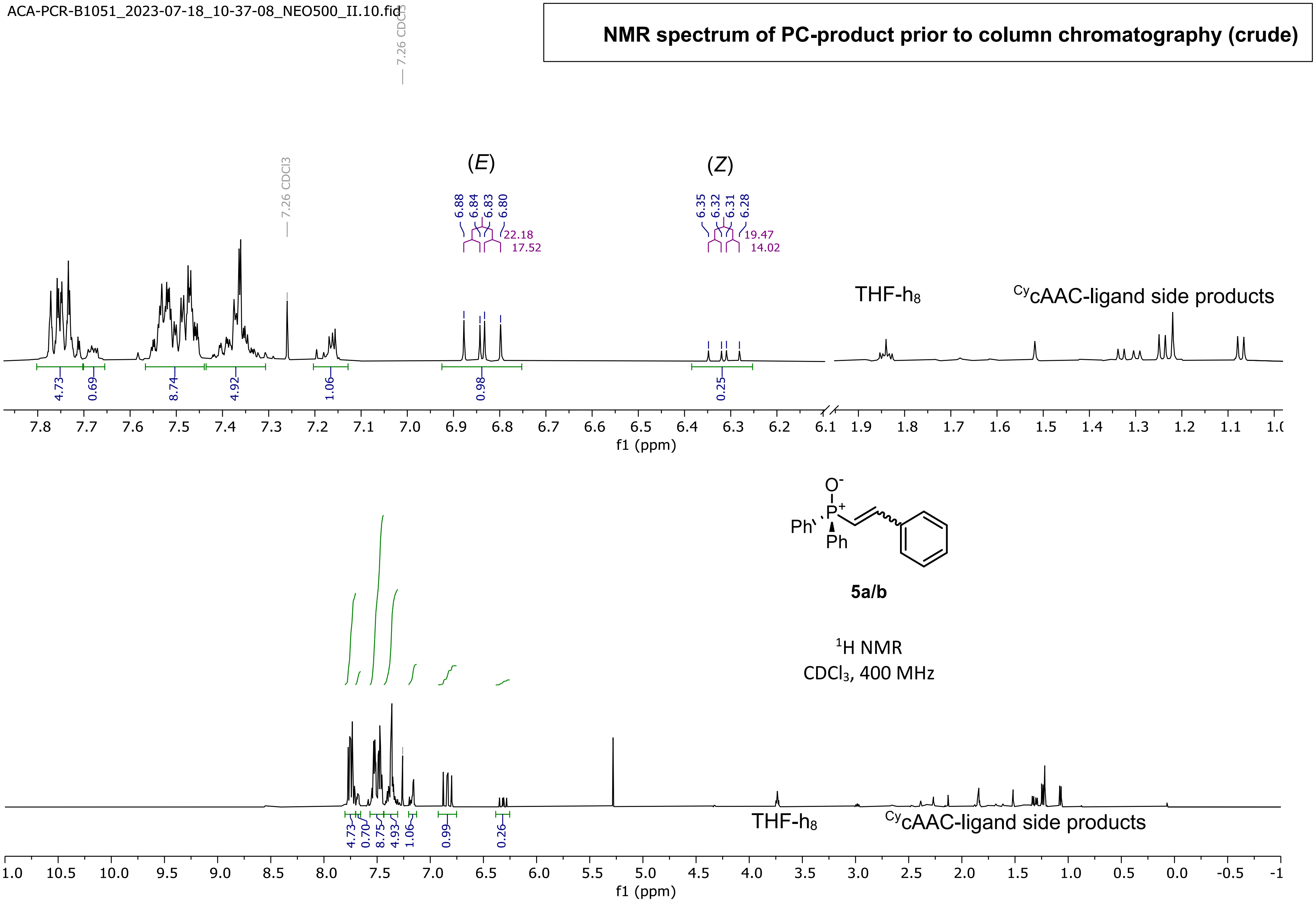

Figure S 78: ^{1}H NMR spectrum of crude **7a/b** from **1c** (400 MHz, $CDCl_3$, 298 K).

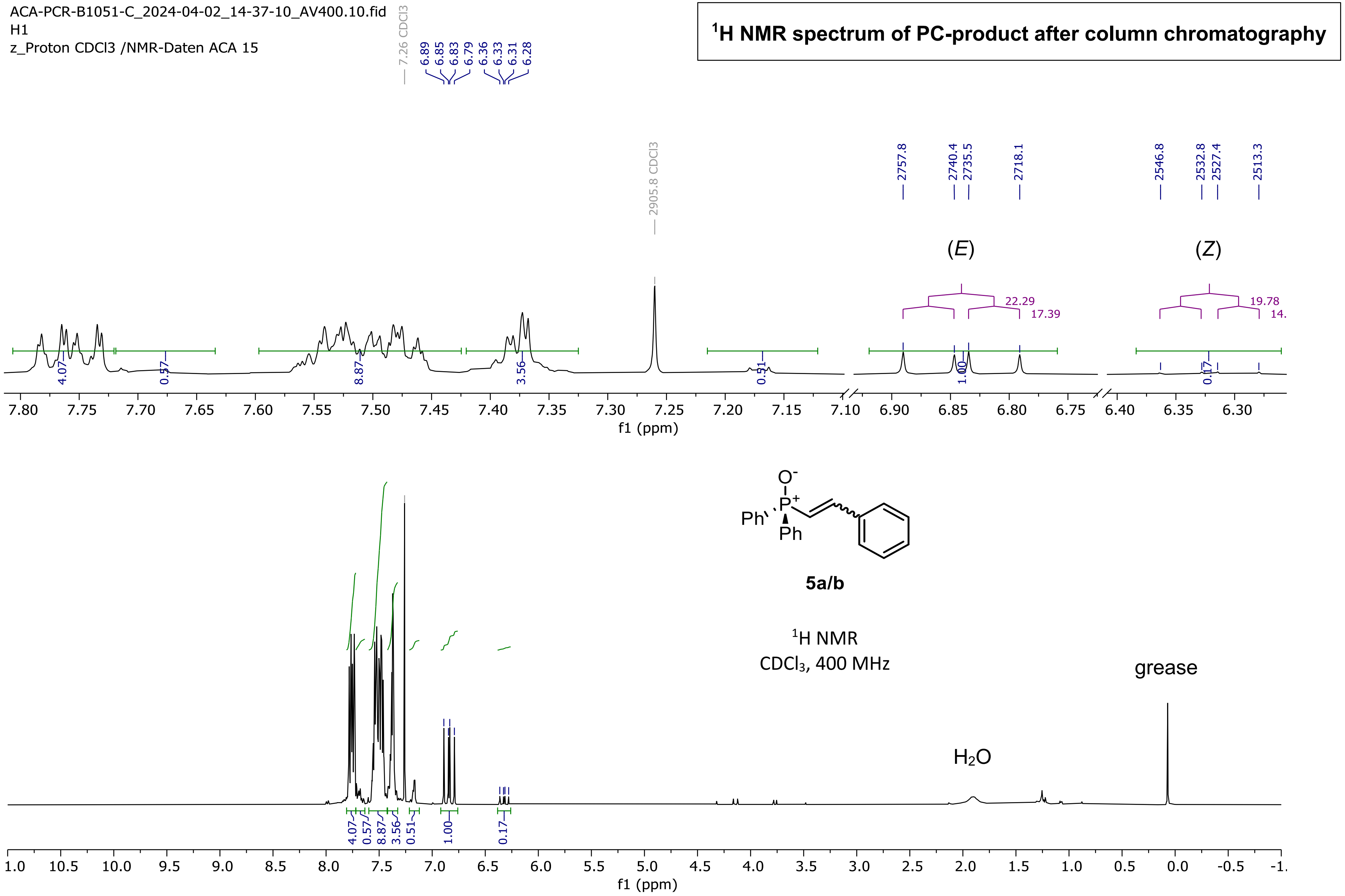

Figure S 79: ^{1}H NMR spectrum of purified **7a/b** from **1c** (400 MHz, $CDCl_3$, 298 K).

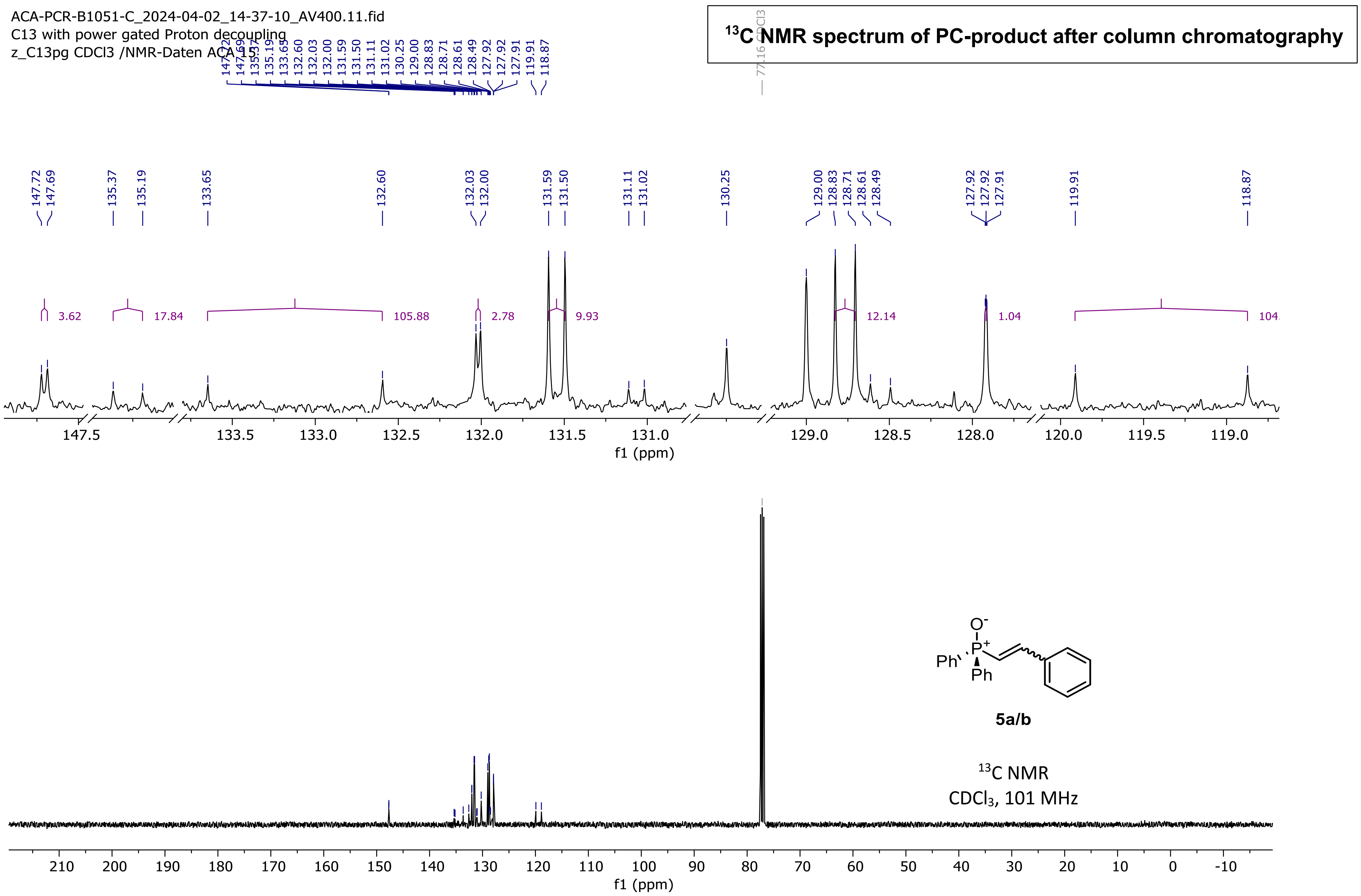

Figure S 80: {^{1}H}^{13}C NMR spectrum of purified **7a/b** from **1c** (101 MHz, $CDCl_3$, 298 K).

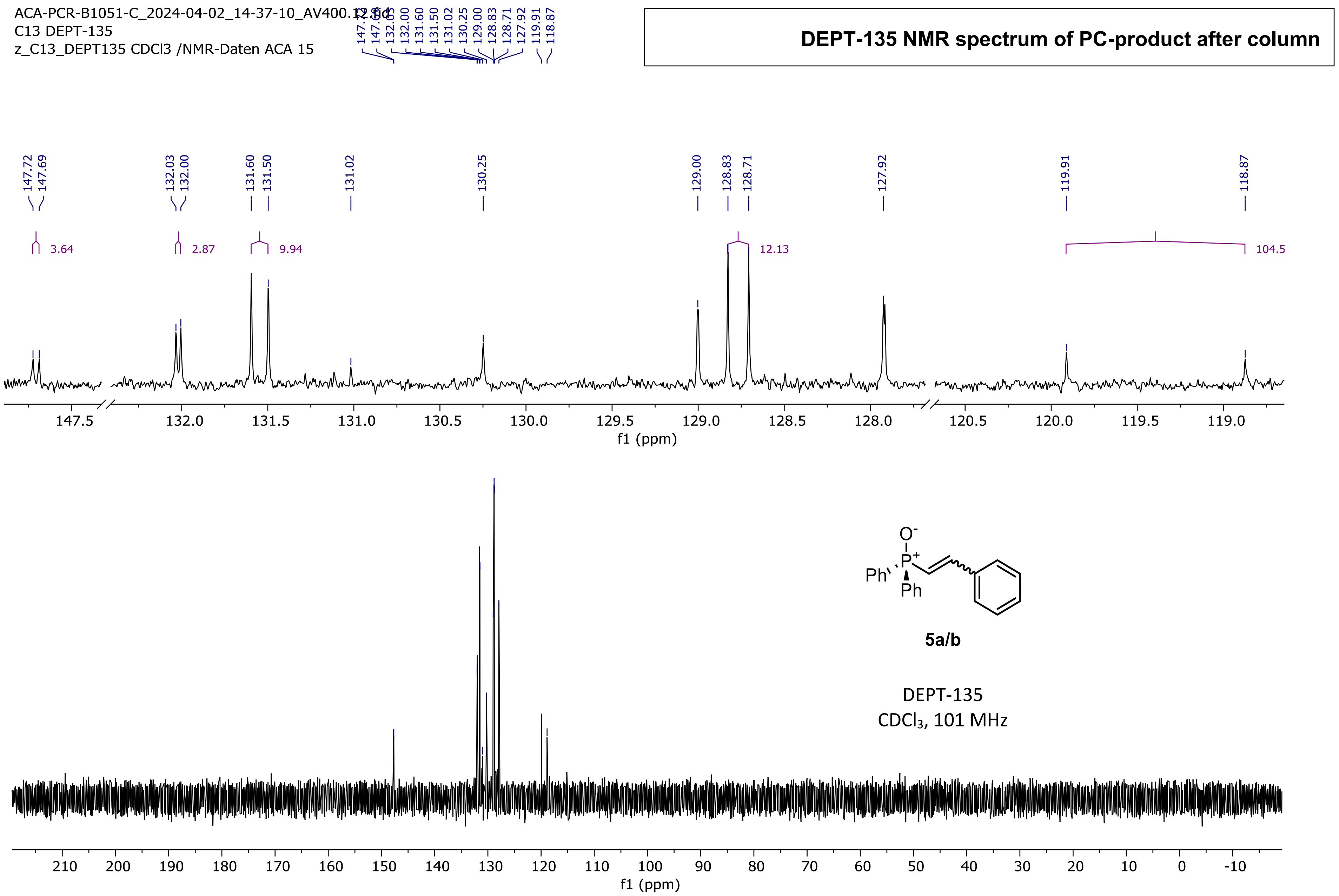

Figure S 81: DEPT-135 NMR spectrum of purified **7a/b** from **1c** (101 MHz, $CDCl_3$, 298 K).

ACA-PCR-B1051-C_2024-04-02-15-37-10_AV500.10.fid

^{31}P NMR spectrum of PC-product after column chromatography

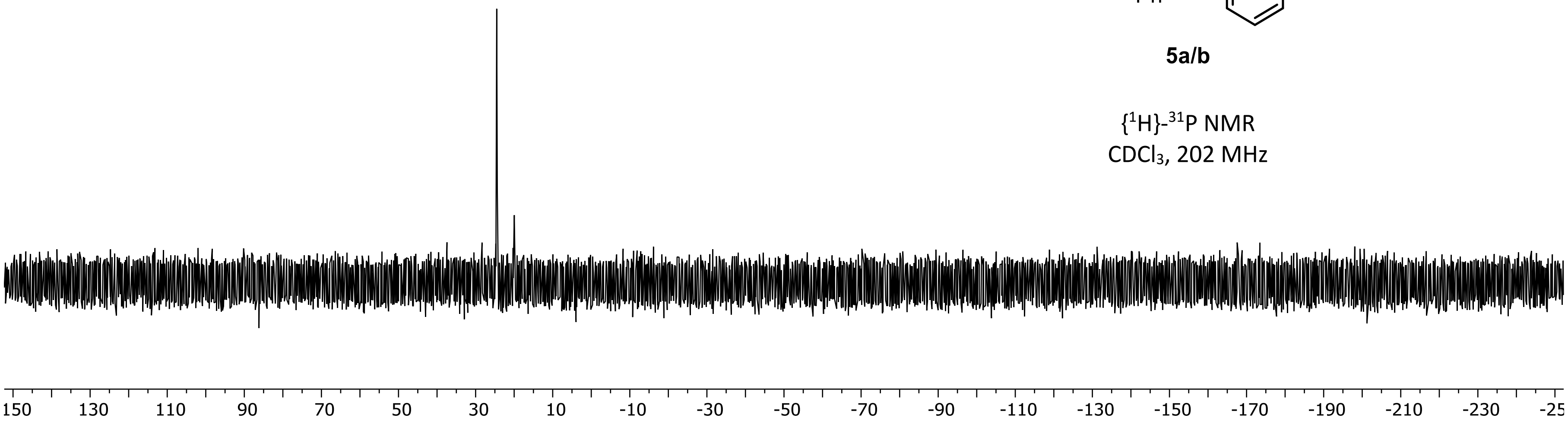

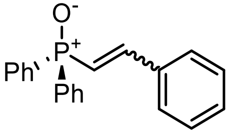

5a/b

{^{1}H}-^{31}P NMR
$CDCl_3$, 202 MHz

Figure S 82: {^{1}H}^{31}P NMR spectrum of purified **7a/b** from **1c** (202 MHz, $CDCl_3$, 298 K).

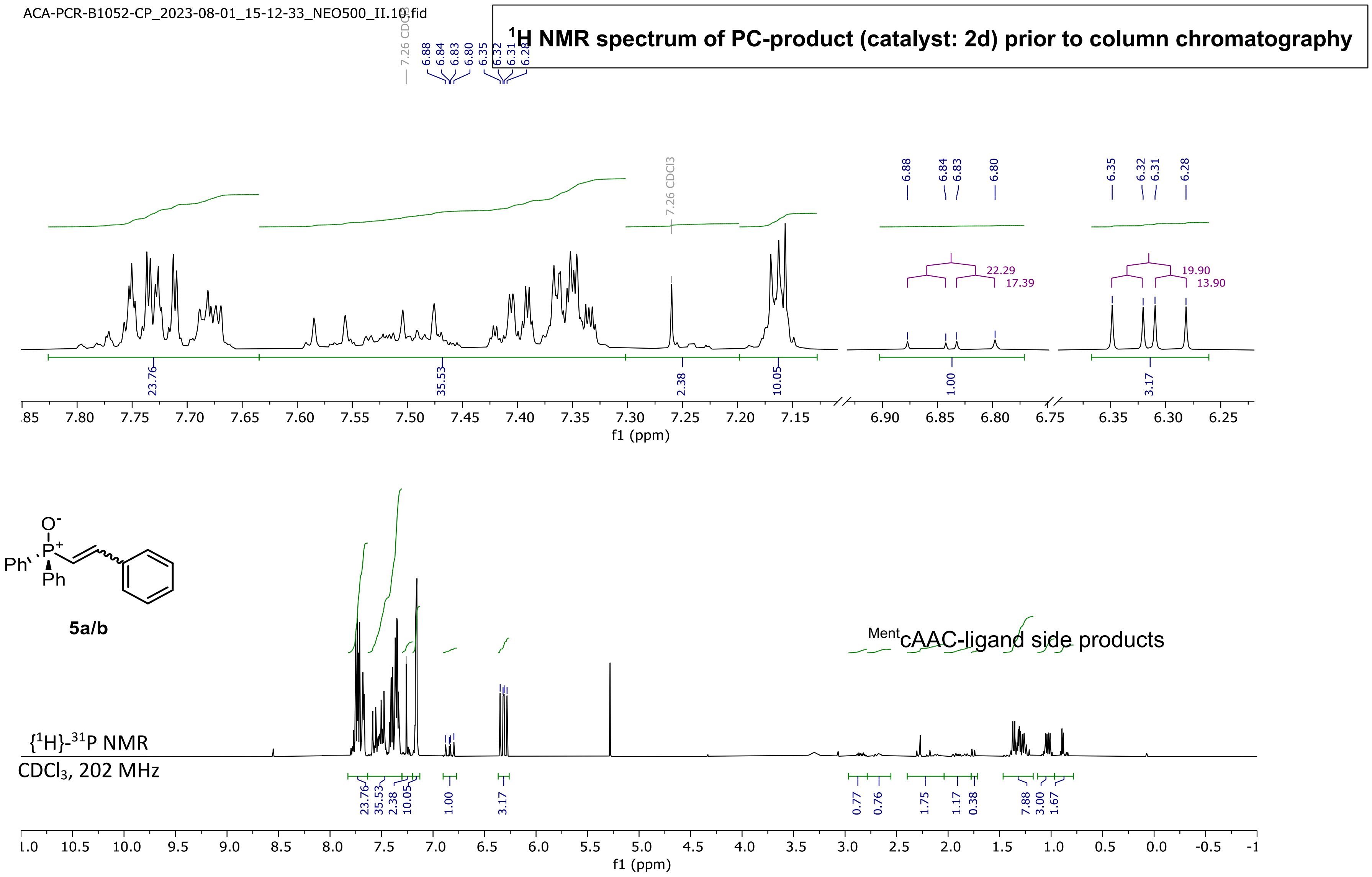

Figure S 83: ^{1}H NMR spectrum of crude **7a/b** from **1d** (101 MHz, $CDCl_3$, 298 K).

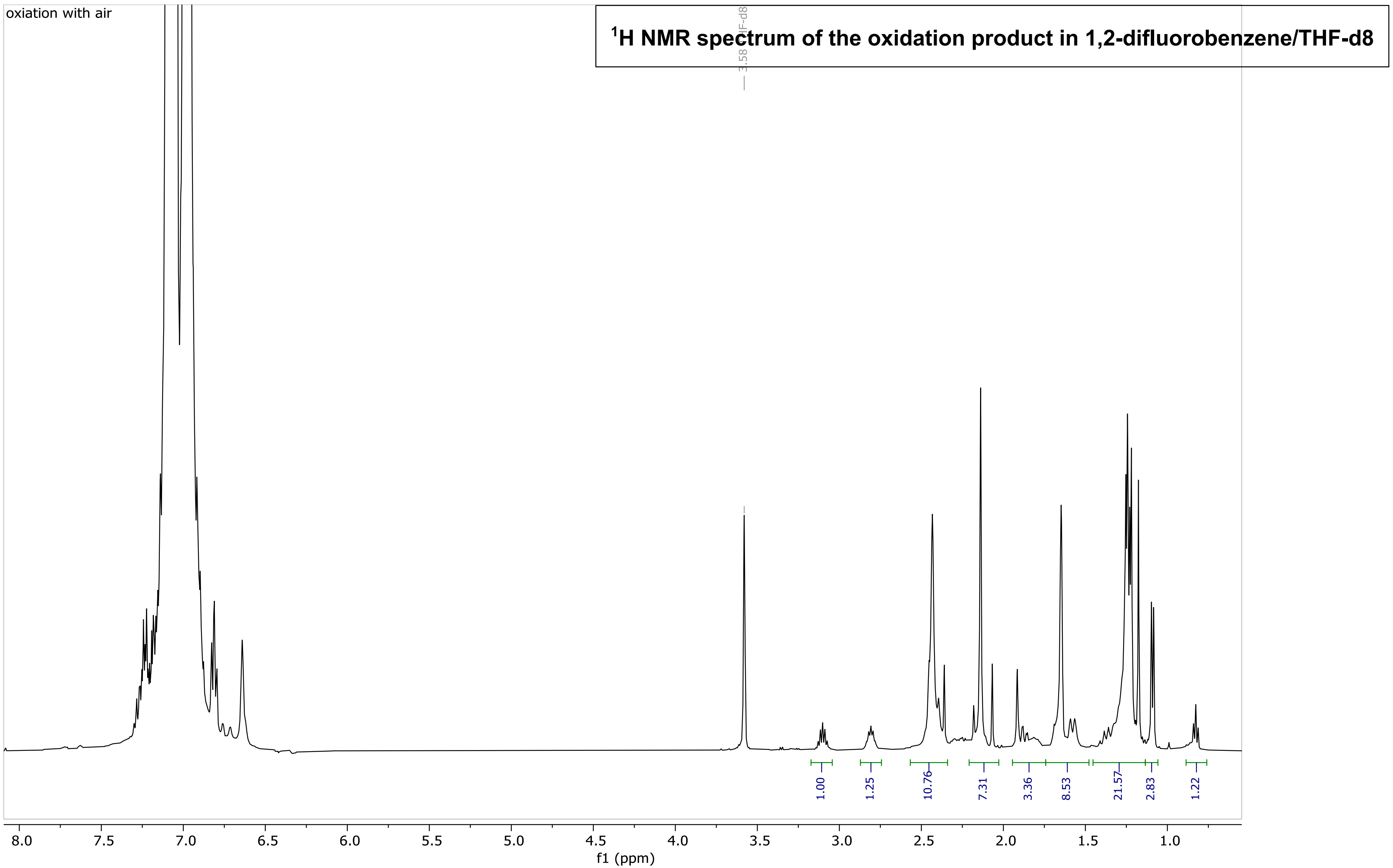

Figure S 84: ^{1}H NMR spectrum of the oxidation product in 1,2-difluorobenzene/THF-d8 (second run).

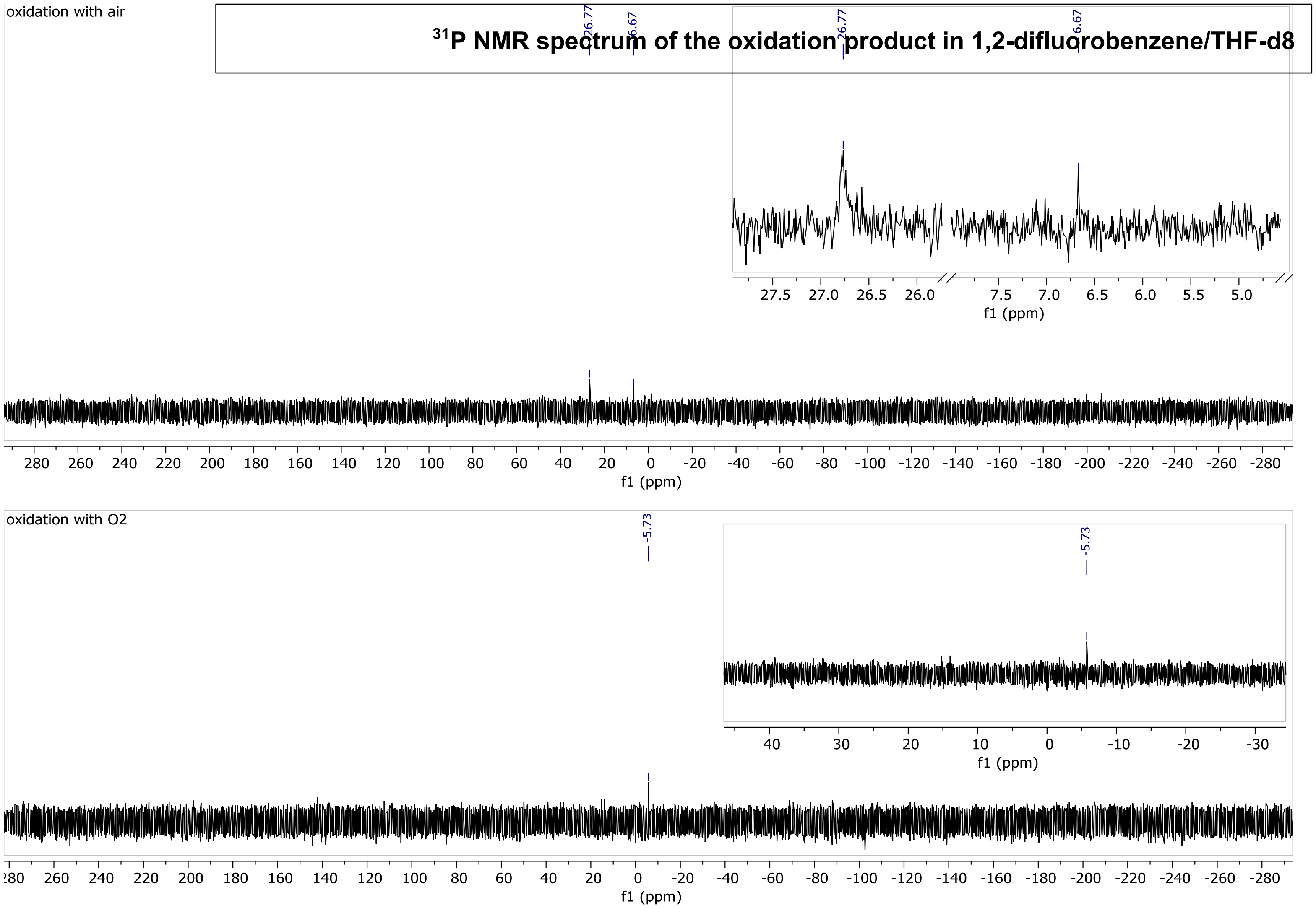

Figure S 85: {^{1}H}^{31}P NMR spectrum spectrum of the oxidation product in 1,2-difluorobenzene/THF-d8 (second run).

4. MIR-absorption-spectra

MIR-Spectra of complexes 2a – 2d

2a [Cu(^{Me}cAAC)(PMes$_2$)]

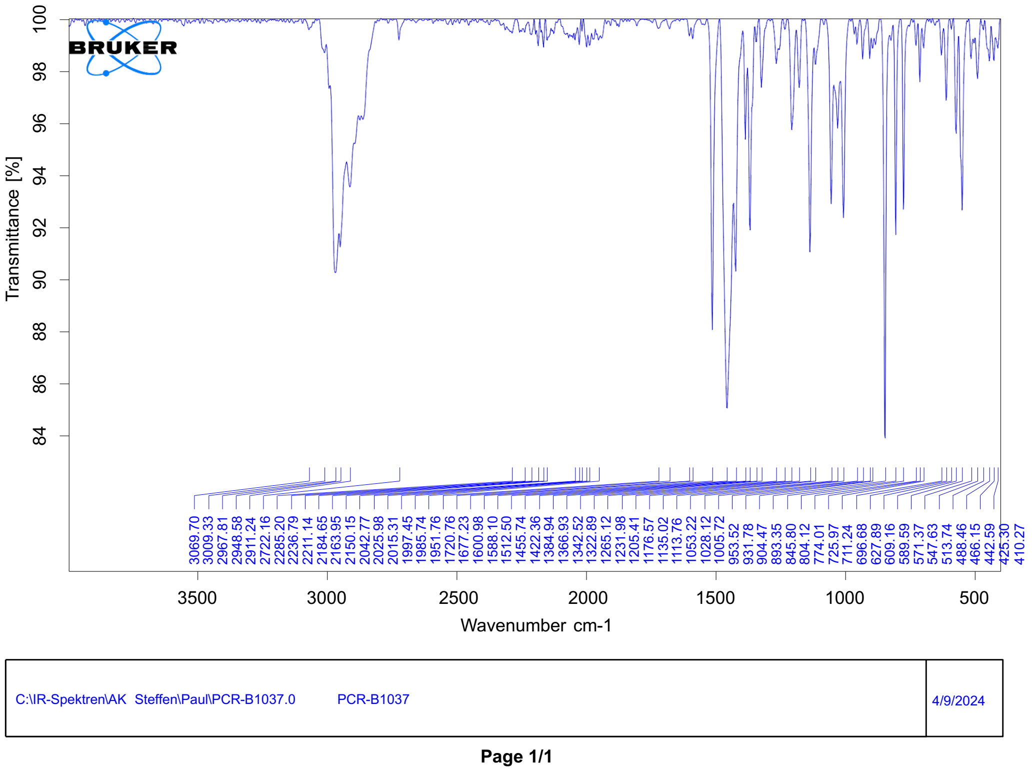

Figure S 86: MIR-Absorption spectrum (ATR) of [Cu(^{Me}cAAC)(PMes$_2$)] (**2a**) (neat, 298 K)

2b [Cu(EtcAAC)(PMes2)]

C:\IR-Spektren\AK Steffen\Paul\ACA-PCR-B1049.0

7/12/2023 11:47:08 AM

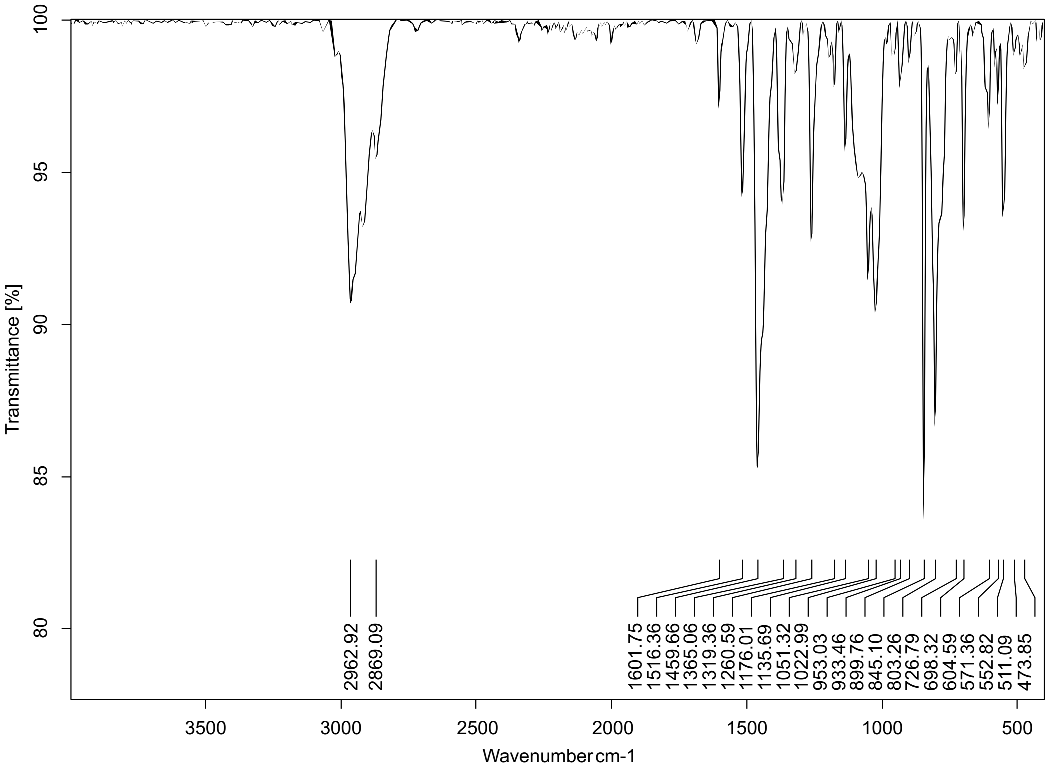

Page 1 of 1

Figure S 87: MIR-Absorption spectrum (ATR) of [Cu(^{Et}cAAC)(PMes$_2$)] (**2b**) (neat, 298 K)

2c [Cu(^{Cy}cAAC)(PMes$_2$)]

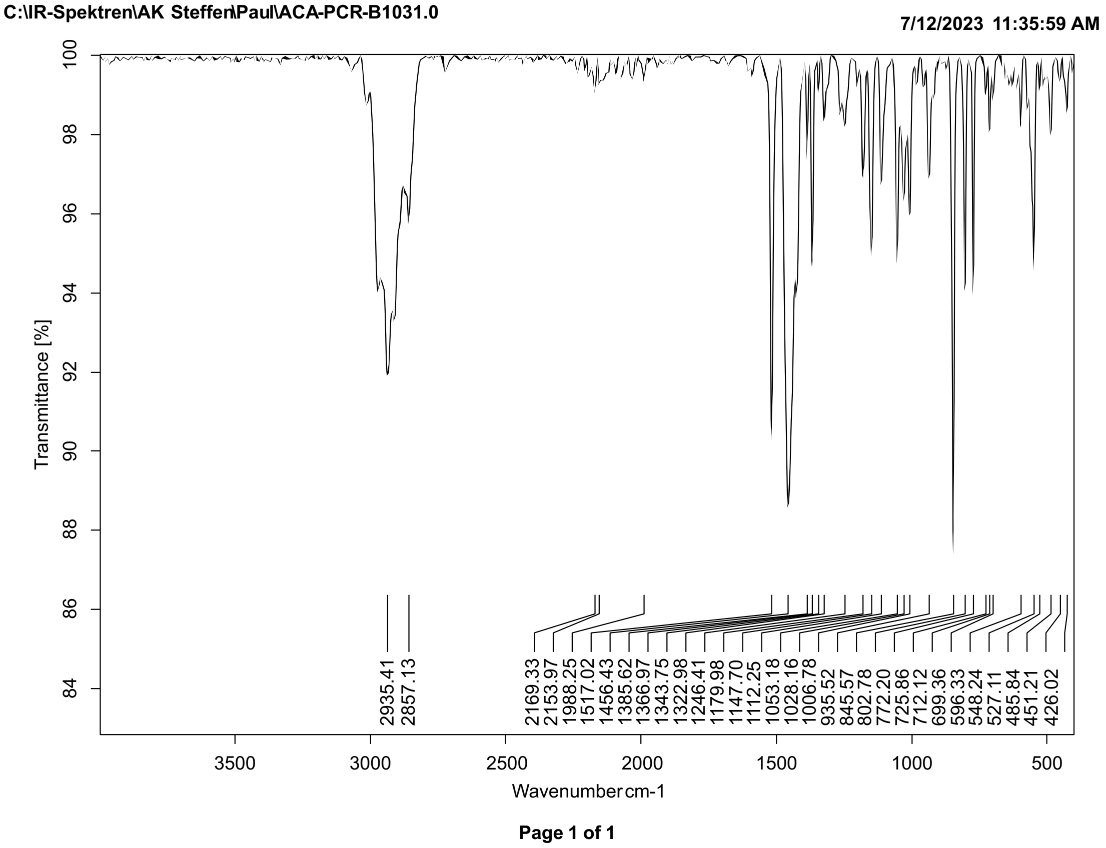

Figure S 88: MIR-Absorption spectrum (ATR) of [Cu(^{Cy}cAAC)(PMes$_2$)] (**2c**) (neat, 298 K)

2d [Cu(^{Ment}cAAC)(PMes$_2$)]

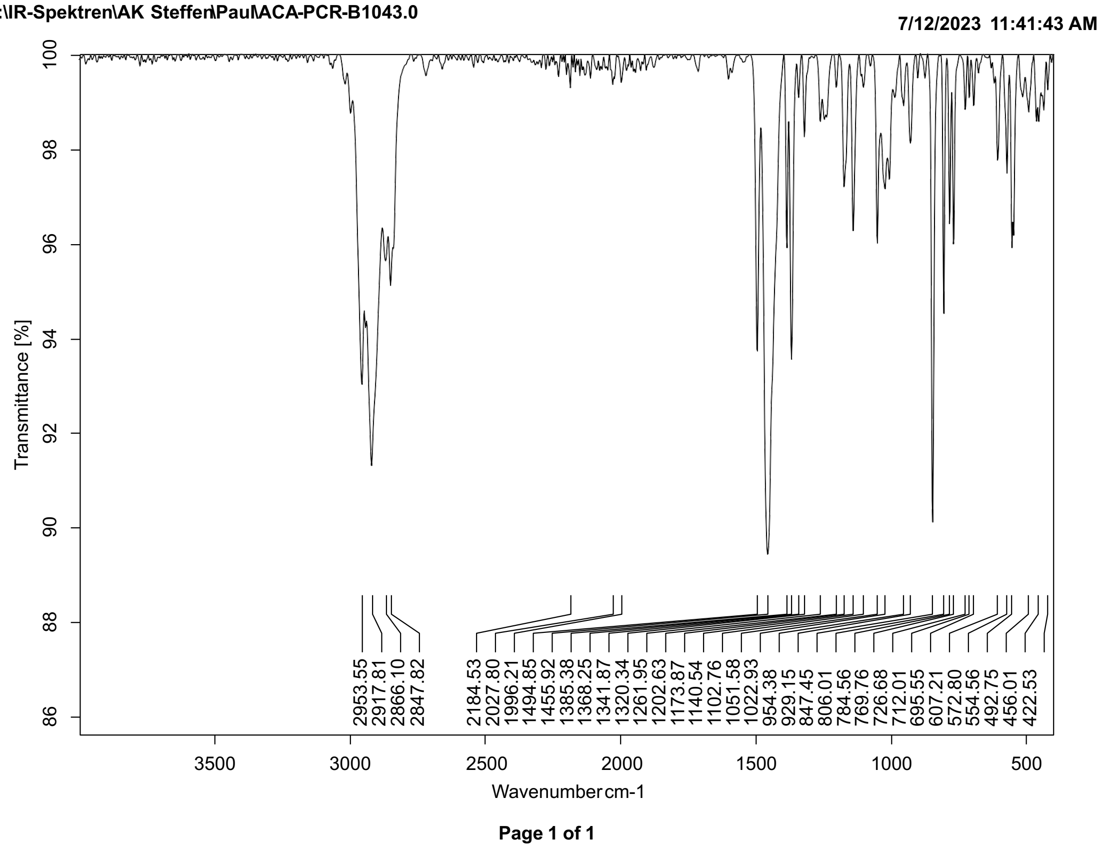

Figure S 89: MIR-Absorption spectrum (ATR) of [Cu(^{Ment}cAAC)(PMes$_2$)] (**2d**) (neat, 298 K)

MIR-Spectrum of Photocatalysis product 5a

Analyst	AK Steffen
Date	Friday, 30 August 2024 10:08

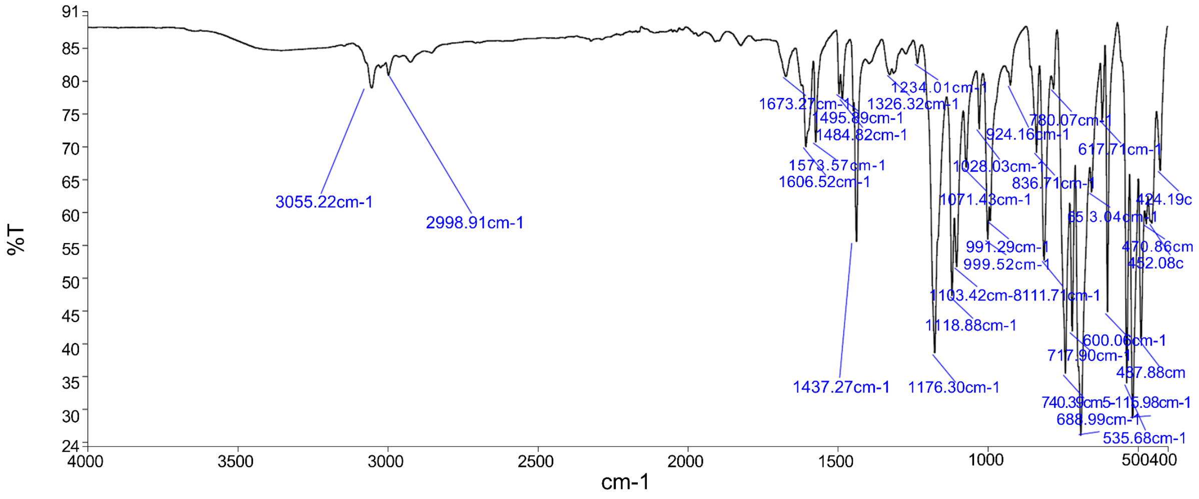

		Quality Checks
ACA-PCR-B1051-1_1	Sample 139 By AK Steffen Date Monday, August 26 2024	The Quality Checks do not report any warnings for the sample.

Figure S 90: MIR-Absorption spectrum (ATR) of **7a/b** (neat, 298 K)

5. HR-Mass-Spectra

HRMS of Complexes 2a – 2d

2a [Cu(^{Me}cAAC)(PMes$_2$)]

Total spectrum

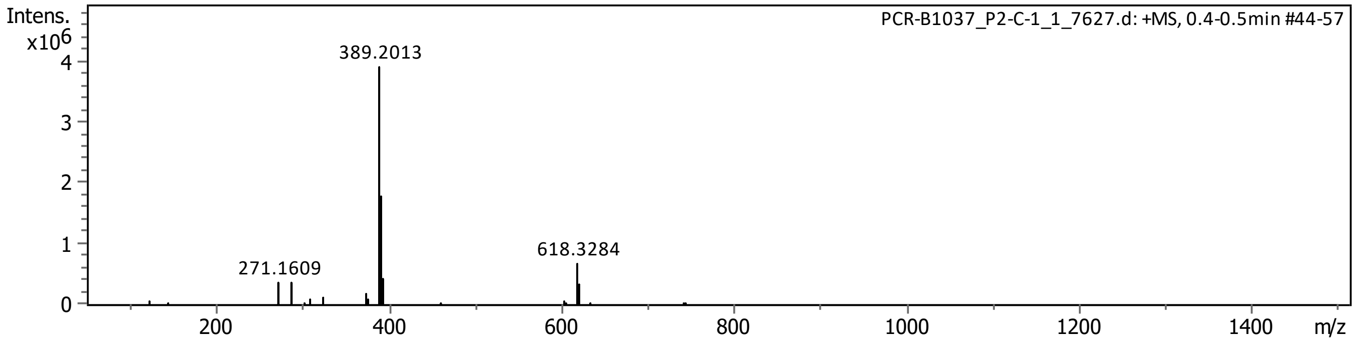

Zoom

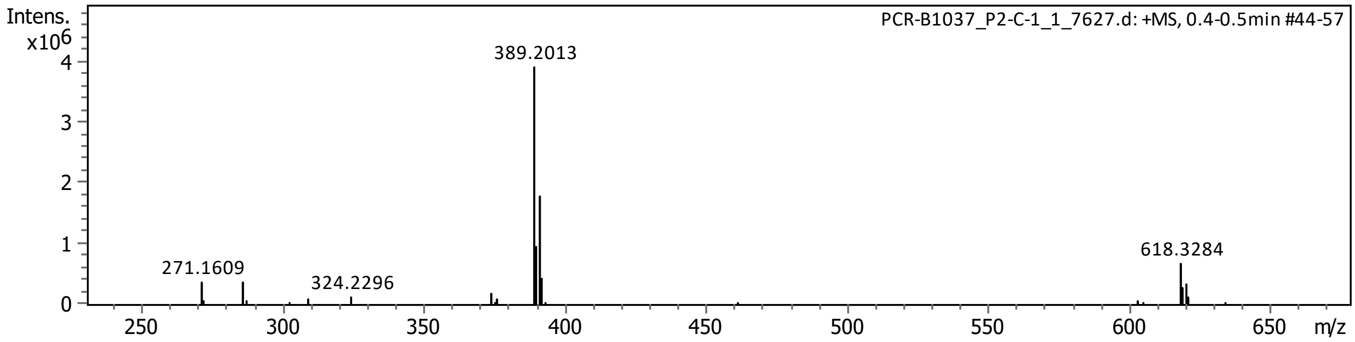

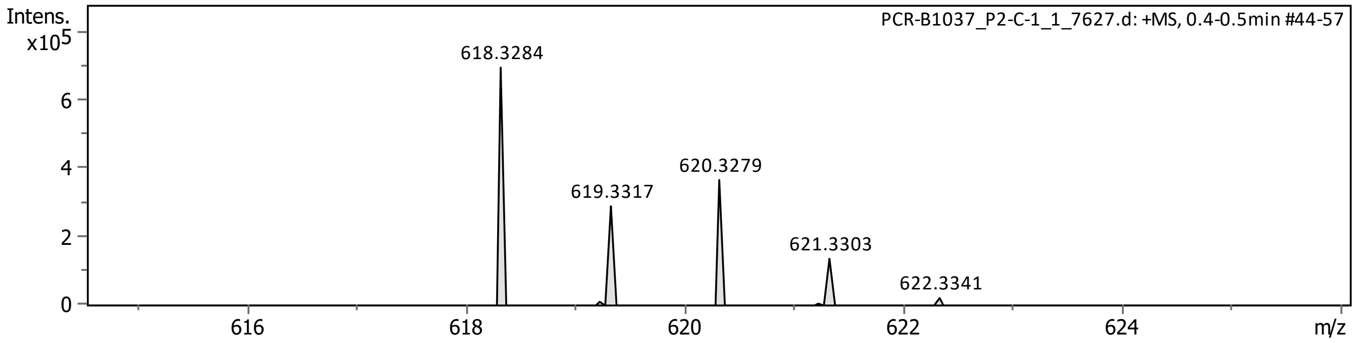

[M+H]$^+$: 0,0 ppm

Figure S 91: ESI-HRMS of [Cu(^{Me}cAAC)(PMes$_2$)] (**2a**) (THF)

2b [Cu(^{Et}cAAC)(PMes$_2$)]

Total spectrum

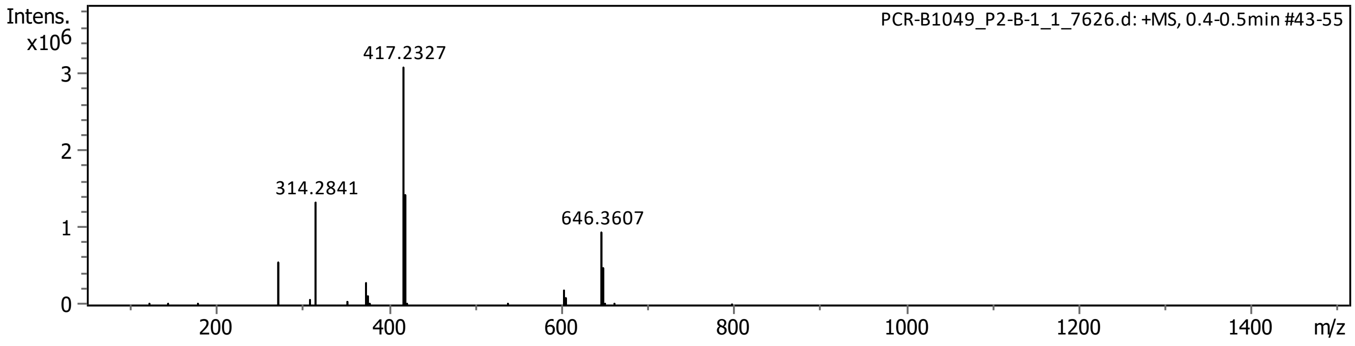

Zoom

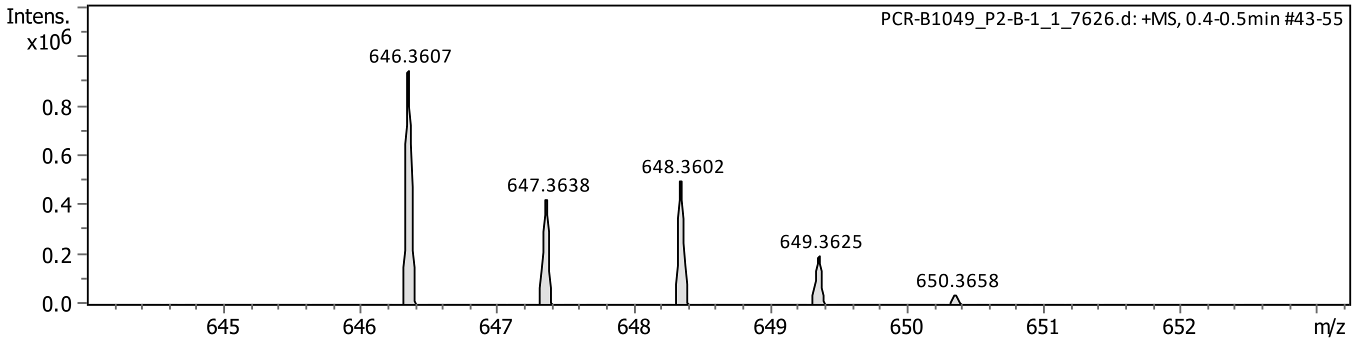

$[M+H]^+$ –1,4 ppm

Figure S 92: ESI-HRMS of [Cu(^{Et}cAAC)(PMes$_2$)] (**2b**) (THF)

2c [Cu(^{Cy}cAAC)(PMes$_2$)]

Total Spectrum

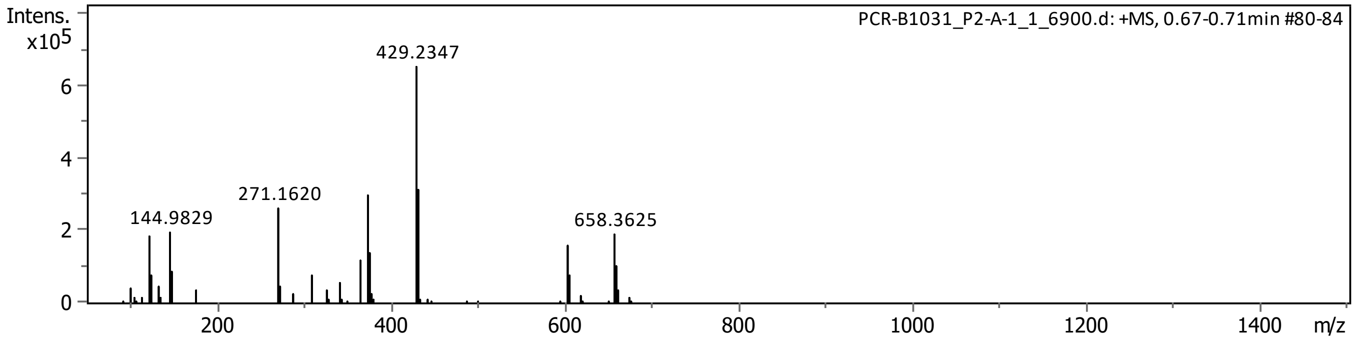

Zoom

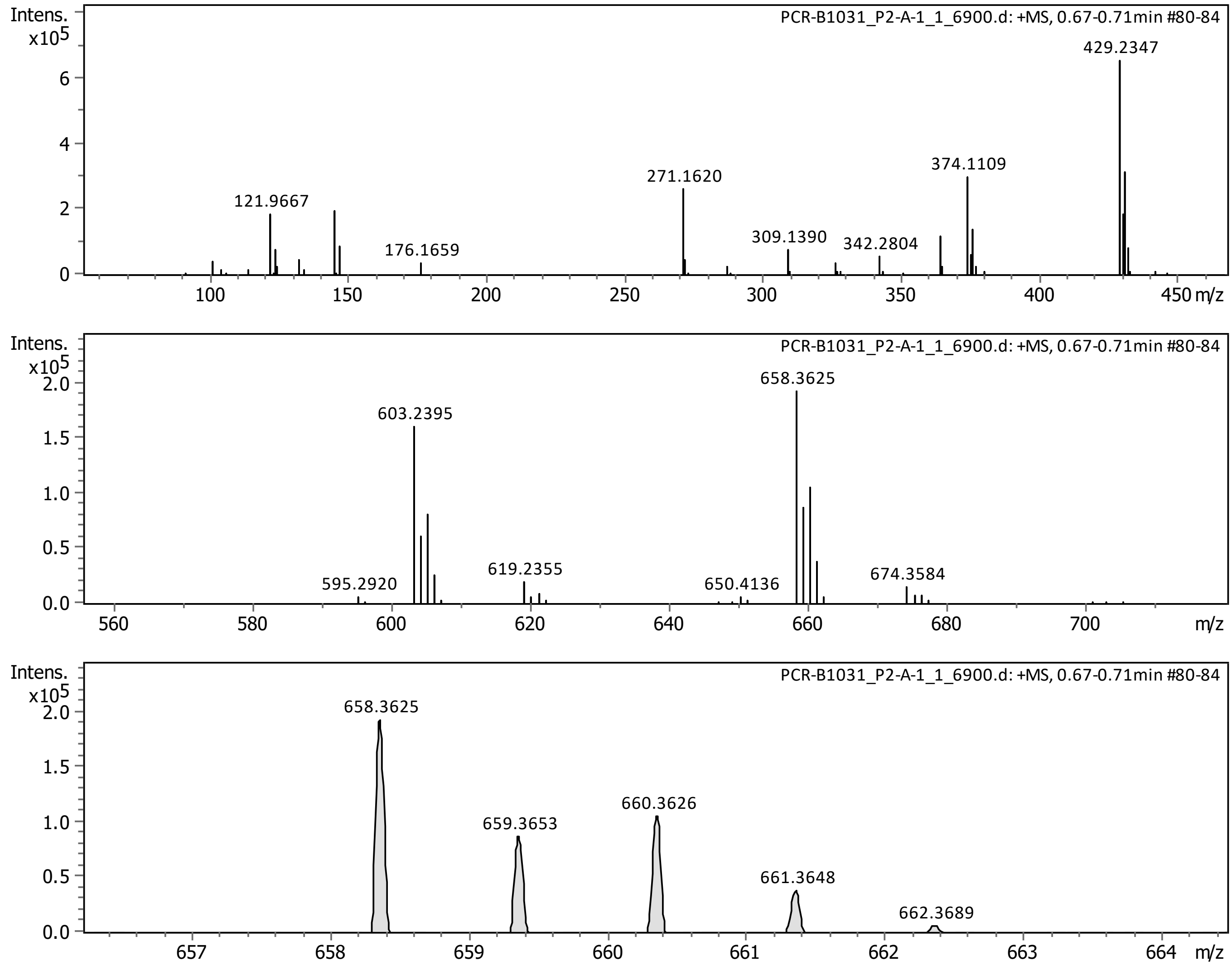

[M+H]$^{+}$ –4,2 ppm

Figure S 93: ESI-HRMS of [Cu(^{Cy}cAAC)($PMes_2$)] (**2c**) (THF)

2d [Cu(^{Ment}CAAC)($PMes_2$)]

Total spectrum:

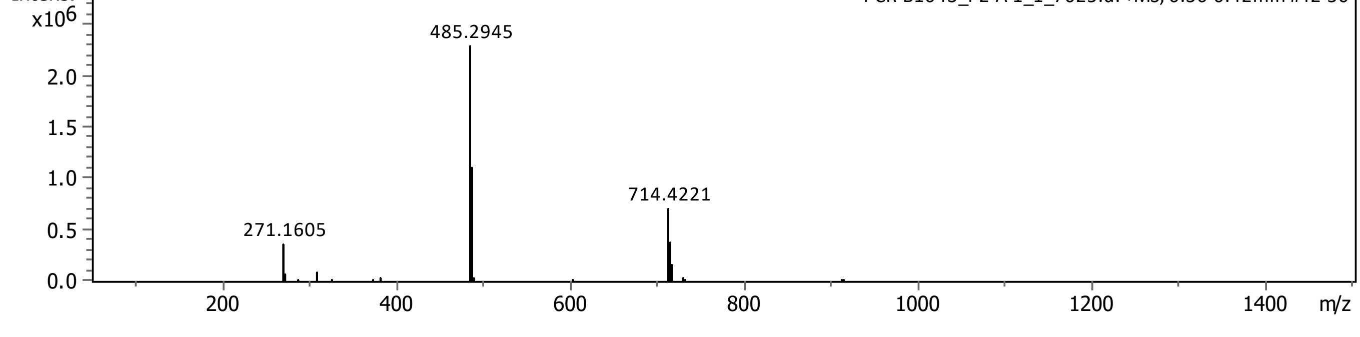

Zoom :

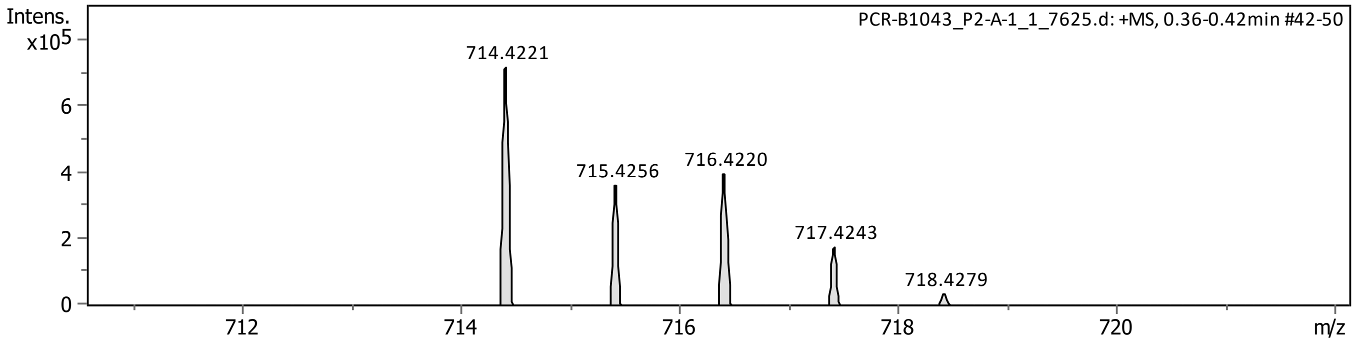

$[M+H]^+$ +0,3 ppm

Figure S 94: ESI-HRMS of [Cu(^{Ment}cAAC)($PMes_2$)] (**2c**) (THF)

HR-mass spectra of the oxidation product

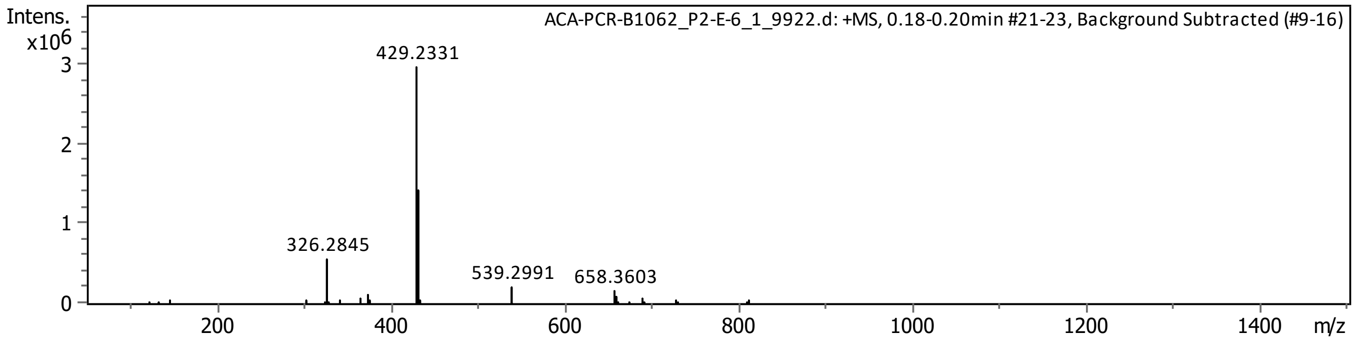

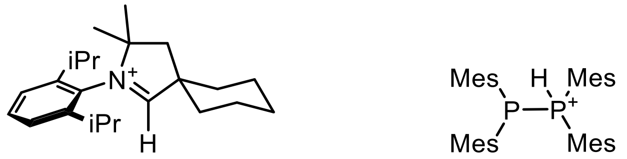

Chemical Formula: $C_{23}H_{36}N^+$
Exact Mass: 326,2842
Molecular Weight: 326,5475

Chemical Formula: $C_{36}H_{45}P_2^+$
Exact Mass: 539,2991
Molecular Weight: 539,7030

Vergrößerung:

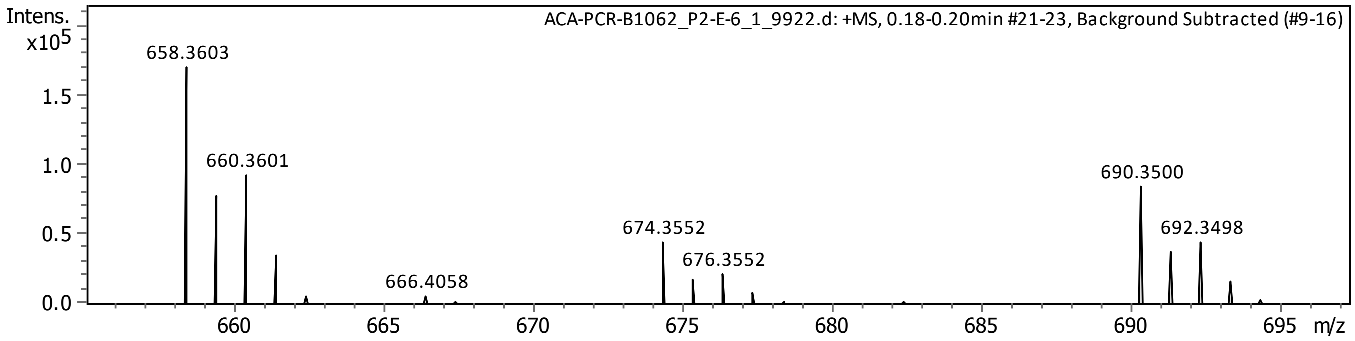

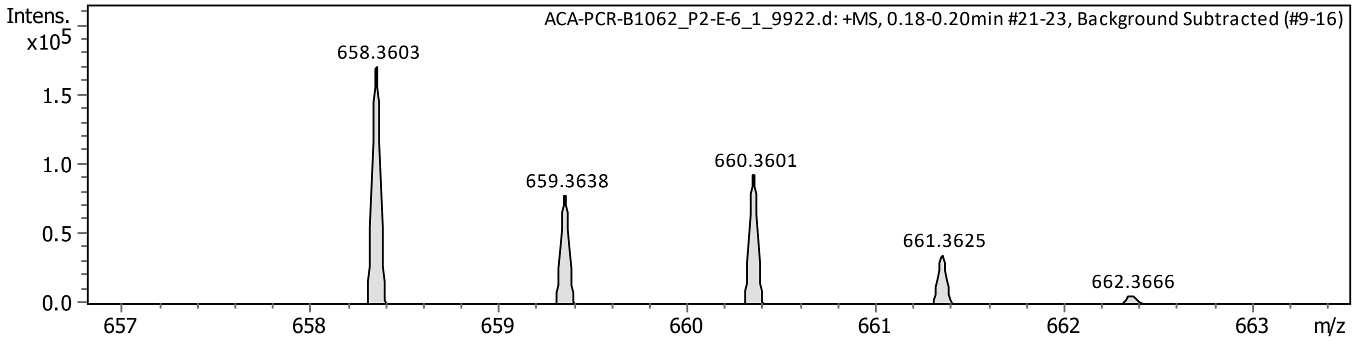

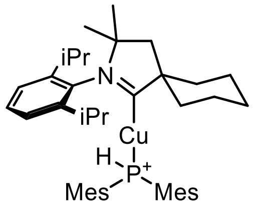

Chemical Formula: $C_{41}H_{58}CuNP^{+}$
Exact Mass: 658,3597
Molecular Weight: 659,4412

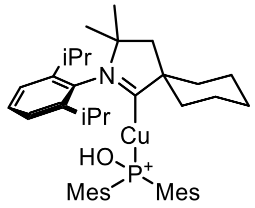

Chemical Formula: $C_{41}H_{58}CuNOP^{+}$
Exact Mass: 674,3547
Molecular Weight: 675,4402

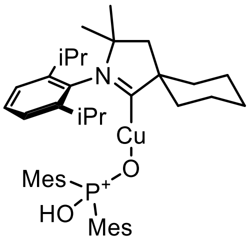

Chemical Formula: $C_{41}H_{58}CuNO_2P^{+}$
Exact Mass: 690,3496
Molecular Weight: 691,4392

$[M+H]^{+}$ -0,9 ppm

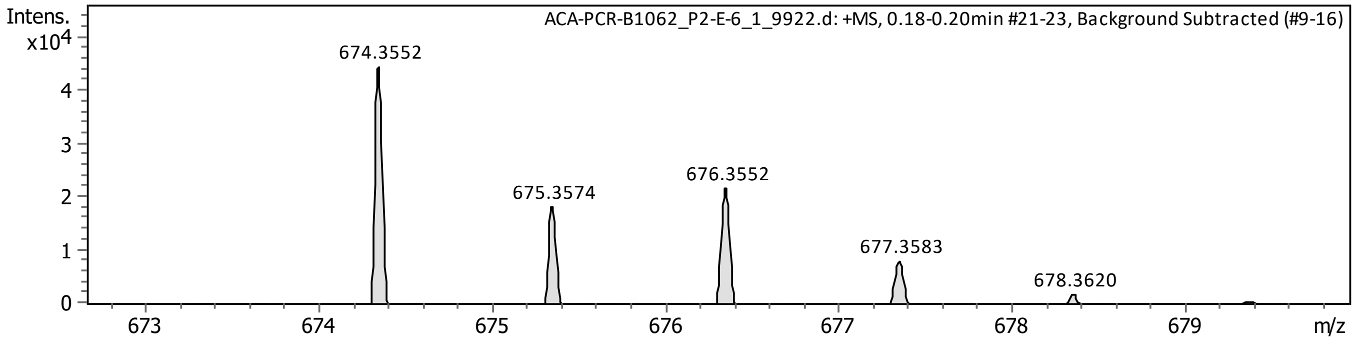

$[M+H]^+$ mit -0,9 ppm Abweichung

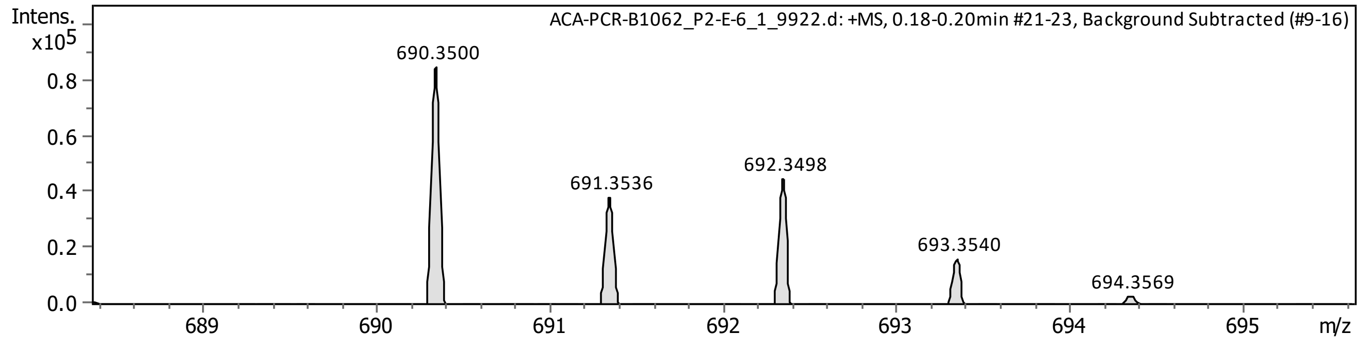

$[M+H]^+$ -0,6 ppm

Figure S 95: ESI-HRMS of the product obtained upon reaction of **2c** with dry oxygen (THF) and identified species and their exact masses.

HR-mass spectra of Photocatalysis products (PC-1, PC-2)

With [Cu(^{Cy}cAAC)Cl] (1c)

Total spectrum

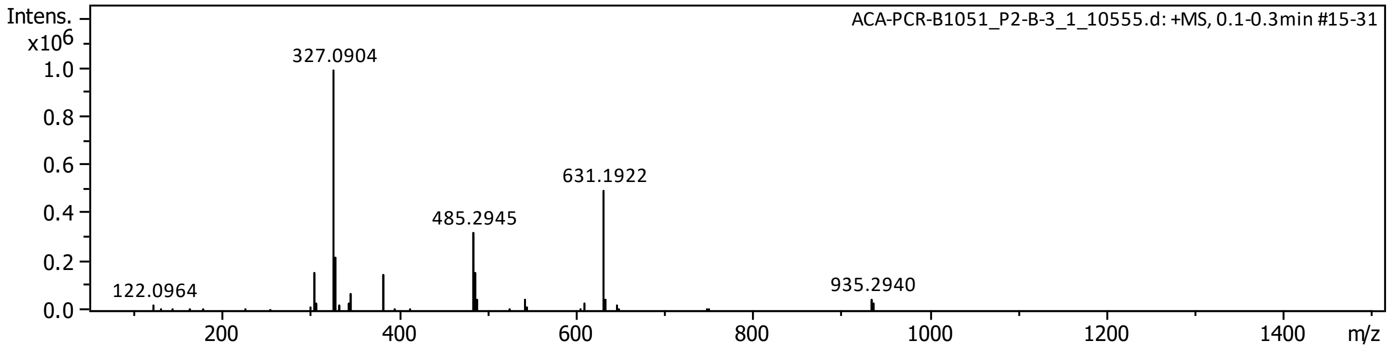

Zoom

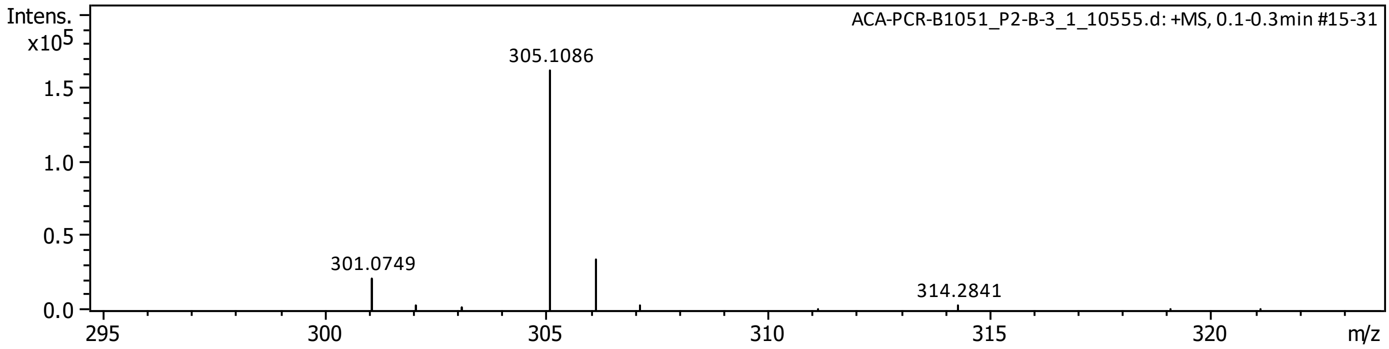

$[M+H]^+$ +1,3ppm

Figure S 96: ESI-HRMS of **7a/b** synthesized with (**1c**) (THF)

With [Cu(^{Ment}cAAC)Cl] (1d)

Total Spectrum

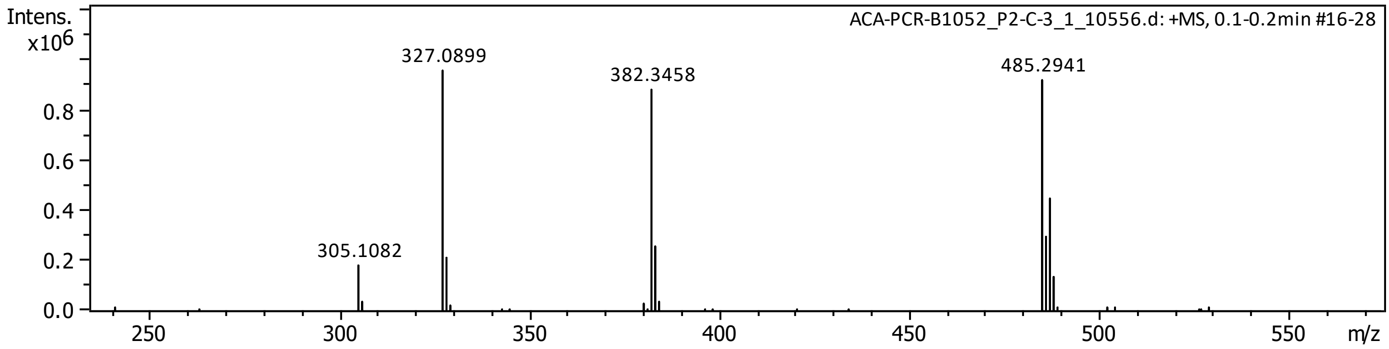

Zoom :

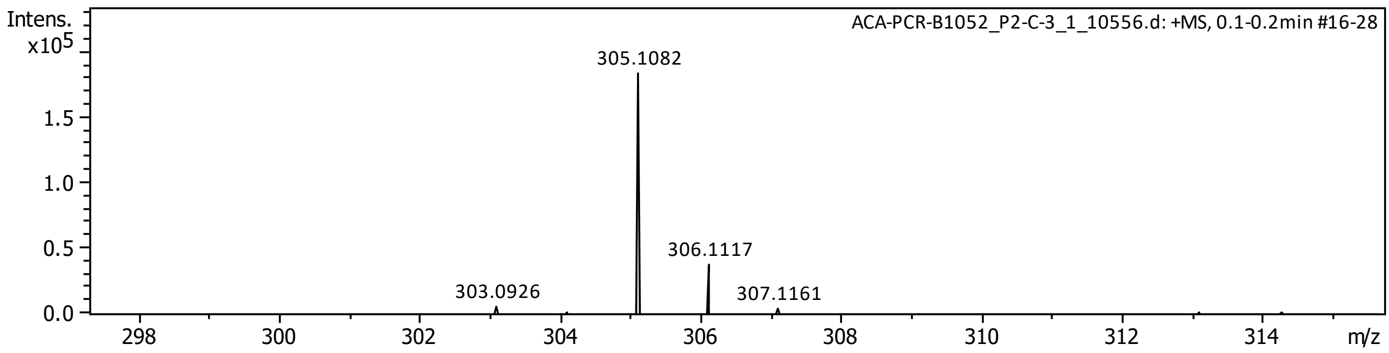

$[M+H]^+$ +2,7 ppm

Figure S 97: ESI-HRMS of **7a/b** synthesized with (**1d**) (THF)

Figures in this document:

Figure S 1: ^{1}H NMR spectra of $HPPh_2$, TMB, PhCCH and 2c (10 mol-%) in THF-d_8 (500 MHz) before and after 16 h irradiation: Signal assignment A + B: *H*-$PMes_2$ (5.23 ppm, d, *J* = 229 Hz, product of ligand exchange, 10 %), C: *H*-PPh_2 (5.19 ppm, d, *J* = 218 Hz), D: (*Z*)-4 (4b, 10%), E – F: $H_{Styrene}$ (6, 5%t). ^{1}H-resonances of (*E*)-4 (4a) are overlapping with catalyst signals and other impurities. Yield determined by ^{31}P NMR spectroscopy (see below). 16

Figure S 2: $^{31}P\{^{1}H\}$ NMR spectra of $HPPh_2$, TMB, PhCCH and 2c (10 mol-%) in THF-d_8 (500 MHz) before and after 16 h irradiation and ^{31}P NMR spectrum after irradiation: A: $HPMes_2$, B: (*Z*)-4 (4b, 10%) and C: (*E*)-4 (4a, 80%). The resonance at –14.7 ppm is assigned to trace amounts of Ph_4P_2.[13] 17

Figure S 3: ^{1}H NMR spectra of $HPPh_2$, PhCCH and 2c (5 mol-%) in THF-d_8 before and after 16 h irradiation: Signal assignment A + B: *H*-$PMes_2$ (5.23 ppm, d, *J* = 229 Hz, product of ligand exchange, 10 %), C: *H*-PPh_2 (5.19 ppm, d, *J* = 218 Hz), D: (*Z*)-4 (4b, 34%), E – G: $H_{Styrene}$ (6, 4%). ^{1}H-resonances of (*E*)-4 (4a) are overlapping with catalyst signals and other impurities. Yield determined by ^{31}P NMR spectroscopy (see below). 18

Figure S 4: $^{31}P\{^{1}H\}$ NMR spectra of $HPPh_2$, PhCCH (3) and 2c (5 mol-%) in THF-d_8 before and after 16 h irradiation. A: $HPMes_2$, B: (*Z*)-4 (4b, 34%) and C: (*E*)-4 (4a, 65%). The resonance at –14.7 ppm is assigned to trace amounts of Ph_4P_2.[13] 19

Figure S 5: (^{1}H,^{31}P)-HMBC NMR spectrum of $HPPh_2$, PhCCH (3) and 2c (5 mol-%) in THF-d_8 after 16 h irradiation in THF-d_8. 20

Figure S 6: ^{1}H NMR spectra of $HPPh_2$, PhCCH and 2c (5 mol-%) in THF-d_8 before and after 16 h irradiation Signal assignment A + B: *H*-$PMes_2$ (5.23 ppm, d, *J* = 229 Hz, product of ligand exchange, 10 %), C: *H*-PPh_2 (5.19 ppm, d, *J* = 218 Hz), D: (*Z*)-4 (4b, 67%), E – G: $H_{Styrene}$ (6, 5%). ^{1}H-resonances of (*E*)-4 (4a) are overlapping with catalyst signals and other impurities. Yield determined by ^{31}P NMR spectroscopy (see below). 21

Figure S 7: $^{31}P\{^{1}H\}$ NMR spectra of $HPPh_2$, PhCCH (3) and 2c (5 mol-%) in THF-d_8 before and after 16 h irradiation. A: $HPMes_2$, B: (*Z*)-4 (4b, 67%) and C: (*E*)-4 (4a, 28%). The resonance at –14.7 ppm is assigned to trace amounts of Ph_4P_2.[13] 22

Figure S 8: Time-conversion plot showing the NMR yield of the different species observed (see above) during the reaction of diphenylphosphine ($HPPh_2$) and phenylacetylene (3) catalyzed by 2c (2 mol-%) under irradiation of light (475 nm). The amount of non-aromatic protons should be equal at all times........ 23

Figure S 9: ^{1}H NMR spectra of $HPPh_2$, PhCCH and 2c (5 mol-%) in THF-d_8 before irradiation (above) and after one catalytic cycle (mid) and after two catalytic cycles (below Signal assignment A + B: *H*-$PMes_2$ (5.23 ppm, d, *J* = 229 Hz, product of ligand exchange, 10 %), C: *H*-PPh_2 (5.19 ppm, d, *J* = 218 Hz), D: (*Z*)-4 (4b, 67%), E – G: $H_{Styrene}$ (6, 5%). ^{1}H-resonances of (*E*)-4 (4a) are overlapping with catalyst signals and other impurities. Yield determined by ^{31}P NMR spectroscopy (see below). 24

Figure S 10: $^{31}P\{^{1}H\}$ NMR spectra of $HPPh_2$, PhCCH (3) and 2c (5 mol-%) in THF-d_8 before and after 16 h irradiation. A: $HPMes_2$, B: (*Z*)-4 (4b, 56%) and C: (*E*)-4 (4a, 32%). 25

Figure S 11: ^{1}H NMR spectra of $HPPh_2$, PhCCH and 2c (5 mol-%) in THF-d_8 before and after 16 h irradiation: Signal assignment A + B: *H*-$PMes_2$ (5.23 ppm, d, *J* = 229 Hz, product of ligand exchange, 10 %), C: *H*-PPh_2

(5.19 ppm, d, *J* = 218 Hz), D: (*Z*)-4 (4b, 83%), E – G: $H_{Styrene}$ (6, 2%), H – I: 5 (1 %). ^{1}H-resonances of (*E*)-4 (4a) are overlapping with catalyst signals and other impurities. Yield determined by ^{31}P NMR spectroscopy (see below). 26

Figure S 12: ^{31}P{^{1}H} NMR spectra of $HPPh_2$, PhCCH (3) and 2d (2 mol-%) in THF-d_8 before and after 16 h irradiation. A: $HPMes_2$, B: (*Z*)-4 (4b, 11%) and C: (*E*)-4 (4a, 83%). The resonance at δ = –4.5 ppm is assigned to the *gem*-addition product 5 and the resonance at δ = –14.7 ppm is assigned to trace amounts of Ph_4P_2.[13] 27

Figure S 13: ^{13}C{^{1}H} NMR spectra of $HPPh_2$, PhCCH and 2d (2 mol-%) in THF-d_8 after 16 h irradiation. TMB = 1,3,5-trimethoxybenzene (internal standard). 27

Figure S 14: (^{1}H,^{1}H)-COSY NMR spectrum of $HPPh_2$, PhCCH (3) and 2c (5 mol-%) in THF-d_8 after 16 h irradiation in THF-d_8. 28

Figure S 15: (^{1}H,^{31}P)-HMBC NMR spectrum of $HPPh_2$, PhCCH (3) and 2c (5 mol-%) in THF-d_8 after 16 h irradiation in THF-d_8. 28

Figure S 16: ^{1}H NMR spectra of $HPPh_2$, PhCCH and 2c (10 mol-%) in C_6D_6 before and after 16 h irradiation: Signal assignment A + B: *H*-$PMes_2$ (5.23 ppm, d, *J* = 229 Hz, product of ligand exchange, 10 %), C: *H*-PPh_2 (5.19 ppm, d, *J* = 218 Hz)$_2$, D: (*Z*)-4 (4b, <5%), E – G: $H_{Styrene}$ (6, 2%). ^{1}H-resonances of (*E*)-4 (4a) are overlapping with catalyst signals and other impurities. Yield determined by ^{31}P NMR spectroscopy (see below). 29

Figure S 17: ^{31}P{^{1}H} NMR spectra of $HPPh_2$, PhCCH (3) and 2d (10 mol-%) in THF-d_8 before and after 16 h irradiation. A: $HPMes_2$, B: (*Z*)-4 (4b, <5%) and C: (*E*)-4 (4a, >95%). 30

Figure S 18: ^{1}H NMR spectra of $HPPh_2$, PhCCH and 2c (2 mol-%) in C_6D_6 before and after 16 h irradiation: Signal assignment A + B: *H*-$PMes_2$ (5.23 ppm, d, *J* = 229 Hz, product of ligand exchange, 10 %), C: *H*-PPh_2 (5.19 ppm, d, *J* = 218 Hz), D: (*Z*)-4 (4b, 63%), E – G: $H_{Styrene}$ (6, 2%). ^{1}H-resonances of (*E*)-4 (4a) are overlapping with catalyst signals and other impurities. Yield determined by ^{31}P NMR spectroscopy (see below). 31

Figure S 19: ^{31}P{^{1}H} NMR spectra of $HPPh_2$, PhCCH (3) and 2d (2 mol-%) in THF-d_8 before and after 16 h irradiation. A: $HPMes_2$, B: (*Z*)-4 (4b, 63%) and C: (*E*)-4 (4a, 28%). 32

Figure S 20: ^{1}H NMR spectra of $HPMes_2$, PhCCH and 2c in THF-d_8 before and after 20 h irradiation. 33

Figure S 21: ^{31}P{^{1}H} NMR spectra of $HPMes_2$, PhCCH and 2c in THF-d_8 before and after 20 h irradiation. 34

Figure S 22: ^{1}H NMR and ^{31}P{^{1}H} NMR spectra of $HPMes_2$, PhCCH and 2c (10 mol-%) in C_6D_6 after 24 h irradiation. 35

Figure S 23: ^{1}H NMR and ^{31}P{^{1}H} NMR spectra of $HPMes_2$, PhCCH and in THF-d_8 after 16 h irradiation. 36

Figure S 24: ^{1}H NMR spectrum of the ligand protolysis reaction (see scheme S1). 37

Figure S 25:{^{1}H}^{31}P NMR spectra of the ligand protolysis reaction (see scheme S1). 38

Figure S 26: Molecular structure of 2a in crystalline state (ORTEP plot, left) and unit cell of the crystal of 2a (right). 42

Figure S 27: Molecular structure of 2b in crystalline state (ORTEP plot, left) and unit cell of the crystal of 2b (right). 43

Figure S 28: Molecular structure of 2c in crystalline state (ORTEP plot, left) and unit cell of the crystal of 2c (right). 44
Figure S 29: Molecular structure of 2d in crystalline state (ORTEP plot, left) and unit cell of the crystal of 2d (right). 45
Figure S 30: Molecular structure of 7a in crystalline state (ORTEP plot, left) and unit cell of the crystal of 7a (right). 46
Figure S 31: Graphical representation of the buried volume (V_{bur} in %) of complexes 2a – 2d and respective molecular structures in solid state with important bond lengths and angles. 47
Figure S 32: ^{1}H NMR spectrum of dimesitylphosphine oxide (400 MHz, $CDCl_3$, 298 K). 49
Figure S 33: {^{1}H}^{13}C NMR spectrum of dimesitylphosphine oxide (400 MHz, $CDCl_3$, 298 K). 50
Figure S 34: {^{1}H}^{31}P NMR spectrum of dimesitylphosphine oxide (162 MHz, $CDCl_3$, 298 K). 51
Figure S 35: ^{1}H NMR spectrum of dimesitylphosphine (500 MHz, $CDCl_3$, 298 K). 52
Figure S 36: {^{1}H}^{13}C NMR spectrum of dimesitylphosphine (151 MHz, $CDCl_3$, 298 K). 53
Figure S 37: ^{31}P NMR spectrum of dimesitylphosphine (203 MHz, $CDCl_3$, 298 K). 54
Figure S 38: ^{1}H NMR spectrum of [Cu(^{Me}cAAC)($PMes_2$)] (2a) (500 MHz, THF-d_8, 298 K). 55
Figure S 39: {^{1}H}^{13}C NMR spectrum of [Cu(^{Me}cAAC)($PMes_2$)] (2a) (126 MHz, THF-d_8, 298 K). 56
Figure S 40: DEPT-135 NMR spectrum of [Cu(^{Me}cAAC)($PMes_2$)] (2a) (126 MHz, THF-d_8, 298 K). 57
Figure S 41: {^{1}H}^{31}P NMR spectrum of [Cu(^{Me}cAAC)($PMes_2$)] (2a) (162 MHz, THF-d_8, 298 K). 58
Figure S 42: (^{1}H,^{15}N)-HMBC spectrum of [Cu(^{Me}cAAC)($PMes_2$)] (2a) (500 MHz, 51 MHz, THF-d_8, 298 K). 59
Figure S 43: (^{1}H,^{1}H)-COSY spectrum of [Cu(^{Me}cAAC)($PMes_2$)] (2a) (500 MHz, THF-d_8, 298 K). 60
Figure S 44: (^{1}H,^{1}H)-NOESY spectrum of [Cu(^{Me}cAAC)($PMes_2$)] (2a) (500 MHz, THF-d_8, 298 K). 61
Figure S 45: (^{1}H,^{13}C)-HSQC spectrum of [Cu(^{Me}cAAC)($PMes_2$)] (2a) (500 MHz, 126 MHz, THF-d_8, 298 K). 62
Figure S 46: (^{1}H,^{13}C)-HMBC spectrum of [Cu(^{Me}cAAC)($PMes_2$)] (2a) (500 MHz, 126 MHz, THF-d_8, 298 K). 63
Figure S 47: ^{1}H NMR spectrum of [Cu(^{Et}cAAC)($PMes_2$)] (2b) (500 MHz, THF-d_8, 298 K). 64
Figure S 48: {^{1}H}^{13}C NMR spectrum of [Cu(^{Et}cAAC)($PMes_2$)] (2b) (126 MHz, THF-d_8, 298 K). 65
Figure S 49: DEPT-135 NMR spectrum of [Cu(^{Et}cAAC)($PMes_2$)] (2b) (126 MHz, THF-d_8, 298 K). 66
Figure S 50: {^{1}H}^{31}P NMR spectrum of [Cu(^{Et}cAAC)($PMes_2$)] (2b) (162 MHz, THF-d_8, 298 K). 67
Figure S 51: (^{1}H,^{15}N)-HMBC NMR spectrum of [Cu(^{Et}cAAC)($PMes_2$)] (2b) (500 MHz, 51 MHz, THF-d_8, 298 K). 68
Figure S 52: (^{1}H,^{1}H)-COSY NMR spectrum of [Cu(^{Et}cAAC)($PMes_2$)] (2b) (500 MHz, THF-d_8, 298 K). 69
Figure S 53: (^{1}H,^{1}H)-NOESY NMR spectrum of [Cu(^{Et}cAAC)($PMes_2$)] (2b) (500 MHz, THF-d_8, 298 K). 70
Figure S 54: (^{1}H,^{13}C)-HSQC NMR spectrum of [Cu(^{Et}cAAC)($PMes_2$)] (2b) (500 MHz, 126 MHz, THF-d_8, 298 K). 71
Figure S 55: (^{1}H,^{13}C)-HMBC NMR spectrum of [Cu(^{Et}cAAC)($PMes_2$)] (2b) (500 MHz, 126 MHz, THF-d_8, 298 K). 72
Figure S 56: ^{1}H NMR spectrum of [Cu(^{Cy}cAAC)($PMes_2$)] (2c) (400 MHz, C_6D_6, 298 K). 73
Figure S 57: ^{1}H NMR spectrum of [Cu(^{Cy}cAAC)($PMes_2$)] (2c) (600 MHz, THF-d_8, 298 K). 74
Figure S 58: {^{1}H}^{13}C NMR spectrum of [Cu(^{Cy}cAAC)($PMes_2$)] (2c) (101 MHz, C_6D_6, 298 K). 75
Figure S 59: {^{1}H}^{13}C NMR spectrum of [Cu(^{Cy}cAAC)($PMes_2$)] (2c) (151 MHz, THF-d_8, 298 K). 76
Figure S 60: DEPT-135 NMR spectrum of [Cu(^{Cy}cAAC)($PMes_2$)] (2c) (151 MHz, THF-d_8, 298 K). 77
Figure S 61: {^{1}H}^{31}P NMR spectrum of [Cu(^{Cy}cAAC)($PMes_2$)] (2c) (126 MHz, C_6D_6, 298 K). 78
Figure S 62: {^{1}H}^{31}P NMR spectrum of [Cu(^{Cy}cAAC)($PMes_2$)] (2c) (243 MHz, THF-d_8, 298 K). 79

Figure S 63: (^{1}H,^{15}N)-HMBC NMR spectrum of [Cu(^{Cy}cAAC)(PMes$_2$)] (2c) (600 MHz, 61 MHz, C_6D_6, 298 K). 80
Figure S 64: (^{1}H,^{15}N)-HMBC NMR spectrum of [Cu(^{Cy}cAAC)(PMes$_2$)] (2c) (600 MHz, 61 MHz, THF-d_8, 298 K). .. 81
Figure S 65: (^{1}H,^{1}H)-COSY NMR spectrum of [Cu(^{Cy}cAAC)(PMes$_2$)] (2c) (600 MHz, THF-d_8, 298 K). 82
Figure S 66: (^{1}H,^{1}H)-NOESY NMR spectrum of [Cu(^{Cy}cAAC)(PMes$_2$)] (2c) (600 MHz, THF-d_8, 298 K). 83
Figure S 67: (^{1}H,^{13}C)-HSQC NMR spectrum of [Cu(^{Cy}cAAC)(PMes$_2$)] (2c) (600 MHz, 151MHz, THF-d_8, 298 K). ... 84
Figure S 68: (^{1}H,^{13}C)-HMBC NMR spectrum of [Cu(^{Cy}cAAC)(PMes$_2$)] (2c) (600 MHz, 151 MHz, THF-d_8, 298 K). . 85
Figure S 69: ^{1}H NMR spectrum of [Cu(^{Ment}cAAC)(PMes$_2$)] (2d) (600 MHz, THF-d_8, 298 K). 86
Figure S 70: {^{1}H}^{13}C NMR spectrum of [Cu(^{Ment}cAAC)(PMes$_2$)] (2d) (151 MHz, THF-d_8, 298 K). 87
Figure S 71: DEPT-135 NMR spectrum of [Cu(^{Ment}cAAC)(PMes$_2$)] (2d) (126 MHz, THF-d_8, 298 K). 88
Figure S 72: {^{1}H}^{31}P NMR spectrum of [Cu(^{Ment}cAAC)(PMes$_2$)] (2d) (243 MHz, THF-d_8, 298 K). 89
Figure S 73: (^{1}H,^{15}N)-HMBC NMR spectrum of [Cu(^{Ment}cAAC)(PMes$_2$)] (2d) (600 MHz, 61 MHz, THF-d_8, 298 K). 90
Figure S 74: (^{1}H,^{1}H)-COSY NMR spectrum of [Cu(^{Ment}cAAC)(PMes$_2$)] (2d) (600 MHz, THF-d_8, 298 K). 91
Figure S 75: (^{1}H,^{1}H)-NOESY NMR spectrum of [Cu(^{Ment}cAAC)(PMes$_2$)] (2d) (600 MHz, THF-d_8, 298 K). 92
Figure S 76: (^{1}H,^{13}C)-HSCQ NMR spectrum of [Cu(^{Ment}cAAC)(PMes$_2$)] (2d) (600 MHz, 151 MHz, THF-d_8, 298 K). 93
Figure S 77: (^{1}H,^{13}C)-HMBC NMR spectrum of [Cu(^{Ment}cAAC)(PMes$_2$)] (2d) (600 MHz, 151 MHz, THF-d_8, 298 K). 94
Figure S 78: ^{1}H NMR spectrum of crude 7a/b from 1c (400 MHz, $CDCl_3$, 298 K). 95
Figure S 79: ^{1}H NMR spectrum of purified 7a/b from 1c (400 MHz, $CDCl_3$, 298 K). 96
Figure S 80: {^{1}H}^{13}C NMR spectrum of purified 7a/b from 1c (101 MHz, $CDCl_3$, 298 K). 97
Figure S 81: DEPT-135 NMR spectrum of purified 7a/b from 1c (101 MHz, $CDCl_3$, 298 K). 98
Figure S 82: {^{1}H}^{31}P NMR spectrum of purified 7a/b from 1c (202 MHz, $CDCl_3$, 298 K). 99
Figure S 83: ^{1}H NMR spectrum of crude 7a/b from 1d (101 MHz, $CDCl_3$, 298 K). 100
Figure S 84: ^{1}H NMR spectrum of the oxidation product in 1,2-difluorobenzene/THF-d8 (second run). 101
Figure S 85: {^{1}H}^{31}P NMR spectrum spectrum of the oxidation product in 1,2-difluorobenzene/THF-d8 (second run). 102
Figure S 86: MIR-Absorption spectrum (ATR) of [Cu(^{Me}cAAC)(PMes$_2$)] (2a) (neat, 298 K) 103
Figure S 87: MIR-Absorption spectrum (ATR) of [Cu(^{Et}cAAC)(PMes$_2$)] (2b) (neat, 298 K) 104
Figure S 88: MIR-Absorption spectrum (ATR) of [Cu(^{Cy}cAAC)(PMes$_2$)] (2c) (neat, 298 K) 105
Figure S 89: MIR-Absorption spectrum (ATR) of [Cu(^{Ment}cAAC)(PMes$_2$)] (2d) (neat, 298 K) 106
Figure S 90: MIR-Absorption spectrum (ATR) of 7a/b (neat, 298 K) 107
Figure S 91: ESI-HRMS of [Cu(^{Me}cAAC)(PMes$_2$)] (2a) (THF) 108
Figure S 92: ESI-HRMS of [Cu(^{Et}cAAC)(PMes$_2$)] (2b) (THF) 109
Figure S 93: ESI-HRMS of [Cu(^{Cy}cAAC)(PMes$_2$)] (2c) (THF) 110
Figure S 94: ESI-HRMS of [Cu(^{Ment}cAAC)(PMes$_2$)] (2c) (THF) 111
Figure S 95: ESI-HRMS of the product obtained upon reaction of 2c with dry oxygen (THF) and identified species and their exact masses. 113
Figure S 96: ESI-HRMS of 7a/b synthesized with (1c) (THF) 114

Figure S 97: ESI-HRMS of 7a/b synthesized with (1d) (THF) 115

Schemes in this document:

Scheme S 1: NMR scale hydrophosphination of phenylacetylene (3) with diphenylphosphine ($HPPh_2$) using compounds [Cu(^{Cy}cAAC)($PMes_2$)] (2c) or [Cu(^{Ment}cAAC)($PMes_2$)] (2d) and important ^{1}H- and ^{31}P NMR resonance values used for assignment of signals: 4b in $CDCl_3$ and [10] 5 in $CDCl_3$.[11] Styrene (6) was measured in THF-d_8 and ^{1}H NMR literature data for the vinylic protons of 4a is inconsistent due to overlap of the signal with the aromatic protons of the compound.[11,12] 15

Scheme S 2: NMR scale photocatalyzed hydrophosphination of phenylacetylene (3) with diphenylphosphine ($HPPh_2$) using 10 mol-% [Cu(^{Cy}cAAC)($PMes_2$)] (2c) in THF-d_8.. Conversion based on consumption of diphenylphosphine 16

Scheme S 3: NMR scale photocatalyzed hydrophosphination of phenylacetylene (3) with diphenylphosphine ($HPPh_2$) using 5 mol-% [Cu(^{Cy}cAAC)($PMes_2$)] (2c) in THF-d_8. Conversion based on consumption of diphenylphosphine. 18

Scheme S 4: NMR scale photocatalyzed hydrophosphination of phenylacetylene (3) with diphenylphosphine ($HPPh_2$) using 2 mol-% [Cu(^{Cy}cAAC)($PMes_2$)] (2c) in THF-d_8.. Conversion based on consumption of diphenylphosphine 21

Scheme S 5: NMR scale photocatalyzed hydrophosphination of phenylacetylene (3) with diphenylphosphine ($HPPh_2$) using 2 mol-% [Cu(^{Cy}cAAC)($PMes_2$)] (2c) in THF-d_8. And readdition of substrates to the reaction mixture. Conversion based on consumption of diphenylphosphine and yields are referring to the total amount of starting material added. 24

Scheme S 6: NMR scale photocatalyzed hydrophosphination of phenylacetylene (3) with diphenylphosphine ($HPPh_2$) using 5 mol-% [Cu(^{Ment}cAAC)($PMes_2$)] (2d) in THF-d_8.. Conversion based on consumption of diphenylphosphine. 26

Scheme S 7: NMR scale photocatalyzed hydrophosphination of phenylacetylene (3) with diphenylphosphine ($HPPh_2$) using 10 mol-% [Cu(^{Cy}cAAC)($PMes_2$)] (2c) in C_6D_6.. Conversion based on consumption of diphenylphosphine. 29

Scheme S 8: NMR scale photocatalyzed hydrophosphination of phenylacetylene (3) with diphenylphosphine ($HPPh_2$) using 2 mol-% [Cu(^{Cy}cAAC)($PMes_2$)] (2c) in C_6D_6.. Conversion based on consumption of diphenylphosphine. 31

Scheme S 9: NMR scale photocatalyzed hydrophosphination of phenylacetylene (3) with dimesitylphosphine ($HPMes_2$) using 10 mol-% [Cu(^{Cy}cAAC)($PMes_2$)] (2d) in THF-d_8.. Conversion based on consumption of dimesitylphosphine. 33

Scheme S 10: NMR scale reaction of phenylacetylene (3) with dimesitylphosphine ($HPMes_2$) using 10 mol-% [Cu(^{Cy}cAAC)($PMes_2$)] (2d) in THF-d_8 under exclusion of light for 24 h.. Conversion based on consumption of dimesitylphosphine. No onversion was observed. 35

Scheme S 11: NMR scale reaction of phenylacetylene (3) with dimesitylphosphine ($HPMes_2$) without any catalyst in THF-d_8. 35

Scheme S 12: Protolysis of 2c by phenylacetylene (3) 37

Tables in this document:

Table S 1: SC/XRD parameters of 2a - 2d and 7a. **41**

6. References

[1] A. Krasovskiy, P. Knochel, *Synthesis* **2006**, *2006*, 890.

[2] W. G. Kofron, L. M. Baclawski, *J. Org. Chem.* **1976**, *41*, 1879.

[3] G. R. Fulmer, A. J. M. Miller, N. H. Sherden, H. E. Gottlieb, A. Nudelman, B. M. Stoltz, J. E. Bercaw, K. I. Goldberg, *Organometallics* **2010**, *29*, 2176.

[4] Ē. Kupče, T. D. W. Claridge, *Angew. Chem. Int. Ed.* **2017**, *56*, 11779; *Angew. Chem.* **2017**, *129*, 11941.

[5] M. Gernert, U. Müller, M. Haehnel, J. Pflaum, A. Steffen, *Chem. Eur. J.* **2017**, *23*, 2206.

[6] A. M. T. Muthig, J. Wieland, C. Lenczyk, S. Koop, J. Tessarolo, G. H. Clever, B. Hupp, A. Steffen, *Chem. Eur. J.* **2023**, *29*, e202300946.

[7] C. A. Busacca, J. C. Lorenz, N. Grinberg, N. Haddad, M. Hrapchak, B. Latli, H. Lee, P. Sabila, A. Saha, M. Sarvestani et al., *Org. Lett.* **2005**, *7*, 4277.

[8] M. Metzler, M. Bolte, A. Virovets, H.-W. Lerner, M. Wagner, *Org. Lett.* **2023**, *25*, 5827.

[9] K. Izod, D. G. Rayner, S. M. El-Hamruni, R. W. Harrington, U. Baisch, *Angew. Chem. Int. Ed.* **2014**, *53*, 3636; *Angew. Chem.* **2014**, *126*, 3710.

[10] Y. Moglie, M. J. González-Soria, I. Martín-García, G. Radivoy, F. Alonso, *Green Chem.* **2016**, *18*, 4896.

[11] Q. Lin, F. Sun, T. Wang, J. Yang, J. Tang, W. Liu, *Adv. Synth. Catal.* **2025**, *367*.

[12] a) M. Hayashi, Y. Matsuura, Y. Watanabe, *J. Org. Chem.* **2006**, *71*, 9248; b) M. Hayashi, Y. Matsuura, Y. Watanabe, *Tetrahedron Lett.* **2004**, *45*, 9167; c) H. Jin, C. Gu, Z. Xiao, Q. Tan, L. Liu, L.-B. Han, W.-H. Chen, T. Chen, *Eur. J. Org. Chem.* **2023**, *26*.

[13] D. L. Dodds, M. F. Haddow, A. G. Orpen, P. G. Pringle, G. Woodward, *Organometallics* **2006**, *25*, 5937.

[14] T. Kottke, D. Stalke, *J. Appl. Cryst.* **1993**, *26*, 615.

[15] G. M. Sheldrick, *Acta Crystallogr. A* **2015**, *71*, 3.

[16] C. B. Hübschle, G. M. Sheldrick, B. Dittrich, *J. Appl. Cryst.* **2011**, *44*, 1281.

[17] G. M. Sheldrick, *Acta Crystallogr. C* **2015**, *71*, 3.

[18] a) D. Kratzert, J. J. Holstein, I. Krossing, *J. Appl. Cryst.* **2015**, *48*, 933; b) D. Kratzert, I. Krossing, *J. Appl. Cryst.* **2018**, *51*, 928.

[19] A. Thorn, B. Dittrich, G. M. Sheldrick, *Acta Crystallogr. A* **2012**, *68*, 448.

[20] A. L. Spek, *Acta Crystallogr. C* **2015**, *71*, 9.

[21] A. L. Spek, *Acta Crystallogr. D* **2009**, *65*, 148.

[22] S. Parsons, H. D. Flack, T. Wagner, *Acta Crystallogr. B* **2013**, *69*, 249.
