# Supplementary material for: Organophosphides: A New Class of Luminophore Ligands for Copper(I) Carbene Based TADF Emitters and Photocatalysts
Source: Angew Chem Int Ed Engl. 2026 Feb 25;65(13):e18530. doi: 10.1002/anie.202518530 (PMC13007583; doi:10.1002/anie.202518530)
Supplement: Supplementary file 2 — Supporting File 2: anie71068‐sup‐0002‐SuppMat2.pdf. [file ANIE-65-e18530-s004.pdf]

Table of Contents

1. General considerations 2

2. Absorption Data 3

3. Emission Data 7

4. Computational Studies 16

   1. Geometry optimized coordinates 16

   2. Calculated Excited States and Transitions 31

   3. Difference density plots 53

5. References 61

1. GENERAL CONSIDERATIONS

All photophysical measurements were conducted under strict exclusion of oxygen, moisture and organohalide compounds in purified, degassed solvents (toluene), in powdered form or embedded in poly(styrene) or poly(methylmethacrylate) matrice. For measurements in films, a solution of 100 mg polymer substrate in 5 mL THF was prepared inside an argon filled glove-box and once the polymer completely dissolved, 2 wt-% of the respective emitter substance was added. The solution was dropcasted onto a glass substrate and the solvent was slowly evaporated. This process was repeated four times and the resulting film was thoroughly dried for 18 h under vacuum. The films were detached with a scalpel and rolled up into a quartz tube for investigation inside an OptiStat system or placed into a cryosphere for quantum yield measurement. UV/Vis absorption spectroscopy was performed using an *Agilent Cary 60* spectrophotometer using 1 cm path length quartz cells equipped with a PTFE J-Young screw-tap. Steady-state emission and excitation spectra were recorded on an *Edinburgh Instrument FLS1000* spectrometer or an *Edinburgh Instrument FS5* spectrometer (visible range only). A 450 W Xenon arc lamp was used as excitation light source.

In excitation and emission pathways, single (FS5, DXUV UV holographic grating, optimised for use in FS5) or double slit (FLS1000, N-DM830/1200 Grating for emission monochromator: 830 grooves/mm - blaze 1200 nm) monochromators were used together with a red-sensitive photomultiplier tube (PMT-980) as detector (for 250 – 850 nm). For NIR-measurements (only FLS1000; 850 nm – 1200 nm), a liquid nitrogen-cooled NIR sensitive photomultiplier tube (PMT-1400) was employed. The excitation and emission spectra were corrected using the standard correction files supplied by the manufacturer for the excitation source's spectral power and the detector's sensitivity. Quantum yields in the solid state or in films were measured using an *FLS1000* spectrometer equipped with an integrating cryosphere (*Microstat N2*) from Oxford Instruments. Quantum yields in solution were recorded via relative external reference method (Oxazine 1 in MeOH, CAS: 24796-94-9) in analogy to a known procedure.[1] The luminescence lifetimes were measured using a *µF2* pulsed 60 W Xenon microsecond flashlamp, with a repetition rate of 100 Hz and a multichannel scaling (MCS) module or with VPLEDs (449.6 nm with 37 mW), with 60 ns to 500 ns pulse width and an MCS module, depending on the time range. The emission was collected at a 90° angle to the excitation source. The low-temperature experiments were performed using a liquid nitrogen-cooled *OptistatDN-V* cryostat from Oxford Instruments. Temperature control was achieved using a *MercuryITC* from *Oxford Instruments* in a temperature range between 77 K and 297 K ($\Delta T = \pm 0.5$ K).

2. ABSORPTION DATA

For all compounds, the absorption was measured with two independently prepared stock solutions of the compound in dry, degassed toluene inside an argon-filled glovebox. Molecular sieves' dust was removed by filtration through a PTFE syringe tip filter (0.2 µm). Volumes were measured using *Eppendorf* pipettes. Continuous and dotted lines represent the respective set of solutions derived from the same stock solution. Purple regression plot is attributed to the higher energy absorption, while the green regression plot represents the lower energy CT absorption band.

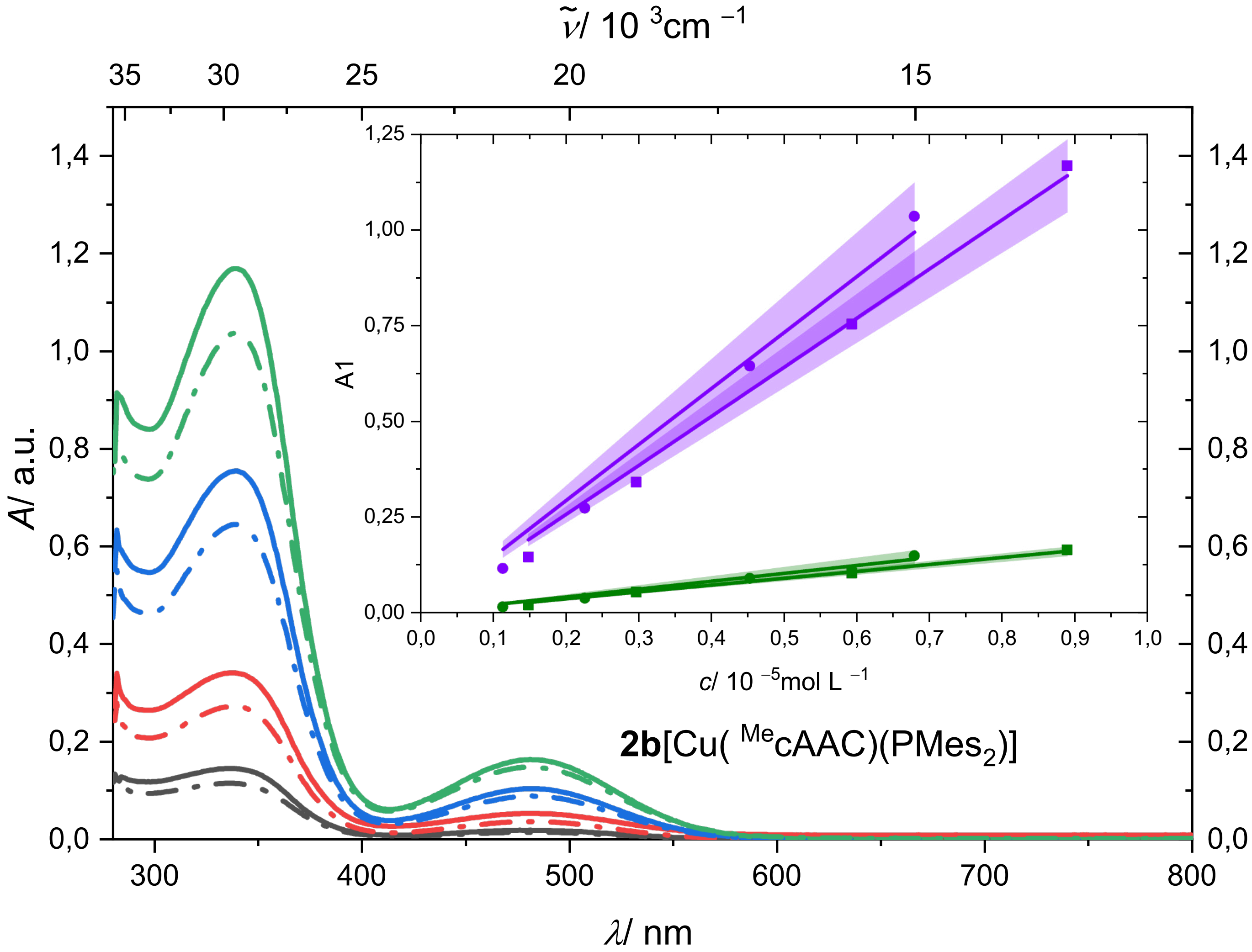

Figure S100: Absorption spectra of 2a

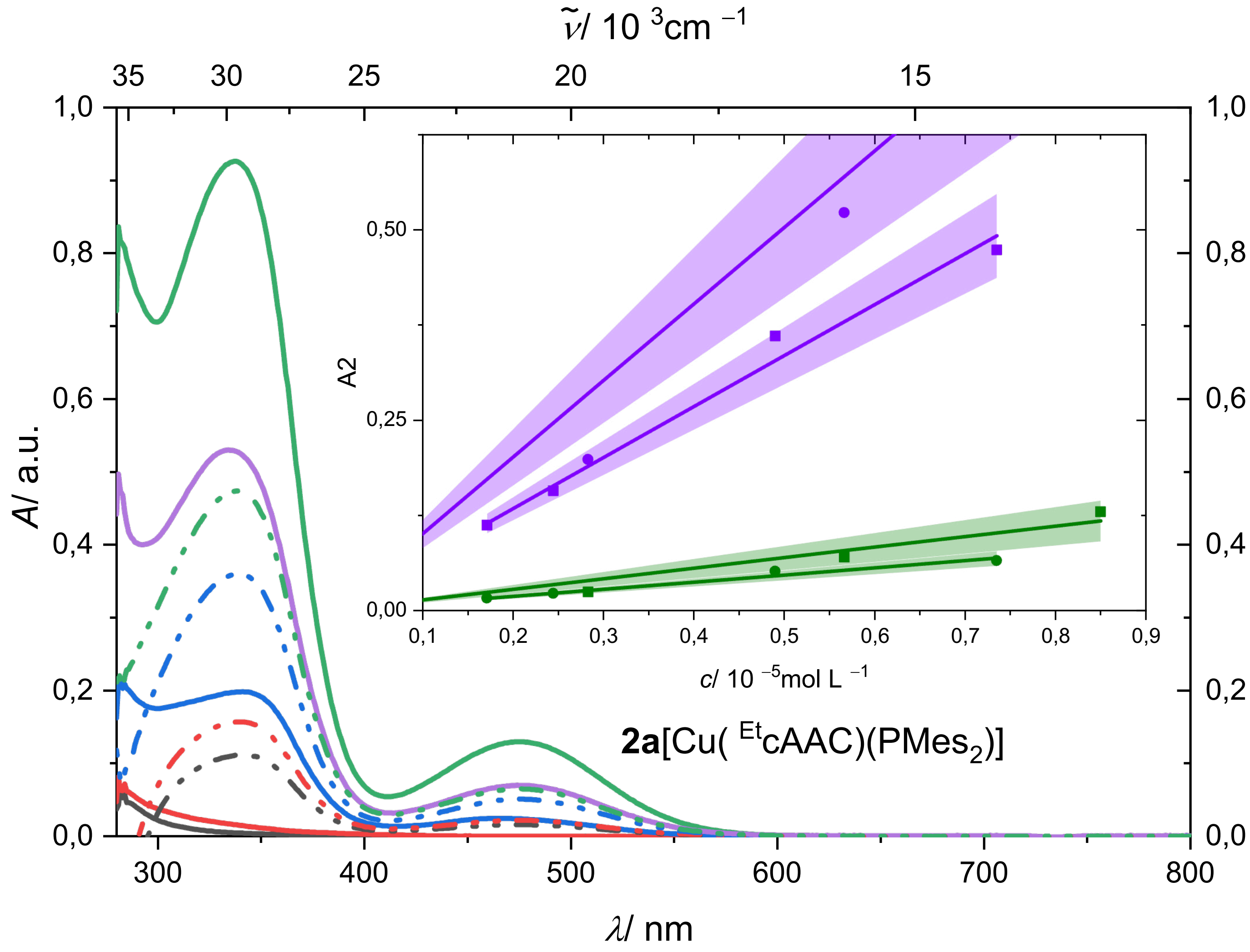

Figure S101: Absorption spectra of 2b

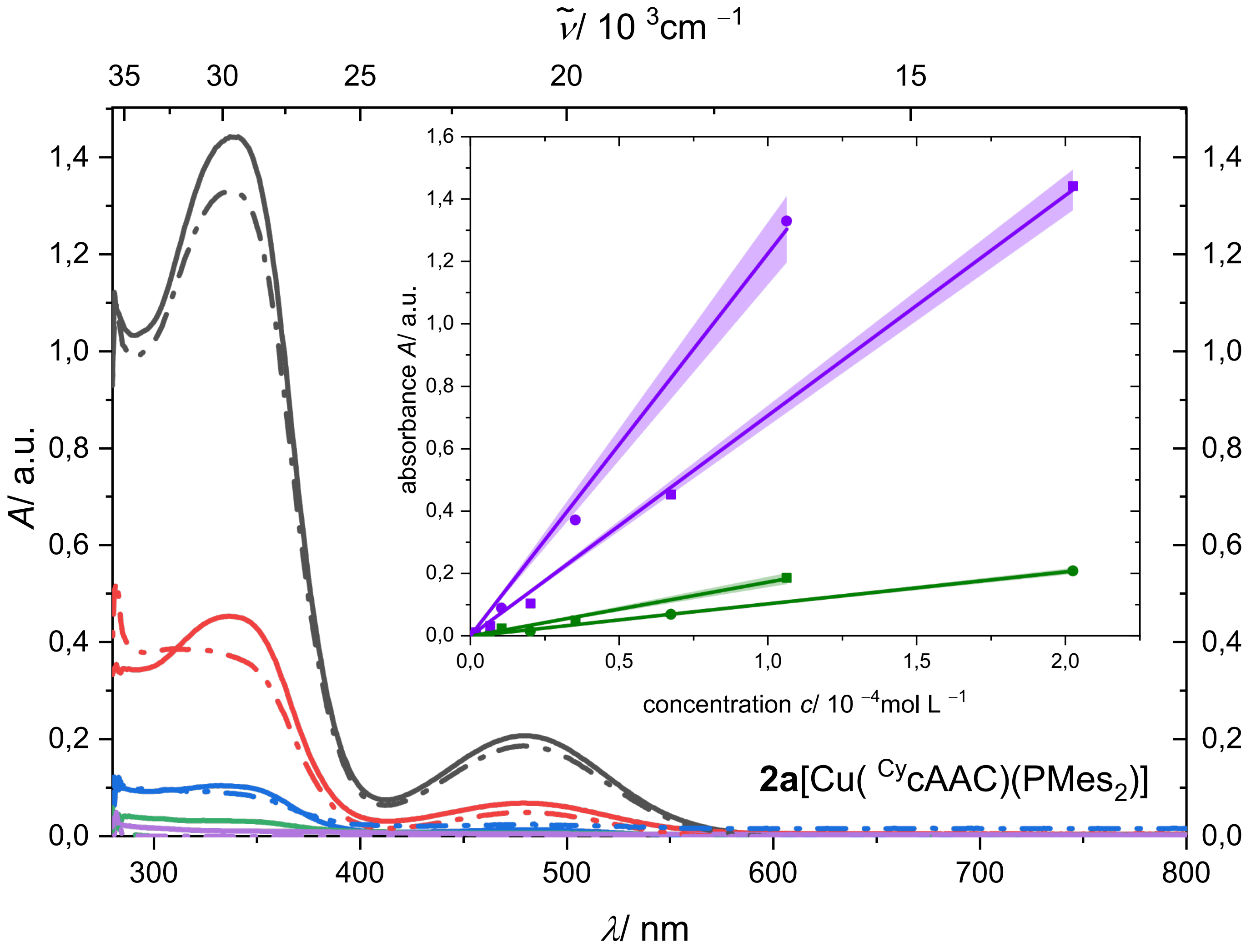

Figure S102: Absorption spectra of 2c

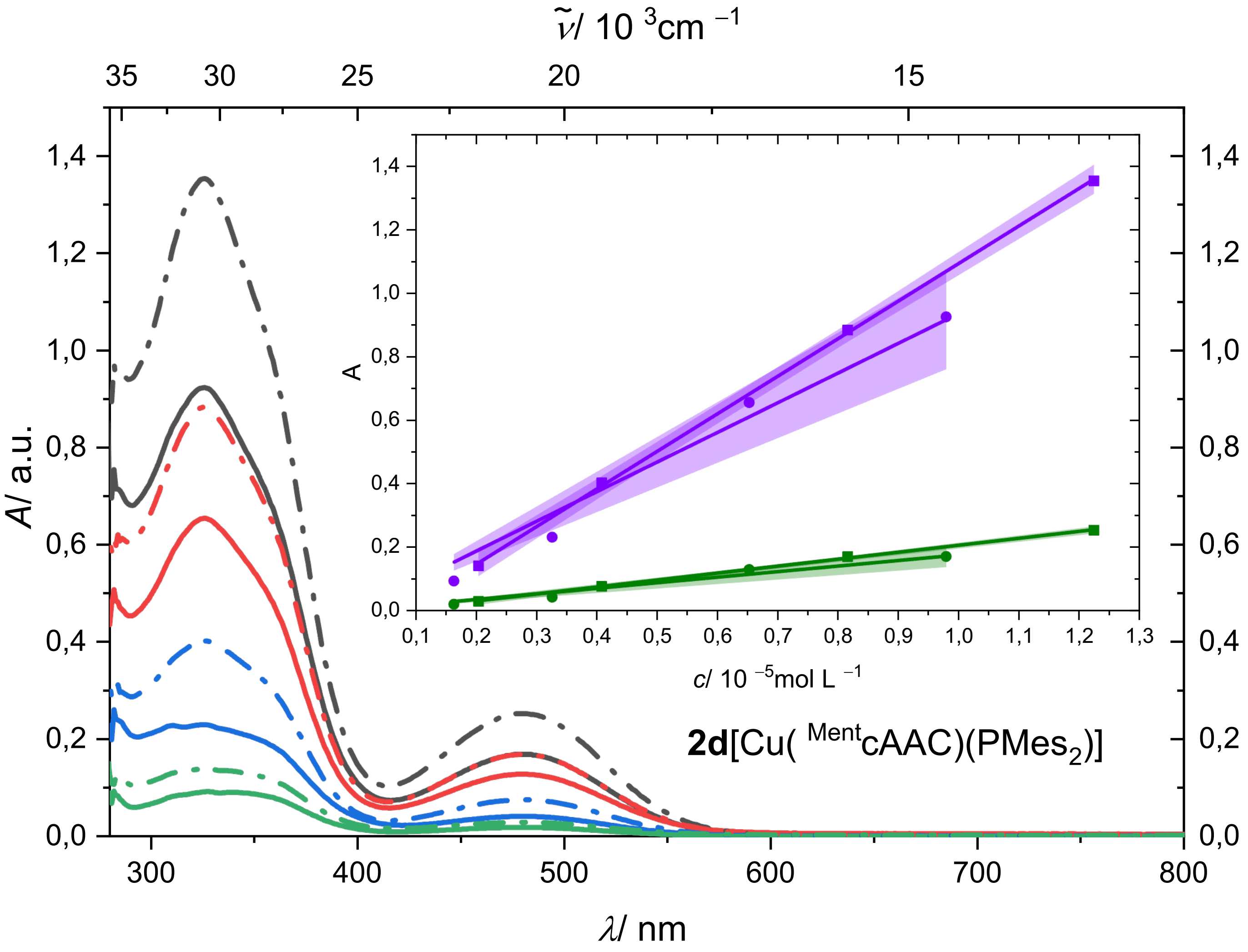

Figure S103: Absorption spectra of 2d

3. EMISSION DATA

Table S2: Summary of photophysical data for compounds 2a - 2d.

Conditions			[Cu(^{R}cAAC)(PMes$_2$)]			
State	T / K	Quantity	Me (**2a**)	Et (**2b**)	Cy (**2c**)	Ment (**2d**)
Toluene solution	297	ε_{max} / (M cm)$^{-1}$ (λ / nm)	15293 (339)<br>2112 (482)	9729 (337)<br>1382 (475)	9900 (338)<br>1400 (480)	10500 (326)<br>1900 (481)
Solid state	297	λ_{em} / nm	626	601	600	602
		$\lambda_{ex\ max}$ / nm	395	364	476	359
		Φ	0.47	0.30	0.52	0.45
		$\tau_{obs,\ averaged}$ / μs	7.91	16.5	12.0	13.5
		(lifetime components)	(2.36; 6.46; 10.8)	(15.6; 20.0)	(5.50; 13.0; 52.7)	(3.9; 14.5; 29.7)
		(weighting factors)	(3642; 23720; 18725)	(30991; 7982)	(3999; 24492; 59)	(3899; 27140; 564)
		$\langle k_r \rangle$ / 10^3 s^{-1}	59. 4	18.2	43.3	31.6
	77	λ_{em} / nm	612	652	610	650
		$\lambda_{ex\ max}$ / nm	392	398	484	419
		Φ	0.35	0.14	0.39	0.31
		$\tau_{obs,\ averaged}$ / μs	1085	264	1079	433
		(lifetime components)	(1053; 2140)	(107; 238; 569; 2130)	(1079)	(139; 446; 832)
		(weighting factors)	(10824; 330)	(3571; 9954; 2279; 67)	(4544)	(6823; 21937; 4328)
		$\langle k_r \rangle$ / 10^3 s^{-1}	0.32	0.53	0.36	0.72
Toluene solution	297	λ_{em} / nm	784	785	818	789
		λ_{ex} / nm	376; 474	371; 472	360; 474	293; 478
		Φ	< 0.01	< 0.01	0.00006[b]	0.0012[b]
		τ_{obs}<br>(lifetime components)<br>(weighting factors)<br>$\langle k_r \rangle$	n.d.[a]			
2 % (wt) in PS	297	λ_{em} / nm	682	679	677	662
		λ_{ex} / nm	472; 360	472; 346	472; 355	472; 342
		$\tau_{obs,\ averaged}$ / μ	1.51	1.62	2.55	5.99
		(lifetime components)	(0.54; 1.69; 4.70)	(0.50; 1.68; 4.44)	(1.09; 3.16; 7.56)	(1.65; 5.67; 12.7)
		(weighting factors)	(6654; 6936; 1641)	(35967; 49273; 13414)	(7432; 7679; 1216)	(11895; 16667; 7969)
		Φ	0.04	0.01	0.05	0.05
		$\langle k_r \rangle$ / 10^3 s^{-1}	26.5	6.17	19.6	8.30
2 % (wt) in PMMA	297	λ_{em} / nm	635	655	655	659
		λ_{ex} / nm	457; 367	457; 349	457; 357	457; 334
		$\tau_{obs,\ averaged}$ / μs	2.56	1.63	2.79	8.15
		(lifetime components)	(0.98; 3.11; 8.14)	(0.52; 1.74; 4.60)	(0.99; 3.13; 8.02)	(2.25; 8.42; 18.8)
		(weighting factors)	(34923; 31373; 5448)	(37496; 48823; 12174)	(16145; 18036; 4378)	(8950; 15455; 5123)
		Φ	0.07	0.07	0.07	0.07
		$\langle k_r \rangle$ / 10^3 s^{-1}	27.3	42.9	25.1	8.50

[a] not determined due to very weak signal. [b] using the reference quantum yield method (Oxazine 1 standard).[1]

Additional emission spectra

Normalized Emission of 2a-d in Toluene solution

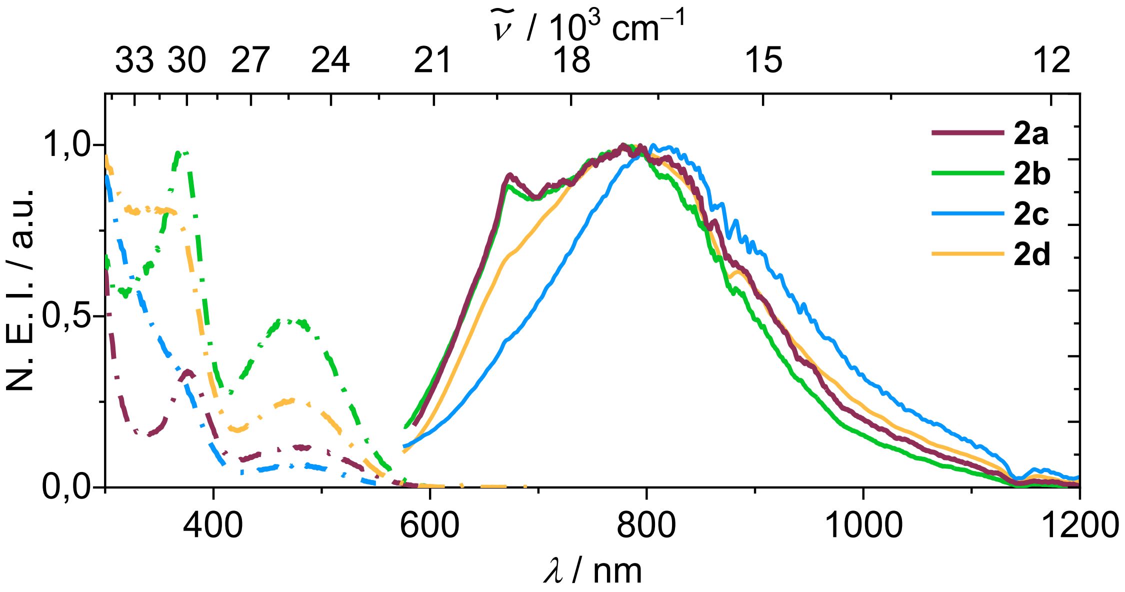

Figure S104: Normalized Emission of complexes 2a - 2d in toluene solution at 297 K

Normalized Emission and excitation spectra of compounds 2 at room temperature and 77 K

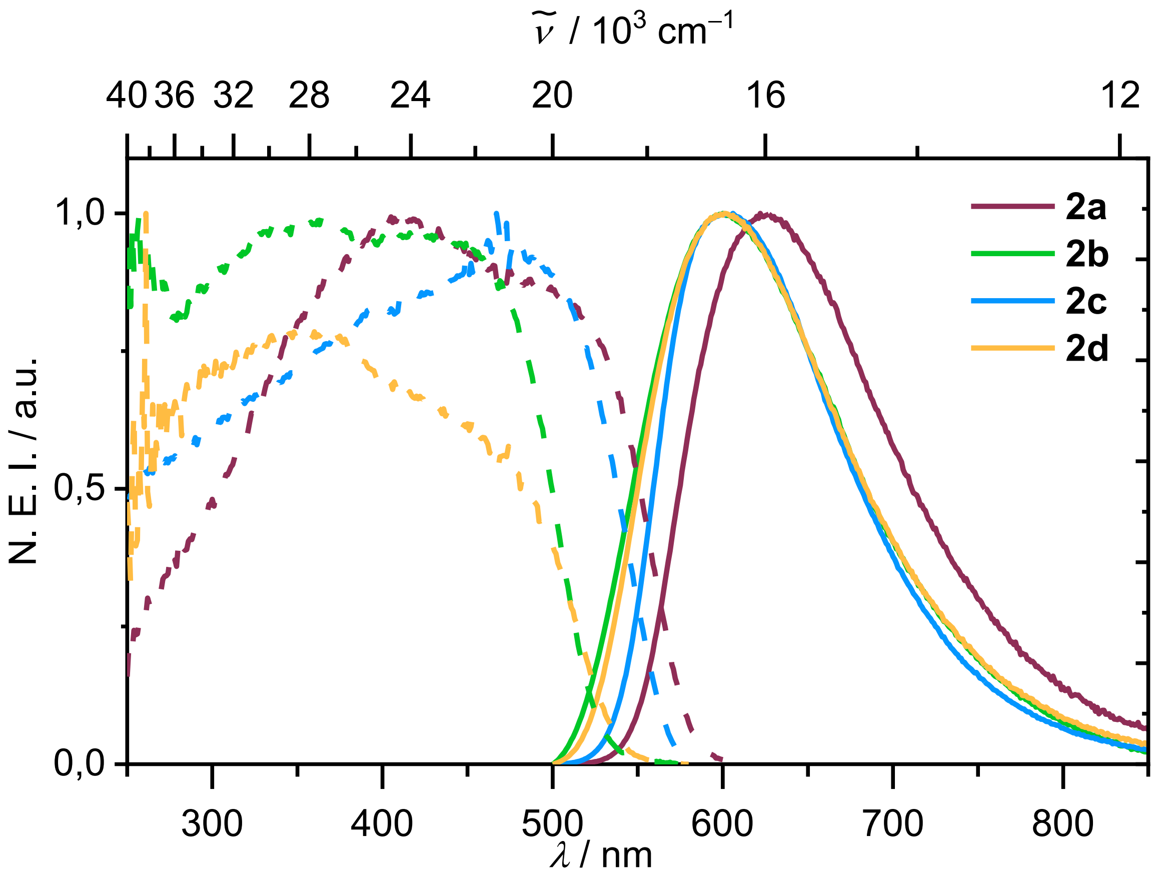

Figure S105: Normalized Emission and excitation spectra of 2a - 2d in solid state at 297 K

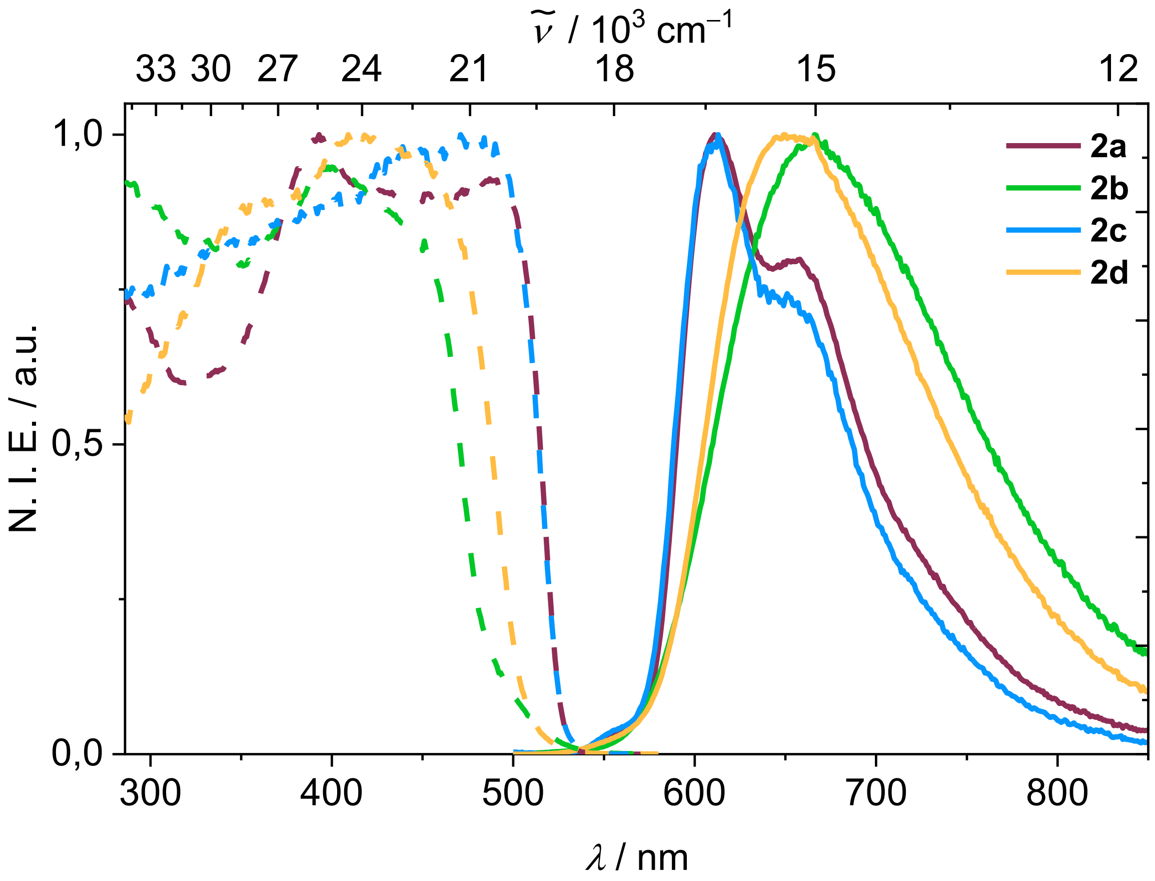

Figure S106: Normalized Emission and Excitation spectra of 2a - 2d in solid state at 77 K

Emission spectra (continuous) and excitation spectra (dotted) at room temperature (left) and 77 K (right) in solid state.

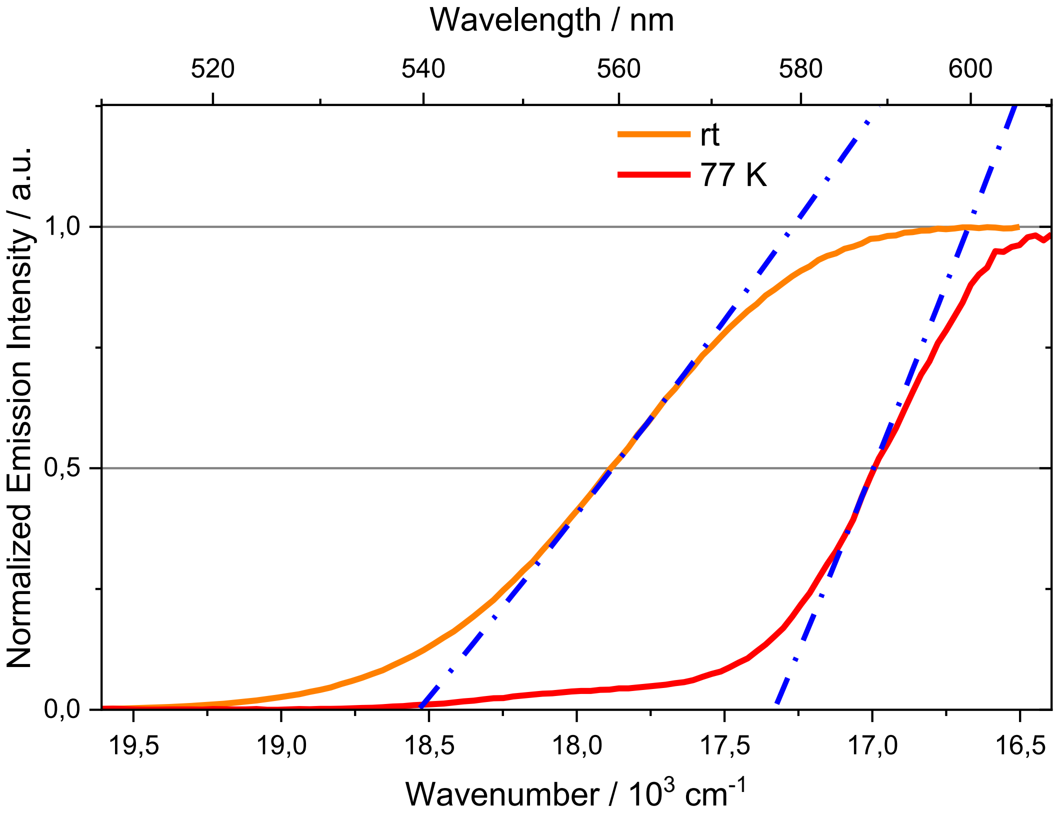

Figure S107: Normalized Emission spectra at 297 K and 77 K comparing the x-intercept o the emission bands

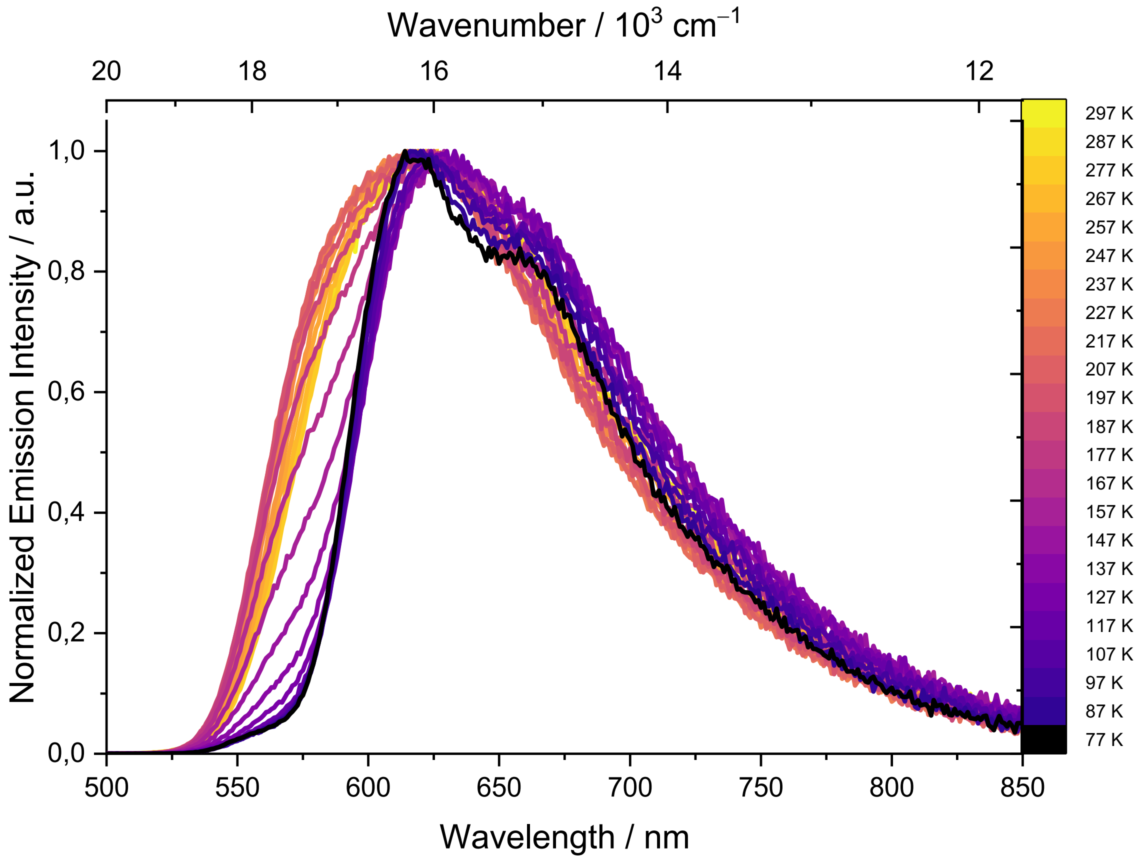

Figure S108: Normalized Emission spectra of 2c in solid state from 77 K to77K in 10 K increments

Normalized Emission- and excitation spectra of 2a-2d in PS and PMMA matrix (2 wt-%):

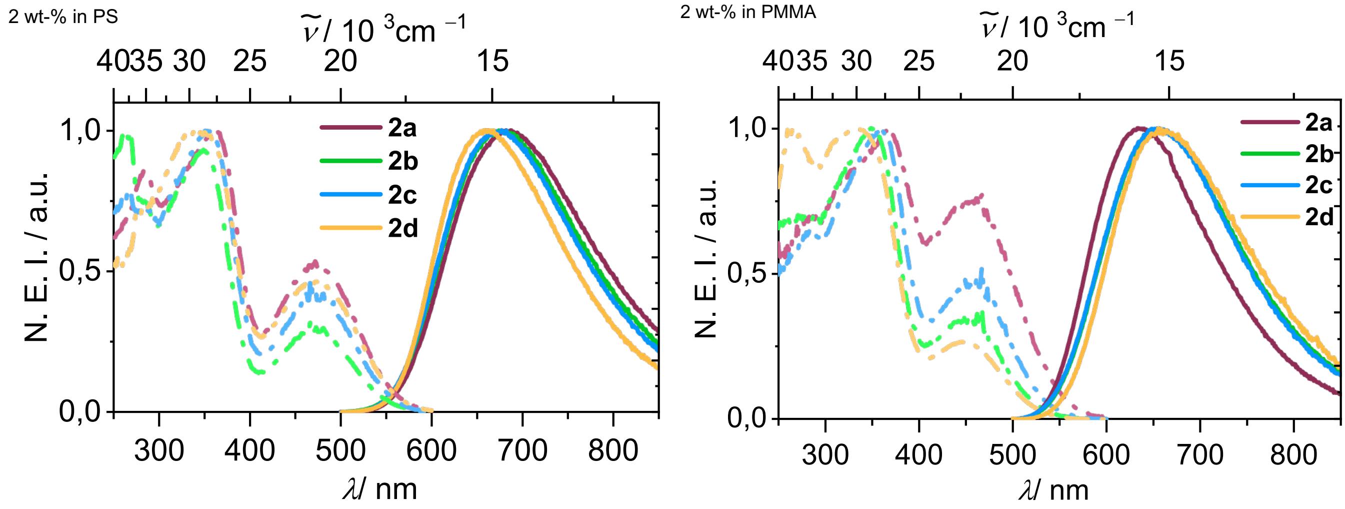

Figure S109: Emission spectra of 2a-d in PS (left) and PMMA (right) (2wt-% respectively) at 297 K.

Decay and observed lifetime

Solid state time resolved analysis

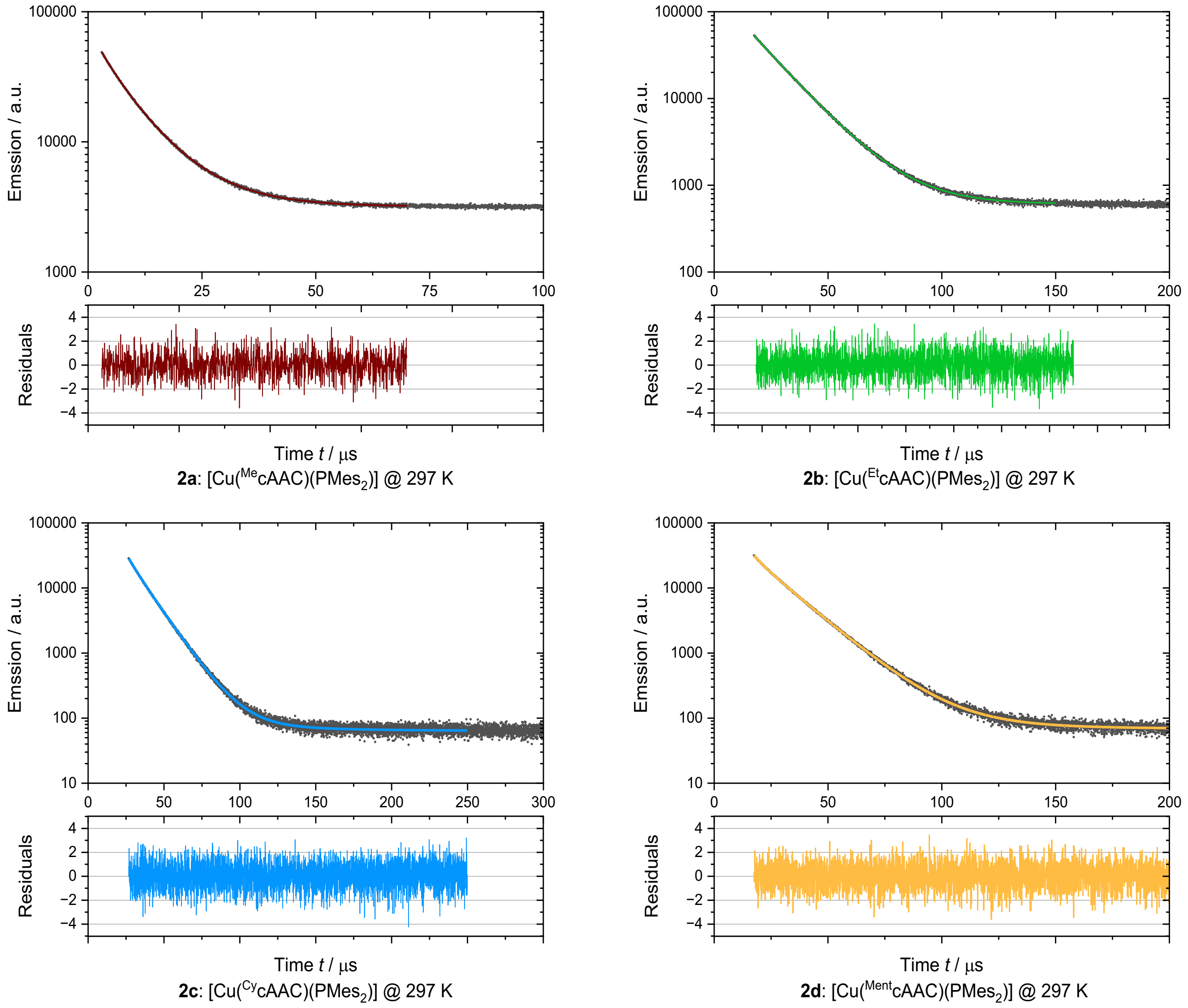

Figure S110: Observed excited states decay (grey), fit curves and residuals (colored) for 2a - 2d in solid state at 297 K

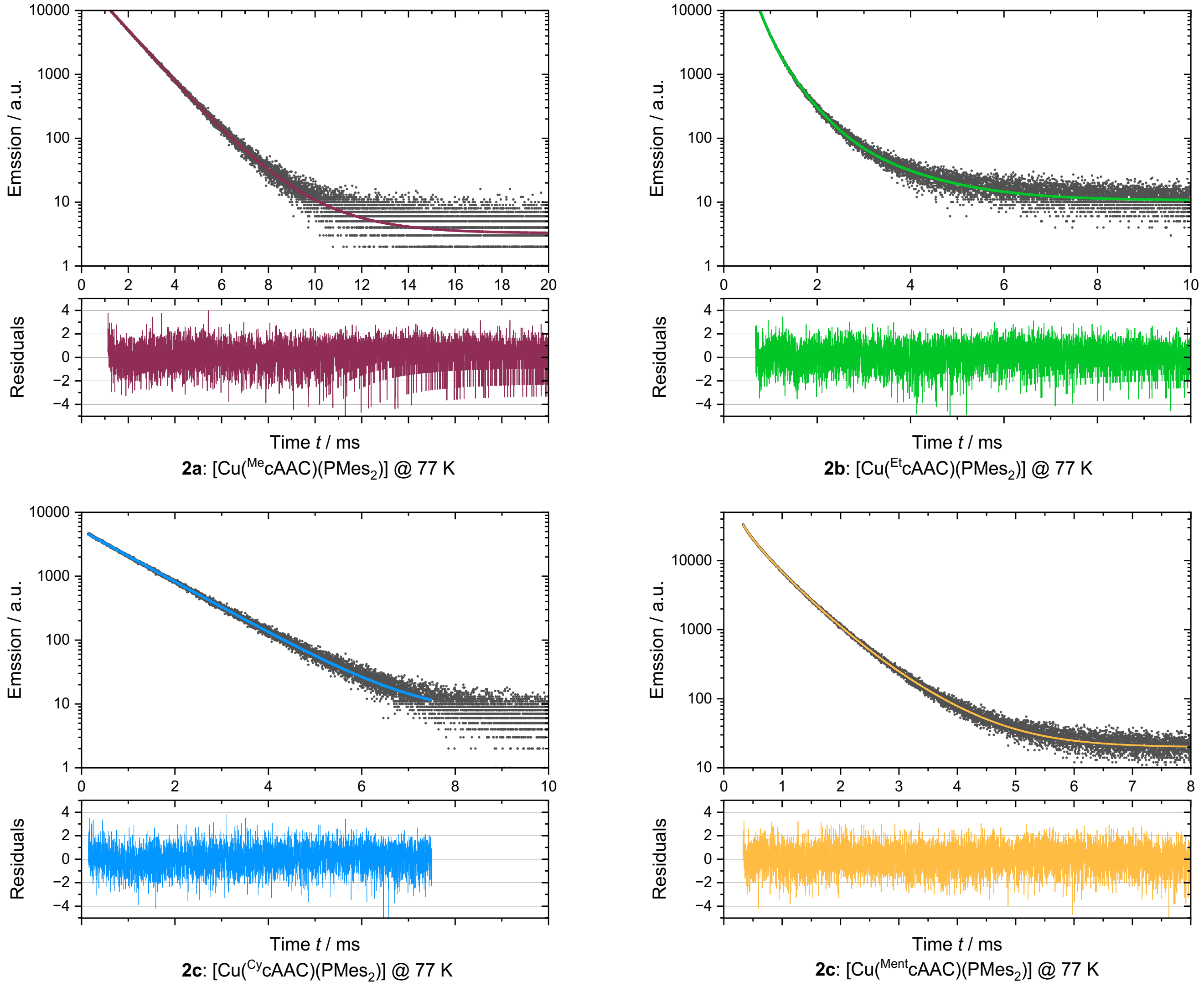

Figure S111: Observed excited states decay (grey), fit curves and residuals (colored) for 2a - 2d in solid state at 297 K

Recorded decay and observed life time fit for 2a-d in PS and PMMA film (2wt-%):

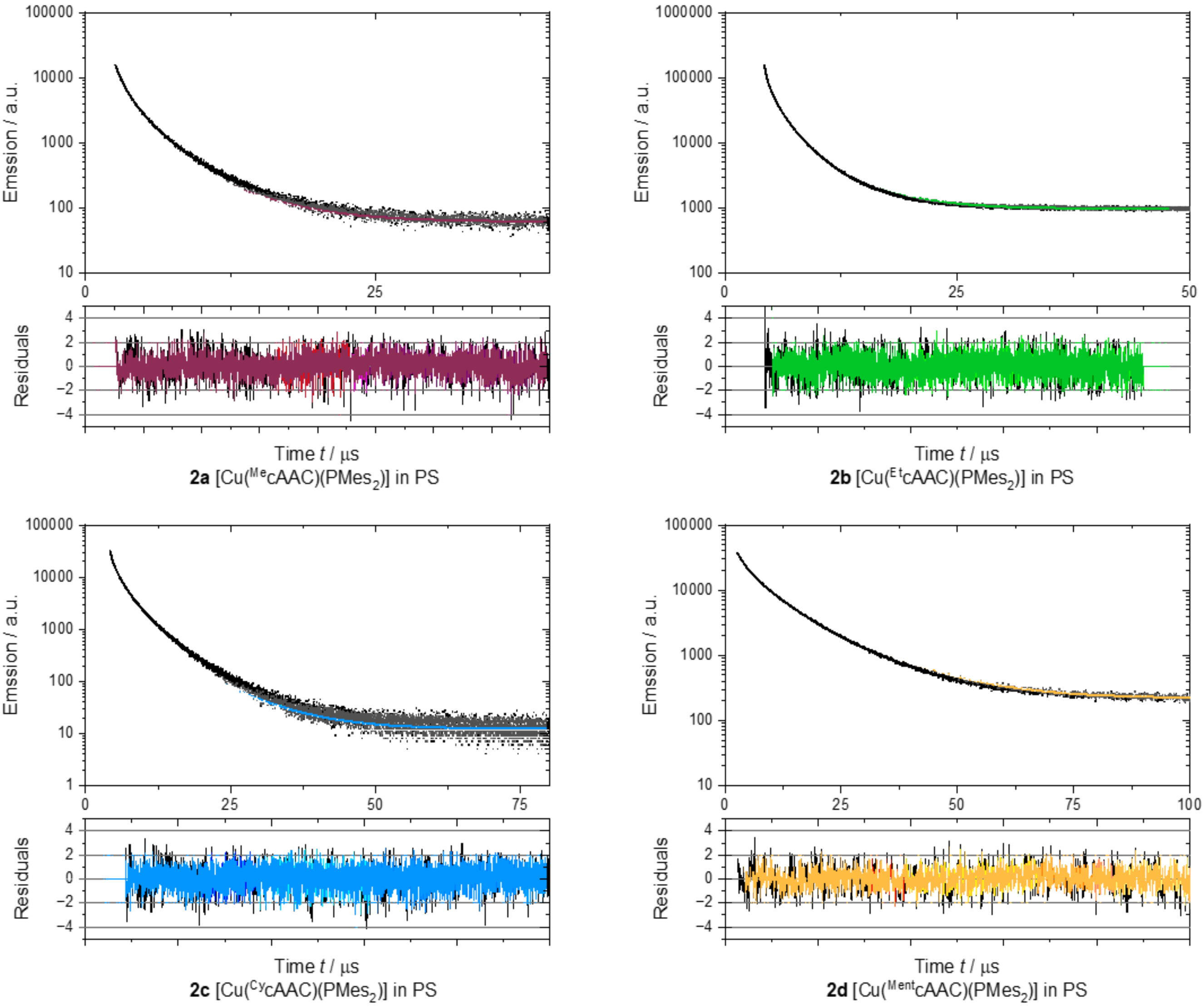

Figure S112: Observed excited states decay (grey), fit curves and residuals (colored) for 2a-d in PS (2wt-%)

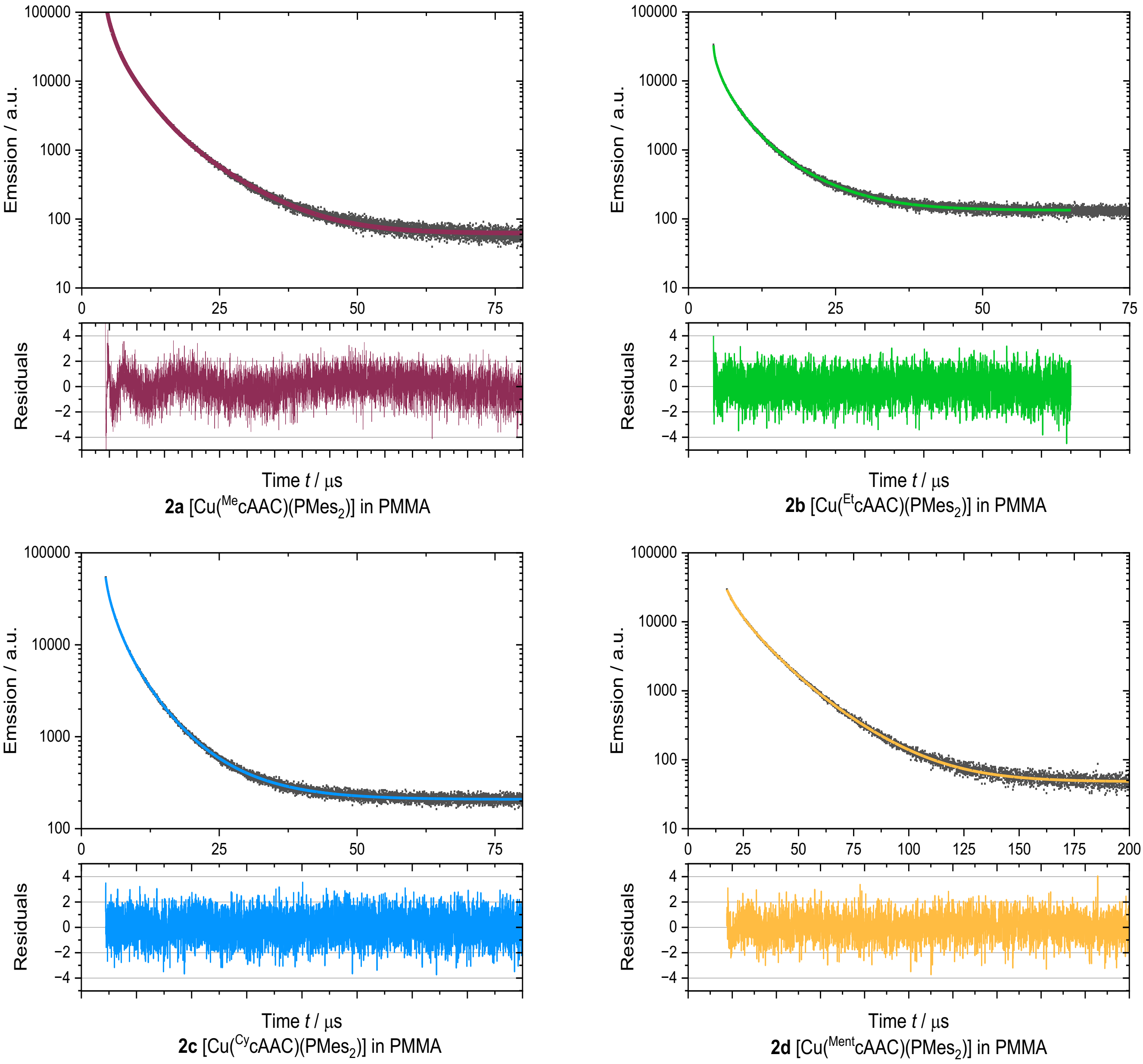

Figure S113: Observed excited states decay (grey), fit curves and residuals (colored) for 2a-d in PMMA (2wt-%)

Time-resolved VT data for 2c; lifetime components

Table S3: Fit-data for all excited states decay of 2c from 77 K to 297 K in 10 K increments

T / K	$<\tau>$ / s	$<\tau>$ / us	τ_1 / µs	τ_2 / µs	τ_3 / µs	B_1	B_2	B_3	$\Sigma_{i=1}(B_i)$	Φ	$<k_r>$ / s^{-1}	$k_r(1)$ / s^{-1}	$k_r(2)$ / s^{-1}	$k_r(3)$ / s^{-1}
**77**	0,00108	1080	1099	-	-	15430	-	-	15430	0,42	389	389	0	0
**97**	9,82E-04	982	1038	499	-	10290	1184	-	11474	0,33	336	301	35	0
**107**	9,28E-04	928	1024	667	-	8494	3095	-	11589	0,39	420	308	112	0
**117**	7,48E-04	748	868	398	-	7600	2524	-	10124	0,3	401	301	100	0
**127**	7,91E-04	791	868	486	-	10154	2509	-	12663	0,35	442	355	88	0
**137**	7,17E-04	717	797	471	-	10121	3286	-	13407	0,31	432	326	106	0
**147**	6,00E-04	600	707	412	-	10920	3866	-	14786	0,36	600	443	157	0
**157**	5,07E-04	507	143	543	1547	26887	3383	290	30560	0,34	671	590	74	6
**167**	4,13E-04	413	480	264	-	16950	4651	-	21601	0,41	993	779	214	0
**177**	3,32E-04	332	357	113	-	4270	587	-	4857	0,4	1205	1059	146	0
**187**	2,49E-04	249	273	97	-	5975	896	-	6871	0,46	1847	1606	241	0
**197**	1,69E-04	169	208	112	18	5580	2486	725	8791	0,48	2840	1803	803	234
**207**	1,34E-04	134	146	47	-	13042	1740	-	14782	0,49	3657	3226	430	0
**217**	8,20E-05	82,0	95,3	31,6	-	7330	1870	-	9200	0,51	6220	4955	1264	0
**227**	6,24E-05	62,4	72,3	25,7	-	9383	2515	-	11898	0,52	8333	6572	1762	0
**237**	4,78E-05	47,8	54,8	18,4	-	13821	3289	-	17110	0,53	11088	8956	2131	0
**247**	3,46E-05	34,6	41,9	13,9	-	13148	4644	-	17792	0,55	15896	11747	4149	0
**257**	2,48E-05	24,8	29,34	12,86	-	13200	9429	-	22629	0,54	21774	12701	9073	0
**267**	2,03E-05	20,3	24,7	7,99	-	16539	5815	-	22354	0,56	27586	20410	7176	0
**277**	1,59E-05	15,9	19	7,7	-	24925	9177	-	34102	0,54	33962	24823	9139	0
**287**	1,33E-05	13,3	15,7	5,4	-	5691	1744	-	7435	0,55	41353	31653	9700	0
**297**	1,21E-05	12,0	13	5,5	59	24492	3999	59	28550	0,55	45455	38994	6367	94

$$k_r(n) = \frac{\Phi \cdot B_n}{<\tau> \cdot \sum_{i=1}^{k} B_i}$$

4. Computational Studies

Calculations were performed with the ORCA 5.0.3 program suite[2] and the PBE0[3] hybrid functional, using density functional theory (DFT) by *Kohn* und *Sham*[4] on the Linux-HPC-Cluster of TU Dortmund University (DFG funded project – Funding No: 271512359). For geometry optimizations, the split-valence basis set def2-SVP (TZVP for Cu, SV(P) for H) with zero-order regular approximation of relativistic effects (ZORA) and Becke-Johnson damping (D3BJ) were used.[5] For time-dependent DFT calculations, the single-valence base set ZORA-def2-TZVP (ZORA-def2-SVP for H) was used as implemented in ORCA 5.0.3 and solvent effects were taken into account by means of a conductor like continuum polarization model (C-PCM).[6] All calculations were performed with tight SCF convergence (ΔE = 1·10-8 au). Molecular orbitals and transition electron densities were plotted with Orca_plot and visualized in ChimeraX with iso = 0.03 – 0.08.

1. Geometry optimized coordinates

Complexes

2a [Cu(^{Me}cAAC)(PMes$_2$)]

Table S4: Optimized Geomtry Atom Coordinates of 2a

```
94
Coordinates from ORCA-job BGK-007
  Cu  3.09691809511836     -1.38709772567185      8.47248406473082
  P   3.71438059886353     -0.03456488974166      6.84294445149775
  C   2.90150831250169     -2.62773700791920      9.89263298427017
  C   2.44499199756272      0.38093233103273      5.58697125338831
  C   4.03320574534309      1.53870078056460      7.75430055463521
  N   3.76366289253113     -2.73186895270628     10.87293983742868
  C   1.78340850373763     -3.61991343363760     10.12112308296614
  C   1.27936607754515     -0.39761747650357      5.40512071229168
  C   2.68629628850634      1.45094334794713      4.68569754635727
  C   5.37037425971581      1.81295506596708      8.12721804812370
  C   3.02144734126889      2.45495778806382      8.13312480263679
  C   3.46149144340192     -3.76384777227081     11.92852797169245
  C   4.93726617240400     -1.89784978384317     10.93235262735900
  C   0.53533313353714     -2.82227722856987     10.52727231064083
  C   1.49056410900939     -4.41281868379797      8.84742695613065
  C   2.31298958865973     -4.52794935226125     11.25220336965434
  C   0.36909579035867     -0.06882184906211      4.39541091018424
  C   0.97367080919120     -1.57805681122295      6.27911790053892
  C   1.74379580828641      1.74930170207524      3.69980569315987
  C   3.93998552352967      2.27676873517456      4.74962159300863
  C   5.66972817708061      2.97469142858688      8.84419171129432
  C   6.48564952320525      0.87392733487399      7.76381142150581
  C   3.37230053538470      3.61848286388536      8.82547760077335
  C   1.56565490566451      2.19633217244346      7.87316127863725
  C   3.03873136963357     -3.10162351098693     13.23887845955163
  C   4.67017761595203     -4.64946842322801     12.20560084411906
  C   6.11569669443155     -2.34225420535445     10.29747466884604
  C   4.86151703681775     -0.64377532652410     11.56931953419710
  H   0.70278358038785     -2.22653341295333     11.43395156460470
  H  -0.30214031106866      -3.50869572555502      10.71731009600843
  H   0.24067458426994     -2.13187190032161      9.72603200501685
  H   2.39177224548524     -4.92151577548461      8.47799690516199
  H   1.12207747993965     -3.75726639466927      8.04857136166913
  H   0.72485492444844     -5.17700038126172      9.04562265281298
  H   1.53151449479355     -4.80440991896060     11.97125715899527
  H   2.70022577371308     -5.46089806677271     10.81955679162146
  H  -0.53049884210199      -0.67795908278844       4.28134020295449
  C   0.56925804766535      1.01096847823140      3.53720971368200
```

H	0.91233575385396	-1.27899741180124	7.34079769198606
H	1.76003737431876	-2.34594923900741	6.21603420991333
H	0.02109870284344	-2.04621164343874	5.99599303692752
H	1.94503568741201	2.58378340709869	3.02380496317184
H	4.81579272871246	1.63940376499231	4.94069605420108
H	3.91028687795676	3.01367361362671	5.56504114925285
H	4.09596466492435	2.81902474446639	3.80773758256875
H	6.70851187094390	3.16522867022184	9.12413635372661
C	4.68802518439086	3.90362005393800	9.19388138099440
H	6.50329778767870	0.68191178119324	6.68142156326100
H	6.36644540584687	-0.10418778153588	8.24884225478747
H	7.45816805338725	1.28198779783539	8.07079941823448
H	2.57947609928324	4.31355780508817	9.11207783644899
H	1.31694713115644	1.14556390715177	8.08543582591889
H	1.28842299006708	2.36841970568072	6.82385655041920
H	0.93867708004614	2.84313762573562	8.50131700174911
H	2.84380463441423	-3.88335574052622	13.98511479291383
H	2.12400020646440	-2.50798122517297	13.13256110670615
H	3.83349073650342	-2.45518007266548	13.63153335197920
H	4.40311849917802	-5.38522864946088	12.97573900984267
H	5.52045764945742	-4.06295872175206	12.57809939055162
H	4.98898384452972	-5.20041662806437	11.31445488042835
C	6.16692245625929	-3.57879804950243	9.41785162936675
C	7.25452226759248	-1.53943748446276	10.40413709849322
C	3.56227569283273	-0.05720634222050	12.08646065612151
C	6.02574985110750	0.12934878731879	11.62639253437313
C	-0.42048267961219	1.35673174606386	2.45938041382110
C	5.03359702233809	5.17210150050347	9.92381042339965
C	6.27086030428534	-3.17366929453300	7.94345577458115
H	5.21746829868591	-4.11634415057106	9.53178816004005
C	7.30163691689398	-4.53032665902223	9.79994629502576
H	8.17802865397789	-1.85851194850366	9.92174864461028
C	7.21809839809545	-0.32160694526792	11.07448619181018
C	3.06230251352924	1.02419789190757	11.12801447495779
H	2.80768286991846	-0.84985255004561	12.08638393055041
C	3.67870555445874	0.47924806217612	13.51318995051999
H	5.98833670557764	1.11390993701723	12.09042826127644
H	-1.31923300044794	0.72977004029108	2.52355259782702
H	0.01101393720868	1.21639321096546	1.45683073359334
H	-0.73968700074793	2.40687237964135	2.52772758775814
H	5.96861169561176	5.06936760285619	10.49068876396278
H	4.24084158459893	5.46176448236581	10.62726586355376
H	5.16750669906096	6.01210910438037	9.22463191113314
H	5.45381338433980	-2.50115642806569	7.64615416713448
H	6.23438286653068	-4.06290698977408	7.29784965968381
H	7.21647459563861	-2.65038372146465	7.74189506756423
H	7.26601079436848	-4.81271192196931	10.86035413762908
H	8.28459910493893	-4.07666748952853	9.61214360367612
H	7.24909322463215	-5.44911428681676	9.19899903542436
H	8.11344701959123	0.29693428361607	11.13058672750412
H	2.89653250140587	0.61314173266576	10.12273990555923
H	3.78597153886431	1.84318760336544	11.01926811741489
H	2.11326651059334	1.45052486090035	11.48430727293039
H	4.07696177927108	-0.27245155672454	14.20857290451538
H	2.69235027158309	0.79456608862708	13.88118491575140
H	4.33667837687262	1.35796180343008	13.56178654030503

2b [Cu(^{Et}cAAC)(PMes$_2$)]

Table S5: Optimized Geomtry Atom Coordinates of 2b

```
100
Coordinates from ORCA-job BGK-008
  Cu  4.19226964647130      3.90319031230421      5.02162157278616
  P   5.65374397773897      5.55991741856379      5.18428833920200
  C   3.27701615707095      2.30934624930850      4.61483038285948
  C   5.61004808899302      6.20699087127778      6.90242918469883
  C   6.91783330469004      4.19620455846578      5.11350854150053
  N   2.00011809562305      2.07075646239006      4.75485251515298
  C   3.95398204690524      1.09093429833191      4.03947356203025
  C   6.79686693517776      6.55444641535635      7.58944891326132
  C   4.36352846856372      6.51527689757506      7.50524682995804
  C   7.57206610285689      4.03271078799022      3.86369303183299
  C   7.17764926614760      3.24081601050211      6.13244704865065
  C   1.51001844463314      0.73894404730142      4.25112856244299
  C   1.11064135116508      3.03653764446399      5.34564343761395
  C   4.89889259732794      1.48356241769157      2.89028070322997
  C   4.76974383528989      0.48328887466481      5.20868861966923
  C   2.78773598307694      0.18190842914986      3.59465843499784
  C   6.71612124335510      7.07641436254796      8.88561131479075
  C   8.15020182946589      6.40601274770301      6.95336297934066
  C   4.33358767731501      7.04082029638093      8.79816256069459
  C   3.06247063307641      6.33464124335679      6.77511385995251
  C   8.44242906280163      2.95860262064606      3.66142058573728
  C   7.32062320693817      4.98014220565455      2.72292753512602
  C   8.06941230636836      2.19093269653428      5.88629543315381
  C   6.47354966176181      3.26905103167311      7.45672317902123
  C   0.37752079785925      0.92662294277650      3.24739311498715
  C   1.00947412678414     -0.12981373092229      5.40074302765984
  C   0.53375938292795      4.02288446623215      4.52011391699474
  C   0.88562841872832      3.01092100940014      6.73722137065408
  C   4.28220813782817      2.38520476538200      1.82929688732397
  H   5.77752745559690      1.98889105974187      3.32022778936960
  H   5.27146901477527      0.56096356867018      2.42068171604156
  C   5.64435005423455     -0.70431063302986      4.83228654012409
  H   5.40740330098013      1.27676582249664      5.62418810786295
  H   4.07585184785440      0.19047331182624      6.01253698690330
  H   2.68218255743085      0.20154244487052      2.50235388025781
  H   2.95152743576498     -0.86580136593048      3.87595370470868
  C   5.49802533232210      7.31109977223075      9.52190102864513
  H   7.64407393950984      7.32148879174072      9.40788283401107
  H   8.89869342056573      7.00380242387803      7.48988347264087
  H   8.49994512856148      5.36344965311491      6.93689246528176
  H   8.12430805509229      6.73582022762111      5.90439100216747
  H   3.36318759723080      7.25555789145868      9.25260471039037
  H   2.89904833471606      5.29230248252234      6.45202815737746
  H   2.21149599751728      6.62724797254046      7.40383479283862
  H   3.03042489585642      6.93995700910759      5.85695128538701
  C   8.71552005483791      2.02326150368240      4.66090110990595
  H   8.92549575810495      2.85319392564532      2.68730596180636
  H   7.47076963383571      6.02416858418530      3.03135843100985
  H   7.98341970479011      4.75886638225204      1.87554077686394
  H   6.28133169095214      4.91996498470945      2.36611172716494
  H   8.24797520174836      1.46385351789520      6.68246347104515
  H   6.82772813013669      4.07659311404042      8.11217060582869
  H   5.39783206569847      3.45358885493346      7.31723065048420
  H   6.60159259739484      2.31659186704068      7.98814315915954
  H   0.70429979454049      1.48952967022429      2.36565650691632
  H   0.03493286813680     -0.05922516698694      2.90579743077735
  H  -0.47947997738019      1.44576101048334      3.69697038348676
  H   0.63927171751925     -1.08096822472345      4.99592782402416
  H   1.80109634754298     -0.36035387009991      6.12297988073238
  H   0.17948663646484      0.35518985796335      5.92990663618738
  C   0.93809138453920      4.22554357239196      3.07201540118226
```

C	-0.36589887759597	4.92007683706337	5.10316930487197
C	1.68326473942147	2.14215326799151	7.69272854972886
C	-0.02900540017430	3.92713769792852	7.26654546197509
H	5.00715361158250	2.59194540917899	1.03089938421446
H	3.39508065513288	1.93797265618928	1.35605010853069
H	3.98517637970290	3.35294302707939	2.25837127980118
H	6.45340772842976	-0.40688925911875	4.15348583902832
H	6.11498674466810	-1.12357784598211	5.73176195412301
H	5.07218019156362	-1.51159079978922	4.35189591084220
C	5.43196743318422	7.89197843390139	10.90751118780409
C	9.67954854690549	0.89181461314458	4.43624714232041
C	1.82921299745965	5.46692916521409	2.95350450273563
C	-0.25755690252523	4.31661536792130	2.12399119018699
H	1.54776226183864	3.36932830967569	2.76291054689634
C	-0.66836029781994	4.85972365460937	6.45868036166364
H	-0.82099744912119	5.69362951550730	4.48548145334419
C	2.67383828021305	3.01097624693530	8.47440122568574
C	0.80588414396332	1.33201825057066	8.64754448688816
H	2.27956146523195	1.44034355016326	7.09992778339936
H	-0.22094832072969	3.92674892182320	8.33912938157848
H	6.38587659861139	7.77508751114557	11.43819918479309
H	5.20135026155499	8.96840395366394	10.88118708617056
H	4.64975742915467	7.40960281512726	11.51041334396390
H	9.77449345617439	0.64964426916224	3.36937524806735
H	10.68624635398659	1.14458138821428	4.80392082769681
H	9.36295700637292	-0.01915934291536	4.96305140402799
H	2.16608376131398	5.60329407960861	1.91591325173419
H	1.29004199958522	6.37513213811918	3.25829901379629
H	2.72319023610436	5.38182888043794	3.58974291516225
H	-0.86045228156355	5.21448131806858	2.31814723273781
H	0.08816086460596	4.37874977364307	1.08262842857381
H	-0.92074876465911	3.44555511540812	2.21139160367588
H	-1.37606344672330	5.56552634945385	6.89310236531252
H	2.15288083168889	3.74541101894015	9.10482860825465
H	3.30354885020141	2.38976834820367	9.12664886737475
H	3.33519657849877	3.56813404181153	7.80052625915206
H	1.42798422492921	0.66896397275475	9.26491276935137
H	0.24934161458675	1.98493293528712	9.33405663291150
H	0.07439773043130	0.71097604465857	8.11414402571670

2c [Cu(^{Cy}cAAC)($PMes_2$)]

Table S6: Optimized Geomtry Atom Coordinates of 2c

```
101
Coordinates from ORCA-job BGK-009
  Cu  4.58927588139417      5.25884031378015      7.34766527640615
  P   4.07101263823695      6.70385403861689      8.93108202717995
  C   4.71121306780097      3.95460862454314      5.97595830379981
  C   3.75169045038245      8.24088365301822      7.96011136547111
  C   5.39090795322631      7.15393248291197     10.12253998576487
  N   3.82776739711241      3.83161185446191      5.01744253867280
  C   5.80282198338968      2.93559321665148      5.77025409454314
  C   4.76662481762521      9.12222489931240      7.51098683362053
  C   2.40907808944555      8.52316050846416      7.61690281439794
  C   5.19721751714962      8.27099613236409     10.97709052898581
  C   6.55254470545182      6.36845962042108     10.30157391489155
  C   4.09135109898722      2.75714786583684      3.99696138123805
  C   2.66259362240577      4.67655107117030      4.95865310013792
  C   7.07706970857880      3.70513495194867      5.35539720780956
  C   6.07673739101656      2.17993916884020      7.08071939610299
  C   5.25883953910938      2.00868153279058      4.66162938470497
  C   4.41327270025146     10.26410599334162      6.78577547787170
  C   6.22567396854900      8.84478332468772      7.73116991054863
  C   2.10607241363965      9.66198741628634      6.86434114477502
  C   1.29120450784055      7.61698843788338      8.04840226024094
  C   6.18014223174641      8.60543358079560     11.91059291931227
  C   3.95254382784540      9.11103579639022     10.91956052692756
  C   7.50489230962815      6.73539611459759     11.25802012186455
  C   6.80873808029827      5.13961329190694      9.48045206632835
  C   4.48140323811161      3.36176981287762      2.64948507253287
  C   2.86755463113470      1.87180926668000      3.79566382251814
  C   1.50167001589078      4.27589560422014      5.65254958486384
  C   2.73064669582248      5.90056674232580      4.26507627312469
  H   6.90693250495384      4.22345379878376      4.40023920848841
  H   7.25319933410672      4.49490925580735      6.10255642586966
  C   8.30474081718939      2.80336730859555      5.26859698437586
  H   6.25082806783038      2.91778990664031      7.87790700653889
  H   5.17717833259787      1.61678728117431      7.37221118969353
  C   7.28675218175975      1.25833099478009      6.97347196014045
  H   6.02074965750217      1.73112571496589      3.92327622097436
  H   4.89145357926540      1.07406527339598      5.10792507065376
  H   5.20833023739497     10.93294859923758      6.44745688319101
  C   3.09055522790820     10.55915136345473      6.44892342766503
  H   6.44572852507697      7.78186401806349      7.55058160621668
  H   6.84222924385067      9.45445000303380      7.05719384250211
  H   6.54243786681937      9.05092446049799      8.76295293153992
  H   1.06222639673179      9.86002357873949      6.60981581358315
  H   1.30723265342423      7.46039359085179      9.13651188113687
  H   0.31569066662068      8.03104710879390      7.75963748764167
  H   1.37844222131227      6.62166233929380      7.59216828613641
  H   6.01464060936360      9.47530878531435     12.55074637969769
  C   7.35168081276366      7.86085035717203     12.06583482739017
  H   3.06300378197056      8.47967932212353     10.77951367746390
  H   3.83326640710940      9.69020704360619     11.84475983171288
  H   3.96686038126856      9.81724658816230     10.07691948279966
  H   8.40042477810725      6.11981939782582     11.36965205336965
  H   6.82528146089539      5.37981342871236      8.40278942963520
  H   7.76807279192567      4.67462527802875      9.74602944060018
  H   6.01795853748928      4.38676991198715      9.62061430259640
  H   4.64532078688527      2.54881271258553      1.92960063012827
  H   3.68416427935341      4.00354464024581      2.25391519262158
  H   5.40691585469242      3.94620922379127      2.70845627036029
  H   3.10359285579190      1.09821251954573      3.05290726528008
  H   2.57244892821548      1.36695315753800      4.72176212891222
  H   2.01091224144885      2.44948652350206      3.42356764543936
  C   1.46675176739635      3.08119774990719      6.58897567035611
```

C	0.36896733522421	5.08771821403393	5.54854898420099
C	4.02057851868122	6.44960746131324	3.68709200742128
C	1.57354073543753	6.68458956358073	4.21165021983439
H	8.18798052602904	2.08489939177256	4.44034934081302
H	9.18549493193902	3.41274869314384	5.01959962759693
C	8.53303373200701	2.04224064615847	6.57151584745358
H	7.44710623655340	0.74841534308734	7.93423031702274
H	7.09391505402858	0.46432454735091	6.23244993287973
C	2.74752804242447	11.80089879724233	5.67308829373179
C	8.38955337660979	8.25272134446680	13.08092835031547
C	1.42617622828783	3.55497051903756	8.04578034779961
H	2.40264528735597	2.52314779251354	6.46360783205511
C	0.30429135792383	2.13217850061090	6.29427555970992
H	-0.54072720536074	4.80295351762259	6.07648026121852
C	0.39573853225274	6.27365405553193	4.82285580945536
C	4.56458906549135	7.55232449174835	4.59579264739658
H	4.76423963041683	5.64666968653393	3.69020139533918
C	3.86670688955241	6.94159743388928	2.24816048780161
H	1.60601163268706	7.64726797758284	3.70335630805644
H	9.39897647687682	1.37136236873161	6.47640477069750
H	8.77881092595281	2.76024579391456	7.37137896934405
H	1.71833206914662	11.76585575626775	5.29231711937243
H	2.83537111413906	12.70176558860671	6.29951535870702
H	3.42056014563449	11.93968698564508	4.81496729133805
H	9.25267215931988	7.57498860300051	13.05475420563267
H	8.76081134383593	9.27273255250067	12.90302538873792
H	7.98166797753015	8.23288648876867	14.10258203909356
H	1.47485605383331	2.69604642127585	8.73037607098923
H	2.26402884502999	4.22695576752975	8.27979768765382
H	0.49691277518861	4.10268022052173	8.25822603660176
H	0.36881457044519	1.23919115618301	6.93180065004960
H	-0.66393643294224	2.60856816333027	6.50146323500880
H	0.29357014773121	1.80431155441913	5.24644827985903
H	-0.49416799536324	6.90024567216430	4.76898031314266
H	4.75139568161780	7.16847524328858	5.60832807600347
H	5.51020012067916	7.95189893192472	4.20136398799870
H	3.85697486239454	8.38594917880306	4.69911151952343
H	3.43626445823447	6.17358252221066	1.59070105303145
H	3.22000185853362	7.82815081202070	2.19134247378176
H	4.84526866354659	7.22994568838787	1.83949411398269

2d [Cu(^{Ment}cAAC)(PMes$_2$)]

Table S7: Optimized Geomtry Atom Coordinates of 2d

```
113
Coordinates from ORCA-job BGK-010
  Cu  3.91473864803517     9.57116957471309    -1.51866146766985
  P   3.79900140490721     8.55228252489826     0.45185777365577
  C   3.68663645158361    10.42549024933309    -3.20674721043846
  C   5.44044709571137     8.17030118578944     1.18525414246092
  C   3.12629197594910     6.93224333958014    -0.14466595939599
  N   4.34985911906695    11.47912220841716    -3.61290393529947
  C   2.77090995733848     9.96994584402220    -4.31946700449953
  C   5.64102273858519     7.03095643299412     2.00544079874222
  C   6.50871169633062     9.09365871621863     1.06053908792876
  C   3.88153223810690     5.95403838473878    -0.83376367356671
  C   1.75247723800175     6.67654680230824     0.09868846588668
  C   5.39355350450549    12.08712413286784    -2.83091746324917
  C   3.98426934597612    12.04014982574333    -4.96270466060087
  C   3.37680646545439     8.65910867549498    -4.88477986604581
  C   1.30570459966332     9.70739261610839    -3.88091014042198
  C   2.86805769170621    11.07686189753838    -5.39625326335572
  C   6.91548627423769     6.76813393478202     2.52011475247725
  C   4.52370601564454     6.10312820213670     2.39692845590236
  C   7.76290666907831     8.78885847794319     1.59332254146579
  C   6.31169253428619    10.43841226362671     0.43105451608896
  C   3.27119567229556     4.75053663688688    -1.21219759958073
  C   5.32634916885659     6.15275897577415    -1.18360941074969
  C   1.18192269231643     5.47242585514880    -0.31629830151094
  C   0.88849051071566     7.67557603100257     0.81810196966866
  C   5.06650499915422    13.08730048789319    -1.89515642535253
  C   6.72068656779481    11.64605432674400    -3.01456768556769
  C   3.49294147859811    13.47725976366082    -4.82117558504514
  C   5.17196316827933    12.02550405098046    -5.92160998930909
  H   2.85741685051206     8.45226418605338    -5.83664277139017
  H   4.43426187182139     8.83155017526026    -5.14454579467672
  C   3.24719715367822     7.43711954807964    -3.98432705925927
  C   0.59637169331063    10.94346513666389    -3.27148104361143
  H   0.78725672871903     9.47874651555838    -4.82984352806074
  C   1.18280791453087     8.46490791831296    -2.99873936634586
  H   3.08794355628441    10.64458452023953    -6.38033752724313
  H   1.91808001470274    11.61499483580024    -5.49852126832747
  C   8.00535944660862     7.60999080350423     2.30006902360580
  H   7.05115187989046     5.87489013603939     3.13472485835016
  H   3.59171768528886     6.65966797627926     2.56751335338265
  H   4.30011731460367     5.35106352893271     1.62710927248904
  H   4.78179565488774     5.56928063465279     3.32130489600293
  H   8.57326807386096     9.51041432216532     1.46401793652124
  H   5.61689076006833    11.05191086894491     1.02354805304717
  H   7.25639901160742    10.98837813170640     0.34506778502406
  H   5.87048572430052    10.36646978594651    -0.57621145753838
  C   1.92679469494098     4.48155153970437    -0.96231858777346
  H   3.87424056483640     3.99968792600742    -1.72836344594156
  H   5.98895337859227     5.96184769551039    -0.32715986820527
  H   5.62640576988574     5.48525387357559    -2.00232182730659
  H   5.51456712401078     7.19321334103318    -1.48047760253094
  H   0.12286694840066     5.29647036075060    -0.11415619996495
  H  -0.12947254124000     7.28651969535252     0.95454337877205
  H   1.30948505997014     7.92631840210265     1.80211597176373
  H   0.81988055160611     8.62678201055706     0.27186735953133
  C   3.63941077797303    13.41448340161648    -1.49786442319277
  C   6.11888152470040    13.72335620335561    -1.22811137068786
  C   7.73349681298685    12.31698786563809    -2.32441701808553
  C   7.06265306002590    10.40513274374499    -3.81857468492290
  H   4.27512535502506    14.12749864616304    -4.40844303265547
  H   3.22133300777544    13.86317192881988    -5.81271992324827
  H   2.60637059295247    13.54506735958040    -4.17990876603900
```

H	4.86881057998406	12.49443699791020	-6.86708494042689
H	6.01935051848102	12.59619977822207	-5.52004374595430
H	5.50839762836953	11.00796414270445	-6.15053254687508
H	3.78123497822309	7.64319713315934	-3.04028489738859
C	1.78067280140210	7.21857250939380	-3.63172376590571
C	3.87794919907004	6.21231904995882	-4.62906838245392
H	1.08014005376207	11.84542441100117	-3.68025259919315
C	0.69926340432037	11.04069372338872	-1.75068662341339
C	-0.86795936173766	10.99075704025566	-3.70477149999221
H	1.69545150009932	8.64275558862478	-2.04042039128202
H	0.12535477775963	8.29272995689874	-2.74877142293090
C	9.37610841886604	7.27134702472049	2.81575726595562
C	1.29478275701764	3.17954484249191	-1.36853024588376
H	2.96387606326237	12.89307368031370	-2.18613139543653
C	3.35778576507171	12.86658397283898	-0.09485290719521
C	3.32303754030303	14.90910835611136	-1.56492729908790
H	5.89249823571549	14.50162288955195	-0.49986109917437
C	7.44165905577063	13.36561762743090	-1.45885629361826
H	8.76681463604004	11.99557712953605	-2.44897752347084
H	6.19744681370127	10.15592115468640	-4.44108037387701
C	8.26279111906454	10.58965311326954	-4.74572580117053
C	7.27087558123121	9.21680824749800	-2.87523199674721
H	1.22259969207521	6.95272977941551	-4.54778845877513
H	1.68764542655645	6.37142128514169	-2.93865770158657
H	3.38642582737163	5.97111444626677	-5.58473459743540
H	4.94783772129121	6.36928545766705	-4.83041223983050
H	3.78242976418960	5.33787848274990	-3.97195476269035
H	0.08351927346699	10.27768476586014	-1.25587512055740
H	1.72882482919652	10.90898055306144	-1.39025375722529
H	0.34484675279921	12.02225753254534	-1.40478224923579
H	-0.96350445324118	11.05481215886309	-4.79826504730996
H	-1.40490530345467	10.08858569798973	-3.37586208254375
H	-1.38559764617142	11.85736628128940	-3.26939535477453
H	9.32477522385325	6.66821375486997	3.73230254216799
H	9.94902869503494	6.68942686439383	2.07649444370728
H	9.95879554858846	8.17543587208558	3.03886432766155
H	0.38478147963069	3.34126181245668	-1.96416858791793
H	1.98313124037571	2.56910128911114	-1.96707199547863
H	1.00298343294934	2.58510365399002	-0.48968571450025
H	3.52057596865061	11.78078761170983	-0.04325920445221
H	4.01204335963268	13.33778895854380	0.65252953031656
H	2.31626440376438	13.06395660423087	0.19462902468083
H	3.89821503643738	15.47598920409270	-0.81975158661694
H	3.54569870659261	15.33617289323604	-2.55120160544028
H	2.25874532997887	15.08070562584518	-1.35155783446186
H	8.24585989151055	13.87897682500873	-0.93210485304538
H	8.14270381195517	11.45789339670895	-5.40758217545649
H	9.19654253706446	10.72442993526614	-4.18269769674186
H	8.39360091282578	9.69813693752863	-5.37463389127569
H	8.09814845294964	9.39395668579862	-2.17404579651359
H	6.36734985602691	9.02728176796861	-2.27960228393580
H	7.49323222302338	8.30410206133846	-3.44631496931057

$[Cu_2(^{Me}cAAC)_2(\mu\text{-}PMes_2)_2]$

Table S8: Optimized Geomtry Atom Coordinates of $[Cu_2(^{Me}cAAC)_2(\mu\text{-}PMes_2)_2]$

```
188
Coordinates from ORCA-job /work/smparuer/R1051-R1075/R1071/R1071-opt
  H   -4.02618226252021      7.73761127328840     -0.05931538273324
  H   -1.87279054889266      6.17465225238516     -0.01995259598430
  H   -2.88458517845993      5.04766778898918     -0.94487010830582
  H   -0.20944740016775      4.00373953999543     -0.39691423911613
  H   -2.91930318049634      7.77714622408513      1.32692105110831
  C   -2.59754579385342      5.35169619063739      0.07212262583184
  C   -3.86002081570890      7.35102406897012      0.95707256427782
  H   -3.15589444841374      9.68909643782376      3.79591640004624
  H   -0.01028063286580      5.16752604559015      0.93091901762420
  H   -5.35366790519726      5.95588969671454     -0.60793620044847
  H   -4.69848377728357      8.96501999357855      3.27171287536999
  C   -0.44204106089220      4.19814688384167      0.66230238709842
  H   -4.68957062050399      7.70184057867656      1.58615983380582
  H   -2.16090501447971      2.59817719968260     -0.59211520852147
  C   -3.81073763329576      5.82473456659093      0.88139540980384
  C   -3.90862277483723      8.92226821618887      4.03461766641408
  H   -2.69598246147900      7.36065445275764      3.18078571817982
  C   -1.95322604660148      4.19685030006079      0.86411656733147
  C   -5.14960529280559      5.36162602803009      0.29464035239260
  H    0.06058947274929      3.43539889094457      1.26650949313657
  C   -2.53215536303640      2.83481970269684      0.41728997934424
  H   -5.14607963933898      4.30623824884638     -0.00100474050686
  C   -3.24399434028773      7.53966305648496      4.11266427477075
  H   -1.48234046941174      8.32211147821727      5.12709827865517
  H   -5.97555409676793      5.53285975955872      0.99962710990256
  H   -3.62942770834050      2.84048965854805      0.38830560260400
  N   -3.49412199566600      5.19859142617259      2.24892413174608
  H   -2.22827922656713      2.03269602907542      1.10126804573064
  C   -2.41652789434191      4.40882180678836      2.31501770361516
  C   -2.21285427301969      7.50687562683992      5.23797421119197
  C   -4.26693003922508      6.42927548896962      4.27147002799919
  H   -1.66131947855474      6.56090123538642      5.22022790514970
  C   -4.39952597191459      5.34733919732679      3.37451095105404
  H   -2.68509223137448      7.61065183785664      6.22582484799353
  H    0.30435004390719      2.18026027170470      2.92099929494522
  C   -5.11935351984439      6.46897563662240      5.38324239720365
  H   -5.04323845824212      3.43187900417466      1.68657120617579
  Cu  -1.49404881793551      3.60399015232428      3.86032393860398
  H   -7.52026920093723      3.98327600643666      1.84643610625928
  H    1.05475099704132      1.14629655457359      1.67713951921938
  C   -5.39604139809970      4.35685135204381      3.55891033677926
  C    0.25021592010405      1.20040810185802      2.42426644738314
  H   -0.71269616148280      1.17053870389990      1.89164903659217
  C   -5.60527692290420      3.20294052589119      2.59378109632656
  C   -6.07827098760590      5.48713578363872      5.59994225379935
  C   -7.07796833576173      3.03841536884222      2.19624274738874
  C   -6.21563251622800      4.44569684969843      4.68846804867757
  H   -7.16587301457239      2.29615399428481      1.38816366946249
  H    1.61791327419093     -1.03935428095818      2.08228869298770
  H   -2.63570278292541      2.96402999508284      7.30709782635568
  C    0.33201274306253      0.06782779661789      3.40084240765226
  H   -7.68354935294373      2.67360317799923      3.03937950991024
  H   -3.96086956660428      2.00305745183530      3.34508197048611
  H   -6.97032635870419      3.67617763275580      4.85803581550035
  P   -1.37711461962280      1.56267018264051      5.15126603110754
  C    1.07688088506460     -1.05826513835196      3.03339893721958
  C   -5.03268484746140      1.89392838822910      3.13142600652954
  H   -4.37146769857467      3.37544934611760      7.39349212363310
  C   -0.38263251161198      0.08609496928519      4.62156672482418
  H   -5.15629809696156      1.09125726201279      2.38777827754267
  C   -3.58567515264486      2.94518530951501      6.75843019127026
```

```
H   -3.46160154611128    3.60649500956224    5.88187212617144
H   2.13654375014308    -3.40733608283132    2.33865922879926
H   -5.53423908950022    1.58306148193666    4.05763093411924
C   1.12249724462967    -2.20811745326241    3.82336481149787
H   2.91041138299600    -3.41832424959204    3.93691165701736
C   1.93622374599597    -3.40750827100134    3.41971179539648
C   -0.30084049053766   -1.06217484583507    5.45712336365963
C   -3.05187878458138    0.83027846894715    5.47588803101299
C   -3.93342378308457    1.56143460169445    6.31654833475245
H   -2.43383548171802   -0.30239091794710    3.02547015387654
H   -0.67968071986362   -0.21197302279066    7.38354768166374
C   0.41506316767773    -2.18423853886065    5.02590600149560
C   -0.93631019152649   -1.12050210619735    6.81473191363663
H   1.42130162004779    -4.34550530317415    3.67654484510332
H   0.44368459283449    -3.06288104588804    5.67839417776209
C   -2.92610732286067   -1.03228348631871    3.68320411673986
C   -3.56770016845173   -0.34592832778818    4.86159603650300
H   -0.58689094051541   -2.00611403324487    7.36485722325457
C   -5.17226741100463    1.03271806726018    6.68312732970401
H   -5.81290600022670    1.62081865221199    7.34757217965550
H   -2.16074423143266   -1.76950514630200    3.96602824064198
H   -2.03424049408969   -1.15176688940391    6.76260557029631
H   -3.70187065737311   -1.55525812789895    3.10522817256917
C   -4.80808161877666   -0.84980703139897    5.27686948858305
C   -5.61375059092611   -0.20829016509004    6.21848407337004
H   -5.17464159889066   -1.76559660271534    4.80195995620166
H   -7.64937155204394   -0.02350697767874    6.92577411256842
C   -6.91453950502200   -0.80524379483984    6.68107832256455
H   -7.34965252018019   -1.46124742954140    5.91254404860120
H   -6.77286769057609   -1.41481961423924    7.58961148904901
H   -5.02008124425973    7.28953283983262    6.09647437698251
H   0.41665836953459     9.30800558108228    9.57834929704420
C   -1.29586466408212    4.47952708461598    11.06876484149388
H   2.56067775722728     5.86265576812036    11.44230767840656
C   0.03598271304079     4.77419331627124    10.79785613769926
H   -2.81206273984002    2.95722784415147    11.08850883226164
H   2.85140988508474     4.28032888803471    12.19924761710269
C   0.83567012963475     9.45624616568996    8.57128367263809
H   1.85023563136031     9.86774258758499    8.67607841698372
H   0.21924240992528    10.22340202985667    8.07285493233695
H   -0.96358759147644    7.38058958561018    8.66347782472306
C   -1.76907023178150    3.18935898298061    10.86543830555939
C   2.99110122284587     4.86352969357807    11.27683469182843
H   4.07134142188220     4.99694590461238    11.11250644251094
H   -0.84398323364333    4.10735310941575    7.74685868853071
C   0.90950875620761     3.79822615904120    10.30803719273750
H   0.73540357593252     0.17593934972455    11.88712005122666
H   -2.93756352802553    0.78320429031320    11.86717782060603
C   -0.16914647968390    7.22324810728132    7.92749005089140
C   0.84556564667545     8.17234332050139    7.78753417733105
H   2.00029846400857     5.97701465619056    8.87514772451807
H   2.57066426099241     1.99322691936371    11.89537849036132
C   -0.94287426570475    2.17315469758107    10.36583347897918
C   2.35942517844248     4.17571818044368    10.05926619610798
C   -2.05921031372917    0.22150140815779    11.51479650958260
C   0.39748722548912     2.49957288072251    10.06473420578744
H   1.99461648587310    -1.05392768237677    11.65464261286106
C   -1.27866181788765    5.06290981135411    7.38907480722184
C   2.53410101619839     5.02067646698924    8.80049114007172
C   1.31451449108524    -0.36777604046515    11.12870944614484
H   -1.80702965884794    4.82753647424895    6.45343172979244
C   -0.20316265806443    6.06544272356205    7.15058956597199
H   3.60133701995004     5.23411946562678    8.63194022098236
H   3.60830779634771     0.55433210594822    11.86204418104522
H   2.90954680978758     3.25028695459497    9.88078911260928
C   3.11507105280718     1.30765328374220    11.23067758884535
H   -2.36689693602512   -0.82728097067653    11.38473078018497
```

H	0.63322024576378	-0.97440186605503	10.51947990190707
C	-1.55031861495884	0.79263854048788	10.18136692902240
C	1.88608864109553	7.85698712244064	6.91260792796528
H	-2.00673041735855	5.40380392272364	8.13652375041916
H	-3.48140861762650	1.49809194367568	9.42761526622930
H	2.75011425858578	8.52702682985425	6.85809058881830
H	2.14798229512301	4.48707023827530	7.92214170418475
H	3.90137822049375	1.86484466520494	10.70864143159263
C	2.15993074632297	0.57866319190210	10.27753171880243
N	1.28213054736389	1.52827973226367	9.44382848622183
H	-0.77063320148094	0.13076518818106	9.78725583137035
C	-2.67772937314196	0.80057094244961	9.14981699265438
C	0.79401335794449	5.80917701034464	6.17134742608719
C	1.91451112874616	6.68665985312467	6.13976597144734
H	-2.29641351511584	1.09360701376550	8.16592037933973
H	-3.11915058895911	-0.20222842468379	9.05127305495026
C	1.46839551288954	1.50815829804058	8.12092707271161
C	2.88718778047657	-0.17911811565439	9.16087635212508
Cu	0.46112937463864	2.35849610056825	6.65965299379908
H	2.41923122042358	-1.16501878089051	9.01918668120070
H	3.94480719000133	-0.34646304492954	9.41046778975267
H	4.01730028485182	6.97448774860809	5.89161629120847
H	0.66423986712205	7.26492093709967	4.00898739755246
P	0.54933794759894	4.27560145269436	5.15835262948264
H	-0.49390555216907	6.19010660042538	3.23255516237470
C	3.20905957458793	6.39294656280773	5.42546411930433
H	4.04483813450750	2.26883966626854	8.57090081958287
C	2.71573169861183	0.64824132455221	7.87150562876066
H	3.46451407441535	5.32654275015535	5.49199256455160
C	0.48657908967157	6.67638261019530	3.09669363502027
H	3.18789618610420	6.64272255937514	4.35480210245660
H	0.43991149383646	7.36622696060498	2.24169979017218
C	3.89018384873777	1.63773871930985	7.68619460662493
H	1.89335995718277	-1.06147498491711	6.81238521053123
H	3.71560213937552	2.29950443679010	6.83013030499352
C	2.62947024152396	-0.26567910259588	6.65451495303364
H	4.81376291808671	1.06567244380544	7.50435452487792
C	1.69791722301677	4.50110967835123	3.71912543889570
C	1.54711933071418	5.63811153513048	2.87193001202189
H	2.43053006412196	2.39372156885104	5.17275652148175
H	2.33553651162085	0.27478313176626	5.74721353764314
H	3.60731021067235	-0.73956061845691	6.47339819166680
H	2.61562481820985	1.41403251364952	3.70920248434667
C	2.96124986155405	2.33049088128998	4.21165713040467
C	2.69582371624485	3.55450221873237	3.38614917582949
C	2.39712953791761	5.80432848168577	1.77577019654604
H	2.26967663732626	6.69340875262035	1.14987841409647
H	4.03878661599301	2.21133564902224	4.40325615319729
C	3.50528326297067	3.75181139709526	2.25895147482951
C	3.38283777515531	4.87185283811277	1.44035576668924
H	4.27259818292447	3.00501375912626	2.03156595051750
H	4.70898457216721	6.07292500015184	0.22383504321355
C	4.25831898855577	5.06815573740886	0.23245445481093
H	3.67924950334644	4.96536262693233	-0.70012855860910
H	5.07030989091384	4.32791721290497	0.20588336939606
H	0.41030770839718	5.78601996955986	10.95999634186960
H	-1.96556558198235	5.25686772899288	11.44227537967878
H	-6.72119730074902	5.53386180610735	6.48121740155504
H	-1.29928112910004	0.26340288649058	12.30737076904997
H	-4.36213700542543	9.19465285675075	4.99975593310791

[Cu_2(^{Me}cAAC)(η^2-HCCPh)(PPh_2)]

Table S9: Optimized Geomtry Atom Coordinates of [Cu(^{Me}cAAC)(η^2-HCCCPh)(PPh_2)]

```
 90
 Coordinates from ORCA-job R1092-opt
   C   -6.37243974765264     -1.53583386584131      2.20746255694669
   C   -5.00677288923253     -2.02999271488435      1.71878381670734
   C   -7.39056801187226     -1.96494033487653      1.12543017242063
   H   -6.35476174570689     -0.43933697394466      2.29217063837048
   H   -6.61726019430584     -1.93478152674868      3.19978395424874
   C   -6.53736269785805     -2.21014632103810     -0.10742479054006
   C   -8.36321674386318     -0.82809898821409      0.78773767785193
   C   -8.20093080879791     -3.20431163158138      1.51612597726573
   N   -5.27087255222434     -2.09751801776838      0.23796944342689
   C   -4.69405143632518     -3.43296109065492      2.24464965916353
   C   -3.85168438021960     -1.10664668655011      2.07966494394064
   Cu  -7.20312068040106     -2.93360272164729     -1.73299480009656
   P   -8.49858466818189     -4.58461347565989     -2.50949016331290
   C   -7.54727897099230     -5.63498275174441     -3.65022968664097
   C   -8.68866461715068     -5.63144675891453     -1.01620520247541
   C   -7.60715894700396     -6.22443031270338     -0.33953236645771
   C   -9.96489670006838     -5.77597897600649     -0.45348289846748
   C   -7.34642551606730     -7.01582437957427     -3.47666199675050
   C   -7.03043208227494     -5.04898855396177     -4.82339169744002
   H   -10.81949407851545     -5.33821616554786     -0.97111469206021
   C   -10.15504372150621     -6.45917941534140      0.75080822307709
   C   -9.07094518335931     -7.04018225816288      1.40576929326424
   C   -7.79339792951850     -6.92795907309822      0.84710204708351
   H   -11.15807019712092     -6.54775076215572      1.17001203527865
   H   -9.21724750905767     -7.58214239016375      2.34040271734029
   H   -6.93681187954123     -7.38170360226723      1.34839545416415
   H   -6.60508075036442     -6.12072544970068     -0.75407528163381
   C   -6.63089579454298     -7.76464349862429     -4.41100709644165
   C   -6.31566335757938     -5.79672583877690     -5.75405405275211
   C   -6.10037002915097     -7.16279517489707     -5.55218090568139
   H   -7.18906710981579     -3.98560790893286     -5.00235726493355
   H   -7.75651876351326     -7.51450715301389     -2.59928791295286
   H   -6.49149104866672     -8.83389605521301     -4.24377185841660
   H   -5.92489086304122     -5.30783052710928     -6.64783538536369
   H   -5.53980128864119     -7.74968238386323     -6.27992644166031
   C   -4.24052984940213     -1.95938375411443     -0.75358851869051
   C   -3.57778075951530     -3.07842641920899     -1.28938192206622
   C   -3.97536675081572     -0.64996149973890     -1.21512537782293
   C   -3.00965934625363     -0.48408031230705     -2.21030138167142
   C   -2.61189255031149     -2.85424924220999     -2.27984135137789
   C   -2.32390981450170     -1.57414044143565     -2.73648350232005
   C   -4.73229936965472      0.56786678587154     -0.71228371764645
   H   -2.79859215916300      0.51550309978414     -2.58876796713700
   H   -2.08655261839742     -3.70697379750204     -2.70914796409960
   H   -1.57330736869900     -1.42617223484444     -3.51265781906119
   C   -3.88238699277876     -4.50904693869567     -0.89270040454247
   C   -5.77295089384405      1.03583744032842     -1.73066604565791
   C   -3.79880037230546      1.71550216019822     -0.32185154514410
   H   -5.28707424749786      0.27310865174787      0.18551858039907
   H   -4.70129042017034     -4.49091637237066     -0.16340187009666
   C   -2.66587393989815     -5.17655347134170     -0.24460930151037
   C   -4.36445503767160     -5.32335527316030     -2.09500223239413
   H   -5.29920066797561      1.30675050822530     -2.68505999584024
   H   -6.30778748714984      1.91944220174523     -1.35349486408241
   H   -6.50728245585624      0.24911236311261     -1.93664397040864
   H   -3.30350741485757      2.15368270215737     -1.19929268361782
   H   -3.01301128749819      1.38965060444340      0.37298753128204
   H   -4.37072911035163      2.52046797006024      0.16069362737394
   H   -2.26010949058959     -4.58509597964385      0.58709885638116
   H   -1.85498885118300     -5.31205493358311     -0.97441222540366
   H   -2.93205385328618     -6.17089415133406      0.14039153081899
```

```
H   -5.28404256577111     -4.90873015841818     -2.52814669537764
H   -4.58588446894280     -6.35861630219390     -1.80209986459133
H   -3.60608464403129     -5.36949562987860     -2.88931480975549
H   -4.04897311541886     -0.06244746045287      1.81711761293810
H   -2.92085938832810     -1.41919648399349      1.58742718540637
H   -3.68786973164642     -1.15003818979472      3.16458576442080
H   -7.83317217485638      0.07557018044186      0.45577043516117
H   -8.96190228611740     -0.56910480611950      1.67369729659404
H   -9.04742398580073     -1.13058570071089     -0.01644788561149
H   -7.57526219944463     -4.08217735709853      1.71202794639412
H   -8.89557578438033     -3.48590399345376      0.71645382256494
H   -8.78681390414341     -2.99133666093815      2.42199936799650
H   -5.46825288485310     -4.15629826313770      1.96413984993756
H   -4.63510557734889     -3.40441737317609      3.34111823487433
H   -3.73065929188965     -3.79586376411229      1.86891067336917
C   -5.96367001000449     -2.00778716826084     -3.70636266663142
C   -6.98321218459342     -1.38459313655219     -3.94774984580129
C   -8.17571647018111     -0.63935850111883     -4.19572354020642
C   -8.08975030806398      0.71263923913847     -4.57370185906020
C   -9.43962450939083     -1.24019895074778     -4.05602638057055
C   -9.24705088907792      1.44765231216337     -4.80993758197940
C   -10.59081331872456     -0.49424271213575     -4.29356228943904
C   -10.49984413560256      0.84677295902013     -4.66998698751826
H   -9.17173945513179      2.49416641842198     -5.10490013618389
H   -11.40519595042493      1.42466763997932     -4.85567784695995
H   -7.10888557955987      1.17346292661929     -4.68143285154164
H   -9.50527111045180     -2.28704573212864     -3.75464380388961
H   -11.56617439049594     -0.96771670393446     -4.18388615827991
H   -5.02555368155800     -2.51793255442645     -3.58143656839514
```

[Cu(^{Me}cAAC)(η^1-CCP)($PHPh_2$)]

Table S10: Optimized Geomtry Atom Coordinates of [Cu(^{Me}cAAC)(η^1-CCCPh)($PHPh_2$)]

```
90
Coordinates from ORCA-job R1093-opt
  C   -6.44917802339712     -1.49821015035238      2.26885992082115
  C   -5.05581795986825     -1.87354007583430      1.75538079590804
  C   -7.44471706893009     -1.97529692791589      1.18614681500824
  H   -6.51372389681520     -0.40690627042854      2.38478751995619
  H   -6.65124907873509     -1.94042245454759      3.25275114771526
  C   -6.58772048435596     -2.19533762992615     -0.04944304733437
  C   -8.46166520628809     -0.87920538715697      0.84253882480691
  C   -8.20094458183097     -3.24341216552929      1.58894079471866
  N   -5.33135005794163     -1.97512749777208      0.27899812664279
  C   -4.60567962768268     -3.24395445442282      2.26606077444241
  C   -3.98772759481224     -0.84732335425299      2.10417085641166
  Cu  -7.14910166950311     -2.75687401578546     -1.78771520449549
  P   -8.21647470108340     -4.73665929536211     -2.02813351245942
  C   -7.75142430738739     -5.78443878979625     -3.44574886791474
  C   -8.40641340624549     -5.92497403418224     -0.66605077762303
  C   -7.26421260519909     -6.32800056329707      0.04018458120342
  C   -9.65626713892753     -6.40750473374186     -0.26075883741607
  C   -7.82729430982293     -7.18225385325660     -3.40948919547140
  C   -7.27721878486566     -5.14508103421385     -4.59946342020096
  H   -10.55405768721944     -6.10877345610033     -0.80261591522148
  C   -9.76363731218987     -7.26775333721862      0.83331903289032
  C   -8.62028442130069     -7.66766256284744      1.52447202567422
  C   -7.36732938892056     -7.20027587705992      1.12052010058902
  H   -10.74429818103687     -7.62989816824176      1.14214552820847
  H   -8.70405784144533     -8.34225156665845      2.37639955260738
  H   -6.46917705515370     -7.50829799989143      1.65614084496739
  H   -6.28825842203214     -5.94314472124528     -0.25493724460543
  C   -7.43943693357580     -7.93296680504969     -4.51866412142757
  C   -6.90040357770475     -5.90105739966741     -5.70861685303185
  C   -6.97807753913706     -7.29390391244994     -5.67064976998063
  H   -7.18633569831287     -4.05646380951182     -4.61698139297724
  H   -8.18643221591453     -7.68706000652291     -2.51298669520634
  H   -7.49903976809958     -9.02096179666188     -4.48232255043015
  H   -6.53238602796482     -5.39466866229701     -6.60091393317502
  H   -6.67431976035098     -7.88258776196108     -6.53654503160946
  C   -4.28226950630731     -1.79959356023140     -0.69103295683287
  C   -3.55842659547322     -2.89515458946059     -1.19270519982552
  C   -4.02127327976035     -0.48247526999983     -1.12881552892006
  C   -2.96771650860180     -0.28413463564282     -2.02357805232180
  C   -2.50165257020928     -2.64050960572989     -2.07386356247416
  C   -2.19473116257442     -1.34783447937956     -2.47693592339609
  C   -4.88170439821245      0.70473947074929     -0.73650500317310
  H   -2.75786242879844      0.72221921290786     -2.38391593058488
  H   -1.92792886226623     -3.47749859210128     -2.47137889231421
  H   -1.37081834863043     -1.17038465963170     -3.16828146294162
  C   -3.95102474457433     -4.33435190383444     -0.93060768768366
  C   -5.81829328008666      1.08278340543462     -1.88753121305929
  C   -4.05643308292394      1.91288500816047     -0.28743654569977
  H   -5.51895435985919      0.40123574096797      0.10333893564692
  H   -4.77296109747399     -4.32648519769029     -0.20384268369918
  C   -2.81281915343585     -5.17886041665903     -0.35838011744145
  C   -4.48407937452211     -4.94638864270881     -2.23009481013350
  H   -5.24688857262412      1.40850559162404     -2.76858966493274
  H   -6.47873231788194      1.90950854917583     -1.58649812873378
  H   -6.43139635308527      0.22907900733646     -2.20090505453169
  H   -3.50855641224892      2.35985529971036     -1.12838989622640
  H   -3.31985085604500      1.65202815337034      0.48455945676768
  H   -4.71567471548868      2.69397745135334      0.11685577789981
  H   -2.39177962730105     -4.74394684939536      0.55808967911565
  H   -1.99047044052662     -5.28364227873361     -1.08003300428041
  H   -3.16834078529363     -6.19220040342873     -0.12184197679449
```

H	-5.27195196144050	-4.31789684207261	-2.66766063385408
H	-4.89270491605783	-5.95366718409016	-2.06565542079497
H	-3.68746433913706	-5.03264697062577	-2.98253285104554
H	-4.28141092577585	0.16883296583281	1.82383484186225
H	-3.03119620532418	-1.07996161479057	1.61795405561389
H	-3.82600771335397	-0.85961523588266	3.19047916448932
H	-7.96414626712811	0.03851578587322	0.50045694043996
H	-9.06595514624469	-0.63471610605460	1.72903759052427
H	-9.13584643181646	-1.20849109761466	0.04062547256089
H	-7.53409131916672	-4.09168380316613	1.78537482498068
H	-8.89152300599384	-3.55305431574713	0.79541546296657
H	-8.78914692090096	-3.05312735594068	2.49829463327589
H	-5.33424648388848	-4.02775499020463	2.02519662317155
H	-4.48787833605733	-3.20973164041777	3.35767702840618
H	-3.63815479235623	-3.52487057809607	1.83282276954730
C	-6.85767271811944	-1.93527331047663	-3.46612019963607
C	-6.45187867683480	-1.38573653057261	-4.49417850790428
H	-3.86798047399039	-0.93736972069282	-4.89677932859855
C	-5.88704591458176	-0.70073018141286	-5.60784644348677
C	-4.48938201956464	-0.52890418686587	-5.69335123809586
H	-7.76611101417885	-0.29008782911200	-6.58086105777796
C	-6.68531777191949	-0.16559579112685	-6.63774481013887
C	-3.91847931656696	0.14960600153391	-6.76574787467062
H	-2.83535412578920	0.27132346435199	-6.81167555404223
C	-6.10820265289130	0.51416911125229	-7.70703193727940
C	-4.72318621551726	0.67579026304172	-7.77905841876300
H	-6.74541828710583	0.92208383670773	-8.49263080250876
H	-4.27461059169392	1.20775372482000	-8.61824346892701
H	-9.59353428834608	-4.52792514345357	-2.30400228573457

2. Calculated Excited States and Transitions

2a [Cu(^{Me}cAAC)(PMes$_2$)]

Table S11: calculated absorption data for 2a

ABSORPTION SPECTRUM VIA TRANSITION ELECTRIC DIPOLE MOMENTS

State	Energy (cm-1)	Wavelength (nm)	fosc	T2 (au**2)	TX (au)	TY (au)	TZ (au)
1	20566.9	486.2	0.063463521	1.01585	0.05205	0.34779	-0.94455
2	27728.0	360.6	0.001828079	0.02170	0.00645	0.02485	-0.14507
3	28040.9	356.6	0.042395638	0.49774	-0.41001	0.38491	-0.42600
4	28578.6	349.9	0.060207625	0.69356	-0.46184	-0.60729	-0.33387
5	29275.7	341.6	0.195028247	2.19314	-0.83963	-0.83310	-0.89113
6	31331.1	319.2	0.181467538	1.90677	0.62726	-0.69671	1.01386
7	31655.3	315.9	0.030652042	0.31878	-0.22976	-0.44246	0.26498
8	32543.9	307.3	0.042207498	0.42697	-0.06116	0.07599	-0.64611
9	32996.8	303.1	0.000625933	0.00624	-0.04901	-0.02390	-0.05719
10	34175.5	292.6	0.005160325	0.04971	-0.08698	-0.19952	-0.04831
11	34575.7	289.2	0.007202190	0.06858	0.13367	-0.21733	0.05895
12	34753.8	287.7	0.009613211	0.09106	0.11087	-0.14151	0.24238
13	35676.0	280.3	0.001211614	0.01118	0.05726	0.03711	-0.08078
14	36944.7	270.7	0.002144807	0.01911	-0.01977	-0.01659	-0.13582
15	37219.4	268.7	0.055363095	0.48970	0.53208	-0.45428	-0.01481
16	38015.2	263.1	0.030397772	0.26324	0.46873	0.18686	-0.09284
17	38112.6	262.4	0.016869098	0.14571	-0.06865	0.12024	-0.35573
18	38784.3	257.8	0.056239492	0.47738	-0.03284	0.37521	-0.57924
19	38941.0	256.8	0.007676415	0.06490	0.00484	-0.18517	0.17488
20	39068.3	256.0	0.073410453	0.61860	-0.13605	-0.59918	0.49099
21	18836.2	530.9	spin forbidden (mult=3)				
22	24889.2	401.8	spin forbidden (mult=3)				
23	25018.9	399.7	spin forbidden (mult=3)				
24	26716.6	374.3	spin forbidden (mult=3)				
25	27737.8	360.5	spin forbidden (mult=3)				
26	28506.0	350.8	spin forbidden (mult=3)				
27	28660.6	348.9	spin forbidden (mult=3)				
28	29827.6	335.3	spin forbidden (mult=3)				
29	30245.4	330.6	spin forbidden (mult=3)				
30	31325.2	319.2	spin forbidden (mult=3)				
31	32177.1	310.8	spin forbidden (mult=3)				
32	32910.2	303.9	spin forbidden (mult=3)				
33	32963.1	303.4	spin forbidden (mult=3)				
34	33491.5	298.6	spin forbidden (mult=3)				
35	33692.6	296.8	spin forbidden (mult=3)				
36	34838.3	287.0	spin forbidden (mult=3)				
37	35038.2	285.4	spin forbidden (mult=3)				
38	35380.3	282.6	spin forbidden (mult=3)				
39	35811.5	279.2	spin forbidden (mult=3)				
40	35850.8	278.9	spin forbidden (mult=3)				

Table S12: calculated SOC-corrected absorption data for 2a

SPIN ORBIT CORRECTED ABSORPTION SPECTRUM VIA TRANSITION ELECTRIC DIPOLE MOMENTS

States		Energy (cm-1)	Wavelength (nm)	fosc	T2 (au**2)	TX (au)	TY (au)	TZ (au)
0	1	18836.1	530.9	0.000000421	0.00001	0.00068	0.00140	0.00222
0	2	18836.2	530.9	0.000000187	0.00000	0.00129	0.00010	0.00126
0	3	18836.7	530.9	0.000002922	0.00005	0.00461	0.00387	0.00385
0	4	20566.9	486.2	0.063411751	1.01502	0.05223	0.34770	0.94414
0	5	24845.1	402.5	0.000000400	0.00001	0.00001	0.00130	0.00190
0	6	24846.8	402.5	0.000001105	0.00001	0.00163	0.00118	0.00325
0	7	24863.0	402.2	0.000171644	0.00227	0.00056	0.03859	0.02799
0	8	25021.7	399.7	0.000000949	0.00001	0.00244	0.00006	0.00255
0	9	25022.4	399.6	0.000000211	0.00000	0.00125	0.00023	0.00107
0	10	25022.4	399.6	0.000000648	0.00001	0.00111	0.00127	0.00238
0	11	26719.4	374.3	0.000001614	0.00002	0.00282	0.00323	0.00121
0	12	26719.5	374.3	0.000000574	0.00001	0.00164	0.00101	0.00183
0	13	26719.6	374.3	0.000000087	0.00000	0.00069	0.00067	0.00039
0	14	27730.8	360.6	0.001680345	0.01995	0.01143	0.01815	0.13960
0	15	27740.7	360.5	0.000002239	0.00003	0.00045	0.00426	0.00286
0	16	27740.7	360.5	0.000008023	0.00010	0.00486	0.00439	0.00723
0	17	27740.7	360.5	0.000001585	0.00002	0.00036	0.00024	0.00431
0	18	27895.2	358.5	0.034323187	0.40507	0.34199	0.39554	0.36285
0	19	28491.8	351.0	0.000250196	0.00289	0.03359	0.03043	0.02893
0	20	28493.7	351.0	0.000346100	0.00400	0.04546	0.02993	0.03219
0	21	28513.4	350.7	0.037720171	0.43551	0.45061	0.29539	0.38107
0	22	28632.7	349.3	0.028023670	0.32221	0.23628	0.49917	0.13120
0	23	28663.7	348.9	0.000008894	0.00010	0.00623	0.00677	0.00418
0	24	28663.7	348.9	0.000002300	0.00003	0.00339	0.00341	0.00180
0	25	28665.9	348.8	0.001735005	0.01993	0.04465	0.13278	0.01738
0	26	29278.7	341.5	0.194836665	2.19076	0.83916	0.83331	0.89004
0	27	29830.5	335.2	0.000000024	0.00000	0.00020	0.00046	0.00009
0	28	29830.5	335.2	0.000000388	0.00000	0.00102	0.00024	0.00178
0	29	29830.5	335.2	0.000000560	0.00001	0.00143	0.00199	0.00043
0	30	30248.3	330.6	0.000001965	0.00002	0.00015	0.00436	0.00154
0	31	30248.3	330.6	0.000003901	0.00004	0.00360	0.00442	0.00316
0	32	30248.3	330.6	0.000000301	0.00000	0.00133	0.00045	0.00114
0	33	31325.7	319.2	0.110924363	1.16574	0.47332	0.55842	0.79365
0	34	31327.8	319.2	0.000000427	0.00000	0.00054	0.00086	0.00186
0	35	31327.9	319.2	0.000147304	0.00155	0.01974	0.01914	0.02814
0	36	31329.3	319.2	0.073475698	0.77209	0.39049	0.45009	0.64578
0	37	31613.7	316.3	0.025513746	0.26569	0.26595	0.39499	0.19733
0	38	32173.9	310.8	0.000001261	0.00001	0.00107	0.00296	0.00174
0	39	32174.6	310.8	0.000002688	0.00003	0.00305	0.00294	0.00309
0	40	32177.1	310.8	0.000006002	0.00006	0.00586	0.00518	0.00051
0	41	32250.2	310.1	0.000007017	0.00007	0.00273	0.00714	0.00364
0	42	32250.9	310.1	0.000001703	0.00002	0.00207	0.00177	0.00316
0	43	32545.5	307.3	0.042603336	0.43095	0.06745	0.07518	0.64865
0	44	32596.7	306.8	0.000973806	0.00984	0.03140	0.07559	0.05599
0	45	32652.9	306.3	0.001553869	0.01567	0.02604	0.11900	0.02879
0	46	32999.9	303.0	0.000618536	0.00617	0.04747	0.02320	0.05813
0	47	33128.9	301.9	0.000978812	0.00973	0.00380	0.07721	0.06124
0	48	33343.3	299.9	0.000579215	0.00572	0.01172	0.05227	0.05338
0	49	33380.5	299.6	0.000886651	0.00874	0.07442	0.02016	0.05292
0	50	33501.3	298.5	0.002616875	0.02572	0.03195	0.13484	0.08070

0	51	33692.0	296.8	0.000009187	0.00009	0.00205	0.00293	0.00878
0	52	33695.7	296.8	0.000032041	0.00031	0.00936	0.01444	0.00411
0	53	33699.3	296.7	0.000072840	0.00071	0.00103	0.02379	0.01202
0	54	33982.4	294.3	0.000055846	0.00054	0.00421	0.01233	0.01927
0	55	34504.1	289.8	0.002526694	0.02411	0.06593	0.13831	0.02515
0	56	34552.3	289.4	0.001182100	0.01126	0.02738	0.09284	0.04352
0	57	34868.6	286.8	0.004381341	0.04137	0.06701	0.14398	0.12707
0	58	34959.4	286.0	0.000305670	0.00288	0.00551	0.04833	0.02264
0	59	35022.6	285.5	0.000658457	0.00619	0.04480	0.04081	0.05016
0	60	35028.5	285.5	0.000159276	0.00150	0.01151	0.00508	0.03659
0	61	35062.2	285.2	0.000003918	0.00004	0.00164	0.00538	0.00227
0	62	35154.5	284.5	0.004876335	0.04567	0.11423	0.10857	0.14432
0	63	35224.4	283.9	0.001055106	0.00986	0.01083	0.07341	0.06599
0	64	35393.1	282.5	0.000000534	0.00000	0.00054	0.00164	0.00141
0	65	35408.3	282.4	0.000591821	0.00550	0.03796	0.03759	0.05146
0	66	35413.0	282.4	0.000230510	0.00214	0.00627	0.03528	0.02931
0	67	35686.5	280.2	0.001094835	0.01010	0.06352	0.03465	0.06974
0	68	35815.4	279.2	0.000005817	0.00005	0.00307	0.00599	0.00285
0	69	35815.8	279.2	0.000002589	0.00002	0.00013	0.00183	0.00452
0	70	35816.6	279.2	0.000012652	0.00012	0.00928	0.00034	0.00548
0	71	35858.4	278.9	0.000007624	0.00007	0.00583	0.00571	0.00185
0	72	35860.3	278.9	0.000022250	0.00020	0.00994	0.00262	0.00992
0	73	35861.2	278.9	0.000067681	0.00062	0.01777	0.01725	0.00283
0	74	36958.2	270.6	0.002064833	0.01839	0.01711	0.01443	0.13376
0	75	37228.6	268.6	0.055412912	0.49002	0.53635	0.44935	0.02090
0	76	38024.2	263.0	0.029330897	0.25395	0.46609	0.17826	0.07024
0	77	38119.7	262.3	0.016695978	0.14419	0.05118	0.12596	0.35455
0	78	38787.9	257.8	0.056140135	0.47649	0.03307	0.37454	0.57889
0	79	38944.5	256.8	0.008336373	0.07047	0.00311	0.19326	0.18197
0	80	39093.0	255.8	0.073790615	0.62141	0.13707	0.60165	0.49055

2b [Cu(^{Et}cAAC)(PMes$_2$)]

Table S13: calculated absorption data for 2b

ABSORPTION SPECTRUM VIA TRANSITION ELECTRIC DIPOLE MOMENTS

State	Energy (cm-1)	Wavelength (nm)	fosc	T2 (au**2)	TX (au)	TY (au)	TZ (au)
1	21741.3	460.0	0.012167347	0.18424	-0.14190	-0.38596	0.12303
2	27625.7	362.0	0.034513995	0.41130	0.41731	0.24362	0.42166
3	28663.9	348.9	0.054339687	0.62411	0.59785	-0.40112	-0.32525
4	29714.2	336.5	0.002516758	0.02788	-0.15970	-0.00409	0.04860
5	30175.7	331.4	0.297577103	3.24652	1.05408	-1.09101	-0.97217
6	31970.8	312.8	0.075755307	0.78007	-0.33043	-0.28697	-0.76716
7	32284.4	309.7	0.022761036	0.23210	-0.33297	-0.28273	0.20321
8	33687.5	296.8	0.037694965	0.36838	0.14418	0.35822	0.46826
9	34665.0	288.5	0.130200693	1.23651	-0.36770	-0.62970	-0.83952
10	34840.6	287.0	0.010324715	0.09756	-0.10317	-0.00013	-0.29481
11	34922.6	286.3	0.004698630	0.04429	-0.20543	-0.00870	0.04491
12	35730.2	279.9	0.037910943	0.34930	-0.48668	-0.30764	-0.13342
13	36245.1	275.9	0.004927956	0.04476	-0.13614	0.16190	-0.00392
14	36386.6	274.8	0.066227512	0.59920	0.71474	-0.26084	-0.14250
15	37416.7	267.3	0.009259859	0.08147	-0.14343	-0.16149	-0.18661
16	37873.8	264.0	0.005168117	0.04492	-0.19877	0.00381	-0.07349
17	38035.2	262.9	0.109931256	0.95150	0.97492	-0.03232	-0.00095
18	38721.5	258.3	0.044192047	0.37572	0.17078	-0.58770	-0.03407
19	39424.8	253.6	0.015250498	0.12735	-0.15950	0.27612	0.16020
20	40390.6	247.6	0.006213081	0.05064	0.20053	-0.10016	-0.01990
21	20605.3	485.3	spin forbidden (mult=3)				
22	24527.2	407.7	spin forbidden (mult=3)				
23	25071.4	398.9	spin forbidden (mult=3)				
24	27241.7	367.1	spin forbidden (mult=3)				
25	28865.2	346.4	spin forbidden (mult=3)				
26	29420.5	339.9	spin forbidden (mult=3)				
27	29612.5	337.7	spin forbidden (mult=3)				
28	29743.3	336.2	spin forbidden (mult=3)				
29	30291.3	330.1	spin forbidden (mult=3)				
30	31400.7	318.5	spin forbidden (mult=3)				
31	32666.9	306.1	spin forbidden (mult=3)				
32	33099.5	302.1	spin forbidden (mult=3)				
33	33540.5	298.1	spin forbidden (mult=3)				
34	33835.6	295.5	spin forbidden (mult=3)				
35	34112.4	293.1	spin forbidden (mult=3)				
36	35070.4	285.1	spin forbidden (mult=3)				
37	35444.4	282.1	spin forbidden (mult=3)				
38	35670.7	280.3	spin forbidden (mult=3)				
39	35922.7	278.4	spin forbidden (mult=3)				
40	36210.7	276.2	spin forbidden (mult=3)				

Table S14: calculated SOC-corrected absorption data for 2b

SPIN ORBIT CORRECTED ABSORPTION SPECTRUM VIA TRANSITION ELECTRIC DIPOLE MOMENTS

States		Energy (cm-1)	Wavelength (nm)	fosc	T2 (au**2)	TX (au)	TY (au)	TZ (au)
0	1	20604.2	485.3	0.000000805	0.00001	0.00311	0.00135	0.00118
0	2	20604.3	485.3	0.000000145	0.00000	0.00020	0.00014	0.00150
0	3	20605.0	485.3	0.000001051	0.00002	0.00216	0.00219	0.00271
0	4	21739.4	460.0	0.012151965	0.18402	0.14176	0.38558	0.12352
0	5	24492.6	408.3	0.000000241	0.00000	0.00087	0.00157	0.00012
0	6	24494.2	408.3	0.000006238	0.00008	0.00251	0.00878	0.00064
0	7	24504.2	408.1	0.000112658	0.00151	0.03025	0.02289	0.00862
0	8	25074.2	398.8	0.000000832	0.00001	0.00202	0.00013	0.00261
0	9	25074.2	398.8	0.000000216	0.00000	0.00021	0.00133	0.00102
0	10	25074.2	398.8	0.000000165	0.00000	0.00114	0.00091	0.00020
0	11	27244.4	367.0	0.000015075	0.00018	0.00683	0.00704	0.00926
0	12	27244.5	367.0	0.000000769	0.00001	0.00045	0.00209	0.00217
0	13	27244.5	367.0	0.000000534	0.00001	0.00136	0.00123	0.00176
0	14	27559.2	362.9	0.033668207	0.40219	0.41289	0.24026	0.41712
0	15	28666.4	348.8	0.054334000	0.62398	0.59731	0.40143	0.32568
0	16	28867.8	346.4	0.000000916	0.00001	0.00226	0.00191	0.00130
0	17	28867.8	346.4	0.000000847	0.00001	0.00186	0.00190	0.00161
0	18	28867.8	346.4	0.000001871	0.00002	0.00385	0.00190	0.00172
0	19	29389.5	340.3	0.000003117	0.00003	0.00461	0.00358	0.00092
0	20	29391.0	340.2	0.000090383	0.00101	0.02465	0.01045	0.01720
0	21	29398.4	340.2	0.000266076	0.00298	0.03881	0.02437	0.02965
0	22	29611.1	337.7	0.000055508	0.00062	0.01922	0.01258	0.00947
0	23	29611.3	337.7	0.000011380	0.00013	0.00923	0.00519	0.00380
0	24	29611.8	337.7	0.000022144	0.00025	0.00690	0.00981	0.01012
0	25	29717.1	336.5	0.002540857	0.02815	0.16057	0.00443	0.04844
0	26	29745.9	336.2	0.000000773	0.00001	0.00210	0.00118	0.00166
0	27	29746.1	336.2	0.000005817	0.00006	0.00108	0.00643	0.00468
0	28	29746.2	336.2	0.000005368	0.00006	0.00179	0.00490	0.00567
0	29	30178.3	331.4	0.297215388	3.24229	1.05368	1.09024	0.97131
0	30	30293.8	330.1	0.000020210	0.00022	0.01131	0.00638	0.00715
0	31	30293.9	330.1	0.000052789	0.00057	0.01445	0.01382	0.01318
0	32	30294.0	330.1	0.000042128	0.00046	0.00833	0.01397	0.01390
0	33	31403.0	318.4	0.000009352	0.00010	0.00801	0.00540	0.00218
0	34	31403.2	318.4	0.000000046	0.00000	0.00034	0.00024	0.00056
0	35	31403.3	318.4	0.000000476	0.00000	0.00200	0.00100	0.00006
0	36	31950.9	313.0	0.070508116	0.72649	0.39612	0.33418	0.67669
0	37	32102.9	311.5	0.021702905	0.22256	0.18160	0.13340	0.41447
0	38	32394.3	308.7	0.000024391	0.00025	0.01210	0.00812	0.00597
0	39	32438.6	308.3	0.001400153	0.01421	0.09609	0.05331	0.04620
0	40	32579.6	306.9	0.002524435	0.02551	0.07938	0.13369	0.03654
0	41	33096.5	302.1	0.000045473	0.00045	0.01707	0.01042	0.00724
0	42	33099.8	302.1	0.000008536	0.00008	0.00881	0.00270	0.00011
0	43	33102.0	302.1	0.000004619	0.00005	0.00190	0.00527	0.00381
0	44	33187.4	301.3	0.001869111	0.01854	0.08652	0.09201	0.05089
0	45	33345.1	299.9	0.000515593	0.00509	0.04498	0.04745	0.02857
0	46	33513.6	298.4	0.001650287	0.01621	0.09307	0.07836	0.03754
0	47	33645.5	297.2	0.004303436	0.04211	0.00581	0.16986	0.11498
0	48	33692.3	296.8	0.034472888	0.33684	0.14568	0.32989	0.45474
0	49	34082.2	293.4	0.005508929	0.05321	0.13759	0.13830	0.12310
0	50	34103.9	293.2	0.000008085	0.00008	0.00455	0.00598	0.00465

0	51	34110.8	293.2	0.000020561	0.00020	0.00079	0.01304	0.00526
0	52	34154.7	292.8	0.010982809	0.10586	0.19589	0.19143	0.17562
0	53	34347.3	291.1	0.000238471	0.00229	0.02603	0.02749	0.02920
0	54	34696.4	288.2	0.120979584	1.14790	0.33705	0.56905	0.84290
0	55	34915.2	286.4	0.003841040	0.03622	0.16147	0.00918	0.10030
0	56	35017.0	285.6	0.001020256	0.00959	0.00658	0.06466	0.07326
0	57	35075.7	285.1	0.000025534	0.00024	0.01104	0.00394	0.01011
0	58	35080.3	285.1	0.000034561	0.00032	0.01685	0.00353	0.00529
0	59	35140.2	284.6	0.001553877	0.01456	0.02005	0.09121	0.07639
0	60	35485.4	281.8	0.004674762	0.04337	0.16588	0.12564	0.00821
0	61	35576.2	281.1	0.000250794	0.00232	0.03676	0.02983	0.00892
0	62	35665.1	280.4	0.000081893	0.00076	0.02075	0.00197	0.01793
0	63	35672.8	280.3	0.000169866	0.00157	0.03589	0.01623	0.00406
0	64	35676.6	280.3	0.000020938	0.00019	0.01124	0.00768	0.00280
0	65	35737.3	279.8	0.000271436	0.00250	0.01171	0.01141	0.04726
0	66	35925.2	278.4	0.000742132	0.00680	0.07449	0.02770	0.02203
0	67	35926.3	278.3	0.000006862	0.00006	0.00704	0.00035	0.00364
0	68	35927.7	278.3	0.000088122	0.00081	0.02397	0.00950	0.01195
0	69	35999.9	277.8	0.029776766	0.27230	0.44229	0.24211	0.13440
0	70	36214.6	276.1	0.000168184	0.00153	0.02074	0.03310	0.00183
0	71	36218.3	276.1	0.000031076	0.00028	0.00889	0.01267	0.00656
0	72	36231.1	276.0	0.002623303	0.02384	0.14391	0.04199	0.03691
0	73	36268.8	275.7	0.010458895	0.09494	0.25844	0.16767	0.00579
0	74	36407.7	274.7	0.057522338	0.52014	0.65650	0.25755	0.15102
0	75	37422.7	267.2	0.009063035	0.07973	0.13619	0.16031	0.18836
0	76	37880.7	264.0	0.004769990	0.04145	0.18892	0.00510	0.07575
0	77	38056.9	262.8	0.111399048	0.96366	0.98056	0.04651	0.00108
0	78	38745.0	258.1	0.043358323	0.36841	0.14632	0.58786	0.03767
0	79	39427.8	253.6	0.015214705	0.12704	0.16025	0.27526	0.15997
0	80	40396.1	247.5	0.006236807	0.05083	0.20149	0.09921	0.01969

2c [Cu(^{Cy}cAAC)($PMes_2$)]

Table S15: calculated absorption data for 2c

ABSORPTION SPECTRUM VIA TRANSITION ELECTRIC DIPOLE MOMENTS

State	Energy (cm-1)	Wavelength (nm)	fosc	T2 (au**2)	TX (au)	TY (au)	TZ (au)
1	20766.2	481.6	0.066793209	1.05889	0.01949	-0.42112	-0.93871
2	27715.0	360.8	0.001837521	0.02183	-0.06122	0.09974	0.09018
3	28174.1	354.9	0.046994761	0.54913	-0.49672	-0.32719	-0.44199
4	28605.8	349.6	0.065650125	0.75554	-0.45644	0.65429	-0.34512
5	29195.4	342.5	0.188206438	2.12225	0.85277	-0.78157	0.88554
6	31338.8	319.1	0.161276165	1.69419	0.64736	0.69252	0.89193
7	31854.7	313.9	0.044625214	0.46119	-0.11552	0.54694	0.38562
8	32508.4	307.6	0.039308093	0.39807	-0.07931	-0.07824	-0.62102
9	32900.2	303.9	0.000611991	0.00612	0.04333	-0.04738	0.04474
10	34341.0	291.2	0.005318177	0.05098	0.09063	-0.20121	0.04779
11	34732.5	287.9	0.007323890	0.06942	-0.13744	-0.21896	-0.05086
12	34922.2	286.4	0.008255506	0.07782	-0.10751	-0.13296	-0.22043
13	35875.9	278.7	0.000966735	0.00887	-0.04714	0.04083	0.07058
14	37144.5	269.2	0.004564630	0.04046	-0.09111	-0.11645	0.13636
15	37300.8	268.1	0.055259895	0.48772	-0.54938	-0.42767	0.05477
16	38068.1	262.7	0.030188653	0.26107	-0.46668	0.18308	-0.09882
17	38242.3	261.5	0.026439757	0.22761	-0.12454	0.25130	0.38593
18	38673.5	258.6	0.064299361	0.54735	0.02555	0.45018	0.58655
19	38965.0	256.6	0.000234755	0.00198	0.03007	0.02988	0.01363
20	39219.4	255.0	0.067899639	0.56996	0.10042	-0.60777	-0.43645
21	19010.2	526.0	spin forbidden (mult=3)				
22	24948.3	400.8	spin forbidden (mult=3)				
23	25028.2	399.5	spin forbidden (mult=3)				
24	26663.8	375.0	spin forbidden (mult=3)				
25	27785.7	359.9	spin forbidden (mult=3)				
26	28665.0	348.9	spin forbidden (mult=3)				
27	28740.0	347.9	spin forbidden (mult=3)				
28	29799.5	335.6	spin forbidden (mult=3)				
29	30188.6	331.3	spin forbidden (mult=3)				
30	31344.8	319.0	spin forbidden (mult=3)				
31	32177.0	310.8	spin forbidden (mult=3)				
32	33105.7	302.1	spin forbidden (mult=3)				
33	33136.1	301.8	spin forbidden (mult=3)				
34	33642.8	297.2	spin forbidden (mult=3)				
35	33683.0	296.9	spin forbidden (mult=3)				
36	34914.7	286.4	spin forbidden (mult=3)				
37	35086.6	285.0	spin forbidden (mult=3)				
38	35530.7	281.4	spin forbidden (mult=3)				
39	35811.5	279.2	spin forbidden (mult=3)				
40	35861.6	278.8	spin forbidden (mult=3)				

Table S16: calculated SOC-corrected absorption data for 2c

SPIN ORBIT CORRECTED ABSORPTION SPECTRUM VIA TRANSITION ELECTRIC DIPOLE MOMENTS

States		Energy (cm-1)	Wavelength (nm)	fosc	T2 (au**2)	TX (au)	TY (au)	TZ (au)
0	1	19009.9	526.0	0.000000371	0.00001	0.00045	0.00134	0.00210
0	2	19010.0	526.0	0.000000204	0.00000	0.00134	0.00019	0.00131
0	3	19010.5	526.0	0.000002956	0.00005	0.00467	0.00344	0.00419
0	4	20766.2	481.6	0.066730896	1.05790	0.01950	0.42089	0.93829
0	5	24948.3	400.8	0.000000656	0.00001	0.00046	0.00265	0.00120
0	6	24948.3	400.8	0.000000825	0.00001	0.00200	0.00258	0.00045
0	7	24951.1	400.8	0.000000875	0.00001	0.00240	0.00204	0.00128
0	8	24988.4	400.2	0.000000071	0.00000	0.00027	0.00044	0.00082
0	9	24990.0	400.2	0.000000851	0.00001	0.00100	0.00035	0.00318
0	10	25002.3	400.0	0.000170790	0.00225	0.00169	0.03985	0.02564
0	11	26666.6	375.0	0.000000812	0.00001	0.00239	0.00204	0.00040
0	12	26666.7	375.0	0.000000555	0.00001	0.00165	0.00126	0.00160
0	13	26666.8	375.0	0.000000098	0.00000	0.00080	0.00049	0.00056
0	14	27717.8	360.8	0.001775935	0.02109	0.06378	0.09647	0.08786
0	15	27788.4	359.9	0.000007506	0.00009	0.00338	0.00795	0.00377
0	16	27788.5	359.9	0.000002123	0.00003	0.00364	0.00164	0.00303
0	17	27788.5	359.9	0.000000420	0.00000	0.00150	0.00085	0.00141
0	18	28046.4	356.6	0.038060497	0.44676	0.41512	0.36934	0.37152
0	19	28574.0	350.0	0.066604063	0.76737	0.53335	0.55107	0.42336
0	20	28666.8	348.8	0.000000118	0.00000	0.00082	0.00002	0.00082
0	21	28666.9	348.8	0.000034895	0.00040	0.01103	0.01420	0.00880
0	22	28667.9	348.8	0.000019104	0.00022	0.00463	0.01395	0.00185
0	23	28726.6	348.1	0.000002149	0.00002	0.00473	0.00136	0.00063
0	24	28728.3	348.1	0.000008900	0.00010	0.00354	0.00490	0.00809
0	25	28817.2	347.0	0.007748490	0.08852	0.01698	0.29167	0.05623
0	26	29198.9	342.5	0.187933741	2.11892	0.85067	0.78535	0.88233
0	27	29802.4	335.5	0.000000151	0.00000	0.00075	0.00013	0.00105
0	28	29802.4	335.5	0.000000254	0.00000	0.00070	0.00054	0.00142
0	29	29802.4	335.5	0.000000748	0.00001	0.00121	0.00260	0.00013
0	30	30191.4	331.2	0.000001353	0.00001	0.00144	0.00259	0.00244
0	31	30191.4	331.2	0.000004303	0.00005	0.00333	0.00543	0.00251
0	32	30191.5	331.2	0.000000524	0.00001	0.00195	0.00031	0.00135
0	33	31338.9	319.1	0.161958425	1.70136	0.64219	0.69947	0.89425
0	34	31347.5	319.0	0.000015265	0.00016	0.00656	0.00609	0.00895
0	35	31347.6	319.0	0.000074509	0.00078	0.01519	0.01461	0.01839
0	36	31347.6	319.0	0.001386791	0.01456	0.06088	0.06328	0.08279
0	37	31808.4	314.4	0.040210563	0.41617	0.13135	0.52263	0.35465
0	38	32175.9	310.8	0.000000661	0.00001	0.00081	0.00208	0.00134
0	39	32176.5	310.8	0.000001959	0.00002	0.00296	0.00103	0.00320
0	40	32177.6	310.8	0.000002550	0.00003	0.00464	0.00067	0.00203
0	41	32432.8	308.3	0.000013990	0.00014	0.00283	0.00926	0.00694
0	42	32433.1	308.3	0.000007882	0.00008	0.00102	0.00433	0.00776
0	43	32511.1	307.6	0.039794003	0.40296	0.08047	0.08219	0.62428
0	44	32784.6	305.0	0.000818974	0.00822	0.03915	0.07944	0.01952
0	45	32822.2	304.7	0.001612447	0.01617	0.03846	0.11062	0.04956
0	46	32903.9	303.9	0.000589888	0.00590	0.03790	0.05281	0.04095
0	47	33308.1	300.2	0.001022659	0.01011	0.00147	0.08087	0.05972
0	48	33516.9	298.4	0.000577461	0.00567	0.00661	0.05079	0.05522
0	49	33547.7	298.1	0.000853679	0.00838	0.07534	0.02038	0.04781
0	50	33638.4	297.3	0.000914434	0.00895	0.02439	0.07920	0.04562

0	51	33663.6	297.1	0.000032572	0.00032	0.00699	0.00074	0.01640
0	52	33671.9	297.0	0.000156943	0.00153	0.02751	0.02574	0.01073
0	53	33699.6	296.7	0.001615879	0.01579	0.02051	0.10973	0.05766
0	54	34157.6	292.8	0.000053485	0.00052	0.00456	0.01248	0.01841
0	55	34661.8	288.5	0.002523771	0.02397	0.06422	0.13937	0.02059
0	56	34696.4	288.2	0.001065981	0.01011	0.02745	0.08802	0.04016
0	57	34957.0	286.1	0.000744711	0.00701	0.02843	0.06054	0.05040
0	58	34994.3	285.8	0.000260348	0.00245	0.01741	0.04632	0.00071
0	59	35020.8	285.5	0.000284755	0.00268	0.01874	0.04821	0.00120
0	60	35069.5	285.1	0.003624217	0.03402	0.06703	0.12930	0.11318
0	61	35170.9	284.3	0.000054641	0.00051	0.00055	0.02075	0.00897
0	62	35311.2	283.2	0.004633111	0.04320	0.11993	0.11019	0.12911
0	63	35384.6	282.6	0.001095094	0.01019	0.00420	0.06848	0.07404
0	64	35539.7	281.4	0.000000421	0.00000	0.00132	0.00146	0.00015
0	65	35550.7	281.3	0.000369148	0.00342	0.03188	0.03912	0.02952
0	66	35552.2	281.3	0.000179610	0.00166	0.00759	0.01513	0.03710
0	67	35817.1	279.2	0.000000517	0.00000	0.00167	0.00030	0.00137
0	68	35817.8	279.2	0.000070368	0.00065	0.00737	0.01383	0.02003
0	69	35818.5	279.2	0.000022689	0.00021	0.00486	0.01334	0.00264
0	70	35867.1	278.8	0.000292707	0.00269	0.01184	0.02981	0.04072
0	71	35872.5	278.8	0.000050253	0.00046	0.00131	0.01003	0.01894
0	72	35876.7	278.7	0.000056617	0.00052	0.00427	0.02080	0.00828
0	73	35893.5	278.6	0.000612750	0.00562	0.05676	0.02322	0.04311
0	74	37156.4	269.1	0.004609685	0.04084	0.09565	0.11473	0.13613
0	75	37309.0	268.0	0.055230893	0.48735	0.55247	0.42255	0.05984
0	76	38073.3	262.7	0.029561694	0.25561	0.46100	0.17544	0.11096
0	77	38255.8	261.4	0.025510959	0.21954	0.12913	0.24945	0.37502
0	78	38677.1	258.6	0.064212714	0.54657	0.02581	0.44952	0.58637
0	79	38969.0	256.6	0.000306065	0.00259	0.02893	0.03721	0.01908
0	80	39242.5	254.8	0.069046786	0.57924	0.10169	0.61320	0.43920

2d [Cu(^{Ment}cAAC)(PMes$_2$)]

Table S17: calculated absorption data for 2d

ABSORPTION SPECTRUM VIA TRANSITION ELECTRIC DIPOLE MOMENTS

State	Energy (cm-1)	Wavelength (nm)	fosc	T2 (au**2)	TX (au)	TY (au)	TZ (au)
1	21278.3	470.0	0.043567139	0.67406	0.04750	0.30825	-0.75946
2	27420.5	364.7	0.071581775	0.85942	0.64541	0.64151	0.17698
3	27679.8	361.3	0.030028647	0.35715	0.32883	-0.45601	0.20266
4	28928.2	345.7	0.016681189	0.18984	-0.29512	-0.32030	0.01222
5	29223.6	342.2	0.148471824	1.67257	1.00666	0.63713	0.50328
6	31729.1	315.2	0.087330901	0.90612	-0.66982	0.45000	-0.50493
7	31977.8	312.7	0.024673766	0.25402	-0.19328	-0.24992	0.39268
8	33179.1	301.4	0.061301196	0.60825	0.52174	-0.31204	0.48853
9	34291.4	291.6	0.179005566	1.71853	-0.94596	0.36191	-0.83229
10	34954.9	286.1	0.003335314	0.03141	-0.00155	0.09867	0.14723
11	35345.4	282.9	0.002255866	0.02101	-0.07546	-0.08887	0.08614
12	35623.7	280.7	0.018138028	0.16762	-0.04225	0.31110	-0.26278
13	35867.2	278.8	0.000469859	0.00431	-0.00776	-0.05983	0.02594
14	36987.8	270.4	0.078096361	0.69510	-0.52535	-0.61443	-0.20393
15	37528.7	266.5	0.007939901	0.06965	0.09871	0.19758	-0.14446
16	38314.6	261.0	0.032700608	0.28097	0.04383	0.44973	-0.27713
17	38428.1	260.2	0.055991447	0.47968	0.32694	0.55616	-0.25193
18	39213.1	255.0	0.013416501	0.11264	0.31520	0.10418	0.04930
19	39648.9	252.2	0.079479382	0.65993	-0.10768	-0.77985	0.20044
20	39732.5	251.7	0.023403349	0.19391	0.42315	0.08598	0.08642
21	19795.7	505.2	spin forbidden (mult=3)				
22	24378.5	410.2	spin forbidden (mult=3)				
23	24781.2	403.5	spin forbidden (mult=3)				
24	26904.9	371.7	spin forbidden (mult=3)				
25	28088.1	356.0	spin forbidden (mult=3)				
26	28947.1	345.5	spin forbidden (mult=3)				
27	29390.7	340.2	spin forbidden (mult=3)				
28	29649.0	337.3	spin forbidden (mult=3)				
29	30378.3	329.2	spin forbidden (mult=3)				
30	31299.8	319.5	spin forbidden (mult=3)				
31	31946.9	313.0	spin forbidden (mult=3)				
32	33448.3	299.0	spin forbidden (mult=3)				
33	33709.7	296.7	spin forbidden (mult=3)				
34	34002.0	294.1	spin forbidden (mult=3)				
35	34108.3	293.2	spin forbidden (mult=3)				
36	35272.7	283.5	spin forbidden (mult=3)				
37	35299.5	283.3	spin forbidden (mult=3)				
38	35577.6	281.1	spin forbidden (mult=3)				
39	35828.6	279.1	spin forbidden (mult=3)				
40	35883.2	278.7	spin forbidden (mult=3)				

Table S18: calculated CD-spectral data for 2d

CD SPECTRUM

State	Energy (cm-1)	Wavelength (nm)	R (1e40*cgs)	MX (au)	MY (au)	MZ (au)
1	21278.3	470.0	6.75751	-0.05096	-0.06769	-0.04954
2	27420.5	364.7	4.39498	-0.14756	0.13101	0.11590
3	27679.8	361.3	-21.14750	0.17157	0.22090	-0.00267
4	28928.2	345.7	0.73585	0.05976	-0.06248	-0.06691
5	29223.6	342.2	65.01414	-0.11539	0.25635	0.18028
6	31729.1	315.2	-1.29692	0.00952	-0.16079	-0.15049
7	31977.8	312.7	17.33696	0.00437	-0.06732	0.05295
8	33179.1	301.4	-34.28905	0.02644	0.10239	-0.11171

9	34291.4	291.6	-118.59008	0.10554	-0.41567	0.00153
10	34954.9	286.1	2.19121	0.02632	0.08763	-0.02688
11	35345.4	282.9	-5.27329	-0.04905	0.11899	-0.05006
12	35623.7	280.7	-2.34602	-0.01326	0.04026	0.06873
13	35867.2	278.8	2.32608	-0.00959	-0.05102	0.06966
14	36987.8	270.4	-6.74579	0.04884	-0.03966	0.06383
15	37528.7	266.5	-6.18547	-0.10494	0.00597	0.02728
16	38314.6	261.0	-15.47029	-0.04494	-0.07307	-0.00727
17	38428.1	260.2	5.91541	-0.13113	0.04345	-0.12404
18	39213.1	255.0	4.81961	0.00486	0.06557	0.03770
19	39648.9	252.2	47.81349	0.06943	-0.10758	0.12472
20	39732.5	251.7	20.68840	0.12820	-0.07387	-0.04648
21	19795.7	505.2	spin forbidden			
22	24378.5	410.2	spin forbidden			
23	24781.2	403.5	spin forbidden			
24	26904.9	371.7	spin forbidden			
25	28088.1	356.0	spin forbidden			
26	28947.1	345.5	spin forbidden			
27	29390.7	340.2	spin forbidden			
28	29649.0	337.3	spin forbidden			
29	30378.3	329.2	spin forbidden			
30	31299.8	319.5	spin forbidden			
31	31946.9	313.0	spin forbidden			
32	33448.3	299.0	spin forbidden			
33	33709.7	296.7	spin forbidden			
34	34002.0	294.1	spin forbidden			
35	34108.3	293.2	spin forbidden			
36	35272.7	283.5	spin forbidden			
37	35299.5	283.3	spin forbidden			
38	35577.6	281.1	spin forbidden			
39	35828.6	279.1	spin forbidden			
40	35883.2	278.7	spin forbidden			

Calculated CD properties

Table S19: calculated absorpton dissymmetry for 2d

States	θ / °	cos(θ)	|$\boldsymbol{\mu}$|	|$\boldsymbol{m}$|	g_{abs} (calcd).
1	79.75	0.177931	0.821007798	0.098148201	0.0006
2	87.48	0.043929	0.927044265	0.228845764	0.0003
3	105.6	0.268340	0.597616403	0.279714504	-0.0036
4	88.13	0.032702	0.435703148	0.109323859	0.00024
5	71.38	0.319283	1.293286415	0.333962652	0.0023

Table S20: calculated SOC-corrected absorption data for 2d

SPIN ORBIT CORRECTED ABSORPTION SPECTRUM VIA TRANSITION ELECTRIC DIPOLE MOMENTS

States		Energy (cm-1)	Wavelength (nm)	fosc	T2 (au**2)	TX (au)	TY (au)	TZ (au)
0	1	19795.5	505.2	0.000000438	0.00001	0.00227	0.00146	0.00008
0	2	19795.5	505.2	0.000000077	0.00000	0.00050	0.00080	0.00062
0	3	19796.1	505.2	0.000003075	0.00005	0.00232	0.00171	0.00654
0	4	21278.1	470.0	0.043534771	0.67356	0.04810	0.30837	0.75905
0	5	24344.8	410.8	0.000000297	0.00000	0.00092	0.00156	0.00086
0	6	24346.0	410.7	0.000002692	0.00004	0.00051	0.00004	0.00601
0	7	24356.0	410.6	0.000118031	0.00160	0.00052	0.03257	0.02311
0	8	24783.8	403.5	0.000001157	0.00002	0.00319	0.00199	0.00112
0	9	24783.8	403.5	0.000000490	0.00001	0.00059	0.00233	0.00085
0	10	24783.9	403.5	0.000000371	0.00000	0.00151	0.00149	0.00066
0	11	26907.4	371.6	0.000008303	0.00010	0.00198	0.00925	0.00349
0	12	26907.5	371.6	0.000003237	0.00004	0.00495	0.00309	0.00236
0	13	26907.6	371.6	0.000000213	0.00000	0.00031	0.00145	0.00063
0	14	27422.9	364.7	0.071620171	0.85980	0.64292	0.64472	0.17547
0	15	27604.1	362.3	0.029236344	0.34868	0.33088	0.44520	0.20246
0	16	28090.6	356.0	0.000000258	0.00000	0.00008	0.00144	0.00097

0	17	28090.6	356.0	0.000000256	0.00000	0.00058	0.00026	0.00161
0	18	28090.7	356.0	0.000010768	0.00013	0.00408	0.01007	0.00285
0	19	28930.5	345.7	0.016553984	0.18837	0.29546	0.31772	0.01136
0	20	28949.4	345.4	0.000005257	0.00006	0.00416	0.00618	0.00207
0	21	28949.5	345.4	0.000016113	0.00018	0.00791	0.01057	0.00300
0	22	28949.6	345.4	0.000025660	0.00029	0.00867	0.01393	0.00476
0	23	29224.8	342.2	0.147006605	1.65600	0.99861	0.64121	0.49764
0	24	29369.1	340.5	0.000164172	0.00184	0.03429	0.02048	0.01566
0	25	29370.9	340.5	0.000009918	0.00011	0.00756	0.00158	0.00717
0	26	29388.5	340.3	0.001836169	0.02057	0.12005	0.00890	0.07796
0	27	29651.3	337.3	0.000005856	0.00007	0.00645	0.00367	0.00315
0	28	29651.3	337.3	0.000003254	0.00004	0.00482	0.00152	0.00326
0	29	29651.4	337.3	0.000012617	0.00014	0.00953	0.00151	0.00685
0	30	30380.9	329.2	0.000000236	0.00000	0.00152	0.00043	0.00022
0	31	30380.9	329.2	0.000000067	0.00000	0.00011	0.00053	0.00066
0	32	30380.9	329.2	0.000003140	0.00003	0.00399	0.00414	0.00098
0	33	31302.2	319.5	0.000005548	0.00006	0.00132	0.00575	0.00485
0	34	31302.2	319.5	0.000000299	0.00000	0.00063	0.00153	0.00064
0	35	31302.3	319.5	0.000000599	0.00001	0.00069	0.00199	0.00136
0	36	31724.2	315.2	0.088714537	0.92062	0.65232	0.46624	0.52699
0	37	31934.9	313.1	0.012283249	0.12663	0.19749	0.15407	0.25275
0	38	31948.4	313.0	0.000005115	0.00005	0.00610	0.00393	0.00031
0	39	31948.9	313.0	0.000005083	0.00005	0.00384	0.00609	0.00075
0	40	31959.0	312.9	0.009948677	0.10248	0.14702	0.15368	0.23927
0	41	32950.0	303.5	0.000010111	0.00010	0.00557	0.00393	0.00739
0	42	32951.6	303.5	0.000008226	0.00008	0.00209	0.00265	0.00841
0	43	33180.7	301.4	0.060362569	0.59890	0.51740	0.30337	0.48904
0	44	33216.6	301.1	0.000498594	0.00494	0.04001	0.05733	0.00734
0	45	33383.9	299.5	0.003767321	0.03715	0.10818	0.10166	0.12294
0	46	33643.0	297.2	0.000170811	0.00167	0.00157	0.00378	0.04068
0	47	33779.6	296.0	0.004226732	0.04119	0.14405	0.06382	0.12795
0	48	33913.5	294.9	0.000671114	0.00651	0.04285	0.06683	0.01456
0	49	34002.3	294.1	0.000121052	0.00117	0.01843	0.02076	0.02003
0	50	34003.3	294.1	0.000002516	0.00002	0.00196	0.00451	0.00040
0	51	34003.9	294.1	0.000060991	0.00059	0.01862	0.01035	0.01168
0	52	34222.6	292.2	0.086778360	0.83478	0.60136	0.31779	0.61005
0	53	34361.9	291.0	0.087229142	0.83572	0.70620	0.18168	0.55136
0	54	34607.8	289.0	0.000008198	0.00008	0.00467	0.00409	0.00628
0	55	35034.5	285.4	0.001451798	0.01364	0.05620	0.09876	0.02700
0	56	35198.8	284.1	0.001205941	0.01128	0.03645	0.01770	0.09817
0	57	35294.9	283.3	0.000036610	0.00034	0.00899	0.00966	0.01294
0	58	35295.9	283.3	0.000019072	0.00018	0.00086	0.01301	0.00278
0	59	35304.6	283.2	0.000116387	0.00109	0.00058	0.00349	0.03275
0	60	35444.4	282.1	0.000882986	0.00820	0.02037	0.04300	0.07705
0	61	35466.1	282.0	0.002964838	0.02752	0.01478	0.07543	0.14701
0	62	35573.5	281.1	0.000194246	0.00180	0.00281	0.02475	0.03431
0	63	35587.1	281.0	0.000141930	0.00131	0.00419	0.03519	0.00757
0	64	35592.5	281.0	0.000066315	0.00061	0.01161	0.01178	0.01843
0	65	35761.1	279.6	0.000185717	0.00171	0.02021	0.03232	0.01602
0	66	35823.6	279.1	0.002557783	0.02351	0.01497	0.12085	0.09315
0	67	35830.6	279.1	0.000064588	0.00059	0.00631	0.01782	0.01536
0	68	35838.9	279.0	0.000491333	0.00451	0.01839	0.04557	0.04581
0	69	35866.9	278.8	0.001972721	0.01811	0.01447	0.10278	0.08564
0	70	35886.7	278.7	0.000030699	0.00028	0.01157	0.01210	0.00109
0	71	35935.6	278.3	0.001322687	0.01212	0.01562	0.06645	0.08636
0	72	35967.7	278.0	0.001101039	0.01008	0.00400	0.09009	0.04411
0	73	36040.8	277.5	0.006963682	0.06361	0.06082	0.20341	0.13614
0	74	36995.5	270.3	0.079212325	0.70489	0.52834	0.62074	0.20107
0	75	37539.7	266.4	0.006809587	0.05972	0.09092	0.17825	0.14028
0	76	38329.0	260.9	0.022307822	0.19160	0.00281	0.36565	0.24062
0	77	38452.6	260.1	0.063993162	0.54788	0.32576	0.60052	0.28484
0	78	39216.9	255.0	0.013414670	0.11261	0.31548	0.10273	0.05032
0	79	39662.3	252.1	0.080471903	0.66795	0.10028	0.78452	0.20597
0	80	39737.3	251.7	0.024106435	0.19971	0.42638	0.10676	0.08075

Table S21: calculated SOC-corrected CD spectral data for 2d

SPIN ORBIT CORRECTED CD SPECTRUM

States		Energy (cm-1)	Wavelength (nm)	R (1e40*sgs)	MX (au)	MY (au)	MZ (au)
0	1	19795.5	505.2	-0.00047	0.00032	0.00008	0.00193
0	2	19795.5	505.2	-0.00023	0.00004	0.00021	0.00048
0	3	19796.1	505.2	-0.00012	0.00085	0.00093	0.00010
0	4	21278.1	470.0	6.79817	0.05088	0.06751	0.04965
0	5	24344.8	410.8	0.00011	0.00143	0.00118	0.00033
0	6	24346.0	410.7	-0.01665	0.00061	0.00541	0.00579
0	7	24356.0	410.6	0.01730	0.00280	0.00087	0.00275
0	8	24783.8	403.5	-0.00019	0.00029	0.00011	0.00028
0	9	24783.8	403.5	0.00000	0.00021	0.00005	0.00029
0	10	24783.9	403.5	-0.00005	0.00001	0.00007	0.00030
0	11	26907.4	371.6	-0.00867	0.00265	0.00260	0.00011
0	12	26907.5	371.6	-0.00006	0.00070	0.00066	0.00056
0	13	26907.6	371.6	0.00015	0.00036	0.00010	0.00055
0	14	27422.9	364.7	3.82852	0.14877	0.12941	0.11591
0	15	27604.1	362.3	-20.03020	0.16767	0.21926	0.00174
0	16	28090.6	356.0	0.00004	0.00022	0.00030	0.00051
0	17	28090.6	356.0	-0.00012	0.00060	0.00039	0.00012
0	18	28090.7	356.0	-0.01431	0.00338	0.00442	0.00012
0	19	28930.5	345.7	0.89787	0.05888	0.06314	0.06677
0	20	28949.4	345.4	0.00057	0.00086	0.00097	0.00056
0	21	28949.5	345.4	-0.00146	0.00173	0.00153	0.00184
0	22	28949.6	345.4	-0.00884	0.00282	0.00119	0.00228
0	23	29224.8	342.2	62.95922	0.11770	0.25221	0.17957
0	24	29369.1	340.5	-0.00192	0.00506	0.00757	0.00092
0	25	29370.9	340.5	-0.01368	0.00211	0.01314	0.00472
0	26	29388.5	340.3	1.47025	0.01821	0.05063	0.01775
0	27	29651.3	337.3	0.00082	0.00094	0.00132	0.00094
0	28	29651.3	337.3	0.00200	0.00039	0.00308	0.00044
0	29	29651.4	337.3	0.01265	0.00207	0.00347	0.00180
0	30	30380.9	329.2	0.00021	0.00005	0.00083	0.00005
0	31	30380.9	329.2	0.00022	0.00008	0.00013	0.00059
0	32	30380.9	329.2	0.00109	0.00052	0.00086	0.00087
0	33	31302.2	319.5	0.00106	0.00047	0.00066	0.00112
0	34	31302.2	319.5	0.00007	0.00034	0.00024	0.00045
0	35	31302.3	319.5	0.00026	0.00040	0.00005	0.00069
0	36	31724.2	315.2	1.06805	0.00901	0.15617	0.15362
0	37	31934.9	313.1	7.47389	0.00444	0.06071	0.02918
0	38	31948.4	313.0	0.00166	0.00001	0.00086	0.00032
0	39	31948.9	313.0	-0.00261	0.00096	0.00066	0.00293
0	40	31959.0	312.9	6.80355	0.00290	0.04821	0.03113
0	41	32950.0	303.5	-0.00581	0.00100	0.00442	0.00007
0	42	32951.6	303.5	0.02341	0.00272	0.00591	0.00336
0	43	33180.7	301.4	-33.66340	0.02819	0.10366	0.11153
0	44	33216.6	301.1	-0.09564	0.01036	0.00238	0.01021
0	45	33383.9	299.5	-0.22783	0.00206	0.00774	0.00066
0	46	33643.0	297.2	-0.01483	0.01195	0.00905	0.00115
0	47	33779.6	296.0	0.67445	0.00179	0.00475	0.01083
0	48	33913.5	294.9	-0.02372	0.01762	0.01169	0.00525
0	49	34002.3	294.1	-0.12080	0.00271	0.00801	0.00200
0	50	34003.3	294.1	-0.00202	0.00112	0.00052	0.00071
0	51	34003.9	294.1	-0.02943	0.00159	0.00333	0.00013
0	52	34222.6	292.2	-68.22567	0.06566	0.28384	0.02464
0	53	34361.9	291.0	-50.10460	0.08413	0.31283	0.01808
0	54	34607.8	289.0	-0.02308	0.00251	0.00468	0.00899
0	55	35034.5	285.4	1.47773	0.02482	0.01688	0.00269
0	56	35198.8	284.1	-0.42286	0.01546	0.08370	0.02997
0	57	35294.9	283.3	-0.01727	0.00327	0.00255	0.00320
0	58	35295.9	283.3	0.03123	0.00233	0.00507	0.00062
0	59	35304.6	283.2	-0.17311	0.00235	0.01415	0.00975

0	60	35444.4	282.1	-0.62056	0.00250	0.00676	0.01265
0	61	35466.1	282.0	-2.84023	0.01018	0.03371	0.02266
0	62	35573.5	281.1	-0.45907	0.00598	0.02176	0.01318
0	63	35587.1	281.0	0.25627	0.00378	0.01652	0.00288
0	64	35592.5	281.0	-0.02061	0.00131	0.00433	0.00043
0	65	35761.1	279.6	-0.83894	0.02387	0.05413	0.03196
0	66	35823.6	279.1	1.04911	0.00358	0.02539	0.00963
0	67	35830.6	279.1	-0.17505	0.00306	0.00382	0.02099
0	68	35838.9	279.0	0.33750	0.00133	0.01701	0.00183
0	69	35866.9	278.8	-2.99977	0.00430	0.01973	0.04990
0	70	35886.7	278.7	0.15360	0.00875	0.01583	0.03033
0	71	35935.6	278.3	-3.97439	0.00132	0.04078	0.06600
0	72	35967.7	278.0	0.19808	0.02930	0.00275	0.01248
0	73	36040.8	277.5	5.87331	0.01291	0.06688	0.01419
0	74	36995.5	270.3	-6.98372	0.04933	0.03883	0.06394
0	75	37539.7	266.4	-6.00413	0.10318	0.00441	0.02951
0	76	38329.0	260.9	-14.84041	0.02721	0.07921	0.01077
0	77	38452.6	260.1	4.50381	0.13568	0.03182	0.12162
0	78	39216.9	255.0	4.82366	0.00513	0.06536	0.03774
0	79	39662.3	252.1	48.79874	0.07442	0.10872	0.12466
0	80	39737.3	251.7	19.85829	0.12609	0.07139	0.04977

[Cu_2(^{Me}cAAC)$_2$(μ-$PMes_2$)$_2$]

Table S22: calculated absorption for [Cu_2(^{Me}cAAC)$_2$(μ-$PMes_2$)$_2$]

ABSORPTION SPECTRUM VIA TRANSITION ELECTRIC DIPOLE MOMENTS

State	Energy (cm-1)	Wavelength (nm)	fosc	T2 (au**2)	TX (au)	TY (au)	TZ (au)
1	21876.7	457.1	0.067503222	1.01583	-0.58294	0.25207	-0.78261
2	21943.6	455.7	0.113499880	1.70280	0.82218	-0.33716	0.95559
3	22439.4	445.6	0.008840857	0.12971	0.11051	-0.23272	0.25166
4	22888.9	436.9	0.108258839	1.55709	0.96020	0.32821	0.72621
5	25392.3	393.8	0.035906189	0.46552	0.42596	-0.17632	-0.50299
6	26851.5	372.4	0.018268623	0.22398	-0.17149	-0.43494	-0.07348
7	27125.5	368.7	0.003092999	0.03754	-0.18559	-0.01222	0.05427
8	27251.4	367.0	0.009664384	0.11675	-0.11446	0.23575	0.21925
9	27717.3	360.8	0.056175657	0.66723	-0.28700	-0.71606	-0.26855
10	27741.2	360.5	0.009914942	0.11766	-0.26169	-0.21881	-0.03615
11	18212.3	549.1	spin forbidden (mult=3)				
12	18391.2	543.7	spin forbidden (mult=3)				
13	19628.9	509.5	spin forbidden (mult=3)				
14	19937.3	501.6	spin forbidden (mult=3)				
15	23273.6	429.7	spin forbidden (mult=3)				
16	23859.7	419.1	spin forbidden (mult=3)				
17	24601.5	406.5	spin forbidden (mult=3)				
18	24868.4	402.1	spin forbidden (mult=3)				
19	25514.4	391.9	spin forbidden (mult=3)				
20	25752.9	388.3	spin forbidden (mult=3)				

Table S23: calculated SOC-corrected absorption data for [Cu_2(^{Me}cAAC)$_2$(μ-$PMes_2$)$_2$]

SPIN ORBIT CORRECTED ABSORPTION SPECTRUM VIA TRANSITION ELECTRIC DIPOLE MOMENTS

States		Energy (cm-1)	Wavelength (nm)	fosc	T2 (au**2)	TX (au)	TY (au)	TZ (au)
0	1	18201.7	549.4	0.000000148	0.00000	0.00090	0.00095	0.00097
0	2	18202.0	549.4	0.000000033	0.00000	0.00001	0.00072	0.00029
0	3	18211.3	549.1	0.000000547	0.00001	0.00026	0.00241	0.00201
0	4	18377.1	544.2	0.000000041	0.00000	0.00059	0.00062	0.00004
0	5	18377.2	544.2	0.000000682	0.00001	0.00065	0.00311	0.00145
0	6	18390.3	543.8	0.000000460	0.00001	0.00067	0.00067	0.00271
0	7	19635.8	509.3	0.000002985	0.00005	0.00668	0.00166	0.00165
0	8	19644.0	509.1	0.000042649	0.00071	0.01621	0.00642	0.02027
0	9	19644.9	509.0	0.000000198	0.00000	0.00151	0.00047	0.00090
0	10	19940.9	501.5	0.000446833	0.00738	0.04800	0.02606	0.06628
0	11	19947.2	501.3	0.000000500	0.00001	0.00127	0.00129	0.00223
0	12	19947.9	501.3	0.000000011	0.00000	0.00015	0.00031	0.00025
0	13	21876.2	457.1	0.058366263	0.87834	0.53887	0.23346	0.73038
0	14	21945.1	455.7	0.122200687	1.83321	0.84958	0.35086	0.99414
0	15	22442.5	445.6	0.008634063	0.12665	0.10430	0.23391	0.24710
0	16	22881.5	437.0	0.107956258	1.55324	0.95890	0.32544	0.72653
0	17	23278.5	429.6	0.000004314	0.00006	0.00558	0.00253	0.00485
0	18	23278.6	429.6	0.000002401	0.00003	0.00325	0.00093	0.00475
0	19	23281.5	429.5	0.000284717	0.00403	0.04824	0.02474	0.03297
0	20	23864.0	419.0	0.000001555	0.00002	0.00375	0.00102	0.00252
0	21	23864.1	419.0	0.000004283	0.00006	0.00631	0.00214	0.00382
0	22	23865.6	419.0	0.000016398	0.00023	0.01287	0.00470	0.00619
0	23	24610.1	406.3	0.000000624	0.00001	0.00085	0.00121	0.00248
0	24	24610.2	406.3	0.000000654	0.00001	0.00072	0.00286	0.00025
0	25	24614.8	406.3	0.000009290	0.00012	0.00272	0.00671	0.00848
0	26	24876.3	402.0	0.000002118	0.00003	0.00033	0.00105	0.00518
0	27	24876.5	402.0	0.000000584	0.00001	0.00037	0.00057	0.00270
0	28	24880.0	401.9	0.000232682	0.00308	0.04333	0.01956	0.02862

0	29	25395.8	393.8	0.035620991	0.46176	0.42283	0.17614	0.50195
0	30	25520.3	391.8	0.000001856	0.00002	0.00322	0.00368	0.00013
0	31	25520.4	391.8	0.000005083	0.00007	0.00537	0.00097	0.00599
0	32	25522.8	391.8	0.000305546	0.00394	0.05483	0.00889	0.02925
0	33	25759.9	388.2	0.000000201	0.00000	0.00051	0.00004	0.00152
0	34	25760.1	388.2	0.000001578	0.00002	0.00363	0.00006	0.00264
0	35	25762.9	388.2	0.000122513	0.00157	0.02677	0.00636	0.02843
0	36	26855.7	372.4	0.018268781	0.22395	0.17149	0.43490	0.07352
0	37	27129.6	368.6	0.003099072	0.03761	0.18581	0.01232	0.05413
0	38	27255.6	366.9	0.009673751	0.11685	0.11414	0.23599	0.21938
0	39	27721.7	360.7	0.055893951	0.66377	0.28514	0.71454	0.26813
0	40	27745.4	360.4	0.010182858	0.12082	0.26351	0.22351	0.03784

[Cu_2(^{Me}cAAC)(η^2-HCCPh)($PMes_2$)]

Table S24: calculated absorption data for [Cu(^{Me}cAAC)(η^2-HCCPh)(PPh_2)]

ABSORPTION SPECTRUM VIA TRANSITION ELECTRIC DIPOLE MOMENTS

State	Energy (cm-1)	Wavelength (nm)	fosc	T2 (au**2)	TX (au)	TY (au)	TZ (au)
1	21091.0	474.1	0.010133341	0.15817	-0.00195	-0.06831	0.39179
2	21703.1	460.8	0.188373194	2.85741	-0.73988	-1.14635	-0.99793
3	25924.0	385.7	0.049873830	0.63335	0.25764	0.01672	-0.75279
4	26971.4	370.8	0.020803097	0.25392	0.08226	-0.31673	0.38319
5	27344.0	365.7	0.001503680	0.01810	-0.05952	-0.06327	0.10275
6	27512.8	363.5	0.006222474	0.07446	-0.08421	-0.25112	0.06562
7	28132.8	355.5	0.014956155	0.17502	0.10180	-0.17082	-0.36807
8	28866.3	346.4	0.060348583	0.68826	-0.47060	-0.15509	0.66539
9	30730.8	325.4	0.018317004	0.19623	0.02549	0.16149	0.41170
10	30969.2	322.9	0.077110347	0.81971	0.39581	-0.64614	0.49552
11	32328.7	309.3	0.001307063	0.01331	-0.02927	0.03624	0.10555
12	32935.0	303.6	0.211181375	2.11093	-0.50911	1.09580	0.80681
13	33787.4	296.0	0.131194893	1.27832	0.60834	-0.66883	-0.67890
14	34656.4	288.5	0.023515353	0.22338	0.12217	-0.27771	0.36240
15	34766.4	287.6	0.007227589	0.06844	-0.04442	-0.07775	-0.24581
16	35597.6	280.9	0.003220338	0.02978	0.12166	-0.05168	0.11095
17	36627.0	273.0	0.048118268	0.43250	-0.45682	-0.26563	0.39148
18	37390.7	267.4	0.010831425	0.09537	0.14112	0.22736	-0.15414
19	37496.9	266.7	0.009151754	0.08035	0.16456	-0.18900	0.13247
20	37681.6	265.4	0.030991542	0.27076	-0.41830	-0.24192	0.19303
21	18139.0	551.3	spin forbidden (mult=3)				
22	20786.0	481.1	spin forbidden (mult=3)				
23	23499.0	425.5	spin forbidden (mult=3)				
24	23850.5	419.3	spin forbidden (mult=3)				
25	25680.4	389.4	spin forbidden (mult=3)				
26	26908.7	371.6	spin forbidden (mult=3)				
27	26949.0	371.1	spin forbidden (mult=3)				
28	27286.9	366.5	spin forbidden (mult=3)				
29	27568.3	362.7	spin forbidden (mult=3)				
30	27903.7	358.4	spin forbidden (mult=3)				
31	28701.0	348.4	spin forbidden (mult=3)				
32	30451.5	328.4	spin forbidden (mult=3)				
33	31076.4	321.8	spin forbidden (mult=3)				
34	31620.1	316.3	spin forbidden (mult=3)				
35	31691.2	315.5	spin forbidden (mult=3)				
36	32564.7	307.1	spin forbidden (mult=3)				
37	32880.9	304.1	spin forbidden (mult=3)				
38	33412.9	299.3	spin forbidden (mult=3)				
39	34358.4	291.0	spin forbidden (mult=3)				
40	34405.9	290.6	spin forbidden (mult=3)				

Table S25: calculated SOC-corrected absorption data for [Cu(^{Me}cAAC)(η^2-HCCPh)(PPh_2)]

SPIN ORBIT CORRECTED ABSORPTION SPECTRUM VIA TRANSITION ELECTRIC DIPOLE MOMENTS

States		Energy (cm-1)	Wavelength (nm)	fosc	T2 (au**2)	TX (au)	TY (au)	TZ (au)
0	1	18136.4	551.4	0.000000883	0.00002	0.00016	0.00231	0.00326
0	2	18136.4	551.4	0.000000709	0.00001	0.00284	0.00216	0.00034
0	3	18138.1	551.3	0.000022164	0.00040	0.00812	0.00183	0.01825
0	4	20788.4	481.0	0.000071697	0.00114	0.01543	0.01856	0.02351
0	5	20788.8	481.0	0.000001547	0.00002	0.00061	0.00345	0.00349
0	6	20788.9	481.0	0.000004227	0.00007	0.00525	0.00086	0.00621
0	7	21093.7	474.1	0.010145959	0.15835	0.00170	0.06745	0.39217
0	8	21701.8	460.8	0.187704987	2.84744	0.73822	1.14512	0.99557
0	9	23483.4	425.8	0.000005000	0.00007	0.00486	0.00636	0.00247
0	10	23484.5	425.8	0.000015799	0.00022	0.00548	0.01340	0.00343
0	11	23493.6	425.6	0.000124362	0.00174	0.01790	0.03028	0.02248
0	12	23847.1	419.3	0.000002394	0.00003	0.00135	0.00347	0.00438
0	13	23847.5	419.3	0.000034264	0.00047	0.00908	0.01741	0.00936
0	14	23849.1	419.3	0.000030716	0.00042	0.00765	0.01121	0.01549
0	15	25681.6	389.4	0.000020130	0.00026	0.00549	0.01194	0.00923
0	16	25683.9	389.3	0.000010077	0.00013	0.00270	0.00178	0.01090
0	17	25684.0	389.3	0.000003861	0.00005	0.00037	0.00401	0.00577
0	18	25926.8	385.7	0.049610045	0.62994	0.25794	0.01519	0.75045
0	19	26877.2	372.1	0.010296809	0.12612	0.05576	0.21894	0.27401
0	20	26910.1	371.6	0.000000600	0.00001	0.00043	0.00084	0.00254
0	21	26910.2	371.6	0.000003235	0.00004	0.00009	0.00429	0.00460
0	22	26929.0	371.3	0.003453788	0.04222	0.03220	0.12413	0.16056
0	23	26950.4	371.1	0.000010900	0.00013	0.00163	0.00655	0.00936
0	24	26950.7	371.0	0.000001824	0.00002	0.00222	0.00302	0.00287
0	25	26972.1	370.8	0.006856367	0.08369	0.04680	0.18676	0.21591
0	26	27289.8	366.4	0.000002169	0.00003	0.00093	0.00286	0.00414
0	27	27290.6	366.4	0.000001711	0.00002	0.00253	0.00377	0.00010
0	28	27290.6	366.4	0.000000163	0.00000	0.00128	0.00002	0.00057
0	29	27347.5	365.7	0.001572671	0.01893	0.05868	0.06569	0.10570
0	30	27516.3	363.4	0.006163456	0.07374	0.08442	0.24987	0.06464
0	31	27571.9	362.7	0.000001776	0.00002	0.00042	0.00381	0.00255
0	32	27572.1	362.7	0.000000185	0.00000	0.00098	0.00105	0.00039
0	33	27572.2	362.7	0.000022528	0.00027	0.00411	0.01491	0.00545
0	34	27906.9	358.3	0.000000727	0.00001	0.00154	0.00013	0.00249
0	35	27907.1	358.3	0.000003608	0.00004	0.00089	0.00644	0.00057
0	36	27908.3	358.3	0.000021942	0.00026	0.00457	0.01429	0.00582
0	37	28136.3	355.4	0.014893306	0.17426	0.10213	0.17159	0.36659
0	38	28704.3	348.4	0.000001678	0.00002	0.00303	0.00070	0.00309
0	39	28704.4	348.4	0.000007020	0.00008	0.00566	0.00003	0.00696
0	40	28704.9	348.4	0.000010875	0.00012	0.00406	0.00754	0.00717
0	41	28869.8	346.4	0.060443796	0.68926	0.47025	0.15571	0.66624
0	42	30400.9	328.9	0.000024221	0.00026	0.00314	0.01575	0.00212
0	43	30402.9	328.9	0.000075801	0.00082	0.00474	0.02117	0.01871
0	44	30442.4	328.5	0.000153772	0.00166	0.02034	0.02062	0.02871
0	45	30731.7	325.4	0.019066297	0.20425	0.04148	0.13429	0.42953
0	46	30923.6	323.4	0.065073410	0.69277	0.36771	0.61178	0.42811
0	47	31064.9	321.9	0.000005510	0.00006	0.00066	0.00322	0.00690
0	48	31065.2	321.9	0.000006551	0.00007	0.00483	0.00408	0.00542
0	49	31093.8	321.6	0.010672212	0.11299	0.13678	0.22116	0.21301
0	50	31623.1	316.2	0.000003656	0.00004	0.00040	0.00139	0.00600
0	51	31623.5	316.2	0.000003325	0.00003	0.00250	0.00236	0.00477
0	52	31623.6	316.2	0.000003252	0.00003	0.00172	0.00278	0.00481
0	53	31691.9	315.5	0.000025367	0.00026	0.00440	0.00294	0.01535
0	54	31692.7	315.5	0.000020294	0.00021	0.00663	0.01262	0.00276
0	55	31693.7	315.5	0.000007392	0.00008	0.00351	0.00714	0.00366
0	56	32154.9	311.0	0.000623667	0.00639	0.02643	0.01606	0.07368
0	57	32496.7	307.7	0.000073379	0.00074	0.01568	0.02219	0.00229
0	58	32511.5	307.6	0.000043608	0.00044	0.00033	0.00700	0.01981
0	59	32560.3	307.1	0.000174806	0.00177	0.00587	0.00246	0.04156

0	60	32685.7	305.9	0.000143323	0.00144	0.01887	0.03229	0.00667
0	61	32693.2	305.9	0.000330817	0.00333	0.02259	0.05203	0.01066
0	62	32787.4	305.0	0.003833107	0.03849	0.04793	0.12187	0.14608
0	63	32934.8	303.6	0.205695593	2.05611	0.50175	1.08356	0.79388
0	64	33274.4	300.5	0.001219524	0.01207	0.06563	0.07706	0.04267
0	65	33401.9	299.4	0.000020340	0.00020	0.00618	0.00691	0.01070
0	66	33521.7	298.3	0.001354308	0.01330	0.02769	0.07641	0.08182
0	67	33786.2	296.0	0.125291083	1.22083	0.60096	0.65044	0.66076
0	68	34008.5	294.0	0.006809454	0.06592	0.10246	0.17749	0.15466
0	69	34392.8	290.8	0.000232942	0.00223	0.00436	0.01223	0.04540
0	70	34397.4	290.7	0.000729813	0.00698	0.00701	0.01112	0.08254
0	71	34399.8	290.7	0.000057220	0.00055	0.01556	0.01706	0.00380
0	72	34649.1	288.6	0.013338890	0.12674	0.12279	0.17624	0.28390
0	73	34731.3	287.9	0.012393537	0.11748	0.05869	0.17024	0.29164
0	74	34794.7	287.4	0.000319640	0.00302	0.00461	0.02560	0.04845
0	75	35333.3	283.0	0.002630589	0.02451	0.03154	0.12084	0.09441
0	76	35844.3	279.0	0.002948481	0.02708	0.11705	0.04983	0.10439
0	77	36637.1	272.9	0.047429457	0.42619	0.45228	0.26116	0.39170
0	78	37409.1	267.3	0.010645630	0.09368	0.13113	0.22765	0.15705
0	79	37501.8	266.7	0.008969601	0.07874	0.16647	0.18507	0.12952
0	80	37687.4	265.3	0.031646761	0.27644	0.42087	0.24585	0.19716

[Cu(^{Me}cAAC)(η^1-CCP)(PHMes$_2$)]

Table S26: calculated absorption data for [Cu(MecAAC)(η1-CCPh)(PHPh$_2$)]

ABSORPTION SPECTRUM VIA TRANSITION ELECTRIC DIPOLE MOMENTS

State	Energy (cm-1)	Wavelength (nm)	fosc	T2 (au**2)	TX (au)	TY (au)	TZ (au)
1	25802.0	387.6	0.055796254	0.71191	-0.30542	-0.77436	0.13781
2	29078.7	343.9	0.051785731	0.58629	0.25841	0.14499	-0.70604
3	30387.8	329.1	0.100645948	1.09037	-0.38220	-0.85125	-0.46869
4	30974.1	322.9	0.157811290	1.67732	-0.06012	-0.56217	1.16519
5	31666.8	315.8	0.017541891	0.18237	-0.18245	-0.27444	-0.27159
6	32644.6	306.3	0.145435007	1.46667	-0.32705	-0.72623	0.91231
7	33073.3	302.4	0.022384578	0.22282	-0.12402	-0.07358	-0.44947
8	33697.9	296.8	0.191884743	1.87462	0.39961	0.58917	-1.16953
9	34197.4	292.4	0.021444391	0.20644	0.00273	-0.09780	-0.44370
10	34249.8	292.0	0.050692273	0.48726	-0.21774	-0.24954	-0.61447
11	35058.4	285.2	0.026179971	0.24584	-0.01976	0.17020	-0.46527
12	35903.0	278.5	0.127161721	1.16601	0.16593	0.79303	-0.71384
13	36133.2	276.8	0.016938475	0.15433	0.30060	0.25066	0.03373
14	36281.5	275.6	0.016614774	0.15076	0.24466	-0.21749	0.20880
15	36317.9	275.3	0.024838463	0.22515	0.22822	0.33584	0.24552
16	36416.3	274.6	0.007947279	0.07185	-0.09415	-0.17212	0.18263
17	36640.9	272.9	0.006431215	0.05778	0.16657	-0.03810	0.16907
18	37324.4	267.9	0.052988192	0.46737	0.29936	0.49242	-0.36780
19	37873.8	264.0	0.008273697	0.07192	0.13911	0.16570	-0.15847
20	38149.6	262.1	0.075839836	0.65446	-0.49963	-0.41407	0.48309
21	22366.7	447.1	spin forbidden (mult=3)				
22	25176.0	397.2	spin forbidden (mult=3)				
23	25875.8	386.5	spin forbidden (mult=3)				
24	27755.5	360.3	spin forbidden (mult=3)				
25	29272.5	341.6	spin forbidden (mult=3)				
26	30969.6	322.9	spin forbidden (mult=3)				
27	31379.8	318.7	spin forbidden (mult=3)				
28	31471.6	317.7	spin forbidden (mult=3)				
29	31692.0	315.5	spin forbidden (mult=3)				
30	31922.8	313.3	spin forbidden (mult=3)				
31	32367.1	309.0	spin forbidden (mult=3)				
32	33027.3	302.8	spin forbidden (mult=3)				
33	33464.7	298.8	spin forbidden (mult=3)				
34	33614.4	297.5	spin forbidden (mult=3)				
35	34233.5	292.1	spin forbidden (mult=3)				
36	34351.2	291.1	spin forbidden (mult=3)				
37	34546.8	289.5	spin forbidden (mult=3)				
38	34805.5	287.3	spin forbidden (mult=3)				
39	35136.5	284.6	spin forbidden (mult=3)				
40	35248.3	283.7	spin forbidden (mult=3)				

Table S27: calculated SOC-corrected absorption data for [Cu(MecAAC)(η1-CCPh)(PHPh$_2$)]

SPIN ORBIT CORRECTED ABSORPTION SPECTRUM VIA TRANSITION ELECTRIC DIPOLE MOMENTS

States		Energy (cm-1)	Wavelength (nm)	fosc	T2 (au**2)	TX (au)	TY (au)	TZ (au)
0	1	22345.8	447.5	0.000002826	0.00004	0.00357	0.00537	0.00023
0	2	22346.3	447.5	0.000007259	0.00011	0.00419	0.00862	0.00388
0	3	22360.9	447.2	0.000262116	0.00386	0.01309	0.00466	0.06055
0	4	25167.8	397.3	0.000820210	0.01073	0.04117	0.09502	0.00235
0	5	25177.0	397.2	0.000001623	0.00002	0.00131	0.00435	0.00077
0	6	25177.4	397.2	0.000006678	0.00009	0.00340	0.00734	0.00468

0	7	25560.8	391.2	0.029710284	0.38266	0.22405	0.56813	0.09841
0	8	25891.8	386.2	0.000003846	0.00005	0.00400	0.00356	0.00450
0	9	25892.8	386.2	0.000038297	0.00049	0.00724	0.01814	0.01026
0	10	26119.6	382.9	0.025381170	0.31990	0.20380	0.51816	0.09942
0	11	27748.4	360.4	0.000004298	0.00005	0.00138	0.00032	0.00700
0	12	27749.2	360.4	0.000005242	0.00006	0.00350	0.00632	0.00317
0	13	27749.8	360.4	0.000004178	0.00005	0.00333	0.00557	0.00272
0	14	29062.6	344.1	0.050682835	0.57412	0.25331	0.14038	0.70018
0	15	29267.7	341.7	0.000008522	0.00010	0.00147	0.00872	0.00421
0	16	29267.9	341.7	0.000003376	0.00004	0.00277	0.00479	0.00271
0	17	29272.8	341.6	0.001123145	0.01263	0.04774	0.04278	0.09232
0	18	30376.4	329.2	0.098834486	1.07114	0.37500	0.83931	0.47547
0	19	30931.0	323.3	0.099167546	1.05548	0.07174	0.48536	0.90264
0	20	30963.3	323.0	0.000075508	0.00080	0.01551	0.02239	0.00783
0	21	30964.4	323.0	0.000059337	0.00063	0.00471	0.02029	0.01404
0	22	30987.8	322.7	0.056379771	0.59897	0.01862	0.30630	0.71050
0	23	31381.5	318.7	0.000028985	0.00030	0.00593	0.01084	0.01230
0	24	31381.7	318.7	0.000014730	0.00015	0.00242	0.00699	0.00999
0	25	31383.5	318.6	0.000252926	0.00265	0.00545	0.00857	0.05050
0	26	31473.7	317.7	0.000000450	0.00000	0.00012	0.00217	0.00008
0	27	31474.3	317.7	0.000000968	0.00001	0.00062	0.00209	0.00232
0	28	31479.5	317.7	0.000554118	0.00579	0.01223	0.03860	0.06446
0	29	31648.2	316.0	0.011658435	0.12127	0.15240	0.24742	0.19192
0	30	31676.6	315.7	0.000229265	0.00238	0.00703	0.01253	0.04665
0	31	31677.5	315.7	0.000165536	0.00172	0.01729	0.03770	0.00010
0	32	31708.5	315.4	0.007466824	0.07752	0.10396	0.10786	0.23470
0	33	31923.4	313.3	0.000010017	0.00010	0.00154	0.00187	0.00987
0	34	31923.5	313.2	0.000046849	0.00048	0.00528	0.01034	0.01866
0	35	31925.5	313.2	0.000205381	0.00212	0.01502	0.00657	0.04300
0	36	32365.5	309.0	0.000099584	0.00101	0.00974	0.02275	0.02001
0	37	32365.9	309.0	0.000027484	0.00028	0.00670	0.01532	0.00001
0	38	32366.3	309.0	0.000020811	0.00021	0.00745	0.01008	0.00738
0	39	32647.3	306.3	0.142388745	1.43583	0.32597	0.72208	0.89899
0	40	33004.8	303.0	0.009885016	0.09860	0.07153	0.04134	0.30294
0	41	33026.7	302.8	0.000161800	0.00161	0.00285	0.01466	0.03728
0	42	33028.1	302.8	0.000050181	0.00050	0.00072	0.00784	0.02094
0	43	33059.4	302.5	0.015905034	0.15839	0.08143	0.03265	0.38818
0	44	33471.3	298.8	0.004509020	0.04435	0.06131	0.09832	0.17585
0	45	33475.7	298.7	0.000248614	0.00244	0.01774	0.01949	0.04184
0	46	33479.1	298.7	0.000425532	0.00418	0.02341	0.01774	0.05763
0	47	33607.2	297.6	0.012593936	0.12337	0.10909	0.15990	0.29309
0	48	33613.8	297.5	0.000163274	0.00160	0.02089	0.02028	0.02741
0	49	33622.0	297.4	0.010222887	0.10010	0.11505	0.16311	0.24547
0	50	33701.1	296.7	0.161170187	1.57440	0.36676	0.53608	1.07355
0	51	34167.2	292.7	0.014685961	0.14150	0.04041	0.13887	0.34726
0	52	34220.8	292.2	0.001981355	0.01906	0.06630	0.04313	0.11316
0	53	34226.6	292.2	0.000099706	0.00096	0.01255	0.00084	0.02830
0	54	34243.1	292.0	0.010069972	0.09681	0.15798	0.15792	0.21660
0	55	34278.9	291.7	0.037021720	0.35555	0.11980	0.16246	0.56108
0	56	34349.6	291.1	0.000106828	0.00102	0.01552	0.02048	0.01907
0	57	34350.7	291.1	0.000030961	0.00030	0.00322	0.00675	0.01552
0	58	34357.1	291.1	0.004724016	0.04527	0.05625	0.06147	0.19576
0	59	34573.5	289.2	0.002506710	0.02387	0.01823	0.00258	0.15339
0	60	34579.9	289.2	0.000099629	0.00095	0.00313	0.01138	0.02845
0	61	34584.4	289.1	0.000681222	0.00648	0.00383	0.01882	0.07820
0	62	34812.9	287.2	0.000221887	0.00210	0.01950	0.02693	0.03151
0	63	34816.7	287.2	0.000066096	0.00062	0.00098	0.00958	0.02307
0	64	34821.1	287.2	0.000138238	0.00131	0.00166	0.01097	0.03441
0	65	35055.3	285.3	0.024069611	0.22604	0.01914	0.16277	0.44630
0	66	35127.5	284.7	0.000145973	0.00137	0.02092	0.00718	0.02965
0	67	35137.4	284.6	0.000514614	0.00482	0.00903	0.01095	0.06797
0	68	35146.3	284.5	0.000129749	0.00122	0.01041	0.01276	0.03073
0	69	35266.3	283.6	0.000443069	0.00414	0.00880	0.00968	0.06297
0	70	35285.3	283.4	0.000013288	0.00012	0.00135	0.01000	0.00471
0	71	35289.0	283.4	0.002067186	0.01928	0.01839	0.06105	0.12337
0	72	35917.3	278.4	0.128054701	1.17373	0.16437	0.79451	0.71796
0	73	36148.1	276.6	0.016792097	0.15293	0.29971	0.24986	0.02594

0	74	36289.2	275.6	0.015336887	0.13913	0.24024	0.21368	0.18910
0	75	36332.7	275.2	0.025216994	0.22849	0.23276	0.33044	0.25520
0	76	36435.1	274.5	0.008205579	0.07414	0.08854	0.18265	0.18150
0	77	36658.1	272.8	0.006888036	0.06186	0.16198	0.05143	0.18160
0	78	37339.0	267.8	0.052508142	0.46296	0.30032	0.49016	0.36402
0	79	37899.4	263.9	0.008627503	0.07494	0.14043	0.16753	0.16479
0	80	38163.0	262.0	0.075455056	0.65091	0.49913	0.41238	0.48137

3. Difference density plots

Blue represents electron loss, yellow represents electron gain.

2a [Cu(^{Me}cAAC)(PMes$_2$)]

Table S28: difference densities for 2a

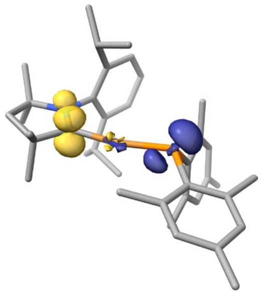	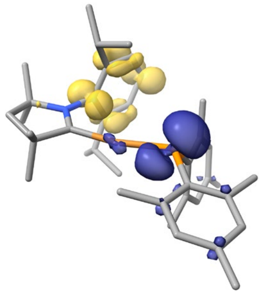	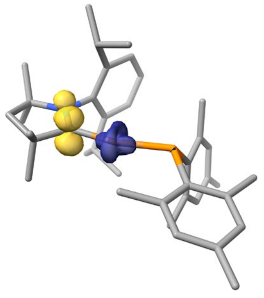	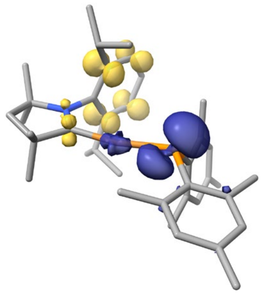	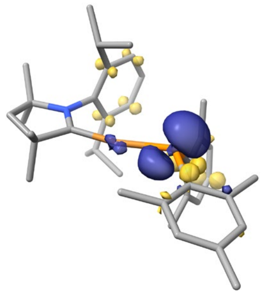
S_1	S_2	S_3	S_4	S_5
T_1	T_2	T_3	T_4	T_5
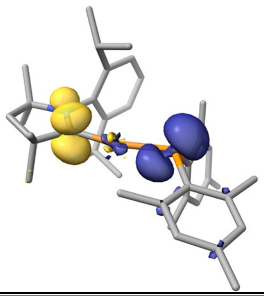	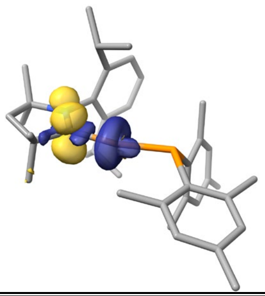	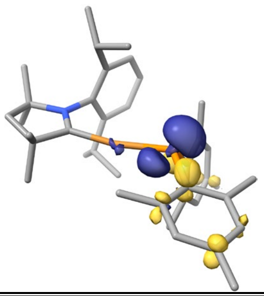	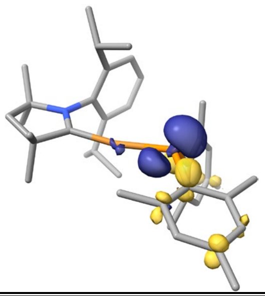	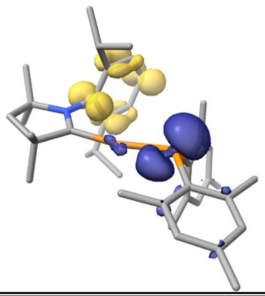
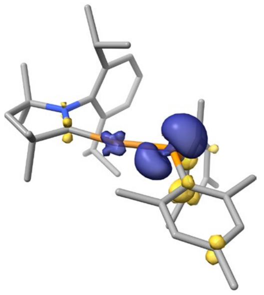	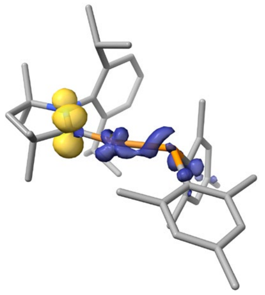	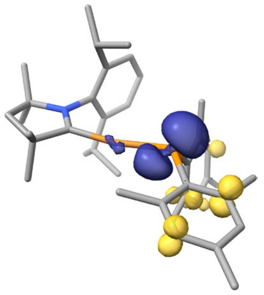	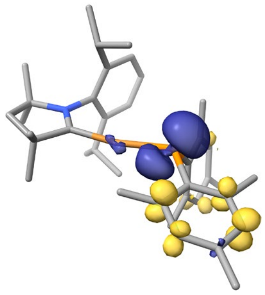	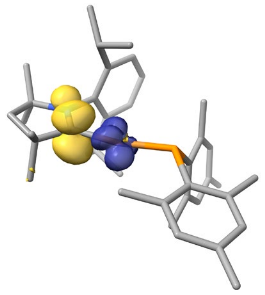
S_6	S_7	S_8	S_9	S_{10}
T_6	T_7	T_8	T_9	T_{10}
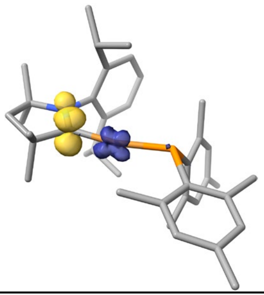	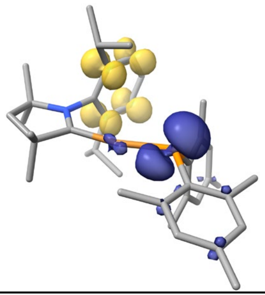	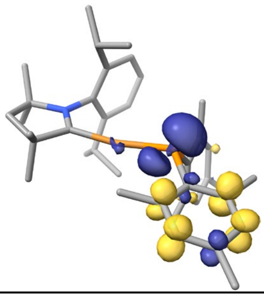	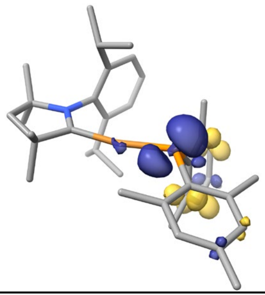	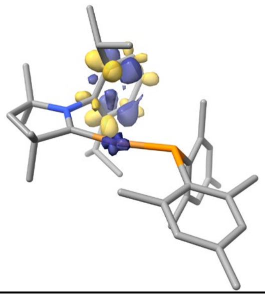

Table S29: transition densities for 2a

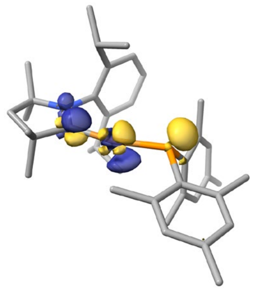 **S_1**	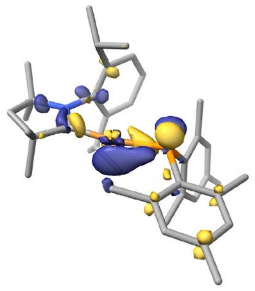 **S_2**	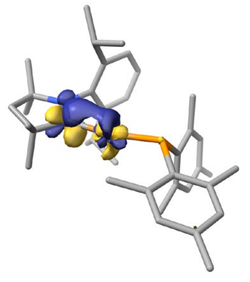 **S_3**	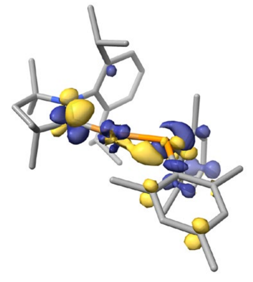 **S_4**	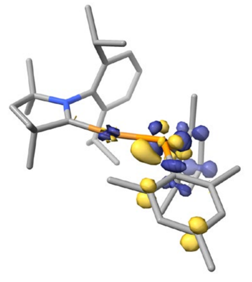 **S_5**
**S_6** 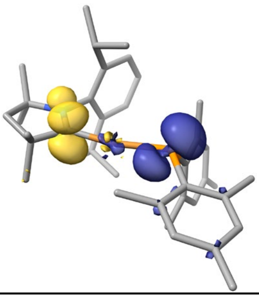	**S_7** 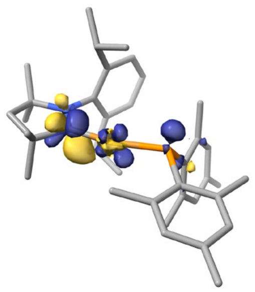	**S_8** 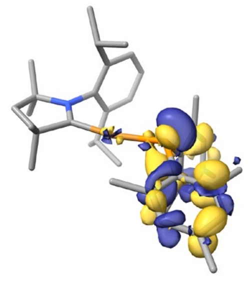	**S_9** 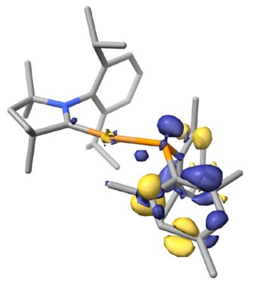	**S_{10}** 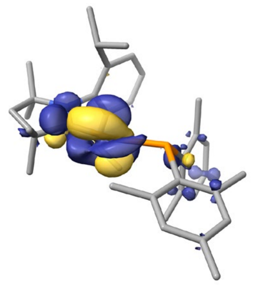

[Cu(^{Me}cAAC)$_2$(μ-PMes$_2$)$_2$]

Table S30: difference densities for [Cu$_2$(^{Me}cAAC)$_2$(μ-PMes$_2$)$_2$]

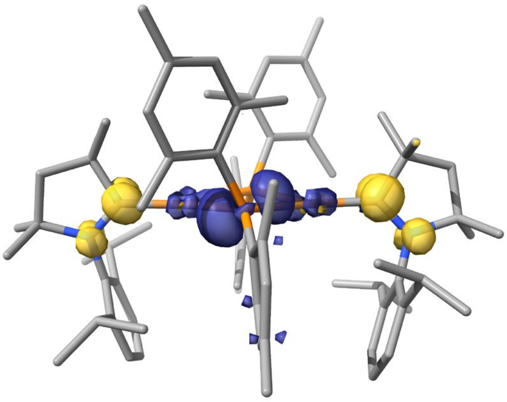<br>**S_1**<br>**T_1**<br>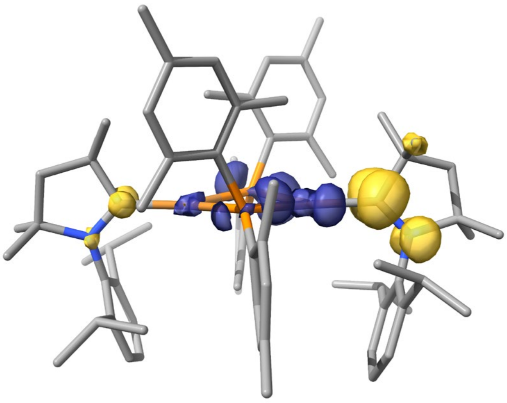	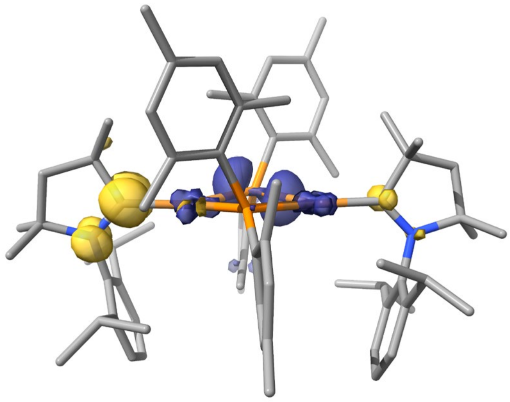<br>**S_2**<br>**T_2**<br>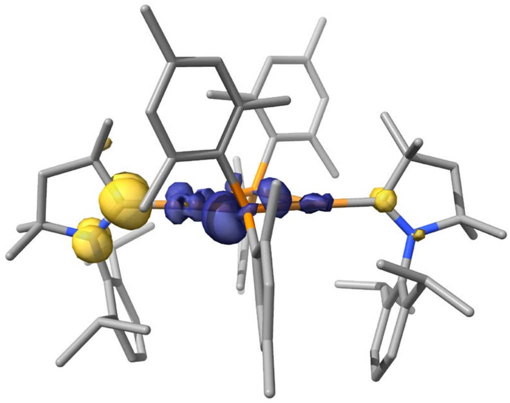
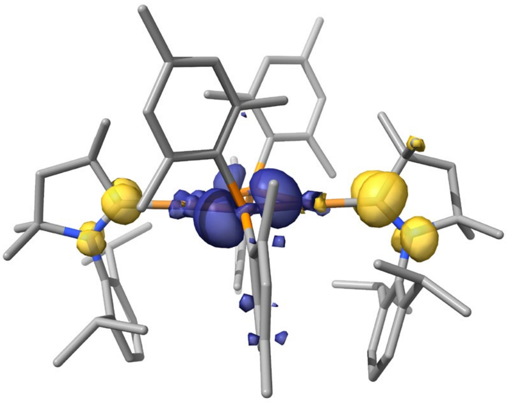<br>**S_3**<br>**T_3**<br>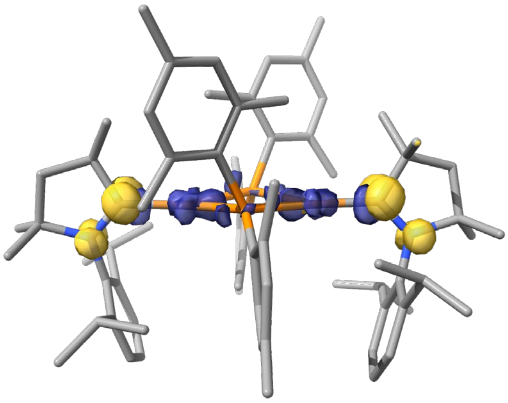	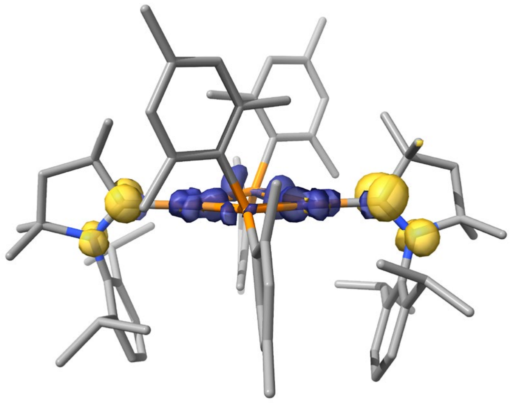<br>**S_4**<br>**T_4**<br>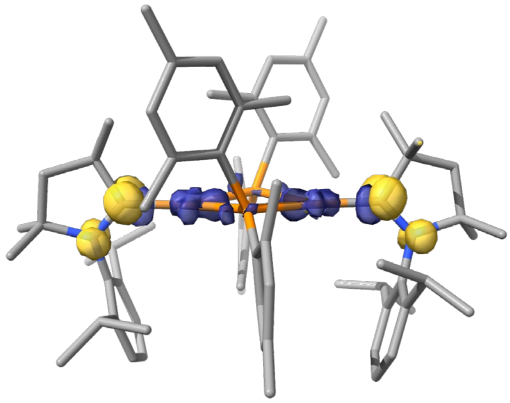

[Cu_2(^{Me}cAAC)(η^2-HCCPh)(PPh_2)]

Table S31: difference densities for [Cu(^{Me}cAAC)(η^1-HCCPh)(PPh_2)]

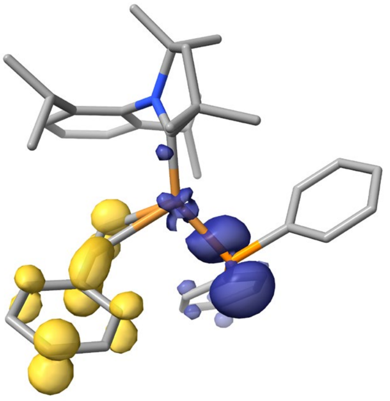<br>**S_1**<br>**T_1**<br>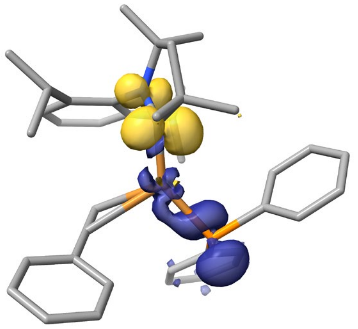	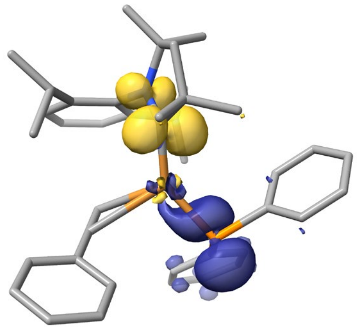<br>**S_2**<br>**T_2**<br>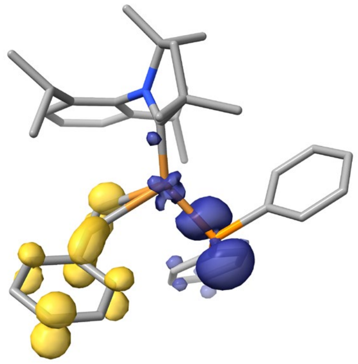
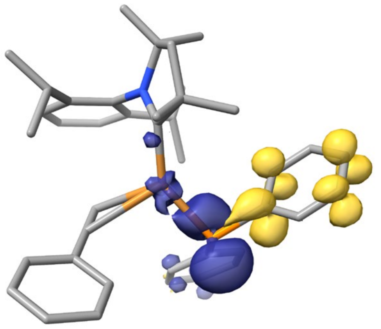<br>**S_3**<br>**T_3**<br>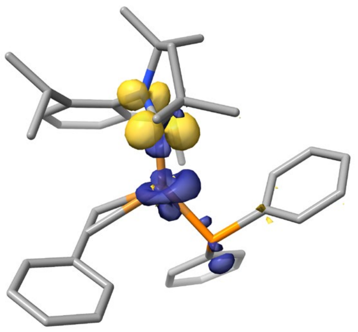	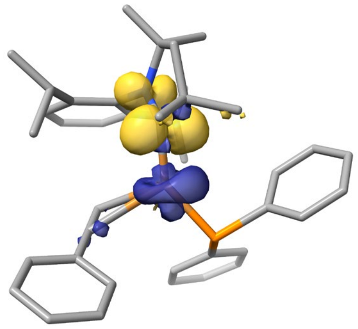<br>**S_4**<br>**T_4**<br>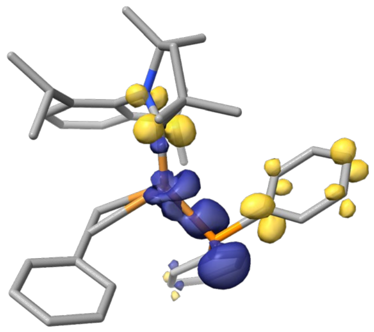

$[Cu_2(^{Me}cAAC)(\eta^2\text{-}HCCPh)(PPh_2)]$

Table S32: difference densities for [Cu(mecAAC)(η2-HCCPh)(PHPh2)]

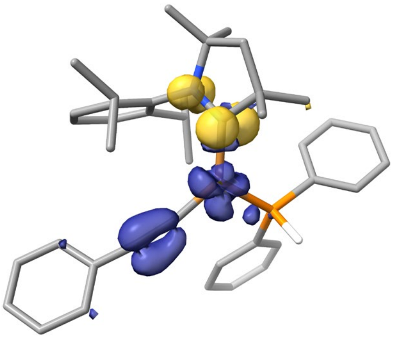<br>**S_1**<br>**T_1**<br>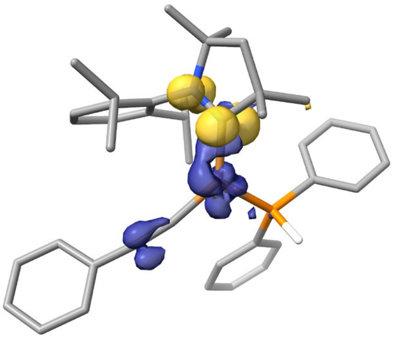	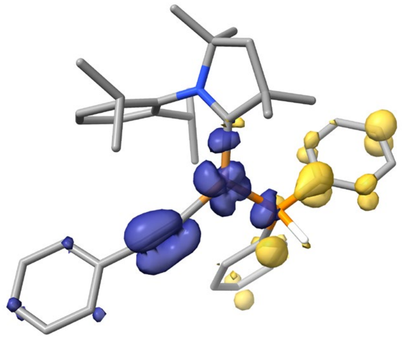<br>**S_2**<br>**T_2**<br>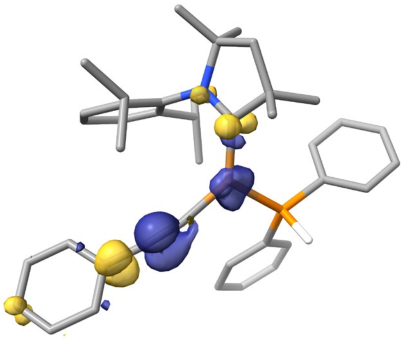
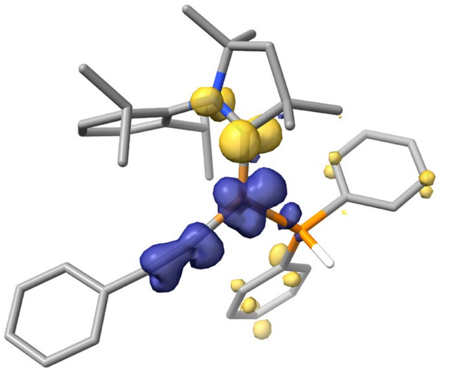<br>**S_3**<br>**T_3**<br>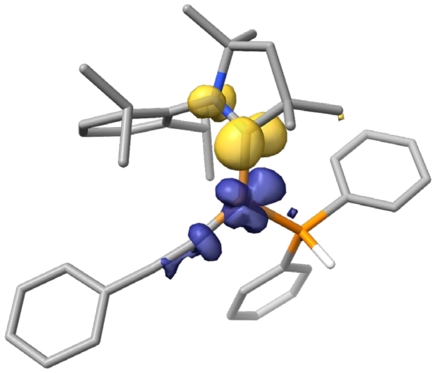	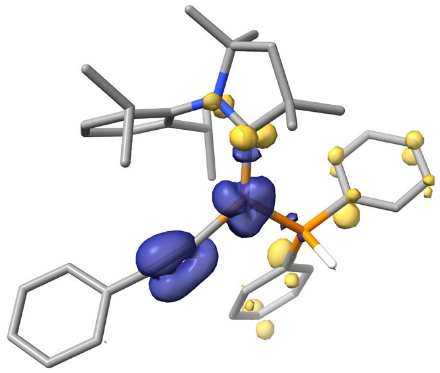<br>**S_4**<br>**T_4**<br>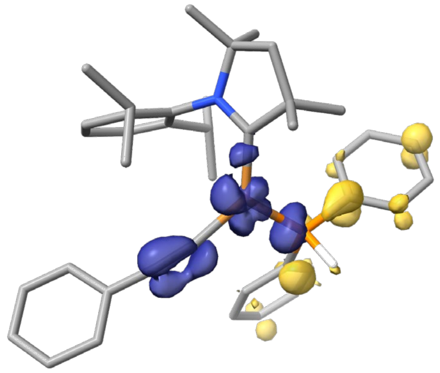

Figures in this document

Figure S100: Absorption spectra of 2a 3

Figure S101: Absorption spectra of 2b 4

Figure S102: Absorption spectra of 2c 5

Figure S103: Absorption spectra of 2d 6

Figure S104: Normalized Emission of complexes 2a - 2d in toluene solution at 297 K 8

Figure S105: Normalized Emission and excitation spectra of 2a - 2d in solid state at 297 K 8

Figure S106: Normalized Emission and Excitation spectra of 2a - 2d in solid state at 77 K 9

Figure S107: Normalized Emission spectra at 297 K and 77 K comparing the x-intercept o the emission bands 9

Figure S108: Normalized Emission spectra of 2c in solid state from 77 K to77K in 10 K increments 10

Figure S109: Emission spectra of 2a-d in PS (left) and PMMA (right) (2wt-% respectively) at 297 K. 10

Figure S110: Observed excited states decay (grey), fit curves and residuals (colored) for 2a - 2d in solid state at 297 K 11

Figure S111: Observed excited states decay (grey), fit curves and residuals (colored) for 2a - 2d in solid state at 297 K 12

Figure S84: Observed excited states decay (grey), fit curves and residuals (colored) for 2a-d in PS (2wt-%) 13

Figure S85: Observed excited states decay (grey), fit curves and residuals (colored) for 2a-d in PMMA (2wt-%) 14

Tables in this document

Table S2: Summary of photophysical data for compounds 2a - 2d. 7

Table S3: Fit-data for all excited states decay of 2c from 77 K to 297 K in 10 K increments 15

Table S4: Optimized Geomtry Atom Coordinates of 2a 16

Table S5: Optimized Geomtry Atom Coordinates of 2b 18

Table S6: Optimized Geomtry Atom Coordinates of 2c 20

Table S7: Optimized Geomtry Atom Coordinates of 2d 22

Table S8: Optimized Geomtry Atom Coordinates of $[Cu_2(^{Me}cAAC)_2(\mu\text{-}PMes_2)_2]$ 24

Table S9: Optimized Geomtry Atom Coordinates of $[Cu(^{Me}cAAC)(\eta^2\text{-}HCCCPh)(PPh_2)]$ 27

Table S10: Optimized Geomtry Atom Coordinates of $[Cu(^{Me}cAAC)(\eta^1\text{-}CCCPh)(PHPh_2)]$ 29

Table S11: calculated absorption data for 2a 31

Table S12: calculated SOC-corrected absorption data for 2a 32

Table S13: calculated absorption data for 2b 34

Table S14: calculated SOC-corrected absorption data for 2b 35

Table S15: calculated absorption data for 2c 37

Table S16: calculated SOC-corrected absorption data for 2c 38

Table S17: calculated absorption data for 2d 40

Table S18: calculated CD-spectral data for 2d 40

Table S19: calculated absorpton dissymmetry for 2d 41

Table S20: calculated SOC-corrected absorption data for 2d 41

Table S21: calculated SOC-corrected CD spectral data for 2d 43

Table S22: calculated absorption for $[Cu_2(^{Me}cAAC)_2(\mu\text{-}PMes_2)_2]$ 45

Table S23: calculated SOC-corrected absorption data for $[Cu_2(^{Me}cAAC)_2(\mu\text{-}PMes_2)_2]$ 45

Table S24: calculated absorption data for $[Cu(^{Me}cAAC)(\eta^2\text{-}HCCPh)(PPh_2)]$ 47

Table S25: calculated SOC-corrected absorption data for $[Cu(^{Me}cAAC)(\eta^2\text{-}HCCPh)(PPh_2)]$ 48

Table S26: calculated absorption data for $[Cu(MecAAC)(\eta 1\text{-}CCPh)(PHPh_2)]$ 50

Table S27: calculated SOC-corrected absorption data for $[Cu(MecAAC)(\eta 1\text{-}CCPh)(PHPh_2)]$ 50

Table S28: difference densities for 2a 53

Table S29: transition densities for 2a 54

Table S30: difference densities for $[Cu_2(^{Me}cAAC)_2(\mu\text{-}PMes_2)_2]$ 55

Table S31: difference densities for [Cu(^{Me}cAAC)(η^1-HCCPh)(PPh_2)] 56

Table S32: difference densities for [Cu(mecAAC)(η2-HCCPh)(PHPh2)] 57

5. REFERENCES

[1] C. Würth, M. Grabolle, J. Pauli, M. Spieles, U. Resch-Genger, *Nature protocols* **2013**, *8*, 1535.

[2] a) F. Neese, *WIREs Comput Mol Sci* **2018**, *8*; b) F. Neese, *WIREs Comput Mol Sci* **2012**, *2*, 73.

[3] J. P. Perdew, M. Ernzerhof, K. Burke, *J. Chem. Phys.* **1996**, *105*, 9982.

[4] W. Kohn, L. J. Sham, *Phys. Rev.* **1965**, *140*, A1133-A1138.

[5] a) S. Grimme, J. Antony, S. Ehrlich, H. Krieg, *J. Chem. Phys.* **2010**, *132*, 154104; b) S. Grimme, S. Ehrlich, L. Goerigk, *J. Comput. Chem.* **2011**, *32*, 1456; c) E. van Lenthe, A. Ehlers, E.-J. Baerends, *J. Chem. Phys.* **1999**, *110*, 8943; d) F. Weigend, R. Ahlrichs, *Phys. Chem. Chem. Phys.* **2005**, *7*, 3297.

[6] V. Barone, M. Cossi, *J. Phys. Chem. A* **1998**, *102*, 1995.
