# Supplementary material for: Organophosphides: A New Class of Luminophore Ligands for Copper(I) Carbene Based TADF Emitters and Photocatalysts
Source: Angew Chem Int Ed Engl. 2026 Feb 25;65(13):e18530. doi: 10.1002/anie.202518530 (PMC13007583; doi:10.1002/anie.202518530)

checkCIF/PLATON report

Structure factors have been supplied for datablock(s) PCRX1021-PCRB1031

THIS REPORT IS FOR GUIDANCE ONLY. IF USED AS PART OF A REVIEW PROCEDURE FOR PUBLICATION, IT SHOULD NOT REPLACE THE EXPERTISE OF AN EXPERIENCED CRYSTALLOGRAPHIC REFEREE.

No syntax errors found. CIF dictionary Interpreting this report

Datablock: PCRX1021-PCRB1031

```
Bond precision:   C-C = 0.0031 A          Wavelength=0.71073

Cell:             a=17.5516(9)     b=13.3084(6)       c=15.9247(7)
                  alpha=90         beta=90.881(2)     gamma=90
Temperature:      100 K

                 Calculated                      Reported
Volume           3719.3(3)                       3719.3(3)
Space group      P 21/c                          P 1 21/c 1
Hall group       -P 2ybc                         -P 2ybc
Moiety formula   C41 H57 Cu N P                  C41 H57 Cu N P
Sum formula      C41 H57 Cu N P                  C41 H57 Cu N P
Mr               658.40                          658.44
Dx,g cm-3        1.176                           1.176
Z                4                               4
Mu (mm-1)        0.658                           0.658
F000             1416.0                          1418.5
F000'            1418.04
h,k,lmax         24,18,22                        24,18,22
Nref             10888                           10851
Tmin,Tmax        0.915,0.964                     0.626,0.746
Tmin'            0.868

Correction method= # Reported T Limits: Tmin=0.626 Tmax=0.746
AbsCorr = MULTI-SCAN

Data completeness= 0.997           Theta(max)= 30.040

R(reflections)= 0.0477( 8398)                       wR2(reflections)=
                                                    0.1049( 10851)
S = 1.059                   Npar= 409
```

The following ALERTS were generated. Each ALERT has the format
test-name_ALERT_alert-type_alert-level.
Click on the hyperlinks for more details of the test.

Alert level C

```
PLAT906_ALERT_3_C Large K Value in the Analysis of Variance ......      2.991 Check
PLAT911_ALERT_3_C Missing FCF Refl Between Thmin & STh/L=    0.600         23 Report
                2  0  0,  -1  1  1,   0  1  1,  -1  0  2,   0  0  2,   1  0  2,
                0  1 11,  -1  0 12,   0  0 12,   0  1 12,   0  1 13,  -1  0 14,
               -1  1 14,   0  0 14,   0  1 14,  -1  1 15,  -2  0 16,  -1  0 16,
               -1  1 16,  -2  1 17,  -3  0 18,  -2  0 18,  -2  1 18,
```

Alert level G

```
PLAT068_ALERT_1_G Reported F000 Differs from Calcd (or Missing)...     Please Check
PLAT328_ALERT_4_G Possible Missing H on sp3? Phosphorus ..........       P1_1 Check
PLAT720_ALERT_4_G Number of Unusual/Non-Standard Labels ..........        101 Note
              Cu1_1   P1_1    N1_1    C35_1   C1A_1   H1AA_1  H1AB_1  H1AC_1
              C1B_1   H1BA_1  H1BB_1  H1BC_1  C2A_1   H2A_1   C2B_1   H2B_1
              C2C_1   H2C_1   C2D_1   H2D_1   C3A_1   H3AA_1  H3AB_1  H3AC_1
              C3B_1   H3BA_1  H3BB_1  H3BC_1  C3C_1   H3CA_1  H3CB_1  H3CC_1
              C3D_1   H3DA_1  H3DB_1  H3DC_1  C4A_1   H4AA_1  H4AB_1  C4B_1
              H4BA_1  H4BB_1  C5_1    H5A_1   H5B_1   C6A_1   H6AA_1  H6AB_1
              H6AC_1  C6B_1   H6BA_1  H6BB_1  H6BC_1  C7A_1   H7AA_1  H7AB_1
              H7AC_1  C7B_1   H7BA_1  H7BB_1  H7BC_1  C8A_1   H8A_1   C8B_1
              H8B_1   C9A_1   H9AA_1  H9AB_1  H9AC_1  C9B_1   H9BA_1  H9BB_1
              H9BC_1  C10A_1  H10A_1  C10B_1  H10B_1  C11_1   H11_1   C12A_1
              C12B_1  C13A_1  C13B_1  C13C_1  C13D_1  C14A_1  C14B_1  C15_1
              C16_1   C17_1   C18_1   C19A_1  C20A_1  H20A_1  H20B_1  C20B_1
              H20C_1  H20D_1  C21_1   H21A_1  H21B_1
PLAT769_ALERT_4_G CIF Embedded Explicitly Supplied Scattering Data     Please Note
PLAT794_ALERT_5_G Tentative Bond Valency for Cu1_1     (I)       .       0.62 Info
PLAT910_ALERT_3_G Missing # of FCF Reflection(s) Below Theta(Min).          1 Note
                1  0  0,
PLAT912_ALERT_4_G Missing # of FCF Reflections Above STh/L=  0.600         10 Note
PLAT913_ALERT_3_G Missing # of Very Strong Reflections in FCF ....          1 Note
                2  0  0,
PLAT933_ALERT_2_G Number of HKL-OMIT Records in Embedded .res File          3 Note
               -1  0  2,   0  1  1,   2  0  0,
PLAT960_ALERT_3_G Number of Intensities with I  <   - 2*sig(I) ...         22 Check
PLAT978_ALERT_2_G Number C-C Bonds with Positive Residual Density.         15 Info
PLAT982_ALERT_1_G The Cu-f’=    0.3283 Deviates from  IT-value =       0.3201 Check
PLAT983_ALERT_1_G The Cu-f"=    1.2708 Deviates from  IT-Value =       1.2651 Check
```

0 **ALERT level A** = Most likely a serious problem - resolve or explain
0 **ALERT level B** = A potentially serious problem, consider carefully
2 **ALERT level C** = Check. Ensure it is not caused by an omission or oversight
13 **ALERT level G** = General information/check it is not something unexpected

3 ALERT type 1 CIF construction/syntax error, inconsistent or missing data
2 ALERT type 2 Indicator that the structure model may be wrong or deficient
5 ALERT type 3 Indicator that the structure quality may be low
4 ALERT type 4 Improvement, methodology, query or suggestion
1 ALERT type 5 Informative message, check

It is advisable to attempt to resolve as many as possible of the alerts in all categories. Often the minor alerts point to easily fixed oversights, errors and omissions in your CIF or refinement strategy, so attention to these fine details can be worthwhile. In order to resolve some of the more serious problems it may be necessary to carry out additional measurements or structure refinements. However, the purpose of your study may justify the reported deviations and the more serious of these should normally be commented upon in the discussion or experimental section of a paper or in the "special_details" fields of the CIF. checkCIF was carefully designed to identify outliers and unusual parameters, but every test has its limitations and alerts that are not important in a particular case may appear. Conversely, the absence of alerts does not guarantee there are no aspects of the results needing attention. It is up to the individual to critically assess their own results and, if necessary, seek expert advice.

Publication of your CIF in IUCr journals

A basic structural check has been run on your CIF. These basic checks will be run on all CIFs submitted for publication in IUCr journals (*Acta Crystallographica*, *Journal of Applied Crystallography*, *Journal of Synchrotron Radiation*); however, if you intend to submit to *Acta Crystallographica Section C* or *E* or *IUCrData*, you should make sure that full publication checks are run on the final version of your CIF prior to submission.

Publication of your CIF in other journals

Please refer to the *Notes for Authors* of the relevant journal for any special instructions relating to CIF submission.

PLATON version of 29/11/2023; check.def file version of 14/09/2023

Datablock PCRX1021-PCRB1031 - ellipsoid plot

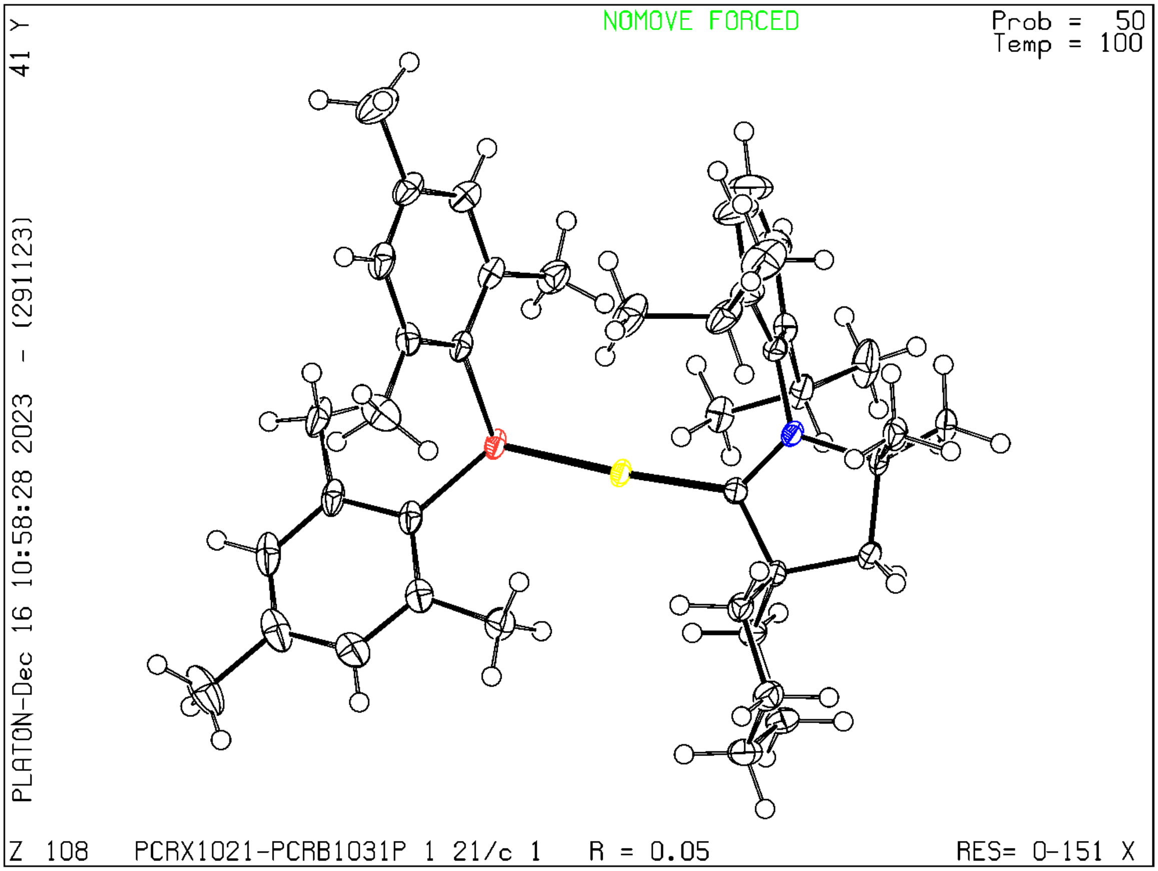

Supplement: Supplementary file 4 — Supporting File 4: anie71068‐sup‐0004‐Data.zip. [file ANIE-65-e18530-s001.zip › checkcif-PCRX1021-PCRB1031-finalcif.pdf]
