# Supplementary material for: Organophosphides: A New Class of Luminophore Ligands for Copper(I) Carbene Based TADF Emitters and Photocatalysts
Source: Angew Chem Int Ed Engl. 2026 Feb 25;65(13):e18530. doi: 10.1002/anie.202518530 (PMC13007583; doi:10.1002/anie.202518530)

checkCIF/PLATON report

Structure factors have been supplied for datablock(s) pcrx1037_pcrb1037_a

THIS REPORT IS FOR GUIDANCE ONLY. IF USED AS PART OF A REVIEW PROCEDURE FOR PUBLICATION, IT SHOULD NOT REPLACE THE EXPERTISE OF AN EXPERIENCED CRYSTALLOGRAPHIC REFEREE.

No syntax errors found. CIF dictionary Interpreting this report

Datablock: pcrx1037_pcrb1037_a

Bond precision:	C-C = 0.0045 A		Wavelength=1.54178
Cell:	a=16.6746(7)	b=13.1698(5)	c=16.0654(6)
	alpha=90	beta=93.351(2)	gamma=90
Temperature:	150 K		

	Calculated	Reported
Volume	3522.0(2)	3521.9(2)
Space group	P 21/c	P 1 21/c 1
Hall group	-P 2ybc	-P 2ybc
Moiety formula	C38 H53 Cu N P	C38 H53 Cu N P
Sum formula	C38 H53 Cu N P	C38 H53 Cu N P
Mr	618.33	618.32
Dx,g cm-3	1.166	1.166
Z	4	4
Mu (mm-1)	1.473	1.473
F000	1328.0	1328.0
F000'	1324.00	
h,k,lmax	19,15,19	19,15,19
Nref	6265	6243
Tmin,Tmax	0.865,0.919	0.615,0.753
Tmin'	0.859	

Correction method= # Reported T Limits: Tmin=0.615 Tmax=0.753
AbsCorr = NONE

Data completeness= 0.996 Theta(max)= 66.923

R(reflections)= 0.0500(5112) wR2(reflections)= 0.1383(6243)

S = 1.034 Npar= 384

The following ALERTS were generated. Each ALERT has the format
test-name_ALERT_alert-type_alert-level.
Click on the hyperlinks for more details of the test.

Alert level C

```
PLAT911_ALERT_3_C Missing FCF Refl Between Thmin & STh/L=    0.597          22 Report
              -19  4  4,  19  2  4,   3 10  5, -17  7  6, -15  9  7, -11 12  7,
                5 10  7,  18  1  7, -15  5  8, -12 11  8, -15  7  9,   9 10  9,
                6 12 10,   8  9 10, -10  4 11, -16  2 12,  11  8 12, -13  2 15,
               -8  8 15,   8  1 17,  -1  2 19,   1  1 19,
```

Alert level G

```
PLAT232_ALERT_2_G Hirshfeld Test Diff (M-X)  Cu01     --C004     .         6.1 s.u.
PLAT328_ALERT_4_G Possible Missing H on sp3? Phosphorus .........        P002 Check
PLAT720_ALERT_4_G Number of Unusual/Non-Standard Labels ..........          90 Note
              Cu01    P002    N003    C004    C005    C006    C007    C008
              C009    C00A    C00B    C00C    H00A    H00B    H00C    C00D
              H00D    C00E    H00E    C00F    H00F    H00G    C00G    C00H
              C00I    C00J    C00K    H00K    C00L    H00H    H00I    H00J
              C00M    H00L    H00M    H00N    C00N    H00O    C00O    C00P
              H00P    H00Q    H00R    C00Q    H00S    H00T    H00U    C00R
              H00V    C00S    H00W    C00T    H00X    H00Y    C00U    C00V
              H00Z    C00W    H00     Ha      Hb      C00X    Hc      Hd
              C00Y    He      Hf      C00Z    Hg      Hh      C010    H01A
              H01B    H01C    C011    H011    C012    H012    C013    H01D
              H01E    H01F    C014    H01G    H01H    H01I    C015    H01J
              H01K    H01L
PLAT794_ALERT_5_G Tentative Bond Valency for Cu01      (I)       .        0.61 Info
PLAT909_ALERT_3_G Percentage of I>2sig(I) Data at Theta(Max) Still         72% Note
PLAT969_ALERT_5_G The 'Henn et al.' R-Factor-gap value ..........       3.256 Note
              Predicted wR2: Based on SigI**2  4.25 or SHELX Weight 13.37
PLAT978_ALERT_2_G Number C-C Bonds with Positive Residual Density.           8 Info
```

---

0 **ALERT level A** = Most likely a serious problem - resolve or explain
0 **ALERT level B** = A potentially serious problem, consider carefully
1 **ALERT level C** = Check. Ensure it is not caused by an omission or oversight
7 **ALERT level G** = General information/check it is not something unexpected

0 ALERT type 1 CIF construction/syntax error, inconsistent or missing data
2 ALERT type 2 Indicator that the structure model may be wrong or deficient
2 ALERT type 3 Indicator that the structure quality may be low
2 ALERT type 4 Improvement, methodology, query or suggestion
2 ALERT type 5 Informative message, check

It is advisable to attempt to resolve as many as possible of the alerts in all categories. Often the minor alerts point to easily fixed oversights, errors and omissions in your CIF or refinement strategy, so attention to these fine details can be worthwhile. In order to resolve some of the more serious problems it may be necessary to carry out additional measurements or structure refinements. However, the purpose of your study may justify the reported deviations and the more serious of these should normally be commented upon in the discussion or experimental section of a paper or in the "special_details" fields of the CIF. checkCIF was carefully designed to identify outliers and unusual parameters, but every test has its limitations and alerts that are not important in a particular case may appear. Conversely, the absence of alerts does not guarantee there are no aspects of the results needing attention. It is up to the individual to critically assess their own results and, if necessary, seek expert advice.

Publication of your CIF in IUCr journals

A basic structural check has been run on your CIF. These basic checks will be run on all CIFs submitted for publication in IUCr journals (*Acta Crystallographica*, *Journal of Applied Crystallography*, *Journal of Synchrotron Radiation*); however, if you intend to submit to *Acta Crystallographica Section C* or *E* or *IUCrData*, you should make sure that full publication checks are run on the final version of your CIF prior to submission.

Publication of your CIF in other journals

Please refer to the *Notes for Authors* of the relevant journal for any special instructions relating to CIF submission.

PLATON version of 04/06/2025; check.def file version of 30/05/2025

Datablock pcrx1037_pcrb1037_a - ellipsoid plot

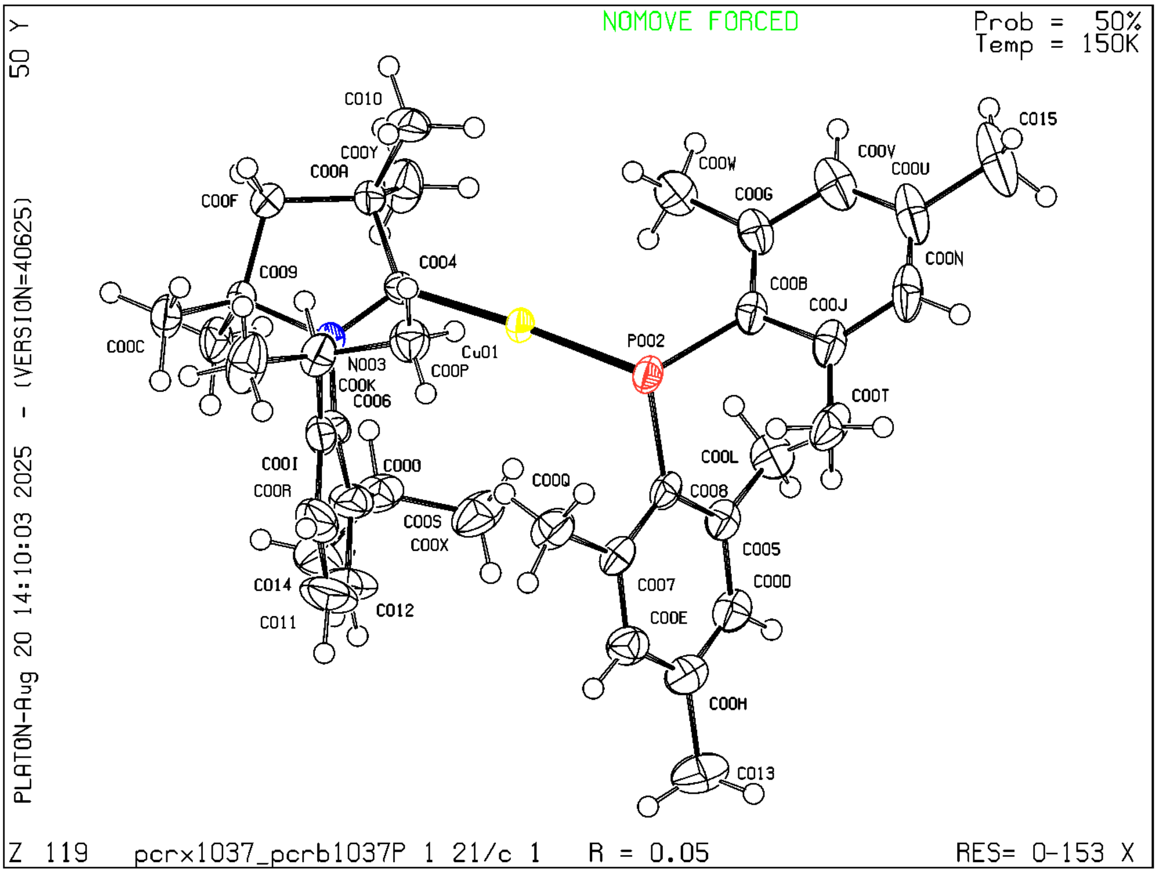

Supplement: Supplementary file 4 — Supporting File 4: anie71068‐sup‐0004‐Data.zip. [file ANIE-65-e18530-s001.zip › checkcif-PCRX1037_PCRB1037_a-finalcif.pdf]
