# Supplementary material for: Organophosphides: A New Class of Luminophore Ligands for Copper(I) Carbene Based TADF Emitters and Photocatalysts
Source: Angew Chem Int Ed Engl. 2026 Feb 25;65(13):e18530. doi: 10.1002/anie.202518530 (PMC13007583; doi:10.1002/anie.202518530)

checkCIF/PLATON report

Structure factors have been supplied for datablock(s) ACA_PCRX1065_PCRB1043_sq

THIS REPORT IS FOR GUIDANCE ONLY. IF USED AS PART OF A REVIEW PROCEDURE FOR PUBLICATION, IT SHOULD NOT REPLACE THE EXPERTISE OF AN EXPERIENCED CRYSTALLOGRAPHIC REFEREE.

No syntax errors found.   CIF dictionary   Interpreting this report

Datablock: ACA_PCRX1065_PCRB1043_sq

Bond precision:	C-C = 0.0043 A		Wavelength=1.54178
Cell:	a=12.2041(4)	b=20.6661(7)	c=27.1497(8)
	alpha=90	beta=101.317(1)	gamma=90
Temperature:	100 K		

	Calculated	Reported
Volume	6714.3(4)	6714.3(4)
Space group	P 21	P 21
Hall group	P 2yb	P 2yb
Moiety formula	3(C45 H65 Cu N P), C5 H12 [+ solvent]	?
Sum formula	C140 H207 Cu3 N3 P3 [+ solvent]	C46.67 H69 Cu N P
Mr	2215.65	738.53
Dx,g cm-3	1.096	1.096
Z	2	6
Mu (mm-1)	1.230	1.230
F000	2400.0	2400.0
F000'	2394.89	
h,k,lmax	15,26,34	15,26,34
Nref	29177[15002]	28280
Tmin,Tmax	0.933,0.972	0.674,0.754
Tmin'	0.878	

Correction method= # Reported T Limits: Tmin=0.674 Tmax=0.754
AbsCorr = MULTI-SCAN

Data completeness= 1.89/0.97   Theta(max)= 79.381

R(reflections)= 0.0303(27227)

wR2(reflections)= 0.0819(28280)

S = 1.017 Npar= 1519

The following ALERTS were generated. Each ALERT has the format
test-name_ALERT_alert-type_alert-level.
Click on the hyperlinks for more details of the test.

Alert level C

```
PLAT220_ALERT_2_C NonSolvent   Resd 1  C   Ueq(max)/Ueq(min) Range        3.3 Ratio
PLAT220_ALERT_2_C NonSolvent   Resd 2  C   Ueq(max)/Ueq(min) Range        3.6 Ratio
PLAT260_ALERT_2_C Large Average Ueq of Residue Including    C1_10A      0.130 Check
PLAT260_ALERT_2_C Large Average Ueq of Residue Including    C1_11B      0.154 Check
PLAT410_ALERT_2_C Short Intra H...H Contact  H27      ..H34B     .       1.99 Ang.
                                                    x,y,z  =      1_555 Check
PLAT911_ALERT_3_C Missing FCF Refl Between Thmin & STh/L=    0.600         7 Report
                0  2  0, -11 16  8,   2  0  9,  10  2 14,  10  0 15,  10  2 16,
                8  3 18,
```

Alert level G

```
PLAT002_ALERT_2_G Number of Distance or Angle Restraints on AtSite         57 Note
PLAT003_ALERT_2_G Number of Uiso or U(i,j) Restrained non-H Atoms         163 Report
PLAT045_ALERT_1_G Calculated and Reported Z Differ by a Factor ...      0.333 Check
PLAT172_ALERT_4_G The CIF-Embedded .res File Contains DFIX Records         24 Report
PLAT173_ALERT_4_G The CIF-Embedded .res File Contains DANG Records         32 Report
PLAT174_ALERT_4_G The CIF-Embedded .res File Contains FLAT Records         20 Report
PLAT175_ALERT_4_G The CIF-Embedded .res File Contains SAME Records          1 Report
PLAT178_ALERT_4_G The CIF-Embedded .res File Contains SIMU Records          1 Report
PLAT187_ALERT_4_G The CIF-Embedded .res File Contains RIGU Records          1 Report
PLAT232_ALERT_2_G Hirshfeld Test Diff (M-X)  Cu1_4    --C1       .        5.5 s.u.
PLAT232_ALERT_2_G Hirshfeld Test Diff (M-X)  Cu1_1    --P1_2     .        5.3 s.u.
PLAT232_ALERT_2_G Hirshfeld Test Diff (M-X)  Cu1_1    --C15_3    .        6.9 s.u.
PLAT301_ALERT_3_G Main Residue  Disorder .............(Resd    1)        19% Note
PLAT302_ALERT_4_G Anion/Solvent/Minor-Residue Disorder (Resd    4)       100% Note
PLAT302_ALERT_4_G Anion/Solvent/Minor-Residue Disorder (Resd    5)       100% Note
PLAT304_ALERT_4_G Non-Integer Number of Atoms in ..... (Resd    4)       9.23 Check
PLAT304_ALERT_4_G Non-Integer Number of Atoms in ..... (Resd    5)       7.77 Check
PLAT328_ALERT_4_G Possible Missing H on sp3? Phosphorus ..........       P1_5 Check
PLAT328_ALERT_4_G Possible Missing H on sp3? Phosphorus ..........       P1_2 Check
PLAT328_ALERT_4_G Possible Missing H on sp3? Phosphorus ..........         P2 Check
PLAT413_ALERT_2_G Short Inter XH3 .. XHn     H55A     ..H1B_10A  .       2.07 Ang.
                                                  x,1+y,z  =      1_565 Check
PLAT605_ALERT_4_G Largest Solvent Accessible VOID in the Structure        129 A**3
PLAT791_ALERT_4_G Model has Chirality at C12        (Sohncke SpGr)          S Verify
PLAT791_ALERT_4_G Model has Chirality at C4B_3      (Sohncke SpGr)          S Verify
PLAT791_ALERT_4_G Model has Chirality at C26        (Sohncke SpGr)          S Verify
PLAT791_ALERT_4_G Model has Chirality at C30        (Sohncke SpGr)          R Verify
PLAT791_ALERT_4_G Model has Chirality at C16_3      (Sohncke SpGr)          R Verify
PLAT791_ALERT_4_G Model has Chirality at C20A_3     (Sohncke SpGr)          R Verify
PLAT791_ALERT_4_G Model has Chirality at C63        (Sohncke SpGr)          R Verify
PLAT791_ALERT_4_G Model has Chirality at C64        (Sohncke SpGr)          R Verify
PLAT791_ALERT_4_G Model has Chirality at C73        (Sohncke SpGr)          R Verify
PLAT794_ALERT_5_G Tentative Bond Valency for Cu1_1     (I)       .       0.57 Info
```

```
PLAT794_ALERT_5_G Tentative Bond Valency for Cu1_6      (I)       .       0.59 Info
PLAT860_ALERT_3_G Number of Least-Squares Restraints ............       2766 Note
PLAT869_ALERT_4_G ALERTS Related to the Use of SQUEEZE  Suppressed         ! Info
PLAT883_ALERT_1_G No Info/Value for _atom_sites_solution_primary .     Please Do !
PLAT910_ALERT_3_G Missing # of FCF Reflection(s) Below Theta(Min).         1 Note
                0  0  1,
PLAT912_ALERT_4_G Missing # of FCF Reflections Above STh/L=  0.600       161 Note
PLAT969_ALERT_5_G The 'Henn et al.' R-Factor-gap value ..........      2.722 Note
              Predicted wR2: Based on SigI**2  3.00 or SHELX Weight  8.05
PLAT978_ALERT_2_G Number C-C Bonds with Positive Residual Density.         3 Info
PLAT992_ALERT_5_G Repd & Actual _reflns_number_gt Values Differ by         3 Check
```

```
   0 ALERT level A = Most likely a serious problem - resolve or explain
   0 ALERT level B = A potentially serious problem, consider carefully
   6 ALERT level C = Check. Ensure it is not caused by an omission or oversight
  41 ALERT level G = General information/check it is not something unexpected

   2 ALERT type 1 CIF construction/syntax error, inconsistent or missing data
  12 ALERT type 2 Indicator that the structure model may be wrong or deficient
   4 ALERT type 3 Indicator that the structure quality may be low
  25 ALERT type 4 Improvement, methodology, query or suggestion
   4 ALERT type 5 Informative message, check
```

It is advisable to attempt to resolve as many as possible of the alerts in all categories. Often the minor alerts point to easily fixed oversights, errors and omissions in your CIF or refinement strategy, so attention to these fine details can be worthwhile. In order to resolve some of the more serious problems it may be necessary to carry out additional measurements or structure refinements. However, the purpose of your study may justify the reported deviations and the more serious of these should normally be commented upon in the discussion or experimental section of a paper or in the "special_details" fields of the CIF. checkCIF was carefully designed to identify outliers and unusual parameters, but every test has its limitations and alerts that are not important in a particular case may appear. Conversely, the absence of alerts does not guarantee there are no aspects of the results needing attention. It is up to the individual to critically assess their own results and, if necessary, seek expert advice.

Publication of your CIF in IUCr journals

A basic structural check has been run on your CIF. These basic checks will be run on all CIFs submitted for publication in IUCr journals (*Acta Crystallographica*, *Journal of Applied Crystallography*, *Journal of Synchrotron Radiation*); however, if you intend to submit to *Acta Crystallographica Section C* or *E* or *IUCrData*, you should make sure that full publication checks are run on the final version of your CIF prior to submission.

Publication of your CIF in other journals

Please refer to the *Notes for Authors* of the relevant journal for any special instructions relating to CIF submission.

PLATON version of 13/05/2024; check.def file version of 04/05/2024

Datablock ACA_PCRX1065_PCRB1043_sq - ellipsoid plot

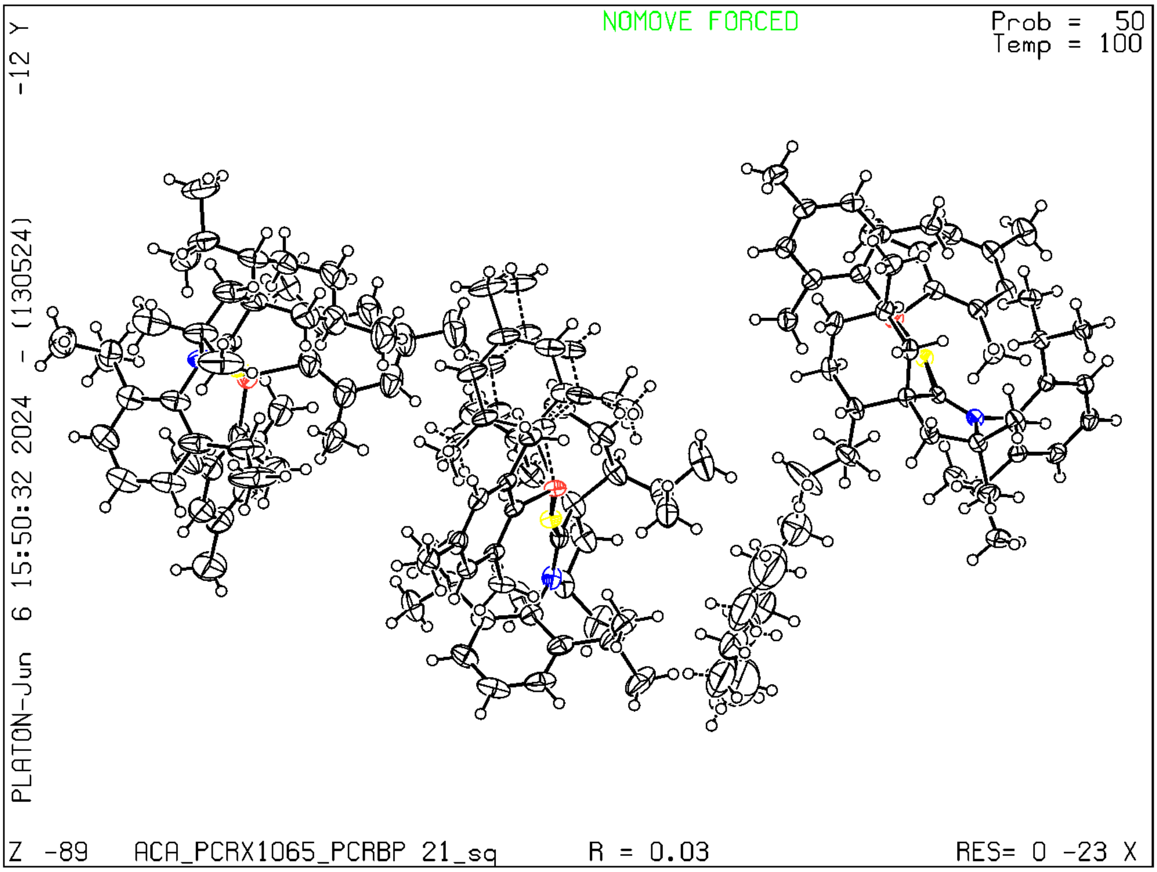

Supplement: Supplementary file 4 — Supporting File 4: anie71068‐sup‐0004‐Data.zip. [file ANIE-65-e18530-s001.zip › checkcif-PCRX1065_PCRB1043-finalcif.pdf]
