# Supplementary material for: Organophosphides: A New Class of Luminophore Ligands for Copper(I) Carbene Based TADF Emitters and Photocatalysts
Source: Angew Chem Int Ed Engl. 2026 Feb 25;65(13):e18530. doi: 10.1002/anie.202518530 (PMC13007583; doi:10.1002/anie.202518530)

checkCIF/PLATON report

Structure factors have been supplied for datablock(s) aca_pcrx1209_pcrb1051

THIS REPORT IS FOR GUIDANCE ONLY. IF USED AS PART OF A REVIEW PROCEDURE FOR PUBLICATION, IT SHOULD NOT REPLACE THE EXPERTISE OF AN EXPERIENCED CRYSTALLOGRAPHIC REFEREE.

No syntax errors found.   CIF dictionary   Interpreting this report

Datablock: aca_pcrx1209_pcrb1051

```
Bond precision:    C-C = 0.0060 A          Wavelength=1.54178

Cell:              a=16.4599(7)     b=5.8055(2)        c=33.8351(13)
                   alpha=90         beta=99.688(3)     gamma=90
Temperature:       100 K

                 Calculated                  Reported
Volume           3187.1(2)                   3187.1(2)
Space group      P 21/n                      P 1 21/n 1
Hall group       -P 2yn                      -P 2yn
Moiety formula   C20 H17 O P                 C20 H17 O P
Sum formula      C20 H17 O P                 C20 H17 O P
Mr               304.31                      304.30
Dx,g cm-3        1.268                       1.268
Z                8                           8
Mu (mm-1)        1.504                       1.504
F000             1280.0                      1280.0
F000'            1285.52
h,k,lmax         20,7,41                     20,7,41
Nref             6073                        5973
Tmin,Tmax        0.973,0.984                 0.592,0.753
Tmin'            0.925

Correction method= # Reported T Limits: Tmin=0.592 Tmax=0.753
AbsCorr = NONE

Data completeness= 0.984           Theta(max)= 70.254

                                                      wR2(reflections)=
R(reflections)= 0.0749( 3784)                         0.1846( 5973)

S = 1.049                  Npar= 397
```

The following ALERTS were generated. Each ALERT has the format
test-name_ALERT_alert-type_alert-level.
Click on the hyperlinks for more details of the test.

Alert level C

```
RINTA01_ALERT_3_C  The value of Rint is greater than 0.12
            Rint given   0.161
PLAT020_ALERT_3_C The Value of Rint is Greater Than 0.12 .........      0.161 Report
PLAT340_ALERT_3_C Low Bond Precision on  C-C Bonds ..............    0.00602 Ang.
PLAT906_ALERT_3_C Large K Value in the Analysis of Variance ......     10.591 Check
PLAT911_ALERT_3_C Missing FCF Refl Between Thmin & STh/L=    0.600         71 Report
                 2   6  3,  -8  3  5,  -8  3  6,  -7  4  6,  -6  6  6,  -8  3  7,
                -8  4  7,  -7  4  7,  -2  6  7,  -8  3  8,  -8  4  8,  -8  4  9,
                -7  5  9,  -7  5 10,  -7  5 11,  -4  2 11,  -7  5 12,  -4  6 12,
                -7  5 13,  -8  5 14,  -7  5 14,  -5  6 14,  -8  5 15, -18  0 16,
                -8  5 16,  -4  6 16,  -5  6 17,  -4  6 17,  -6  0 18,  -5  6 18,
                    (   41 More Missing: see the .ckf listing file)
```

Alert level G

```
PLAT720_ALERT_4_G Number of Unusual/Non-Standard Labels ..........         78 Note
              P001    P002    O003    O004    C005    H005    C006    C007
              C008    H008    C009    H009    C00A    C00B    H00B    C00C
              H00C    C00D    C00E    H00E    C00F    C00G    C00H    H00H
              C00I    H00I    C00J    H00J    C00K    H00K    C00L    H00L
              C00M    H00M    C00N    H00N    C00O    H00O    C00P    H00P
              C00Q    H00Q    C00R    H00R    C00S    H00S    C00T    H00T
              C00U    H00U    C00V    H00V    C00W    H00W    C00X    H00X
              C00Y    H00Y    C00Z    H00Z    C010    H010    C011    H011
              C012    H012    C013    H013    C014    H014    C015    H015
              C016    H016    C017    H017    C018    H018
PLAT912_ALERT_4_G Missing # of FCF Reflections Above STh/L=  0.600         29 Note
PLAT933_ALERT_2_G Number of HKL-OMIT Records in Embedded .res File          1 Note
               -4  2 11,
PLAT969_ALERT_5_G The 'Henn et al.' R-Factor-gap value ..........      1.851 Note
              Predicted wR2: Based on SigI**2  9.98 or SHELX Weight 17.61
PLAT978_ALERT_2_G Number C-C Bonds with Positive Residual Density.          0 Info
```

0 **ALERT level A** = Most likely a serious problem - resolve or explain
0 **ALERT level B** = A potentially serious problem, consider carefully
5 **ALERT level C** = Check. Ensure it is not caused by an omission or oversight
5 **ALERT level G** = General information/check it is not something unexpected

0 ALERT type 1 CIF construction/syntax error, inconsistent or missing data
2 ALERT type 2 Indicator that the structure model may be wrong or deficient
5 ALERT type 3 Indicator that the structure quality may be low
2 ALERT type 4 Improvement, methodology, query or suggestion
1 ALERT type 5 Informative message, check

It is advisable to attempt to resolve as many as possible of the alerts in all categories. Often the minor alerts point to easily fixed oversights, errors and omissions in your CIF or refinement strategy, so attention to these fine details can be worthwhile. In order to resolve some of the more serious problems it may be necessary to carry out additional measurements or structure refinements. However, the purpose of your study may justify the reported deviations and the more serious of these should normally be commented upon in the discussion or experimental section of a paper or in the "special_details" fields of the CIF. checkCIF was carefully designed to identify outliers and unusual parameters, but every test has its limitations and alerts that are not important in a particular case may appear. Conversely, the absence of alerts does not guarantee there are no aspects of the results needing attention. It is up to the individual to critically assess their own results and, if necessary, seek expert advice.

Publication of your CIF in IUCr journals

A basic structural check has been run on your CIF. These basic checks will be run on all CIFs submitted for publication in IUCr journals (*Acta Crystallographica*, *Journal of Applied Crystallography*, *Journal of Synchrotron Radiation*); however, if you intend to submit to *Acta Crystallographica Section C* or *E* or *IUCrData*, you should make sure that full publication checks are run on the final version of your CIF prior to submission.

Publication of your CIF in other journals

Please refer to the *Notes for Authors* of the relevant journal for any special instructions relating to CIF submission.

PLATON version of 04/06/2025; check.def file version of 30/05/2025

Datablock aca_pcrx1209_pcrb1051 - ellipsoid plot

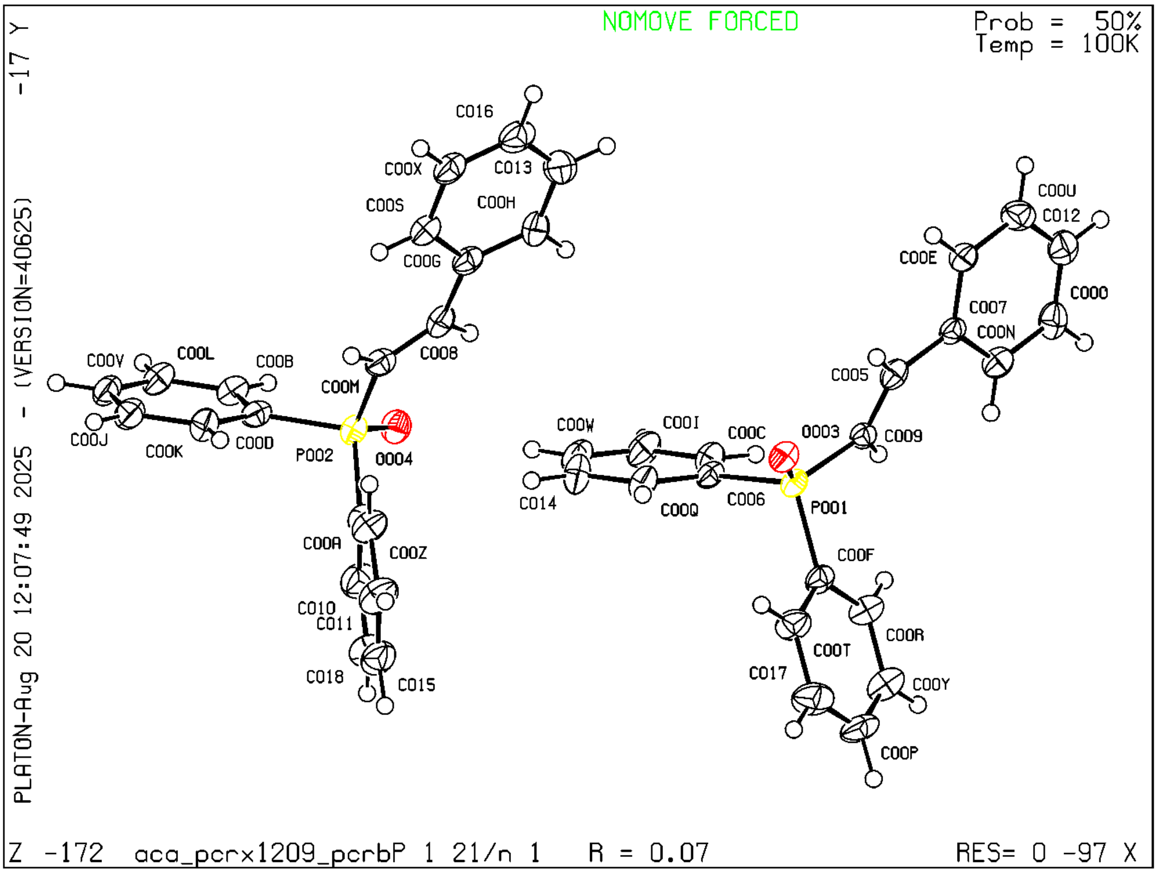

Supplement: Supplementary file 4 — Supporting File 4: anie71068‐sup‐0004‐Data.zip. [file ANIE-65-e18530-s001.zip › checkcif-PCRX1209_PCRB1051-finalcif.pdf]
