# Supplementary material for: Organophosphides: A New Class of Luminophore Ligands for Copper(I) Carbene Based TADF Emitters and Photocatalysts
Source: Angew Chem Int Ed Engl. 2026 Feb 25;65(13):e18530. doi: 10.1002/anie.202518530 (PMC13007583; doi:10.1002/anie.202518530)

checkCIF/PLATON report

Structure factors have been supplied for datablock(s) pcrx1012_pcrb1024_a

THIS REPORT IS FOR GUIDANCE ONLY. IF USED AS PART OF A REVIEW PROCEDURE FOR PUBLICATION, IT SHOULD NOT REPLACE THE EXPERTISE OF AN EXPERIENCED CRYSTALLOGRAPHIC REFEREE.

No syntax errors found.   CIF dictionary   Interpreting this report

Datablock: pcrx1012_pcrb1024_a

```
Bond precision:   C-C = 0.0021 A           Wavelength=0.71073

Cell:             a=12.6056(6)     b=17.6043(8)        c=17.3103(9)
                  alpha=90         beta=111.236(2)     gamma=90
Temperature:      100 K

                 Calculated                   Reported
Volume           3580.5(3)                    3580.5(3)
Space group      P 21/c                       P 1 21/c 1
Hall group       -P 2ybc                      -P 2ybc
Moiety formula   C40 H57 Cu N P               C40 H57 Cu N P
Sum formula      C40 H57 Cu N P               C40 H57 Cu N P
Mr               646.39                       646.37
Dx,g cm-3        1.199                        1.199
Z                4                            4
Mu (mm-1)        0.682                        0.682
F000             1392.0                       1392.0
F000'            1394.03
h,k,lmax         18,25,25                     18,25,25
Nref             11932                        11924
Tmin,Tmax        0.874,0.942                  0.705,0.747
Tmin'            0.837

Correction method= # Reported T Limits: Tmin=0.705 Tmax=0.747
AbsCorr = NONE

Data completeness= 0.999           Theta(max)= 31.498

                                                      wR2(reflections)=
R(reflections)= 0.0399( 9689)                         0.0985( 11924)

S = 1.039                 Npar= 402
```

The following ALERTS were generated. Each ALERT has the format
test-name_ALERT_alert-type_alert-level.
Click on the hyperlinks for more details of the test.

Alert level C

```
PLAT906_ALERT_3_C Large K Value in the Analysis of Variance ......      2.114 Check
PLAT911_ALERT_3_C Missing FCF Refl Between Thmin & STh/L=    0.600           7 Report
                0  2  0,   1  2  0,   0  2  1,  -1  0  2,   0  0  2,  -1  1  2,
                0  1  2,
```

Alert level G

```
PLAT232_ALERT_2_G Hirshfeld Test Diff (M-X)  Cu01     --P002     .        7.3 s.u.
PLAT328_ALERT_4_G Possible Missing H on sp3? Phosphorus .........       P002 Check
PLAT720_ALERT_4_G Number of Unusual/Non-Standard Labels .........         97 Note
              Cu01    P002    N003    C004    C005    C006    C007    C008
              C009    C00A    C00B    C00C    H00C    C00D    H00A    H00B
              H00D    C00E    C00F    C00G    H00E    H00F    H00G    C00H
              C00I    C00J    H00H    H00I    C00K    C00L    H00L    C00M
              H00M    C00N    H00J    H00K    H00N    C00O    H00O    H00P
              H00Q    C00P    H00R    H00S    C00Q    H00T    C00R    H00U
              H00V    C00S    H00W    C00T    H00X    C00U    C00V    H00Y
              C00W    H00Z    C00X    H00     Ha      C00Y    Hb      Hc
              C00Z    Hd      He      C010    H010    C011    H01A    H01B
              H01C    C012    H01D    H01E    H01F    C013    H01G    H01H
              H01I    C014    H01J    H01K    H01L    C015    H01M    H01N
              H01O    C016    H01P    H01Q    H01R    C017    H01S    H01T
              H01U
PLAT794_ALERT_5_G Tentative Bond Valency for Cu01      (I)       .       0.60 Info
PLAT910_ALERT_3_G Missing FCF Reflection(s) Below Theta(Min)[Deg]=       2.09 Note
                1  0  0,   0  1  1,
PLAT913_ALERT_3_G Missing # of Very Strong Reflections in FCF ....          1 Note
                0  1  2,
PLAT967_ALERT_5_G Note: Two-Theta Cutoff Value in Embedded .res ..       63.0 Degree
PLAT969_ALERT_5_G The 'Henn et al.' R-Factor-gap value ..........       3.583 Note
              Predicted wR2: Based on SigI**2  2.75 or SHELX Weight  9.48
PLAT978_ALERT_2_G Number C-C Bonds with Positive Residual Density.         21 Info
PLAT992_ALERT_5_G Repd & Actual _reflns_number_gt Values Differ by          2 Check
```

0 **ALERT level A** = Most likely a serious problem - resolve or explain
0 **ALERT level B** = A potentially serious problem, consider carefully
2 **ALERT level C** = Check. Ensure it is not caused by an omission or oversight
10 **ALERT level G** = General information/check it is not something unexpected

0 ALERT type 1 CIF construction/syntax error, inconsistent or missing data
2 ALERT type 2 Indicator that the structure model may be wrong or deficient
4 ALERT type 3 Indicator that the structure quality may be low
2 ALERT type 4 Improvement, methodology, query or suggestion
4 ALERT type 5 Informative message, check

It is advisable to attempt to resolve as many as possible of the alerts in all categories. Often the minor alerts point to easily fixed oversights, errors and omissions in your CIF or refinement strategy, so attention to these fine details can be worthwhile. In order to resolve some of the more serious problems it may be necessary to carry out additional measurements or structure refinements. However, the purpose of your study may justify the reported deviations and the more serious of these should normally be commented upon in the discussion or experimental section of a paper or in the "special_details" fields of the CIF. checkCIF was carefully designed to identify outliers and unusual parameters, but every test has its limitations and alerts that are not important in a particular case may appear. Conversely, the absence of alerts does not guarantee there are no aspects of the results needing attention. It is up to the individual to critically assess their own results and, if necessary, seek expert advice.

Publication of your CIF in IUCr journals

A basic structural check has been run on your CIF. These basic checks will be run on all CIFs submitted for publication in IUCr journals (*Acta Crystallographica*, *Journal of Applied Crystallography*, *Journal of Synchrotron Radiation*); however, if you intend to submit to *Acta Crystallographica Section C* or *E* or *IUCrData*, you should make sure that full publication checks are run on the final version of your CIF prior to submission.

Publication of your CIF in other journals

Please refer to the *Notes for Authors* of the relevant journal for any special instructions relating to CIF submission.

PLATON version of 04/06/2025; check.def file version of 30/05/2025

Datablock pcrx1012_pcrb1024_a - ellipsoid plot

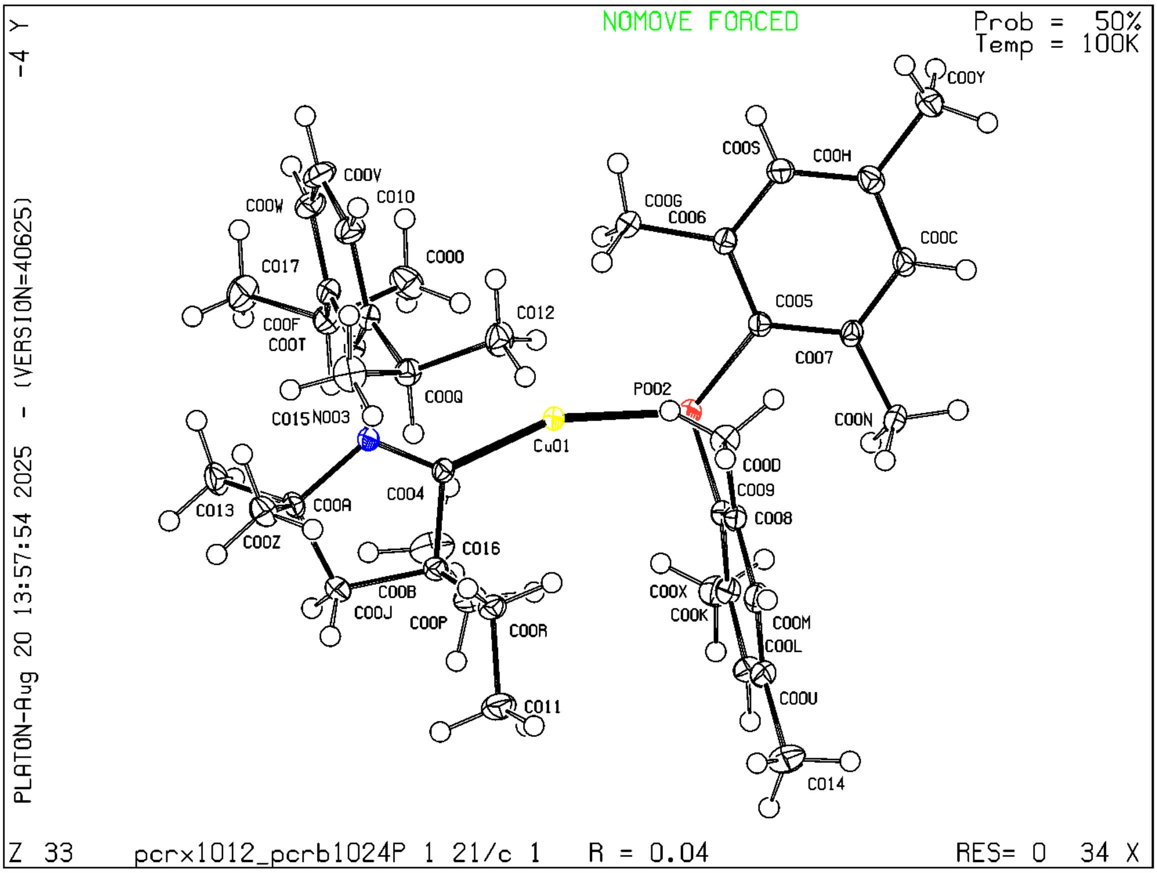

Supplement: Supplementary file 4 — Supporting File 4: anie71068‐sup‐0004‐Data.zip. [file ANIE-65-e18530-s001.zip › checkcif-PCRX1012_PCRB1024_a-finalcif.pdf]
